# Social bonds are related to health behaviors and positive well-being globally

**DOI:** 10.1126/sciadv.add3715

**Published:** 2023-01-13

**Authors:** Bahar Tunçgenç, Valerie van Mulukom, Martha Newson

**Affiliations:** ^1^ Department of Psychology, Nottingham Trent University, Nottingham, UK.; ^2^ Institute of Human Sciences, University of Oxford, Oxford, UK.; ^3^ Centre for Trust, Peace and Social Relations, Coventry University, Coventry, UK.; ^4^ Centre for the Study of Social Cohesion, University of Oxford, Oxford, UK.; ^5^ School of Anthropology and Conservation, University of Kent, Canterbury, UK.

## Abstract

At times of turmoil, such as during disasters, social crises, or pandemics, our social bonds can be key to receiving support and gaining certainty about the right course of action. In an analysis combining two global datasets (*N* = 13,264) collected during the first wave of the COVID-19 pandemic, this study examined how social bonds with close social circles (i.e., family and friends) and extended groups (i.e., country, government, and humanity) relate to engagement in health behaviors and psychological well-being. Results revealed that only family bonding was associated with self-reported engagement in health behaviors. Being strongly bonded with both close circles and extended groups predicted less anxiety and depression and better well-being, particularly for those who were bonded with more groups. These findings highlight that close and extended social bonds offer different sources of support and direction during the most challenging of circumstances and that continuous investment is needed to forge and maintain both.

## Teaser

Only family bonds relate to health behaviours, while bonds with family, friends, country or humanity relate to positive wellbeing.

## INTRODUCTION

“No man is an island,” as the 16th century poet, John Donne, said. Our connection to the rest of humankind has been at the forefront of our minds in recent years, made prominent by the COVID-19 (coronavirus disease 2019) pandemic. Akin to observed social outcomes of other social crises or disasters (*1*–*3*), the pandemic initially created feelings of community (e.g., clapping for the National Health Service in the United Kingdom or communal singing on balconies in Italy) and opportunities to unite more strongly with close social circles of family and friends, as well as with extended groups such as one’s country. Still, repeated, lengthy lockdowns during the pandemic led to increased social isolation and distress for many people (*4*–*6*), and reports of growing prejudice, hostility toward outside groups, and sociopolitical polarization abounded in the first year of the pandemic (*7*, *8*).

The pandemic offered opportunities to forge community connections while simultaneously elevating worries around physical and mental health (*9*). Considering these contrasting influences, here, we investigate what role social bonds play in guiding people’s health behaviors and psychological well-being. We conducted a combined analysis of two global-scale datasets gathered at the start of the COVID-19 pandemic to examine how bonds with close social circles (i.e., family and friends) and extended groups (i.e., country, government, and humanity) are associated with engagement in pandemic-related health behaviors, mental health (i.e., anxiety and depression), and overall psychosocial well-being.

### The human need for bonding with close circles and extended groups

Humans are an innately social species, evolved to rely on interacting and bonding with other people for childcare (*10*), resources (*11*), and buffering against stress (*12*). The complex human societies most of us find ourselves in today are not immune from this need for deep social bonds: From sprawling cities comprising millions of individuals, people tend to seek out social circles of a handful of individuals whom they come to rely on (*13*–*15*). Beyond close social circles, humans are also uniquely able to bond with extended groups, including nonkin and people they have never met before (*16*, *17*). This extended bonding (e.g., as seen in patriotism or sports fandom) is based around symbols and markers of group identity and allows individuals to recognize their shared allegiance when they could not possibly maintain relationships with every individual in an extended group.

Whether it be with a small circle of friends or with extended groups, such as one’s country, forming intense social bonds is entrenched in our evolutionary history. Intense social bonds are characterized by strong, and often mutual, feelings of affiliation, trust, commitment, and willingness to engage in self-sacrificial altruistic behavior for the benefit of the other (*18*–*20*). Such intense social bonding can be captured by the concept and measure of identity fusion (*21*), which refers to a strong form of social cohesion, whereby group membership becomes a central part of one’s identity, that is, one’s group and personal identities become irrevocably “fused” (*21*, *22*).

### Social bonding and health behaviors

Powerful social bonds with close and extended groups have important consequences for many behaviors (*18*, *22*, *23*) and may also confer substantial physical and mental health benefits in times of stress. During the COVID-19 pandemic, individuals tended to follow the behavior of their close social network rather than the behavior that they perceived to occur in the rest of their country or the wider world (*24*), which, in turn, promoted well-being (*25*). Research into both disaster psychology and health psychology offers a wealth of evidence demonstrating how social bonds with close circles of friends and family provide support and direction during challenging times. For instance, during tornadoes or fire emergencies, people wait for and look up to loved ones to decide how to behave and whether to flee or not (*3*). When critical life events occur, such as a heart failure (*26*) or stroke (*27*), people are more likely to make healthy lifestyle changes if they have deep bonds with their close social circles.

A panel study in the U.K. shows, for instance, that strong identification with the local community before the pandemic was associated with increased likelihood of providing pandemic-related support to neighbors and adherence to rules (*28*). Studies with more varied cultural samples showed that even more extended bonds, i.e., to the country, predicted increased public health support (*29*) and better perceived quality of life (*30*). These findings exemplify that deep bonds with extended groups nurture people’s social needs, just as bonds with close social circles do (*31*). This approach aligns with the Social Cure Model, which purports that a deep sense of belonging to, or identifying with, groups can positively affect people’s health, well-being, and coping with adverse circumstances (*32*).

### Social bonding and psychological well-being

Surveys conducted at the start of the COVID-19 pandemic have revealed an initial rise in anxiety and depressive symptoms and a decline in overall well-being (*6*, *33*–*36*). However, the data are mixed as to the longevity of this observed initial decline and as to which specific symptoms need closer attention in relation to public health interventions (*37*, *38*). World Health Organization’s definition captures the view that mental health is not the absence of a mental illness but is “a state of well-being in which the individual realizes his or her own abilities, can cope with the normal stresses of life, can work productively and fruitfully, and is able to make a contribution to his or her community” (*39*). Accordingly, positive mental health and well-being have a subjective/emotional and a social/cognitive component, with poor well-being not necessarily indicating the presence of mental health symptoms (*40*, *41*). Thus, to gain a comprehensive understanding of the determinants of psychological health, it is important to examine both common mental health symptoms and overall well-being (*42*).

Research into social identities and the Social Cure Model suggest that having multiple group identities can boost resilience and encourage health behaviors or norms (*27*, *43*). When making real-life decisions on how to adapt behaviors in response to changing circumstances, individuals are under the simultaneous influences of the multiple groups that they are bonded with. How varied forms of intense bonding with close and extended groups affect behavior and help people navigate threatening or uncertain situations has been largely overlooked in previous studies. Still, one study with a relatively small U.K. sample has provided evidence that family, country, and humanity bonds may have different effects on pandemic-related actions and well-being (*44*). 

### Current study

Using two large datasets that examined bonding with both close social circles (i.e., family and friends) and extended groups (i.e., country, government, and humanity) in the first months following the outbreak of the COVID-19 pandemic in 2020, this study examined the effects of both bonding (versus no bonding) and bonding with multiple groups on pandemic-related health behaviors and psychological well-being. We recognize that although subject pools in psychological research have diversified somewhat in the past decade, the Northern Hemisphere (specifically North America and North-Western Europe) is still substantially overrepresented (*45*, *46*). Much research thus speaks to the psychology underlying WEIRD (Western, educated, industrialized, rich, and democratic) participants (*45*) rather than the human condition more broadly. We address this by including participants from a total of 122 countries (see Materials and Methods), with samples of greater than 100 participants in three countries from the Global South (i.e., Bangladesh, Brazil, and Peru). The need to belong and connect with others is a human universal (*11*, *13*, *16*), but the ways in which individuals enact their relationships is culturally variable. It is thus important to consider how social bonds can buffer against negative physical and mental health effects from a more global perspective.

We hypothesized that (Hypothesis 1A) being intensely bonded versus not being intensely bonded and (Hypothesis 1B) being intensely bonded to multiple groups would be associated with more self-reported engagement in pandemic-related health behaviors. Moreover, (Hypothesis 2A) being intensely bonded versus not being intensely bonded and (Hypothesis 2B) being intensely bonded to multiple groups would be associated with better mental health and well-being. . In both datasets used in this study to examine these hypotheses, pre-established measures of intense social bonding, mental health, and well-being were used (see Materials and Methods for details). All analysis scripts, datasets, and results are available on the project’s Open Science Framework page: https://doi.org/10.17605/OSF.IO/BGZUF.

## RESULTS

### Intense social bonding with family and with more groups predict engagement with pandemic-related health behaviors

**Fig. 1.**
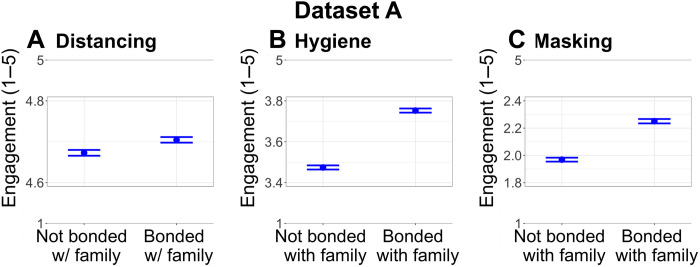
Significant associations between bonding with family and pandemic-related health behaviors. Family bonding is related with higher engagement in (**A**) distancing, (**B**) hygiene, and (**C**) mask wearing in dataset A. In all graphs, the horizontal bars show SEMs.

Dataset A analysis provided support for Hypothesis 1A by revealing that being bonded with family (versus not being bonded) predicted increased engagement with all pandemic-related health behaviors (**Distancing**: model *R*^2^ = 0.43, β = 0.09, SE = 0.03, *F*_1,6452_ *=* 8.87, η_p_*^2^* = 0.002, *P* = 0.003; **Hygiene**: model *R*^2^ = 0.11, β = 0.31, SE = 0.03, *F*_1,6436_ *=* 116.88, η_p_*^2^* = 0.001, *P* < 0.0001; **Masking**: model *R*^2^ = 0.19, β = 0.12, SE = 0.03, *F*_1,6443_ *=* 19.26, η_p_*^2^* = 0.004, *P* < 0.0001; Fig. 1). No associations were found between any of the other bonding variables and pandemic-related health behaviors [all *P* > 0.05; see Supplementary Results and table S5 (A and B)].

**Fig. 2.**
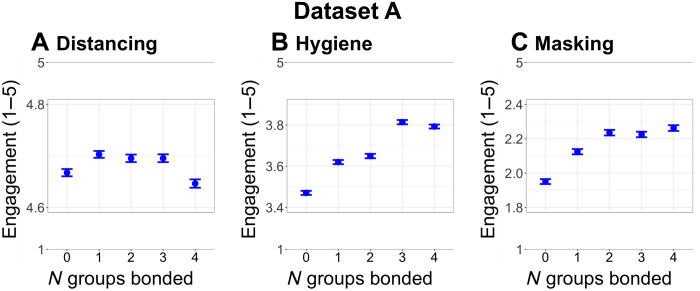
Significant associations between bonding with multiple groups and pandemic-related health behaviors. Bonding with more groups is related with higher engagement in (**A**) distancing, (**B**) hygiene, and (**C**) mask wearing in dataset A. In all graphs, the horizontal bars show SEMs.

Dataset A analysis also provided support for Hypothesis 1B by revealing that being bonded with multiple groups was associated with more engagement in both hygiene and masking health behaviors but not distancing behavior (**Hygiene**: model *R*^2^ = 0.11; zero versus one group: β = 0.18, SE = 0.03; zero versus two groups: β = 0.21, SE = 0.04; zero versus three groups: β = 0.38, SE = 0.07; zero versus four groups: β = 0.31, SE = 0.11, *F*_4,6448_ *=* 19.60, η_p_*^2^* = 0.02, *P* < 0.0001; **Masking**: zero versus one group: β = 0.06, SE = 0.03; zero versus two groups: β = 0.12, SE = 0.05; zero versus three groups and zero versus four groups: not significant, *F*_4,6455_ *=* 2.63, η_p_*^2^* = 0.002, *P* = 0.03; Fig. 2). Bonding with multiple groups was not associated with health behaviors in dataset B [see Supplementary Results and table S6 (A and B)]. This discrepancy is likely because the two bonding targets assessed in dataset B were extended groups (i.e., country and government), with only a small minority of participants (5%) being bonded to both their country and their government. In contrast, in dataset A, both close circles and extended groups (i.e., family, friends, country, and humanity) were assessed, and 15% of participants were bonded with at least two targets.

### Intense social bonding is associated with better mental health and well-being

**Fig. 3.**
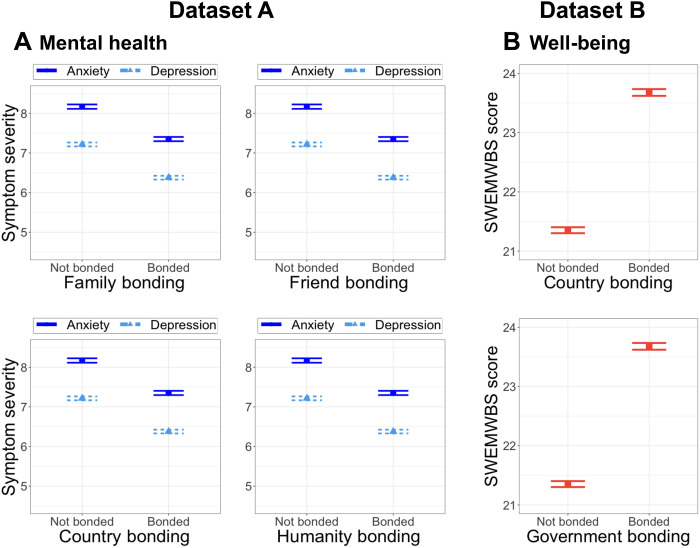
Significant associations between bonding and mental health and well-being. Bonding with all targets is related to (**A**) better mental health in dataset A (left, blue) and (**B**) positive well-being in dataset B (right, orange). In all graphs, horizontal bars show SEMs.

Analyses from both datasets A and B supported Hypothesis 2A by revealing that being bonded (versus not being bonded) with all targets was positively associated with better mental health and well-being, respectively (**Dataset A anxiety**: family, β = −0.09, SE = 0.03, *F*_1,6463_ *=* 10.63, η_p_*^2^* = 0.004, *P* = 0.001; friends, β = −0.11, SE = 0.04, *F*_1,6463_ *=* 7.61, η_p_*^2^* = 0.0007, *P* = 0.006; country, β = −0.19, SE = 0.06, *F*_1,6463_ *=* 12.08, η_p_*^2^* = 0.003, *P* = 0.0005; humanity, β = −0.06, SE = 0.03, *F*_1,6463_ *=* 3.98, η_p_*^2^* = 0.0005, *P* = 0.05; **Dataset A depression**: family, β = −0.17, SE = 0.03, *F*_1, 6465_ *=* 27.46, η_p_*^2^* = 0.01, *P* < 0.0001; friends, β = −0.26, SE = 0.04, *F*_1,6465_ *=* 36.55, η_p_*^2^* = 0.06, *P* < 0.0001; country, β = −0.15, SE = 0.05, *F*_1,6465_ *=* 8.49, η_p_*^2^* = 0.003, *P* = 0.004; humanity, β = −0.14, SE = 0.03, *F*_1,6465_ *=* 19.38, η_p_*^2^* = 0.003, *P* < 0.0001; Fig. 3A; **Dataset B well-being**: country, β = 0.27, SE = 0.05, *F*_1,6164_ *=* 29.82, η_p_*^2^* = 0.01, *P* < 0.0001; government, β = 0.25, SE = 0.06, *F*_1,6164_ *=* 18.83, η_p_^2^ = 0.004, *P* < 0.0001; Fig. 3B).

**Fig. 4.**
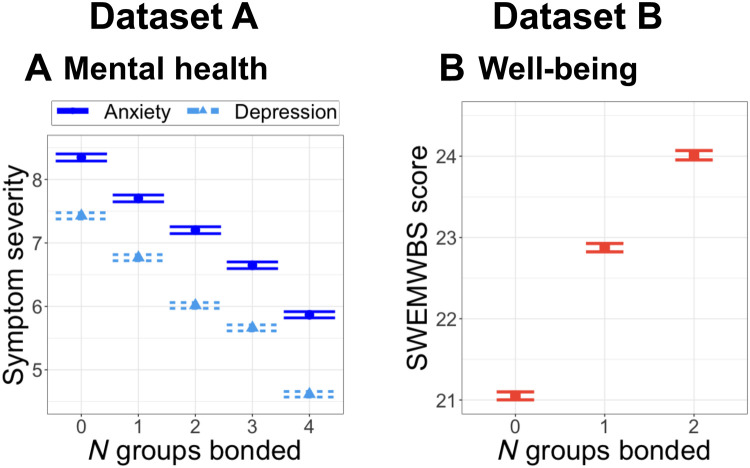
Significant associations between bonding with multiple groups and mental health and well-being. Bonding with more groups is related with (**A**) better mental health in dataset A (left, blue) and (**B**) positive well-being in dataset B (right, orange). In all graphs, horizontal bars show SEMs.

Similarly, analyses of both datasets A and B supported Hypothesis 2B by revealing that being bonded with multiple groups was associated with less anxiety (**Dataset A anxiety**: model *R*^2^ = 0.13, zero versus one group: β = −0.13, SE = 0.03; zero versus two groups: β = −0.21, SE = 0.04; zero versus three groups: β = −0.30, SE = 0.06; zero versus four groups: β = −0.38, SE = 0.10, *F*_4,6475_ *=* 15.65, η*_p_^2^* = 0.01, *P* < 0.0001; Fig. 4A), less depression (**Dataset A depression**: zero versus one group: β = −0.20, SE = 0.03; zero versus two groups: β = −0.39, SE = 0.04; zero versus three groups: β = −0.45, SE = 0.06; zero versus four groups: β = −0.68, SE = 0.11, *F*_4,6476_ *=* 42.23, η_p_*^2^* = 0.03, *P* < 0.0001; Fig. 4A), and better well-being (**Dataset B well-being**: zero versus one group: β = 0.26, SE = 0.05; zero versus two groups: β = 0.51, SE = 0.06, *F*_2,6381_ *=* 43.40, η_p_*^2^* = 0.02, *P* < 0.0001; Fig. 4B).

## DISCUSSION

In a global sample, we examined how intense bonding with close (i.e., family and friends) and extended groups (i.e., country, government, and humanity) was associated with self-reported engagement with pandemic-related health behaviors (i.e., keeping physical distance, handwashing, and mask wearing) and psychological well-being during the early months of the COVID-19 pandemic. Results provide evidence that only bonding with family, but not other groups, is linked to greater engagement with pandemic-related health behaviors. Furthermore, the results suggest that bonding of all sorts, both with close social circles and with extended groups, is associated with better mental health and well-being. We also found that intense bonding with a greater number of groups is associated with more engagement in health behaviors (except for distancing) and with better mental health and well-being. These findings show that bonding with close circles and external groups pose complementary yet distinct benefits, in support of previous research (*24*, *25*, *27*, *43*).

Several mechanisms may help explain how bonding is linked with health behaviors and psychological well-being. Group norms are likely to play a crucial role in the strength and direction of this relationship (*47*). Specifically, bonding is likely to promote behaviors that align with the group’s norms or values, an effect that may be particularly prominent where identity fusion is concerned because of the synergy between personal and group identities (*21*, *24*). The varied norms that different social networks may have held could explain the fact that bonding with multiple groups was not associated with enhanced distancing behavior. In addition to encouraging health behaviors through norms, as per the Social Cure Model, social group membership can positively affect physical and mental health through providing support, a sense of meaning and opportunities to enhance self-esteem, and perceived control (*48*). Relatedly, identity fusion theory posits that individuals who are fused to their group are willing to engage in personally costly behaviors because of the relational ties principle, i.e., they view others in the group as unique individuals who resemble kin rather than interchangeable group members (*21*). Future work should invest in designs that go beyond cross-sectional correlations to unpick the mechanisms responsible for the effects of social bonding on health behaviors, mental health, and well-being.

To examine the robustness of our findings, we conducted several supplementary analyses (please see Materials and Methods). Supplementary longitudinal analyses confirmed that bonding at time-1 was predictive of mental health and well-being at time-2, whereas such a longitudinal effect was not found for health behaviors. Previous research has revealed mixed results on whether close connections or identification with one’s country is linked to pandemic-related health behaviors (*29*) or not (*33*). In our main analysis and supplementary analyses using models including only country bonding as the predictor variable, we found no evidence that country bonding is associated with health behaviors. This discrepancy across studies suggests how nuanced differences in the way country bonding is measured can yield divergent conclusions. In particular, our measure comprised a single pictorial item, while Van Bavel *et al.* (*29*) used several verbal items. Moreover, this study found significant country-level differences in the percentage of participants who were bonded with their country and in how bonding related to health behaviors and psychological well-being. Hence, a broader range of possible external factors that may affect bonding and its links with collectivism/individualism, conformity to social norms, and other sociocultural values need to be considered when evaluating the robustness of social bonding effects in multiple contexts.

Using geographically and demographically diverse samples that include less WEIRD countries is a key strength of this research. Still, the results are constrained by the nonrepresentative nature of our sample. In addition, this research used self-reported measures. Although such measures may suffer from limitations such as social desirability or memory biases, self-reported behaviors have been found to convincingly mirror real-world behavior during the pandemic, when direct observation of others’ behaviors has been challenging or impossible. For instance, individuals who reported more social distancing were found to have a significantly greater step reduction via a smartphone app than those who reported little social distancing (*49*). Nonetheless, caution should be taken when applying this research to policy, and we advise triangulating these findings with experiments that use actual behaviors and representative samples.

The results of this study indicate that public messaging aiming to promote health behaviors could focus on smaller networks and bonds with multiple groups (*44*). More specifically, crisis or emergency communications may benefit from focusing on the advantages conferred to families (or similarly influential small groups), and policy or campaigns could be most influential when they encourage individuals to share their protective health behaviors within their close social circles (*24*). Leveraging smaller networks, particularly the family, for larger-scale interventions is likely to be successful (*28*), but it means taking the time to work with grassroots organizations and individuals (*50*). Such initiatives must be handled with care, with insight from social scientists and practitioners with detailed knowledge of the community and intergroup dynamics, who can foresee potential issues such as defensive, hostile, or xenophobic behaviors oft associated with intensely bonded groups, particularly toward threatening out-groups (*23*). It is also worth examining how people’s bondedness with different networks (e.g., family versus country) shifts in times of crisis (*9*). These socially positive small groups may then be a starting point from which to engage with more extended groups (*1*).

The clear message of this study is that to provide rapid psychosocial support at times of societal change, we must leverage social connections at all levels. Broadening our social bonds, particularly intense social bonds, which this research focused on, is not an automatic fix. Rather, social prescribing within health systems (*51*) and funding shared social spaces and celebrations of diverse identities will help solidify bonds to varied groups, which sustain psychological health (*52*). For instance, healthcare systems can use social prescribing to reduce the reliance on pharmaceutical treatment, especially when what may be missing in an individual’s life are the supportive and directive buffers that come from social bonds to a close group, such as a family. Similarly, organizations seeking to improve their workers’ well-being might consider group bonding exercises that tap into more extended groups, even stretching to humanity and the connections maintained to identities beyond the workplace. With regard to both the health behavior and well-being results, our data speak to such a moment in time that dwarfed others in emotional intensity and magnitude of shift in psychological states. Ultimately, if society wants a chance at buffering people against the physical and emotional impacts of global crises, policy-makers and other influential stakeholders need to continually invest in social bonds.

## MATERIALS AND METHODS

### Participants

#### Dataset A

**Fig. 5.**
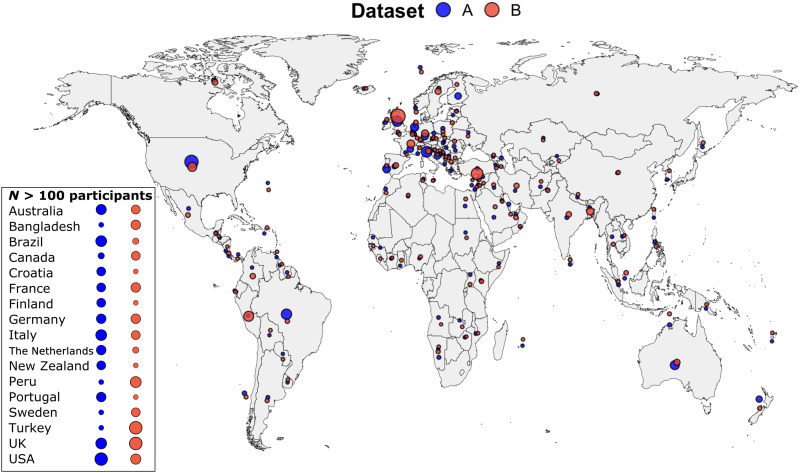
Distribution of participants across countries. The box highlights countries from which at least one dataset (dataset A, blue; dataset B, orange) had *N* > 100 participants, with circle size reflecting number of participants.

Participants were 6589 adults (58% women and 40% men; see table SI1) from 74 countries (Fig. 5, blue dots). Participants completed an online survey hosted by Qualtrics in one of eight languages (Brazilian Portuguese, Dutch, German, English, French, Italian, Portuguese, and Spanish), depending on their preference, between 28 March 2020 and 24 April 2020. The study was approved by the ethics committee at Coventry University, U.K.

#### Dataset B

Participants were 6675 adults (65% women and 33% men; see table SI1) from 115 countries (Fig. 5, orange dots). Participants completed an online survey built using jsPsych in 1 of 12 languages (Arabic, Bangla, English, French, German, Hindi, Italian, Mandarin, Persian, Spanish, Swedish, and Turkish), depending on their preference, between 09 April 2020 and 24 May 2020. Follow-up surveys, not analyzed here except for the supplementary longitudinal analyses, were sent to participants fortnightly. The study was approved by the School of Psychology Ethics Committee of the University of Nottingham, U.K.

For both datasets, data were gathered through snowball and volunteer sampling, and the only inclusion criterion was age (minimum 18 years for dataset A and 16 years for dataset B). Advertisements were shared via the authors’ social networks, media channels (e.g., local radios and TV channels), institutional press releases, and social media (e.g., international COVID-19 groups on Facebook, Reddit, and Twitter). These datasets have been previously used in the publication of other studies answering different research questions (*24*, *25*, *53*). Please see Supplementary Results for full descriptive statistics on the number of participants in demographic categories (table S1) and number of participants per country (table S2).

### Measures

#### Social bonding

##### Dataset A

**Fig. 6.**
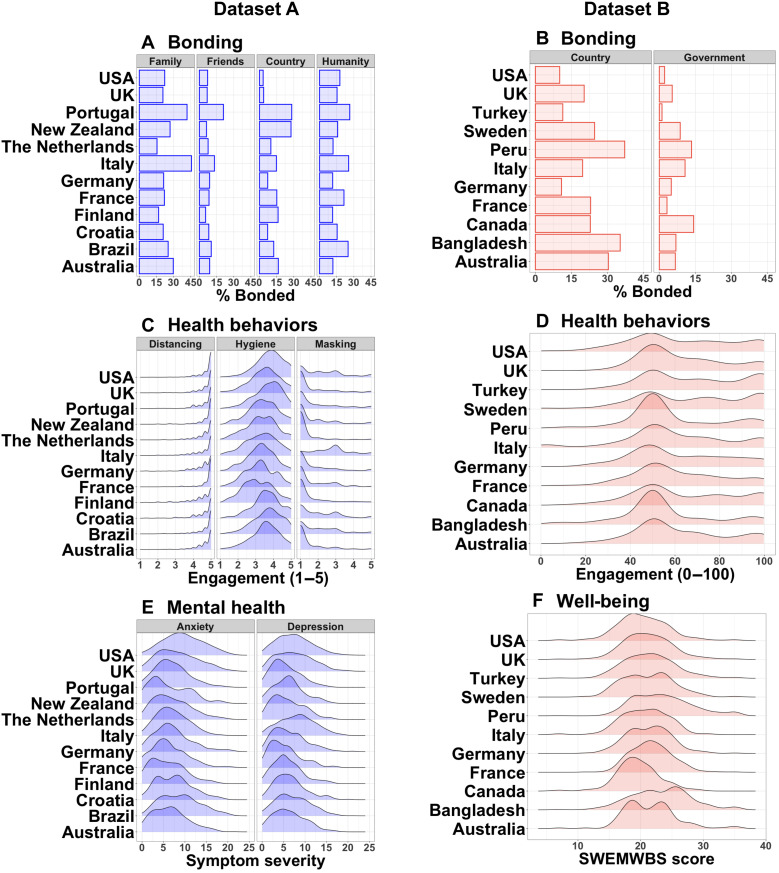
Descriptive data of key variables within countries with a sample size of *N* > 100. Data distributions in both datasets (dataset A, blue; dataset B, orange) showing (**A** and **B**) percentage of people feeling bonded and density plots of (**C** and **D**) health behaviors, (**E**) mental health, and (**F**) well-being. Country names appear in reverse-alphabetical order.

Social bonding with family, friends, country of residence, and humanity were assessed (see Fig. 6A for data distributions) using a well-established pictorial measure widely used in the identity fusion literature (*22*) adapted from the Inclusion of Self in Other scale, with the final option being total immersion in the group (*5*). As shown in table SI2, the bonding measure presented participants with five options. Each option depicted two circles, one representing self and the other representing the group under question, with the two circles getting gradually closer to each other across options until they fully overlap. Following scale scoring guidelines, participants received a score of 1 if they selected the fully overlapping option (i.e., they were intensely bonded to the group) and a score of 0 otherwise (i.e., not intensely bonded). This measure was dichotomized to ensure that the cases considered as “bonded” were perceived to be inseparably close to each other.

##### Dataset B

Social bonding with the country of current residence and the government of that country were assessed (see Fig. 6B for data distributions) using the same five-point pictorial measure as in dataset A (see table SI2).

### Pandemic-related health behaviors

#### Dataset A

A total of 13 items across three categories as determined by a principal component analysis (*55*) were used (see table SI2): distancing (three items, e.g., “Stay at home”), hygiene behaviors (five items, e.g., “Wash hands for minimum 20 seconds”), and mask wearing (two items, e.g., “Wear a mask of any kind”). For each item, participants indicated how much they had performed that action in the past week on a 5-point scale ranging from “1= Not at all” to “5= As much as possible.” The items comprising each type of pandemic-related health behavior were averaged to obtain the score of that variable, yielding a range of 1 to 5 (see Fig. 6C for data distributions).

#### Dataset B

Participants rated how much they had followed the general advice of “keeping physical distance from others” in the past week on a continuous scale with options ranging from “0= Not been following the advice at all” through “50= Been following the advice exactly” to “100= Been doing more than is what advised” (see table SI2; see Fig. 6D for data distributions).

### Mental health and well-being

#### Dataset A

Mental health was measured using the Hospital Anxiety and Depression Scale, as shown in table SI2 (*56*). This scale comprises seven items on anxiety symptoms (e.g., “I get sudden feelings of panic”) and seven on depressive symptoms (e.g., “I look forward with enjoyment to things,” reverse item), with varied answer options across items that indicate symptom severity. Each item could receive a score between 0 and 3. Following scale scoring guidelines, participants’ anxiety and depressive symptom scores were calculated by summing up the scores of the seven items comprising each scale, with the total scale scores ranging between 0 and 21 (see Fig. 6E for data distributions) and higher scores indicating more severe symptoms and poorer mental health.

#### Dataset B

A seven-item measure of overall well-being, the short Warwick-Edinburgh Mental Well-being Scale, which has been validated and translated into many languages (*57*), was used (see table SI2). Each item (e.g., “I’ve been feeling optimistic about the future” and “I’ve been feeling useful”) was rated on a 5-point scale ranging from “None of the time” to “All of the time”. Following scale scoring guidelines (*58*), participant scores, which ranged between 7 and 35, were converted into a metric score (see Fig. 6F for data distributions), with higher scores indicating better well-being.

### Statistical analyses

Mixed-effects multiple linear regression models were conducted using the nlme, lme4, and lmerTest packages in R statistical software (*59*–*62*). The outcome variables used for Hypotheses 1A and 1B were self-reported distancing, hygiene, and masking behaviors in dataset A and self-reported distancing behavior in dataset B. The outcome variables used for Hypotheses 2A and 2B were self-reported anxiety and depression symptoms in dataset A and self-reported well-being in dataset B.

We analyzed intense bonding using the well-established identity fusion measure and, as per the literature (*21*), dichotomized the variable with respect to its more extreme nature. In models that displayed heteroscedasticity, we identified new variance structures using the varIdent() command within lme() to allow for unequal variances for the different levels of the bonding variables (i.e., more people in the not bonded versus bonded categories). In Hypotheses 1A and 2A models, the fixed-effects variables were the dichotomous bonding variables (dataset A fixed effects: bonding to family, friend, country, or humanity; dataset B fixed effects: bonding to country or government). In Hypotheses 1B and 2B models, the fixed-effect variable was the categorical variable of the number of groups with which the participants were intensely bonded (dataset A range, 0 to 4; dataset B range, 0 to 2). In all models, restricted maximum likelihood was used; outcome variables were scaled; age, gender, education, and country’s gross domestic product (GDP) per capita in 2020 (*63*) were covariates; and country was the random intercept. Data for GDP per capita were not available for a total of 10 participants who responded to the country question (Guernsey, *n* = 2; Jersey, *n* = 1; Syria, *n* = 2; Taiwan, *n* = 3; Yugoslavia, *n* = 2). For full model outputs of main analyses, see tables S5 to S8.

In addition, several supplementary analyses were conducted to check the robustness of our findings. First, we endeavored to allow for bonding to vary across countries by adding it as a random slope; however, because of singularity, many models did not converge. For models that did converge, findings were unchanged [see table S9 (A and B)]. Second, we re-ran Hypotheses 1A and 2A models using the continuous version of the bonding variables. The findings largely held, with the only discrepancy being significant associations emerging between country and humanity bonding and some of the health behaviors [see table S10 (A and B)]. Third, to ensure that the results were not biased by countries with large samples, all analyses were repeated with datasets composed only of countries with more than 100 participants (*n* = 11 countries per dataset); all but one finding held [i.e., multiple group bonding was no longer associated with distancing in dataset B; see table S11 (A and B)]. Fourth, because of moderate zero-order correlations observed between country bonding and other bonding variables, we re-ran the Hypotheses 1A and 2A models using country bonding as the only fixed-effect variable; all findings held [see table S12 (A and B)]. Lastly, using data from different time points in both datasets, we examined the directionality of the hypothesized associations. These analyses revealed that the findings presented here largely held at multiple time points over a 6-month period by bonding at time-1 predicting mental health and well-being at time-2 [see table S13 (A and B)], although no longitudinal effects were found for health behaviors. All analysis scripts, datasets, and results are available on the project’s Open Science Framework page (https://doi.org/10.17605/OSF.IO/BGZUF).

## Supplementary Materials

### This PDF file includes:

Tables SI1 and SI2 Descriptive Statistics Main analyses: Complete outputs of models reported in the paper Supplementary Analyses Table S1 to S13 Data Preparation

## Notes

View/request a protocol for this paper from *Bio-protocol*.
