# Supplementary material for: Social bonds are related to health behaviors and positive well-being globally
Source: Sci Adv. 2023 Jan 13;9(2):eadd3715. doi: 10.1126/sciadv.add3715 (PMC10957100; doi:10.1126/sciadv.add3715)
Supplement: Supplementary file 1 — Tables SI1 and SI2 Descriptive Statistics Main analyses: Complete outputs of models reported in the paper Supplementary Analyses Table S1 to S13 Data Preparation [file sciadv.add3715_sm.pdf]

Science Advances
AAAS

Supplementary Materials for

Social bonds are related to health behaviors and positive well-being globally

Bahar Tunçgenç *et al.*

Corresponding author: Bahar Tunçgenç, bahartuncgenc@gmail.com; Martha Newson, m.newson@kent.ac.uk

Sci. Adv. **9**, eadd3715 (2023)
DOI: 10.1126/sciadv.add3715

This PDF file includes:

Tables SI1 and SI2
Descriptive Statistics
Main analyses: Complete outputs of models reported in the paper
Supplementary Analyses
Table S1 to S13
Data Preparation

Supplementary Information

Table SI1. Sociodemographic characteristics of the samples of both datasets at T1

		Dataset A	Dataset B
**Age**	**16–24 years** (min. age in Dataset A = 18)	960	1505
	**25–34 years**	2194	2093
	**35–44 years**	1505	1310
	**45–54 years**	943	812
	**55–64 years**	679	633
	**65–74 years**	264	264
	**75–90 years** (max. age in Dataset A = 81)	30	58
**Gender**	**Male / Man**	2672	2204
	**Female / Woman**	3854	4356
	**Non-binary / Other**	61	59
	**Not disclosed**	2	56
**Household**	**Solo**	792	818
	**Cohabiting**	5797	5857
**Education (highest level attained)**	**No formal education up to secondary school**	1717	1102
	**Undergraduate / Occupational degree**	2864	1071
	**Postgraduate (i.e., Master's or Doctoral degree)**	1955	3096
	**Not disclosed**	53	N/A
**Work/study status**	**Active (i.e., has employment and/or study)**	N/A	5532
	**Inactive (i.e., no employment or study)**	N/A	1143
**Household income**	**Far below average**	483	N/A
	**Below average**	1184	N/A
	**Average**	1770	N/A
	**Above average**	2397	N/A
	**Far above average**	563	N/A
	**Not disclosed**	182	N/A
	**TOTAL**	**6589**	**6675**

Table SI2. Survey items used to measure bonding, engagement in health behaviours, mental health and wellbeing in both datasets.

<table>
<tr><th></th><th>Dataset A</th><th>Dataset B</th></tr>
<tr><td rowspan="2">Bonding measure</td><td colspan="2">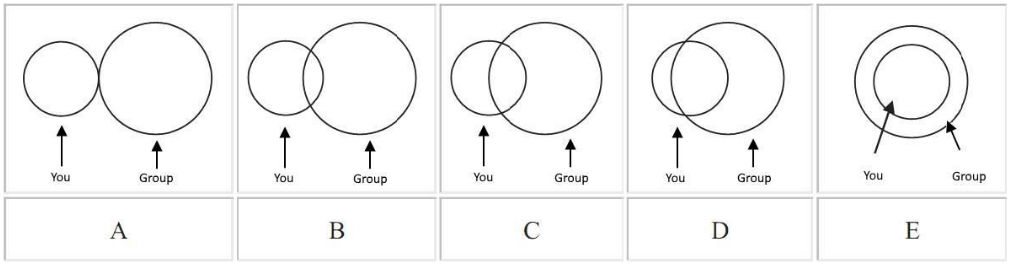
</td></tr>
<tr><td>Group = Family, friends, country of residence, humanity</td><td>Group = Country of residence, government of country of residence</td></tr>
<tr><td>Health behaviours</td><td>In the past week, how much have you made it a priority to…
Distancing:
“Stay home”
“Avoid shaking hands with other people”
“Avoid any physical contact with other people”
Hygiene:
“Wash your hands”
“Wash your hands for at least 20 seconds”
“Clean and disinfect your home”
“Use antibacterial products”
“Not touch your face”
Masking:
“Wear a mask of any kind”
“Wear an N-95 or higher (health grade) mask”
Response options: 0 = Not at all, 1 = A little bit, 2 = Some, 3 = A lot, 4 = As much as possible</td><td>Distancing:
The general advice for the coronavirus disease (COVID-19) is to keep physical distance from others. Think about YOUR OWN actions and beliefs IN THE PAST 7 DAYS. Consider how this advice has been implemented where you currently live.
“I have been following this general advice where I live…”
Response options: 0 = Not been following the advice at all, 50 = Been following the advice exactly, 100 = Been doing more than what is advised.</td></tr>
<tr><td>Mental Health & Wellbeing</td><td>Anxiety:
“I feel tense or 'wound up'”
“I get a sort of frightened feeling as if something awful is about to happen”
“Worrying thoughts go through my mind”
“I can sit at ease and feel relaxed”
“I get a sort of frightened feeling like 'butterflies' in the stomach”
“I feel restless as if I have to be on the move”
“I get sudden feelings of panic”</td><td>Wellbeing:
“I’ve been feeling optimistic about the future”
“I’ve been feeling useful”
“I’ve been feeling relaxed”
“I’ve been dealing with problems well”
“I’ve been thinking clearly”
“I’ve been feeling close to other people”
“I’ve been able to make up my own mind about things”</td></tr>
</table>

	**Depression**:<br>“I still enjoy the things I used to enjoy”<br>“I can laugh and see the funny side of things”<br>“I feel cheerful”<br>“I feel as if I am slowed down”<br>“I have lost interest in my appearance”<br>“I look forward with enjoyment to things”<br>“I can enjoy a good book or radio or TV programme”<br><br>**Response options**: Varies from item to item. Please refer to the original scale for details on scoring: The Hospital Anxiety and Depression Scale (Zigmond & Snaith,1983).	**Response options**: 1 = None of the time, 2 = Rarely, 3 = Some of the time, 4 = Often, 5 = All of the time<br>Short version of the Warwick-Edinburgh Mental Wellbeing Scale (WEMWBS) *(58)*

Supplementary Results: Social bonding boosts health behaviour and psychological wellbeing

Contents

Data Preparation **3**

Dataset A . . . 4

Dataset B . . . 6

Descriptive Statistics **8**

Table S1: Number of participants in demographic and bonding categories . . . 8

Dataset A . . . 8

Dataset B . . . 9

Table S2: Number of participants per country . . . 10

Dataset A . . . 10

Dataset B . . . 10

Table S3: Zero-order correlations among key variables . . . 10

Dataset A . . . 10

Dataset B . . . 12

Table S4: Descriptive statistics of key variables in N > 100 countries and in the entire sample . . . 13

Dataset A, N>100 countries . . . 13

Dataset A, entire sample . . . 16

Dataset B, N>100 countries . . . 27

Dataset B, entire sample . . . 68

Main Analyses: Complete outputs of models reported in the paper **109**

Study hypotheses . . . 109

Table S5A: Hypothesis 1a, Dataset A: Does bonding (vs no bonding) predict health behaviours? . 109

Hypothesis 1a, Dataset A models . . . 109

Hypothesis 1a, Dataset A model check for assumptions and fit . . . 115

Table S5B: Hypothesis 1a, Dataset B: Does bonding (vs no bonding) predict health behaviours? . 120

Hypothesis 1a, Dataset B model . . . 120

Hypothesis 1a, Dataset B model check for assumptions and fit . . . 122

Table S6A: Hypothesis 1b, Dataset A: Does bonding with multiple groups predict health behaviours?126

Hypothesis 1b, Dataset A model . . . 126
Hypothesis 1b, Dataset A model check for assumptions and fit . . . 131
Table S6B: Hypothesis 1b, Dataset B: Does bonding with multiple groups predict health behaviours? 133
Hypothesis 1b, Dataset B model . . . 133
Hypothesis 1b, Dataset B model check for assumptions and fit . . . 135
Table S7A: Hypothesis 2a, Dataset A: Does bonding (vs no bonding) predict mental health? . . . 136
Hypothesis 2a, Dataset A model . . . 136
Hypothesis 2a, Dataset A model check for assumptions and fit . . . 141
Table S7B: Hypothesis 2a, Dataset B: Does bonding (vs no bonding) predict wellbeing? . . . 150
Hypothesis 2a, Dataset B model . . . 150
Hypothesis 2a, Dataset B model check for assumptions and fit . . . 152
Table S8A: Hypothesis 2b, Dataset A: Does bonding with multiple groups predict mental health? . 156
Hypothesis 2b, Dataset A model . . . 156
Hypothesis 2b, Dataset A model check for assumptions and fit . . . 159
Table S8B: Hypothesis 2b, Dataset B: Does bonding with multiple groups predict wellbeing? . . . 164
Hypothesis 2b, Dataset B model . . . 164
Hypothesis 2b, Dataset B model check for assumptions and fit . . . 166

Supplementary Analyses **167**
Table S9A: Bonding variables as random slopes, Dataset A . . . 167
Hypothesis 1a, Dataset A . . . 167
Hypothesis 1b, Dataset A . . . 168
Hypothesis 2a, Dataset A . . . 168
Hypothesis 2b, Dataset A . . . 168
Table S9B: Bonding variables as random slopes, Dataset B . . . 169
Hypothesis 1a, Dataset B . . . 169
Hypothesis 1b, Dataset B . . . 170
Hypothesis 2a, Dataset B . . . 170
Hypothesis 2b, Dataset B . . . 172
Table S10A: Continuous bonding variables, Dataset A . . . 172
Hypothesis 1a, Dataset A . . . 172
Hypothesis 2a, Dataset A . . . 176
Table S10B: Continuous bonding variables, Dataset B . . . 179
Hypothesis 1a, Dataset B . . . 179
Hypothesis 2a, Dataset B . . . 181
Table S11A: Main analyses only within N>100 countries, Dataset A . . . 182
Hypothesis 1a, Dataset A . . . 182

Hypothesis 1b, Dataset A 187
Hypothesis 2a, Dataset A 193
Hypothesis 2b, Dataset A 196
Table S11B: Main analyses only within N>100 countries, Dataset B 200
Hypothesis 1a, Dataset B 200
Hypothesis 1b, Dataset B 201
Hypothesis 2a, Dataset B 203
Hypothesis 2b, Dataset B 205
Table S12A: Only country bonding as fixed-effect, Dataset A 207
Hypothesis 1a, Dataset A 208
Hypothesis 2a, Dataset A 211
Table S12B: Only country bonding as fixed-effect, Dataset B 214
Hypothesis 1a, Dataset B 214
Hypothesis 2a, Dataset B 215
Table S13A: Longitudinal comparisons, Dataset A 216
Hypothesis 1a, Dataset A 216
Hypothesis 1b, Dataset A 219
Hypothesis 2a, Dataset A 222
Hypothesis 2b, Dataset A 228
Table S13B: Longitudinal comparisons, Dataset B 233
Hypothesis 1a, Dataset B 233
Hypothesis 1b, Dataset B 236
Hypothesis 2a, Dataset B 238
Hypothesis 2b, Dataset B 241

Data Preparation

These data files can be accessed at the project's OSF page: https://doi.org/10.17605/OSF.IO/BGZUF.

```
# Load libraries
library("Hmisc")
library("rmarkdown")
library("tidyr")
library("tidyverse")
library("effectsize")
library("directlabels")
library("foreign")
library("ggnewscale")
library("geosphere")
library("scales")
library("nlme")
```

```r
library("lme4")
library("lmerTest")
library("readr")
library("performance")
library("car")
library("report")
library("fontawesome")

rm(list=ls())


# Load data
DatasetA_raw<-read.spss("DatasetA_bonding_pandemic_OSF.sav", to.data.frame=T)
DatasetB_raw<-read.csv("DatasetB_bonding_pandemic_OSF.csv")

ppt_data<- read.csv("Country_ppt_numbers.csv")
gdp_data <- read.csv("Country_gdpcapita.csv")
```

Dataset A

```r
# Scale variables and organise Dataset A
DatasetA_raw$Country_Name<- gsub("'", "", DatasetA_raw$Country_Name)
DatasetA_raw$Country_Name<- gsub('\\s{2,}','',DatasetA_raw$Country_Name)

DatasetA_raw$GDP<-rep(NA,nrow(DatasetA_raw))
for (i in gdp_data$Country) {
DatasetA_raw$GDP[which((str_detect(DatasetA_raw$Country_Name,i)))] <-
  gdp_data$GDPcap[which(gdp_data$Country==i)]
}

DatasetA_raw$bond_family<-rep(NA,nrow(DatasetA_raw))
DatasetA_raw$bond_family[which(DatasetA_raw$Fusion_Family1 !=  5)]<- 0
DatasetA_raw$bond_family[which(DatasetA_raw$Fusion_Family1 ==  5)]<- 1
DatasetA_raw$bond_friend<-rep(NA,nrow(DatasetA_raw))
DatasetA_raw$bond_friend[which(DatasetA_raw$Fusion_Friends1 !=  5)]<- 0
DatasetA_raw$bond_friend[which(DatasetA_raw$Fusion_Friends1 ==  5)]<- 1
DatasetA_raw$bond_country<-rep(NA,nrow(DatasetA_raw))
DatasetA_raw$bond_country[which(DatasetA_raw$Fusion_Country1 !=  5)]<- 0
DatasetA_raw$bond_country[which(DatasetA_raw$Fusion_Country1 ==  5)]<- 1
DatasetA_raw$bond_humanity<-rep(NA,nrow(DatasetA_raw))
DatasetA_raw$bond_humanity[which(DatasetA_raw$Fusion_Humanity1 !=  5)]<- 0
DatasetA_raw$bond_humanity[which(DatasetA_raw$Fusion_Humanity1 ==  5)]<- 1

DatasetA_raw$bond_mult<-rep(NA,nrow(DatasetA_raw))
bond_mult<-data.frame(cbind(DatasetA_raw$bond_family,DatasetA_raw$bond_friend,
                            DatasetA_raw$bond_country,DatasetA_raw$bond_humanity))
DatasetA_raw$bond_mult<- ifelse(apply(is.na(bond_mult),1,all),NA,rowSums(bond_mult,na.rm=T))
DatasetA_raw$bond_mult<-factor(DatasetA_raw$bond_mult)

DatasetA_raw$bond_family <- factor(DatasetA_raw$bond_family)
DatasetA_raw$bond_friend <- factor(DatasetA_raw$bond_friend)
DatasetA_raw$bond_country <- factor(DatasetA_raw$bond_country)
```

```r
DatasetA_raw$bond_humanity <- factor(DatasetA_raw$bond_humanity)

DatasetA_raw$Pro_Distancing_scaled<-scale(DatasetA_raw$Pro_Distancing,center=TRUE,scale=TRUE)
DatasetA_raw$Pro_Hygiene_scaled<-scale(DatasetA_raw$Pro_Hygiene,center=TRUE,scale=TRUE)
DatasetA_raw$Pro_MaskWearing_scaled<-scale(DatasetA_raw$Pro_MaskWearing,center=TRUE,scale=TRUE)

DatasetA_raw$anx_sum1_scaled<-scale(DatasetA_raw$anx_sum1,center=TRUE,scale=TRUE)
DatasetA_raw$dep_sum1_scaled<-scale(DatasetA_raw$dep_sum1,center=TRUE,scale=TRUE)

DatasetA_raw$Pro_Distancing_pos<- DatasetA_raw$Pro_Distancing+1
DatasetA_raw$Pro_Hygiene_pos<- DatasetA_raw$Pro_Hygiene+1
DatasetA_raw$Pro_MaskWearing_pos<- DatasetA_raw$Pro_MaskWearing+1

DatasetA_raw$demo_education <- make.names(DatasetA_raw$demo_education)
DatasetA_raw$demo_education[which(DatasetA_raw$demo_education
             =="No.schooling.completed..or.less.than.1.year")]<-"secondary"
DatasetA_raw$demo_education[which(DatasetA_raw$demo_education
             =="Nursery..kindergarten..and.primary.school")]<-"secondary"
DatasetA_raw$demo_education[which(DatasetA_raw$demo_education
             =="Some.secondary.school")]<-"secondary"
DatasetA_raw$demo_education[which(DatasetA_raw$demo_education
             =="Completed.secondary.school")]<-"secondary"
DatasetA_raw$demo_education[which(DatasetA_raw$demo_education
            =="College.A.levels")]<-"secondary"
DatasetA_raw$demo_education[which(DatasetA_raw$demo_education
             =="Associate.s.degree..including.occupational.or.academic.degrees.")]<-"undergraduate"
DatasetA_raw$demo_education[which(DatasetA_raw$demo_education
            =="Bachelor.s.degree..BA..BSc..AB..etc.")]<-"undergraduate"
DatasetA_raw$demo_education[which(DatasetA_raw$demo_education
            =="Master.s.degree..MA..MSc..MENG..MSW..etc.")]<-"postgraduate"
DatasetA_raw$demo_education[which(DatasetA_raw$demo_education
            =="Doctorate.degree..PhD..EdD..etc.")]<-"postgraduate"
DatasetA_raw$demo_education[which(DatasetA_raw$demo_education
            =="NA.")]<-"NA"
DatasetA_raw$demo_education<- factor(DatasetA_raw$demo_education,
            levels=c("secondary","undergraduate","postgraduate","NA"))

DatasetA_raw$demo_age_ten<-rep(NA,nrow(DatasetA_raw))
DatasetA_raw$demo_age_ten<-cut(DatasetA_raw$demo_age,c(
  min(DatasetA_raw$demo_age,na.rm=TRUE),24,34,44,54,64,74,
  max(DatasetA_raw$age,na.rm=TRUE)),include.lowest=TRUE)
DatasetA_raw$demo_age_ten<-factor(DatasetA_raw$demo_age_ten)

# T2 variables
DatasetA_raw$fuactions2<- factor(DatasetA_raw$fuactions2, labels = c("0", "1", "2", "3", "4"))
DatasetA_raw$fuactions2<- as.numeric(DatasetA_raw$fuactions2)
DatasetA_raw$fuactions3<- factor(DatasetA_raw$fuactions3, labels = c("0", "1", "2", "3", "4"))
DatasetA_raw$fuactions3<- as.numeric(DatasetA_raw$fuactions3)
DatasetA_raw$fuactions5<- factor(DatasetA_raw$fuactions5, labels = c("0", "1", "2", "3", "4"))
DatasetA_raw$fuactions5<- as.numeric(DatasetA_raw$fuactions5)
DatasetA_raw$Pro_Distancing2 <-rep(NA,nrow(DatasetA_raw))
DatasetA_raw$Pro_Distancing2 <- rowMeans(DatasetA_raw[,c("fuactions2", "fuactions3","fuactions5")], na.
DatasetA_raw$Pro_Distancing2_scaled<-scale(DatasetA_raw$Pro_Distancing2,center=TRUE,scale=TRUE)
```

```
DatasetA_raw$anx_sum2_scaled<-scale(DatasetA_raw$anx_sum2,center=TRUE,scale=TRUE)
DatasetA_raw$dep_sum2_scaled<-scale(DatasetA_raw$dep_sum2,center=TRUE,scale=TRUE)

DatasetA_raw$bond_family2<-rep(NA,nrow(DatasetA_raw))
DatasetA_raw$bond_family2[which(DatasetA_raw$Fusion_Family2 !=  5)]<- 0
DatasetA_raw$bond_family2[which(DatasetA_raw$Fusion_Family2 ==  5)]<- 1
DatasetA_raw$bond_friend2<-rep(NA,nrow(DatasetA_raw))
DatasetA_raw$bond_friend2[which(DatasetA_raw$Fusion_Friends2 !=  5)]<- 0
DatasetA_raw$bond_friend2[which(DatasetA_raw$Fusion_Friends2 ==  5)]<- 1
DatasetA_raw$bond_country2<-rep(NA,nrow(DatasetA_raw))
DatasetA_raw$bond_country2[which(DatasetA_raw$Fusion_Country2 !=  5)]<- 0
DatasetA_raw$bond_country2[which(DatasetA_raw$Fusion_Country2 ==  5)]<- 1
DatasetA_raw$bond_humanity2<-rep(NA,nrow(DatasetA_raw))
DatasetA_raw$bond_humanity2[which(DatasetA_raw$Fusion_Humanity2 !=  5)]<- 0
DatasetA_raw$bond_humanity2[which(DatasetA_raw$Fusion_Humanity2 ==  5)]<- 1

DatasetA_raw$bond_mult2<-rep(NA,nrow(DatasetA_raw))
bond_mult2<-data.frame(cbind(DatasetA_raw$bond_family2,DatasetA_raw$bond_friend2,
                            DatasetA_raw$bond_country2,DatasetA_raw$bond_humanity2))
DatasetA_raw$bond_mult2<- ifelse(apply(is.na(bond_mult2),1,all),NA,rowSums(bond_mult2,na.rm=T))
DatasetA_raw$bond_mult2<-factor(DatasetA_raw$bond_mult2)

DatasetA<- DatasetA_raw

# Subset N>100 countries
DatasetA_100<- cbind("Australia", "Brazil", "Croatia", "Finland", "France",
                     "Germany", "Italy", "New Zealand", "Portugal", "UK", "USA")

DatasetA_N100 <- subset(DatasetA_raw, str_detect(DatasetA_raw$Country_Name,"Australia") |
          str_detect(DatasetA_raw$Country_Name,"Brazil") | str_detect(DatasetA_raw$Country_Name,"Croatia
          str_detect(DatasetA_raw$Country_Name,"Finland") | str_detect(DatasetA_raw$Country_Name,"France
          str_detect(DatasetA_raw$Country_Name,"Germany") |  str_detect(DatasetA_raw$Country_Name,"Italy
          str_detect(DatasetA_raw$Country_Name,"New Zealand") | str_detect(DatasetA_raw$Country_Name,"Po
          str_detect(DatasetA_raw$Country_Name,"UK") | str_detect(DatasetA_raw$Country_Name,"USA"))
```

Dataset B

```
# Scale variables and organise Dataset B
DatasetB_raw$GDP<-rep(NA,nrow(DatasetB_raw))
for (i in gdp_data$Country) {
  DatasetB_raw$GDP[which((str_detect(DatasetB_raw$country_now_Name,i)))] <-
    gdp_data$GDPcap[which(gdp_data$Country==i)]
}

DatasetB_raw$comply_self_scale<-scale(DatasetB_raw$comply_self,center=TRUE,scale=TRUE)
DatasetB_raw$wellbeing_scale<-scale(DatasetB_raw$wellbeing,center=TRUE,scale=TRUE)

DatasetB_raw$bond_country<-rep(NA,nrow(DatasetB_raw))
DatasetB_raw$bond_country[which(DatasetB_raw$ios_country !=  4)]<- 0
DatasetB_raw$bond_country[which(DatasetB_raw$ios_country ==  4)]<- 1
```

```
DatasetB_raw$bond_gvmt<-rep(NA,nrow(DatasetB_raw))
DatasetB_raw$bond_gvmt[which(DatasetB_raw$ios_gvmt !=  4)]<- 0
DatasetB_raw$bond_gvmt[which(DatasetB_raw$ios_gvmt ==  4)]<- 1

DatasetB_raw$bond_country<-factor(DatasetB_raw$bond_country)
DatasetB_raw$bond_gvmt<-factor(DatasetB_raw$bond_gvmt)

DatasetB_raw$bond_mult<-rep(NA,nrow(DatasetB_raw))
bond_mult<-data.frame(cbind(DatasetB_raw$bond_country,DatasetB_raw$bond_gvmt)-1)
DatasetB_raw$bond_mult<- ifelse(apply(is.na(bond_mult),1,all),NA,rowSums(bond_mult,na.rm=T))
DatasetB_raw$bond_mult<-factor(DatasetB_raw$bond_mult)

DatasetB_raw$education[which(DatasetB_raw$education==0)]<-"secondary"
DatasetB_raw$education[which(DatasetB_raw$education==1)]<-"secondary"
DatasetB_raw$education[which(DatasetB_raw$education==2)]<-"secondary"
DatasetB_raw$education[which(DatasetB_raw$education==3)]<-"undergraduate"
DatasetB_raw$education[which(DatasetB_raw$education==4)]<-"postgraduate"
DatasetB_raw$education<- factor(DatasetB_raw$education,
                         levels=c("secondary","undergraduate","postgraduate","NA"))

DatasetB_raw$age_ten<-rep(NA,nrow(DatasetB_raw))
DatasetB_raw$age_ten<-cut(DatasetB_raw$age,
                      c(min(DatasetB_raw$age,na.rm=TRUE),24,34,44,54,64,74,
                        max(DatasetB_raw$age,na.rm=TRUE)),include.lowest=TRUE)
DatasetB_raw$gender<- factor(DatasetB_raw$gender, levels=c("man","woman","nb","none"))
DatasetB_raw$status<-rep(NA,nrow(DatasetB_raw))
DatasetB_raw$status<- ifelse((DatasetB_raw$work=="no" & DatasetB_raw$student=="no"),
                             "inactive","active")

DatasetB<- DatasetB_raw

# T6 variables
DatasetB_wide<-reshape(DatasetB, idvar='trial_id', timevar='phase', direction='wide')
colnames(DatasetB_wide)<- gsub(x=colnames(DatasetB_wide),"\\.", "")


# Subset based on phase
DatasetB_phase1 <- DatasetB[DatasetB$phase == "1",]

# Subset N>100 countries
DatasetB_100<- cbind("Australia", "Bangladesh", "Canada", "France", "Germany",
                     "Italy", "Peru", "Sweden", "Turkey", "UK", "USA")
DatasetB_N100 <- subset(DatasetB_phase1, str_detect(DatasetB_phase1$country_now_Name,"Australia") |
        str_detect(DatasetB_phase1$country_now_Name,"Bangladesh") | str_detect(DatasetB_phase1$country_
        str_detect(DatasetB_phase1$country_now_Name,"France") | str_detect(DatasetB_phase1$country_now_N
        str_detect(DatasetB_phase1$country_now_Name,"Italy") | str_detect(DatasetB_phase1$country_now_Na
        str_detect(DatasetB_phase1$country_now_Name,"Sweden") | str_detect(DatasetB_phase1$country_now_N
        str_detect(DatasetB_phase1$country_now_Name,"UK") | str_detect(DatasetB_phase1$country_now_Name
```

Descriptive Statistics

Table S1: Number of participants in demographic and bonding categories

Dataset A

```
table(DatasetA$demo_age_ten)
```

```
##
## [18,24] (24,34] (34,44] (44,54] (54,64] (64,74] (74,81]
##     960    2194    1505     943     679     264      30
```

```
table(DatasetA$demo_gender)
```

```
##
##             Male           Female Other/non-binary
##             2672             3854               61
```

```
table(DatasetA$demo_education)
```

```
##
##     secondary undergraduate  postgraduate            NA
##          1717          2864          1955            53
```

```
table(DatasetA$demo_income)
```

```
##
##  Far below average      Below average            Average      Above average
##                483               1184               1770               2397
##  Far above average I'd rather not say
##                563                182
```

```
table(DatasetA$bond_family)
```

```
##
##    0    1
## 4805 1771
```

```
table(DatasetA$bond_friend)
```

```
##
##    0    1
## 5953  621
```

```
table(DatasetA$bond_country)
```

```
##
##    0    1
## 5876  695
```

```
table(DatasetA$bond_humanity)
```

```
##
##    0    1
## 5334 1239
```

```
table(DatasetA$bond_mult)
```

```
##
##    0    1    2    3    4
## 3665 1899  698  233   83
```

Dataset B

```
table(DatasetB_phase1$age_ten)
```

```
##
## [16,24] (24,34] (34,44] (44,54] (54,64] (64,74] (74,90]
##    1505    2093    1310     812     633     264      58
```

```
table(DatasetB_phase1$gender)
```

```
##
##   man woman    nb  none
##  2204  4356    59    56
```

```
table(DatasetB_phase1$household_number)
```

```
##
##    0    1    2    3    4    5    6    7    8    9   10
##  818 1660 1430 1377  728  334  132   72   39   30   55
```

```
table(DatasetB_phase1$education)
```

```
##
##     secondary undergraduate  postgraduate            NA
##          1102          3096          2477             0
```

```
table(DatasetB_phase1$status)
```

```
##
##   active inactive
##     5532     1143
```

```
table(DatasetB_phase1$bond_country)
```

```
##
##    0    1
## 5035 1427
```

```
table(DatasetB_phase1$bond_gvmt)
```

```
##
##    0    1
## 5906  433
```

```
table(DatasetB_phase1$bond_mult)
```

```
##
##    0    1    2
## 5001 1158  351
```

Table S2: Number of participants per country

The output of the below code has been compiled into a spreadsheet entitled 'Country_ppt_numbers.csv', which can be found on the project's OSF website: https://doi.org/10.17605/OSF.IO/BGZUF.

Dataset A

```
countries_a<- data.frame(unclass(table(DatasetA$Country_Name)))
colnames(countries_a)<- c("country")
```

Dataset B

```
countries_b <- data.frame(unclass((table(DatasetB_phase1$country_now_Name))))
colnames(countries_b)<- c("country")
countries<-rbind(countries_a,countries_b)
# write.csv(countries,"Country_ppt_numbers.csv")
```

Table S3: Zero-order correlations among key variables

Dataset A

```
# Dichotomous bonding variables
cors_DatA <- as.matrix(cbind(DatasetA$bond_family, DatasetA$bond_friend,
                             DatasetA$bond_country, DatasetA$bond_humanity,
                             DatasetA$Pro_Distancing, DatasetA$Pro_Hygiene, DatasetA$Pro_MaskWearing,
                             DatasetA$anx_sum1, DatasetA$dep_sum1))
rcorr(cors_DatA, type=c("spearman"))
```

```
##        [,1]  [,2]  [,3]  [,4] [,5] [,6] [,7]  [,8]  [,9]
##  [1,]  1.00  0.27  0.16  0.08 0.04 0.15 0.10 -0.08 -0.09
##  [2,]  0.27  1.00  0.16  0.12 0.01 0.07 0.02 -0.05 -0.11
##  [3,]  0.16  0.16  1.00  0.31 0.01 0.01 0.00 -0.12 -0.11
##  [4,]  0.08  0.12  0.31  1.00 0.04 0.03 0.06 -0.04 -0.07
##  [5,]  0.04  0.01  0.01  0.04 1.00 0.28 0.13  0.10  0.02
##  [6,]  0.15  0.07  0.01  0.03 0.28 1.00 0.27  0.20  0.04
##  [7,]  0.10  0.02  0.00  0.06 0.13 0.27 1.00  0.13  0.11
##  [8,] -0.08 -0.05 -0.12 -0.04 0.10 0.20 0.13  1.00  0.62
##  [9,] -0.09 -0.11 -0.11 -0.07 0.02 0.04 0.11  0.62  1.00
##
## n
##       [,1] [,2] [,3] [,4] [,5] [,6] [,7] [,8] [,9]
##  [1,] 6576 6572 6569 6571 6560 6545 6551 6572 6573
##  [2,] 6572 6574 6569 6571 6558 6543 6549 6570 6572
##  [3,] 6569 6569 6571 6570 6555 6540 6546 6567 6568
##  [4,] 6571 6571 6570 6573 6557 6542 6548 6569 6570
##  [5,] 6560 6558 6555 6557 6572 6547 6553 6569 6569
##  [6,] 6545 6543 6540 6542 6547 6556 6538 6553 6553
##  [7,] 6551 6549 6546 6548 6553 6538 6563 6560 6560
##  [8,] 6572 6570 6567 6569 6569 6553 6560 6584 6582
##  [9,] 6573 6572 6568 6570 6569 6553 6560 6582 6585
##
## P
##       [,1]   [,2]   [,3]   [,4]   [,5]   [,6]   [,7]   [,8]   [,9]
##  [1,]        0.0000 0.0000 0.0000 0.0040 0.0000 0.0000 0.0000 0.0000
##  [2,] 0.0000        0.0000 0.0000 0.3567 0.0000 0.1144 0.0000 0.0000
##  [3,] 0.0000 0.0000        0.0000 0.4303 0.3039 0.8886 0.0000 0.0000
##  [4,] 0.0000 0.0000 0.0000        0.0019 0.0152 0.0000 0.0004 0.0000
##  [5,] 0.0040 0.3567 0.4303 0.0019        0.0000 0.0000 0.0000 0.1655
##  [6,] 0.0000 0.0000 0.3039 0.0152 0.0000        0.0000 0.0000 0.0014
##  [7,] 0.0000 0.1144 0.8886 0.0000 0.0000 0.0000        0.0000 0.0000
##  [8,] 0.0000 0.0000 0.0000 0.0004 0.0000 0.0000 0.0000        0.0000
##  [9,] 0.0000 0.0000 0.0000 0.0000 0.1655 0.0014 0.0000 0.0000
```

```r
# Continuous bonding variables  (1-5)
cors_cont_DatA <- as.matrix(cbind(DatasetA$Fusion_Family1, DatasetA$Fusion_Friends1,
                                  DatasetA$Fusion_Country1, DatasetA$Fusion_Humanity1,
                                  DatasetA$Pro_Distancing, DatasetA$Pro_Hygiene, DatasetA$Pro_MaskWeari
                                  DatasetA$anx_sum1, DatasetA$dep_sum1))
rcorr(cors_cont_DatA, type=c("pearson"))
```

```
##        [,1]  [,2]  [,3]  [,4]  [,5] [,6]  [,7]  [,8]  [,9]
##  [1,]  1.00  0.32  0.30  0.18  0.02 0.15  0.07 -0.11 -0.16
##  [2,]  0.32  1.00  0.30  0.23  0.00 0.05  0.00 -0.13 -0.19
##  [3,]  0.30  0.30  1.00  0.40 -0.01 0.00 -0.03 -0.23 -0.21
##  [4,]  0.18  0.23  0.40  1.00  0.05 0.06  0.05 -0.07 -0.13
##  [5,]  0.02  0.00 -0.01  0.05  1.00 0.32  0.12  0.10  0.02
##  [6,]  0.15  0.05  0.00  0.06  0.32 1.00  0.28  0.21  0.04
##  [7,]  0.07  0.00 -0.03  0.05  0.12 0.28  1.00  0.12  0.10
##  [8,] -0.11 -0.13 -0.23 -0.07  0.10 0.21  0.12  1.00  0.62
##  [9,] -0.16 -0.19 -0.21 -0.13  0.02 0.04  0.10  0.62  1.00
##
## n
```

```
##       [,1] [,2] [,3] [,4] [,5] [,6] [,7] [,8] [,9]
##  [1,] 6576 6572 6569 6571 6560 6545 6551 6572 6573
##  [2,] 6572 6574 6569 6571 6558 6543 6549 6570 6572
##  [3,] 6569 6569 6571 6570 6555 6540 6546 6567 6568
##  [4,] 6571 6571 6570 6573 6557 6542 6548 6569 6570
##  [5,] 6560 6558 6555 6557 6572 6547 6553 6569 6569
##  [6,] 6545 6543 6540 6542 6547 6556 6538 6553 6553
##  [7,] 6551 6549 6546 6548 6553 6538 6563 6560 6560
##  [8,] 6572 6570 6567 6569 6569 6553 6560 6584 6582
##  [9,] 6573 6572 6568 6570 6569 6553 6560 6582 6585
##
## P
##       [,1]   [,2]   [,3]   [,4]   [,5]   [,6]   [,7]   [,8]   [,9]
##  [1,]        0.0000 0.0000 0.0000 0.0439 0.0000 0.0000 0.0000 0.0000
##  [2,] 0.0000        0.0000 0.0000 0.9239 0.0000 0.9884 0.0000 0.0000
##  [3,] 0.0000 0.0000        0.0000 0.4413 0.9968 0.0071 0.0000 0.0000
##  [4,] 0.0000 0.0000 0.0000        0.0000 0.0000 0.0000 0.0000 0.0000
##  [5,] 0.0439 0.9239 0.4413 0.0000        0.0000 0.0000 0.0000 0.0944
##  [6,] 0.0000 0.0000 0.9968 0.0000 0.0000        0.0000 0.0000 0.0014
##  [7,] 0.0000 0.9884 0.0071 0.0000 0.0000 0.0000        0.0000 0.0000
##  [8,] 0.0000 0.0000 0.0000 0.0000 0.0000 0.0000 0.0000        0.0000
##  [9,] 0.0000 0.0000 0.0000 0.0000 0.0944 0.0014 0.0000 0.0000
```

Dataset B

```
# Dichotomous bonding variables
cors_DatB <- as.matrix(cbind(DatasetB_phase1$bond_country, DatasetB_phase1$bond_gvmt,
                             DatasetB_phase1$comply_self, DatasetB_phase1$wellbeing))
rcorr(cors_DatB, type=c("spearman"))
```

```
##       [,1] [,2]  [,3] [,4]
## [1,]  1.00 0.39 -0.02 0.20
## [2,]  0.39 1.00  0.00 0.13
## [3,] -0.02 0.00  1.00 0.01
## [4,]  0.20 0.13  0.01 1.00
##
## n
##      [,1] [,2] [,3] [,4]
## [1,] 6462 6291 6462 6462
## [2,] 6291 6339 6339 6339
## [3,] 6462 6339 6675 6675
## [4,] 6462 6339 6675 6675
##
## P
##      [,1]   [,2]   [,3]   [,4]
## [1,]        0.0000 0.0956 0.0000
## [2,] 0.0000        0.8871 0.0000
## [3,] 0.0956 0.8871        0.3179
## [4,] 0.0000 0.0000 0.3179
```

```r
# Continuous bonding variables  (1-5)
cors_cont_DatB <- as.matrix(cbind(DatasetB_phase1$ios_country, DatasetB_phase1$ios_gvmt,
                                  DatasetB_phase1$comply_self, DatasetB_phase1$wellbeing))
rcorr(cors_cont_DatB, type=c("pearson"))
```

```
##       [,1]  [,2]  [,3] [,4]
## [1,]  1.00  0.53 -0.05 0.24
## [2,]  0.53  1.00 -0.03 0.18
## [3,] -0.05 -0.03  1.00 0.02
## [4,]  0.24  0.18  0.02 1.00
##
## n
##      [,1] [,2] [,3] [,4]
## [1,] 6462 6291 6462 6462
## [2,] 6291 6339 6339 6339
## [3,] 6462 6339 6675 6675
## [4,] 6462 6339 6675 6675
##
## P
##      [,1]   [,2]   [,3]   [,4]
## [1,]        0.0000 0.0000 0.0000
## [2,] 0.0000        0.0053 0.0000
## [3,] 0.0000 0.0053        0.1065
## [4,] 0.0000 0.0000 0.1065
```

Table S4: Descriptive statistics of key variables in N > 100 countries and in the entire sample

Dataset A, N>100 countries

```r
# Bonding variables in N>100 countries
table(DatasetA_N100$bond_family, DatasetA_N100$Country_Name)
```

```
##
##     Australia Brazil Croatia Finland France Germany Italy New Zealand Portugal
##   0       366    599     144     137    139     184   488         106      193
##   1       155    202      38      28     39      50   412          39      139
##
##       UK  USA
##   0  651 1263
##   1  170  361
```

```r
table(DatasetA_N100$bond_friend, DatasetA_N100$Country_Name)
```

```
##
##     Australia Brazil Croatia Finland France Germany Italy New Zealand Portugal
##   0       474    718     167     156    162     212   782         136      262
##   1        47     83      15       9     15      21   119           9       70
##
##       UK  USA
```

```
##   0  764 1507
##   1   56  117
```

```r
table(DatasetA_N100$bond_country, DatasetA_N100$Country_Name)
```

```
##
##     Australia Brazil Croatia Finland France Germany Italy New Zealand Portugal
##   0       434    701     169     139    151     215   767         105      236
##   1        86    100      13      27     27      17   134          40       94
##
##        UK  USA
##   0   790 1571
##   1    30   52
```

```r
table(DatasetA_N100$bond_humanity, DatasetA_N100$Country_Name)
```

```
##
##     Australia Brazil Croatia Finland France Germany Italy New Zealand Portugal
##   0       459    601     154     147    140     206   670         122      244
##   1        61    200      28      19     38      27   230          23       88
##
##        UK  USA
##   0   694 1334
##   1   126  289
```

```r
table(DatasetA_N100$bond_mult, DatasetA_N100$Country_Name)
```

```
##
##     Australia Brazil Croatia Finland France Germany Italy New Zealand Portugal
##   0       301    402     113     108    100     151   336          66      124
##   1       129    264      49      41     45      58   330          54      106
##   2        60     94      15       9     25      19   160          19       47
##   3        24     31       5       8      8       5    55           5       29
##   4         7     10       0       0      0       1    20           1       26
##
##        UK  USA
##   0   535 1008
##   1   209  452
##   2    61  128
##   3    13   33
##   4     3    3
```

```r
# Health behaviours in N>100 countries
aggregate(DatasetA_N100$Pro_Distancing, list(DatasetA_N100$Country_Name), mean, na.rm=T)
```

```
##        Group.1        x
## 1    Australia 3.654511
## 2       Brazil 3.705882
## 3      Croatia 3.620879
## 4      Finland 3.443775
## 5       France 3.825843
```

```
## 6      Germany 3.529915
## 7        Italy 3.551955
## 8  New Zealand 3.793103
## 9     Portugal 3.673695
## 10          UK 3.701626
## 11         USA 3.783385
```

```
aggregate(DatasetA_N100$Pro_Hygiene, list(DatasetA_N100$Country_Name), mean, na.rm=T)
```

```
##        Group.1        x
## 1    Australia 2.540883
## 2       Brazil 2.685464
## 3      Croatia 2.617582
## 4      Finland 2.126506
## 5       France 2.226136
## 6      Germany 2.262393
## 7        Italy 2.310181
## 8  New Zealand 2.440000
## 9     Portugal 2.684337
## 10          UK 2.502071
## 11         USA 2.798397
```

```
aggregate(DatasetA_N100$Pro_MaskWearing, list(DatasetA_N100$Country_Name), mean, na.rm=T)
```

```
##        Group.1         x
## 1    Australia 0.4692308
## 2       Brazil 1.1506250
## 3      Croatia 1.0412088
## 4      Finland 0.5210843
## 5       France 0.6723164
## 6      Germany 0.7500000
## 7        Italy 1.7905935
## 8  New Zealand 0.7689655
## 9     Portugal 0.9559271
## 10          UK 0.7115854
## 11         USA 1.2760320
```

```
# Mental health variables in N>100 countries
aggregate(DatasetA_N100$anx_sum1, list(DatasetA_N100$Country_Name), mean, na.rm=T)
```

```
##        Group.1        x
## 1    Australia 6.533589
## 2       Brazil 8.878901
## 3      Croatia 7.478022
## 4      Finland 5.951807
## 5       France 6.325843
## 6      Germany 7.025641
## 7        Italy 7.025499
## 8  New Zealand 6.268966
## 9     Portugal 6.900901
## 10          UK 8.231144
## 11         USA 9.617231
```

```r
aggregate(DatasetA_N100$dep_sum1, list(DatasetA_N100$Country_Name), mean, na.rm=T)
```

```
##        Group.1        x
## 1    Australia 6.273946
## 2       Brazil 7.178527
## 3      Croatia 6.500000
## 4      Finland 5.897590
## 5       France 5.276836
## 6      Germany 6.333333
## 7        Italy 8.197121
## 8  New Zealand 6.144828
## 9     Portugal 5.423423
## 10          UK 7.475091
## 11         USA 7.413538
```

```r
### Dataset A, N>100 countries
```

Dataset A, entire sample

```r
# Bonding variables in entire sample
table(DatasetA$bond_family, DatasetA$Country_Name)
```

```
##    
##          Afghanistan Albania Andorra Angola Argentina Australia Austria
##   0    5           1       1       0      1         0       366       8
##   1    0           0       1       1      1         4       155       0
##    
##     Azerbeijan Bangladesh Belgium Bosnia Brazil Bulgaria Canada Chile
##   0          1          1       4      4    599        0     30     8
##   1          0          0       5      1    202        1      8    12
##    
##     Cote dIvoire Croatia Cyprus Czech Republic Denmark Egypt Finland France
##   0            1     144      1              4       6     1     137    139
##   1            0      38      0              1       0     0      28     39
##    
##     Georgia Germany Greece Hong Kong Hungary India Indonesia Ireland Israel
##   0       1     184      1         1       0     3         5      10      2
##   1       0      50      1         1       1     3         4       2      0
##    
##     Italy Japan Jordan Luxembourg Malaysia Mexico Monaco Montenegro Morocco
##   0   488     2      1          1        2      4      2          1       1
##   1   412     0      0          1        0      0      0          0       0
##    
##     Mozambique Netherlands New Zealand Nicaragua Norway Oman Pakistan Peru
##   0          1         320         106         1      5    1        0    1
##   1          0          59          39         0      0    0        1    1
##    
##     Philippines Poland Portugal Qatar Romania Saudi Arabia Serbia Singapore
##   0           1      9      193     7       3            1      2         2
##   1           3      1      139     0       2            1      0         2
```

```
##
##     Slovakia Slovenia South Africa Spain Sri Lanka Sweden Switzerland Thailand
##   0        1        1            3    28         1      7          16        1
##   1        0        0            3     7         0      4           5        0
##
##     Turkey Uganda   UK United Arab Emirates  USA Vietnam Yugoslavia Zimbabwe
##   0      3      1  651                    1 1263       1          2        1
##   1      1      0  170                    0  361       0          0        0
```

```
table(DatasetA$bond_friend, DatasetA$Country_Name)
```

```
##
##          Afghanistan Albania Andorra Angola Argentina Australia Austria
##   0    5           1       2       0      1         3       474       8
##   1    0           0       0       1      1         1        47       0
##
##     Azerbeijan Bangladesh Belgium Bosnia Brazil Bulgaria Canada Chile
##   0          1          1       7      5    718        1     34    17
##   1          0          0       2      0     83        0      4     3
##
##     Cote dIvoire Croatia Cyprus Czech Republic Denmark Egypt Finland France
##   0            1     167      1              4       6     1     156    162
##   1            0      15      0              1       0     0       9     15
##
##     Georgia Germany Greece Hong Kong Hungary India Indonesia Ireland Israel
##   0       1     212      2         2       1     5         9       9      0
##   1       0      21      0         0       0     1         0       3      2
##
##     Italy Japan Jordan Luxembourg Malaysia Mexico Monaco Montenegro Morocco
##   0   782     2      1          2        2      4      2          1       1
##   1   119     0      0          0        0      0      0          0       0
##
##     Mozambique Netherlands New Zealand Nicaragua Norway Oman Pakistan Peru
##   0          1         350         136         1      4    1        1    2
##   1          0          29           9         0      1    0        0    0
##
##     Philippines Poland Portugal Qatar Romania Saudi Arabia Serbia Singapore
##   0           4     10      262     7       4            2      2         3
##   1           0      0       70     0       1            0      0         1
##
##     Slovakia Slovenia South Africa Spain Sri Lanka Sweden Switzerland Thailand
##   0        1        1            5    31         1      9          19        1
##   1        0        0            1     4         0      2           2        0
##
##     Turkey Uganda   UK United Arab Emirates  USA Vietnam Yugoslavia Zimbabwe
##   0      4      1  764                    1 1507       1          2        1
##   1      0      0   56                    0  117       0          0        0
```

```
table(DatasetA$bond_country, DatasetA$Country_Name)
```

```
##
##          Afghanistan Albania Andorra Angola Argentina Australia Austria
##   0    4           1       2       0      1         1       434       6
```

```
##   1    1           0       0       1      1         3        86       2
##
##     Azerbeijan Bangladesh Belgium Bosnia Brazil Bulgaria Canada Chile
##   0          1          1       8      5    701        1     35    17
##   1          0          0       1      0    100        0      3     3
##
##     Cote dIvoire Croatia Cyprus Czech Republic Denmark Egypt Finland France
##   0            1     169      1              3       6     1     139    151
##   1            0      13      0              2       0     0      27     27
##
##     Georgia Germany Greece Hong Kong Hungary India Indonesia Ireland Israel
##   0       0     215      2         2       1     4         7       9      1
##   1       1      17      0         0       0     2         2       3      1
##
##     Italy Japan Jordan Luxembourg Malaysia Mexico Monaco Montenegro Morocco
##   0   767     2      1          2        2      4      2          1       1
##   1   134     0      0          0        0      0      0          0       0
##
##     Mozambique Netherlands New Zealand Nicaragua Norway Oman Pakistan Peru
##   0          1         341         105         1      5    0        0    2
##   1          0          38          40         0      0    1        1    0
##
##     Philippines Poland Portugal Qatar Romania Saudi Arabia Serbia Singapore
##   0           4     10      236     6       5            2      1         4
##   1           0      0       94     1       0            0      1         0
##
##     Slovakia Slovenia South Africa Spain Sri Lanka Sweden Switzerland Thailand
##   0        1        1            6    33         1      8          19        1
##   1        0        0            0     2         0      3           2        0
##
##     Turkey Uganda   UK United Arab Emirates  USA Vietnam Yugoslavia Zimbabwe
##   0      4      1  790                    1 1571       1          2        1
##   1      0      0   30                    0   52       0          0        0
```

```
table(DatasetA$bond_humanity, DatasetA$Country_Name)
```

```
##
##          Afghanistan Albania Andorra Angola Argentina Australia Austria
##   0    3           1       2       0      0         1       459       7
##   1    2           0       0       1      2         3        61       1
##
##     Azerbeijan Bangladesh Belgium Bosnia Brazil Bulgaria Canada Chile
##   0          1          1       7      5    601        1     31    15
##   1          0          0       2      0    200        0      7     5
##
##     Cote dIvoire Croatia Cyprus Czech Republic Denmark Egypt Finland France
##   0            1     154      1              5       5     1     147    140
##   1            0      28      0              0       1     0      19     38
##
##     Georgia Germany Greece Hong Kong Hungary India Indonesia Ireland Israel
##   0       0     206      2         2       1     4         5      10      1
##   1       1      27      0         0       0     2         4       2      1
##
##     Italy Japan Jordan Luxembourg Malaysia Mexico Monaco Montenegro Morocco
```

```
##   0   670     2      0          2        2      4      2          1        1
##   1   230     0      1          0        0      0      0          0        0
##
##     Mozambique Netherlands New Zealand Nicaragua Norway Oman Pakistan Peru
##   0          1         334         122         1      4    1        0    2
##   1          0          45          23         0      1    0        1    0
##
##     Philippines Poland Portugal Qatar Romania Saudi Arabia Serbia Singapore
##   0           3      8      244     6       3            2      2         3
##   1           1      2       88     1       2            0      0         1
##
##     Slovakia Slovenia South Africa Spain Sri Lanka Sweden Switzerland Thailand
##   0        1        0            5    25         1      7          18        1
##   1        0        1            1    10         0      4           3        0
##
##     Turkey Uganda   UK United Arab Emirates  USA Vietnam Yugoslavia Zimbabwe
##   0      3      1  694                    1 1334       1          2        0
##   1      1      0  126                    0  289       0          0        1
```

```
table(DatasetA$bond_mult, DatasetA$Country_Name)
```

```
##
##          Afghanistan Albania Andorra Angola Argentina Australia Austria
##   0    2           1       1       0      0         0       301       6
##   1    3           0       1       0      0         1       129       1
##   2    0           0       0       0      1         0        60       1
##   3    0           0       0       0      1         2        24       0
##   4    0           0       0       1      0         1         7       0
##
##     Azerbeijan Bangladesh Belgium Bosnia Brazil Bulgaria Canada Chile
##   0          1          1       3      4    402        0     21     7
##   1          0          0       4      1    264        1     13     8
##   2          0          0       1      0     94        0      3     2
##   3          0          0       0      0     31        0      1     1
##   4          0          0       1      0     10        0      0     2
##
##     Cote dIvoire Croatia Cyprus Czech Republic Denmark Egypt Finland France
##   0            1     113      1              3       5     1     108    100
##   1            0      49      0              0       1     0      41     45
##   2            0      15      0              2       0     0       9     25
##   3            0       5      0              0       0     0       8      8
##   4            0       0      0              0       0     0       0      0
##
##     Georgia Germany Greece Hong Kong Hungary India Indonesia Ireland Israel
##   0       0     151      1         1       0     2         2       6      0
##   1       0      58      1         1       1     2         5       3      1
##   2       1      19      0         0       0     1         1       2      0
##   3       0       5      0         0       0     0         1       1      1
##   4       0       1      0         0       0     1         0       0      0
##
##     Italy Japan Jordan Luxembourg Malaysia Mexico Monaco Montenegro Morocco
##   0   336     2      0          1        2      4      2          1       1
##   1   330     0      1          1        0      0      0          0       0
##   2   160     0      0          0        0      0      0          0       0
```

```
##   3     55      0       0           0         0       0       0           0       0
##   4     20      0       0           0         0       0       0           0       0
##
##     Mozambique Netherlands New Zealand Nicaragua Norway Oman Pakistan Peru
##   0          1         263          66         1      4    0        0    1
##   1          0          77          54         0      0    1        0    1
##   2          0          28          19         0      1    0        0    0
##   3          0           6           5         0      0    0        1    0
##   4          0           5           1         0      0    0        0    0
##
##     Philippines Poland Portugal Qatar Romania Saudi Arabia Serbia Singapore
##   0           0      7      124     6       2            1      1         1
##   1           4      3      106     0       1            1      1         2
##   2           0      0       47     1       2            0      0         1
##   3           0      0       29     0       0            0      0         0
##   4           0      0       26     0       0            0      0         0
##
##     Slovakia Slovenia South Africa Spain Sri Lanka Sweden Switzerland Thailand
##   0        1        0            2    20         1      4          13        1
##   1        0        1            3    10         0      2           4        0
##   2        0        0            1     3         0      4           4        0
##   3        0        0            0     1         0      1           0        0
##   4        0        0            0     1         0      0           0        0
##
##     Turkey Uganda   UK United Arab Emirates  USA Vietnam Yugoslavia Zimbabwe
##   0      3      1  535                    1 1008       1          2        0
##   1      0      0  209                    0  452       0          0        1
##   2      1      0   61                    0  128       0          0        0
##   3      0      0   13                    0   33       0          0        0
##   4      0      0    3                    0    3       0          0        0
```

```
# Health behaviours in entire sample
aggregate(DatasetA$Pro_Distancing, list(DatasetA$Country_Name), mean, na.rm=T)
```

```
##                Group.1        x
## 1                      3.933333
## 2          Afghanistan 0.000000
## 3              Albania 4.000000
## 4              Andorra 1.333333
## 5               Angola 3.000000
## 6            Argentina 3.583333
## 7            Australia 3.654511
## 8              Austria 3.583333
## 9           Azerbeijan      NaN
## 10          Bangladesh 4.000000
## 11             Belgium 3.925926
## 12              Bosnia 3.200000
## 13              Brazil 3.705882
## 14            Bulgaria 4.000000
## 15              Canada 3.833333
## 16               Chile 3.523810
## 17         Cote dIvoire 2.333333
## 18             Croatia 3.620879
## 19              Cyprus 4.000000
```

```
## 20       Czech Republic 3.533333
## 21              Denmark 3.611111
## 22                Egypt 3.000000
## 23              Finland 3.443775
## 24               France 3.825843
## 25              Georgia 4.000000
## 26              Germany 3.529915
## 27               Greece 3.500000
## 28            Hong Kong 2.833333
## 29              Hungary 3.000000
## 30                India 3.222222
## 31            Indonesia 3.259259
## 32              Ireland 3.916667
## 33               Israel 2.500000
## 34                Italy 3.551955
## 35                Japan 2.166667
## 36               Jordan 4.000000
## 37           Luxembourg 4.000000
## 38             Malaysia 4.000000
## 39               Mexico 4.000000
## 40               Monaco 2.000000
## 41           Montenegro 3.666667
## 42              Morocco 4.000000
## 43           Mozambique 3.000000
## 44          Netherlands 3.674541
## 45          New Zealand 3.793103
## 46            Nicaragua 3.666667
## 47               Norway 3.866667
## 48                 Oman 4.000000
## 49             Pakistan 0.000000
## 50                 Peru 3.833333
## 51          Philippines 3.750000
## 52               Poland 3.733333
## 53             Portugal 3.673695
## 54                Qatar 3.285714
## 55              Romania 3.733333
## 56         Saudi Arabia 3.666667
## 57               Serbia 3.166667
## 58            Singapore 3.500000
## 59             Slovakia 2.000000
## 60             Slovenia 4.000000
## 61         South Africa 3.611111
## 62                Spain 3.790476
## 63            Sri Lanka 3.333333
## 64               Sweden 3.515152
## 65          Switzerland 3.507937
## 66             Thailand 4.000000
## 67               Turkey 4.000000
## 68               Uganda 4.000000
## 69                   UK 3.701626
## 70 United Arab Emirates 4.000000
## 71                  USA 3.783385
## 72              Vietnam 3.333333
## 73           Yugoslavia 4.000000
```

```
## 74              Zimbabwe 4.000000
```

```r
aggregate(DatasetA$Pro_Hygiene, list(DatasetA$Country_Name), mean, na.rm=T)
```

```
##                Group.1        x
## 1                       2.480000
## 2          Afghanistan 0.000000
## 3              Albania 3.500000
## 4              Andorra 2.600000
## 5               Angola 2.600000
## 6            Argentina 3.050000
## 7            Australia 2.540883
## 8              Austria 2.150000
## 9           Azerbeijan 3.000000
## 10          Bangladesh 2.800000
## 11             Belgium 2.711111
## 12              Bosnia 2.760000
## 13              Brazil 2.685464
## 14            Bulgaria 4.000000
## 15              Canada 2.405263
## 16               Chile 2.800000
## 17        Cote dIvoire 2.400000
## 18             Croatia 2.617582
## 19              Cyprus 2.600000
## 20      Czech Republic 2.600000
## 21             Denmark 2.433333
## 22               Egypt 3.400000
## 23             Finland 2.126506
## 24              France 2.226136
## 25             Georgia 2.200000
## 26             Germany 2.262393
## 27              Greece 2.300000
## 28           Hong Kong 2.300000
## 29             Hungary 2.800000
## 30               India 2.666667
## 31           Indonesia 2.466667
## 32             Ireland 2.883333
## 33              Israel 1.800000
## 34               Italy 2.310181
## 35               Japan 1.800000
## 36              Jordan 2.800000
## 37          Luxembourg 2.500000
## 38            Malaysia 2.500000
## 39              Mexico 3.050000
## 40              Monaco 0.900000
## 41          Montenegro 2.400000
## 42             Morocco 2.200000
## 43          Mozambique 2.400000
## 44         Netherlands 2.305263
## 45         New Zealand 2.440000
## 46           Nicaragua 3.200000
## 47              Norway 2.360000
## 48                Oman 3.000000
## 49            Pakistan 0.600000
```

```
## 50                 Peru 3.200000
## 51          Philippines 2.550000
## 52               Poland 2.100000
## 53             Portugal 2.684337
## 54                Qatar 2.542857
## 55              Romania 2.640000
## 56         Saudi Arabia 3.000000
## 57               Serbia 2.600000
## 58            Singapore 1.950000
## 59             Slovakia 0.800000
## 60             Slovenia 2.400000
## 61         South Africa 2.800000
## 62                Spain 2.285714
## 63            Sri Lanka 2.600000
## 64               Sweden 2.218182
## 65          Switzerland 2.352381
## 66             Thailand 4.000000
## 67               Turkey 2.700000
## 68               Uganda 3.600000
## 69                   UK 2.502071
## 70 United Arab Emirates 2.800000
## 71                  USA 2.798397
## 72              Vietnam 1.000000
## 73           Yugoslavia 3.600000
## 74             Zimbabwe 3.200000
```

```
aggregate(DatasetA$Pro_MaskWearing, list(DatasetA$Country_Name), mean, na.rm=T)
```

```
##              Group.1         x
## 1                    0.9000000
## 2        Afghanistan 0.0000000
## 3            Albania 3.0000000
## 4            Andorra 0.0000000
## 5             Angola 1.0000000
## 6          Argentina 0.2500000
## 7          Australia 0.4692308
## 8            Austria 1.0625000
## 9         Azerbeijan 0.5000000
## 10        Bangladesh 4.0000000
## 11           Belgium 0.3888889
## 12            Bosnia 1.1000000
## 13            Brazil 1.1506250
## 14          Bulgaria 4.0000000
## 15            Canada 0.6447368
## 16             Chile 1.4523810
## 17       Cote dIvoire 0.0000000
## 18           Croatia 1.0412088
## 19            Cyprus 0.5000000
## 20    Czech Republic 2.3000000
## 21           Denmark 0.0000000
## 22             Egypt 0.0000000
## 23           Finland 0.5210843
## 24            France 0.6723164
## 25           Georgia 0.0000000
```

```
## 26              Germany 0.7500000
## 27               Greece 1.0000000
## 28            Hong Kong 2.0000000
## 29              Hungary 0.5000000
## 30                India 1.6666667
## 31            Indonesia 1.6666667
## 32              Ireland 0.1666667
## 33               Israel 1.7500000
## 34                Italy 1.7905935
## 35                Japan 2.7500000
## 36               Jordan 0.0000000
## 37           Luxembourg 3.5000000
## 38             Malaysia 1.0000000
## 39               Mexico 3.2500000
## 40               Monaco 1.0000000
## 41           Montenegro 0.0000000
## 42              Morocco 0.0000000
## 43           Mozambique 0.0000000
## 44          Netherlands 0.2960526
## 45          New Zealand 0.7689655
## 46            Nicaragua 0.0000000
## 47               Norway 0.0000000
## 48                 Oman 4.0000000
## 49             Pakistan 0.0000000
## 50                 Peru 2.7500000
## 51          Philippines 1.8750000
## 52               Poland 1.6000000
## 53             Portugal 0.9559271
## 54                Qatar 1.3571429
## 55              Romania 2.2000000
## 56         Saudi Arabia 1.5000000
## 57               Serbia 1.0000000
## 58            Singapore 1.7500000
## 59             Slovakia 2.0000000
## 60             Slovenia 0.5000000
## 61         South Africa 0.8333333
## 62                Spain 1.1142857
## 63            Sri Lanka       NaN
## 64               Sweden 0.3636364
## 65          Switzerland 0.5952381
## 66             Thailand 4.0000000
## 67               Turkey 1.5000000
## 68               Uganda 2.0000000
## 69                   UK 0.7115854
## 70 United Arab Emirates 4.0000000
## 71                  USA 1.2760320
## 72              Vietnam 2.0000000
## 73           Yugoslavia 3.0000000
## 74             Zimbabwe 1.5000000
```

```r
# Mental health variables in entire sample
aggregate(DatasetA$anx_sum1, list(DatasetA$Country_Name), mean, na.rm=T)
```

```
##                 Group.1         x
```

```
## 1                     6.800000
## 2        Afghanistan  0.000000
## 3            Albania 11.000000
## 4            Andorra  1.000000
## 5             Angola  7.500000
## 6          Argentina  6.750000
## 7          Australia  6.533589
## 8            Austria  6.875000
## 9         Azerbeijan  5.000000
## 10        Bangladesh  7.000000
## 11           Belgium  3.888889
## 12            Bosnia  4.000000
## 13            Brazil  8.878901
## 14          Bulgaria  9.000000
## 15            Canada  9.342105
## 16             Chile  8.380952
## 17      Cote dIvoire  6.000000
## 18           Croatia  7.478022
## 19            Cyprus  7.000000
## 20    Czech Republic  5.400000
## 21           Denmark  5.166667
## 22             Egypt  6.000000
## 23           Finland  5.951807
## 24            France  6.325843
## 25           Georgia  3.000000
## 26           Germany  7.025641
## 27            Greece  7.000000
## 28         Hong Kong  7.000000
## 29           Hungary  7.000000
## 30             India  9.166667
## 31         Indonesia  8.555556
## 32           Ireland  8.833333
## 33            Israel  8.500000
## 34             Italy  7.025499
## 35             Japan  1.000000
## 36            Jordan  2.000000
## 37        Luxembourg  2.500000
## 38          Malaysia  6.000000
## 39            Mexico 10.750000
## 40            Monaco  9.500000
## 41        Montenegro  8.000000
## 42           Morocco  6.000000
## 43        Mozambique  7.000000
## 44       Netherlands  6.952632
## 45       New Zealand  6.268966
## 46         Nicaragua 13.000000
## 47            Norway  8.600000
## 48              Oman 10.000000
## 49          Pakistan  0.000000
## 50              Peru  4.500000
## 51       Philippines  5.250000
## 52            Poland  5.500000
## 53          Portugal  6.900901
## 54             Qatar  5.142857
```

```
## 55              Romania  8.600000
## 56         Saudi Arabia  3.500000
## 57               Serbia  5.500000
## 58            Singapore 10.750000
## 59             Slovakia  1.000000
## 60             Slovenia  7.000000
## 61         South Africa  8.166667
## 62                Spain  6.485714
## 63            Sri Lanka  8.000000
## 64               Sweden  6.363636
## 65          Switzerland  5.047619
## 66             Thailand 13.000000
## 67               Turkey  6.500000
## 68               Uganda  8.000000
## 69                   UK  8.231144
## 70 United Arab Emirates 13.000000
## 71                  USA  9.617231
## 72              Vietnam  2.000000
## 73           Yugoslavia  3.500000
## 74             Zimbabwe 13.000000
```

```
aggregate(DatasetA$dep_sum1, list(DatasetA$Country_Name), mean, na.rm=T)
```

```
##                Group.1         x
## 1                        4.800000
## 2          Afghanistan  0.000000
## 3              Albania  9.000000
## 4              Andorra  2.000000
## 5               Angola  6.500000
## 6            Argentina  4.000000
## 7            Australia  6.273946
## 8              Austria  7.875000
## 9           Azerbeijan  4.000000
## 10          Bangladesh  1.000000
## 11             Belgium  3.777778
## 12              Bosnia  4.200000
## 13              Brazil  7.178527
## 14            Bulgaria  4.000000
## 15              Canada  7.921053
## 16               Chile  5.619048
## 17         Cote dIvoire  4.000000
## 18             Croatia  6.500000
## 19              Cyprus 13.000000
## 20      Czech Republic  6.800000
## 21             Denmark  4.833333
## 22               Egypt 12.000000
## 23             Finland  5.897590
## 24              France  5.276836
## 25             Georgia  9.000000
## 26             Germany  6.333333
## 27              Greece  8.500000
## 28           Hong Kong  8.000000
## 29             Hungary  5.000000
## 30               India  9.333333
```

```
## 31            Indonesia  8.111111
## 32              Ireland  6.500000
## 33               Israel  5.000000
## 34                Italy  8.197121
## 35                Japan  0.500000
## 36               Jordan  7.000000
## 37           Luxembourg  4.000000
## 38             Malaysia  6.500000
## 39               Mexico 10.750000
## 40               Monaco  4.500000
## 41           Montenegro 10.000000
## 42              Morocco 13.000000
## 43           Mozambique  7.000000
## 44          Netherlands  5.912929
## 45          New Zealand  6.144828
## 46            Nicaragua  5.000000
## 47               Norway  8.200000
## 48                 Oman  6.000000
## 49             Pakistan  3.000000
## 50                 Peru  2.000000
## 51          Philippines  6.250000
## 52               Poland  7.200000
## 53             Portugal  5.423423
## 54                Qatar  6.285714
## 55              Romania  5.600000
## 56         Saudi Arabia  4.000000
## 57               Serbia  4.500000
## 58            Singapore  7.750000
## 59             Slovakia  2.000000
## 60             Slovenia  2.000000
## 61         South Africa  6.666667
## 62                Spain  5.657143
## 63            Sri Lanka 15.000000
## 64               Sweden  5.454545
## 65          Switzerland  4.952381
## 66             Thailand  8.000000
## 67               Turkey  7.250000
## 68               Uganda 13.000000
## 69                   UK  7.475091
## 70 United Arab Emirates 14.000000
## 71                  USA  7.413538
## 72              Vietnam  2.000000
## 73           Yugoslavia  3.000000
## 74             Zimbabwe  5.000000
```

Dataset B, N>100 countries

```
# Bonding variables in N>100 countries
table(DatasetB_N100$bond_country, DatasetB_N100$country_now_Name)
```

```
##
##             Afghanistan Albania Algeria Argentina Armenia Australia Austria
```

```
##    0    0           0       0       0         0       0        94       0
##    1    0           0       0       0         0       0        41       0
##
##      Azerbaijan Bangladesh Belgium Belize Bermuda Bolivia Bosnia and Herzegovina
##    0          0        158       0      0       0       0                      0
##    1          0         97       0      0       0       0                      0
##
##      Brasil Bulgaria Canada Chile China Colombia Costa Rica Croatia
##    0      0        0     76     0     0        0          0       0
##    1      0        0     24     0     0        0          0       0
##
##      Czech Republic Denmark Ecuador Egypt Estonia Fiji Finland France Georgia
##    0              0       0       0     0       0    0       0    264       0
##    1              0       0       0     0       0    0       0     79       0
##
##      Germany Ghana Greece Guatemala Guernsey Guinea Guyana Hong Kong Hungary
##    0     194     0      0         0        0      0      0         0       0
##    1      24     0      0         0        0      0      0         0       0
##
##      Iceland India Indonesia Iran Iraq Ireland Israel Italy Japan Jersey Jordan
##    0       0     0         0    0    0       0      0    87     0      0      0
##    1       0     0         0    0    0       0      0    22     0      0      0
##
##      Kazakhstan Kenya Kuwait Kyrgyzstan Lebanon Lithuania Luxembourg Malaysia
##    0          0     0      0          0       0         0          0        0
##    1          0     0      0          0       0         0          0        0
##
##      Malta Mauritius Mexico Monaco Morocco Mozambique Namibia Nepal Netherlands
##    0     0         0      0      0       0          0       0     0           0
##    1     0         0      0      0       0          0       0     0           0
##
##      New Zealand Nigeria North Macedonia Norway Oman Pakistan Panama
##    0           0       0               0      0    0        0      0
##    1           0       0               0      0    0        0      0
##
##      Papua New Guinea Paraguay Peru Philippines Poland Portugal Puerto Rico
##    0                0        0  355           0      0        0           0
##    1                0        0  269           0      0        0           0
##
##      Qatar Romania Russia Saudi Arabia Senegal Serbia Singapore Slovakia
##    0     0       0      0            0       0      0         0        0
##    1     0       0      0            0       0      0         0        0
##
##      Slovenia Somalia South Africa South Korea Spain Sri Lanka Sudan Suriname
##    0        0       0            0           0     0         0     0        0
##    1        0       0            0           0     0         0     0        0
##
##      Sweden Switzerland Syria Taiwan Thailand Trinidad and Tobago Tunisia Turkey
##    0    104           0     0      0        0                   0       0    980
##    1     34           0     0      0        0                   0       0    131
##
##       UAE Uganda   UK Ukraine Uruguay  USA Vietnam Zambia
##    0    0      0 1525       0       0  481       0      0
##    1    0      0  393       0       0   55       0      0
```

```
table(DatasetB_N100$bond_gvmt, DatasetB_N100$country_now_Name)
```

```
##
##          Afghanistan Albania Algeria Argentina Armenia Australia Austria
##   0    0           0       0       0         0       0       125       0
##   1    0           0       0       0         0       0         9       0
##
##     Azerbaijan Bangladesh Belgium Belize Bermuda Bolivia Bosnia and Herzegovina
##   0          0        222       0      0       0       0                      0
##   1          0         19       0      0       0       0                      0
##
##     Brasil Bulgaria Canada Chile China Colombia Costa Rica Croatia
##   0      0        0     87     0     0        0          0       0
##   1      0        0     15     0     0        0          0       0
##
##     Czech Republic Denmark Ecuador Egypt Estonia Fiji Finland France Georgia
##   0              0       0       0     0       0    0       0    328       0
##   1              0       0       0     0       0    0       0     11       0
##
##     Germany Ghana Greece Guatemala Guernsey Guinea Guyana Hong Kong Hungary
##   0     204     0      0         0        0      0      0         0       0
##   1      11     0      0         0        0      0      0         0       0
##
##     Iceland India Indonesia Iran Iraq Ireland Israel Italy Japan Jersey Jordan
##   0       0     0         0    0    0       0      0    97     0      0      0
##   1       0     0         0    0    0       0      0    12     0      0      0
##
##     Kazakhstan Kenya Kuwait Kyrgyzstan Lebanon Lithuania Luxembourg Malaysia
##   0          0     0      0          0       0         0          0        0
##   1          0     0      0          0       0         0          0        0
##
##     Malta Mauritius Mexico Monaco Morocco Mozambique Namibia Nepal Netherlands
##   0     0         0      0      0       0          0       0     0           0
##   1     0         0      0      0       0          0       0     0           0
##
##     New Zealand Nigeria North Macedonia Norway Oman Pakistan Panama
##   0           0       0               0      0    0        0      0
##   1           0       0               0      0    0        0      0
##
##     Papua New Guinea Paraguay Peru Philippines Poland Portugal Puerto Rico
##   0                0        0  527           0      0        0           0
##   1                0        0   97           0      0        0           0
##
##     Qatar Romania Russia Saudi Arabia Senegal Serbia Singapore Slovakia
##   0     0       0      0            0       0      0         0        0
##   1     0       0      0            0       0      0         0        0
##
##     Slovenia Somalia South Africa South Korea Spain Sri Lanka Sudan Suriname
##   0        0       0            0           0     0         0     0        0
##   1        0       0            0           0     0         0     0        0
##
##     Sweden Switzerland Syria Taiwan Thailand Trinidad and Tobago Tunisia Turkey
##   0    126           0     0      0        0                   0       0   1013
```

```
##   1     12           0     0      0        0                   0       0     13
##
##      UAE Uganda   UK Ukraine Uruguay  USA Vietnam Zambia
##   0    0      0 1814       0       0  523       0      0
##   1    0      0  104       0       0   12       0      0
```

```
table(DatasetB_N100$bond_mult, DatasetB_N100$country_now_Name)
```

```
##
##          Afghanistan Albania Algeria Argentina Armenia Australia Austria
##   0    0           0       0       0         0       0        93       0
##   1    0           0       0       0         0       0        34       0
##   2    0           0       0       0         0       0         8       0
##
##     Azerbaijan Bangladesh Belgium Belize Bermuda Bolivia Bosnia and Herzegovina
##   0          0        158       0      0       0       0                      0
##   1          0         82       0      0       0       0                      0
##   2          0         17       0      0       0       0                      0
##
##     Brasil Bulgaria Canada Chile China Colombia Costa Rica Croatia
##   0      0        0     72     0     0        0          0       0
##   1      0        0     21     0     0        0          0       0
##   2      0        0      9     0     0        0          0       0
##
##     Czech Republic Denmark Ecuador Egypt Estonia Fiji Finland France Georgia
##   0              0       0       0     0       0    0       0    263       0
##   1              0       0       0     0       0    0       0     70       0
##   2              0       0       0     0       0    0       0     10       0
##
##     Germany Ghana Greece Guatemala Guernsey Guinea Guyana Hong Kong Hungary
##   0     190     0      0         0        0      0      0         0       0
##   1      21     0      0         0        0      0      0         0       0
##   2       7     0      0         0        0      0      0         0       0
##
##     Iceland India Indonesia Iran Iraq Ireland Israel Italy Japan Jersey Jordan
##   0       0     0         0    0    0       0      0    84     0      0      0
##   1       0     0         0    0    0       0      0    16     0      0      0
##   2       0     0         0    0    0       0      0     9     0      0      0
##
##     Kazakhstan Kenya Kuwait Kyrgyzstan Lebanon Lithuania Luxembourg Malaysia
##   0          0     0      0          0       0         0          0        0
##   1          0     0      0          0       0         0          0        0
##   2          0     0      0          0       0         0          0        0
##
##     Malta Mauritius Mexico Monaco Morocco Mozambique Namibia Nepal Netherlands
##   0     0         0      0      0       0          0       0     0           0
##   1     0         0      0      0       0          0       0     0           0
##   2     0         0      0      0       0          0       0     0           0
##
##     New Zealand Nigeria North Macedonia Norway Oman Pakistan Panama
##   0           0       0               0      0    0        0      0
##   1           0       0               0      0    0        0      0
##   2           0       0               0      0    0        0      0
##
```

```
##    Papua New Guinea Paraguay Peru Philippines Poland Portugal Puerto Rico
##   0                0        0  372           0      0        0           0
##   1                0        0  196           0      0        0           0
##   2                0        0   85           0      0        0           0
##
##    Qatar Romania Russia Saudi Arabia Senegal Serbia Singapore Slovakia
##   0     0       0      0            0       0      0         0        0
##   1     0       0      0            0       0      0         0        0
##   2     0       0      0            0       0      0         0        0
##
##    Slovenia Somalia South Africa South Korea Spain Sri Lanka Sudan Suriname
##   0        0       0            0           0     0         0     0        0
##   1        0       0            0           0     0         0     0        0
##   2        0       0            0           0     0         0     0        0
##
##    Sweden Switzerland Syria Taiwan Thailand Trinidad and Tobago Tunisia Turkey
##   0    104           0     0      0        0                   0       0    987
##   1     22           0     0      0        0                   0       0    120
##   2     12           0     0      0        0                   0       0     12
##
##     UAE Uganda   UK Ukraine Uruguay  USA Vietnam Zambia
##   0   0      0 1511       0       0  477       0      0
##   1   0      0  325       0       0   55       0      0
##   2   0      0   86       0       0    6       0      0
```

```
# Health behaviours in N>100 countries
table(DatasetB_N100$comply_self, DatasetB_N100$country_now_Name)
```

```
##
##            Afghanistan Albania Algeria Argentina Armenia Australia Austria
##   1      0           0       0       0         0       0         0       0
##   2      0           0       0       0         0       0         0       0
##   3      0           0       0       0         0       0         0       0
##   4      0           0       0       0         0       0         0       0
##   5      0           0       0       0         0       0         0       0
##   6      0           0       0       0         0       0         0       0
##   7      0           0       0       0         0       0         0       0
##   8      0           0       0       0         0       0         0       0
##   9      0           0       0       0         0       0         0       0
##   10     0           0       0       0         0       0         0       0
##   11     0           0       0       0         0       0         0       0
##   12     0           0       0       0         0       0         0       0
##   13     0           0       0       0         0       0         0       0
##   14     0           0       0       0         0       0         0       0
##   15     0           0       0       0         0       0         0       0
##   16     0           0       0       0         0       0         1       0
##   17     0           0       0       0         0       0         0       0
##   18     0           0       0       0         0       0         0       0
##   19     0           0       0       0         0       0         0       0
##   20     0           0       0       0         0       0         0       0
##   21     0           0       0       0         0       0         1       0
##   22     0           0       0       0         0       0         0       0
##   23     0           0       0       0         0       0         0       0
##   24     0           0       0       0         0       0         0       0
```

```
##    25  0              0       0       0         0       0         1       0
##    26  0              0       0       0         0       0         0       0
##    27  0              0       0       0         0       0         1       0
##    28  0              0       0       0         0       0         0       0
##    29  0              0       0       0         0       0         0       0
##    30  0              0       0       0         0       0         1       0
##    31  0              0       0       0         0       0         0       0
##    32  0              0       0       0         0       0         1       0
##    33  0              0       0       0         0       0         0       0
##    34  0              0       0       0         0       0         0       0
##    35  0              0       0       0         0       0         1       0
##    36  0              0       0       0         0       0         2       0
##    37  0              0       0       0         0       0         2       0
##    38  0              0       0       0         0       0         1       0
##    39  0              0       0       0         0       0         2       0
##    40  0              0       0       0         0       0         0       0
##    41  0              0       0       0         0       0         3       0
##    42  0              0       0       0         0       0         2       0
##    43  0              0       0       0         0       0         0       0
##    44  0              0       0       0         0       0         1       0
##    45  0              0       0       0         0       0         1       0
##    46  0              0       0       0         0       0         0       0
##    47  0              0       0       0         0       0         0       0
##    48  0              0       0       0         0       0         1       0
##    49  0              0       0       0         0       0         4       0
##    50  0              0       0       0         0       0        29       0
##    51  0              0       0       0         0       0         2       0
##    52  0              0       0       0         0       0         3       0
##    53  0              0       0       0         0       0         5       0
##    54  0              0       0       0         0       0         1       0
##    55  0              0       0       0         0       0         4       0
##    56  0              0       0       0         0       0         1       0
##    57  0              0       0       0         0       0         0       0
##    58  0              0       0       0         0       0         1       0
##    59  0              0       0       0         0       0         1       0
##    60  0              0       0       0         0       0         1       0
##    61  0              0       0       0         0       0         1       0
##    62  0              0       0       0         0       0         1       0
##    63  0              0       0       0         0       0         2       0
##    64  0              0       0       0         0       0         1       0
##    65  0              0       0       0         0       0         3       0
##    66  0              0       0       0         0       0         3       0
##    67  0              0       0       0         0       0         0       0
##    68  0              0       0       0         0       0         3       0
##    69  0              0       0       0         0       0         2       0
##    70  0              0       0       0         0       0         3       0
##    71  0              0       0       0         0       0         1       0
##    72  0              0       0       0         0       0         3       0
##    73  0              0       0       0         0       0         0       0
##    74  0              0       0       0         0       0         2       0
##    75  0              0       0       0         0       0         2       0
##    76  0              0       0       0         0       0         1       0
##    77  0              0       0       0         0       0         0       0
##    78  0              0       0       0         0       0         0       0
```

```
##    79  0          0       0       0         0       0         1       0
##    80  0          0       0       0         0       0         1       0
##    81  0          0       0       0         0       0         0       0
##    82  0          0       0       0         0       0         0       0
##    83  0          0       0       0         0       0         0       0
##    84  0          0       0       0         0       0         4       0
##    85  0          0       0       0         0       0         0       0
##    86  0          0       0       0         0       0         2       0
##    87  0          0       0       0         0       0         0       0
##    88  0          0       0       0         0       0         0       0
##    89  0          0       0       0         0       0         1       0
##    90  0          0       0       0         0       0         1       0
##    91  0          0       0       0         0       0         0       0
##    92  0          0       0       0         0       0         0       0
##    93  0          0       0       0         0       0         5       0
##    94  0          0       0       0         0       0         0       0
##    95  0          0       0       0         0       0         3       0
##    96  0          0       0       0         0       0         1       0
##    97  0          0       0       0         0       0         2       0
##    98  0          0       0       0         0       0         0       0
##    99  0          0       0       0         0       0         0       0
##    100 0          0       0       0         0       0        12       0
##
##       Azerbaijan Bangladesh Belgium Belize Bermuda Bolivia
##    1           0          1       0      0       0       0
##    2           0          0       0      0       0       0
##    3           0          0       0      0       0       0
##    4           0          0       0      0       0       0
##    5           0          1       0      0       0       0
##    6           0          1       0      0       0       0
##    7           0          0       0      0       0       0
##    8           0          1       0      0       0       0
##    9           0          2       0      0       0       0
##    10          0          1       0      0       0       0
##    11          0          0       0      0       0       0
##    12          0          2       0      0       0       0
##    13          0          0       0      0       0       0
##    14          0          1       0      0       0       0
##    15          0          0       0      0       0       0
##    16          0          0       0      0       0       0
##    17          0          0       0      0       0       0
##    18          0          1       0      0       0       0
##    19          0          0       0      0       0       0
##    20          0          0       0      0       0       0
##    21          0          0       0      0       0       0
##    22          0          0       0      0       0       0
##    23          0          1       0      0       0       0
##    24          0          1       0      0       0       0
##    25          0          3       0      0       0       0
##    26          0          0       0      0       0       0
##    27          0          0       0      0       0       0
##    28          0          2       0      0       0       0
##    29          0          1       0      0       0       0
##    30          0          2       0      0       0       0
```

```
##   31          0          1     0     0     0     0
##   32          0          4     0     0     0     0
##   33          0          3     0     0     0     0
##   34          0          1     0     0     0     0
##   35          0          2     0     0     0     0
##   36          0          0     0     0     0     0
##   37          0          3     0     0     0     0
##   38          0          1     0     0     0     0
##   39          0          2     0     0     0     0
##   40          0          3     0     0     0     0
##   41          0          2     0     0     0     0
##   42          0          3     0     0     0     0
##   43          0          0     0     0     0     0
##   44          0          1     0     0     0     0
##   45          0          2     0     0     0     0
##   46          0          2     0     0     0     0
##   47          0          2     0     0     0     0
##   48          0          8     0     0     0     0
##   49          0         12     0     0     0     0
##   50          0         85     0     0     0     0
##   51          0         12     0     0     0     0
##   52          0         14     0     0     0     0
##   53          0          6     0     0     0     0
##   54          0          1     0     0     0     0
##   55          0          1     0     0     0     0
##   56          0          2     0     0     0     0
##   57          0          1     0     0     0     0
##   58          0          0     0     0     0     0
##   59          0          3     0     0     0     0
##   60          0          1     0     0     0     0
##   61          0          0     0     0     0     0
##   62          0          1     0     0     0     0
##   63          0          1     0     0     0     0
##   64          0          1     0     0     0     0
##   65          0          1     0     0     0     0
##   66          0          2     0     0     0     0
##   67          0          2     0     0     0     0
##   68          0          1     0     0     0     0
##   69          0          3     0     0     0     0
##   70          0          4     0     0     0     0
##   71          0          5     0     0     0     0
##   72          0          1     0     0     0     0
##   73          0          2     0     0     0     0
##   74          0          0     0     0     0     0
##   75          0          3     0     0     0     0
##   76          0          1     0     0     0     0
##   77          0          0     0     0     0     0
##   78          0          0     0     0     0     0
##   79          0          1     0     0     0     0
##   80          0          1     0     0     0     0
##   81          0          4     0     0     0     0
##   82          0          0     0     0     0     0
##   83          0          0     0     0     0     0
##   84          0          0     0     0     0     0
```

```
##   85           0          0       0       0       0       0
##   86           0          0       0       0       0       0
##   87           0          1       0       0       0       0
##   88           0          1       0       0       0       0
##   89           0          2       0       0       0       0
##   90           0          4       0       0       0       0
##   91           0          4       0       0       0       0
##   92           0          2       0       0       0       0
##   93           0          4       0       0       0       0
##   94           0          3       0       0       0       0
##   95           0          0       0       0       0       0
##   96           0          5       0       0       0       0
##   97           0          1       0       0       0       0
##   98           0          1       0       0       0       0
##   99           0          1       0       0       0       0
##   100          0         13       0       0       0       0
##
##       Bosnia and Herzegovina Brasil Bulgaria Canada Chile China Colombia
##   1                        0      0        0      0     0     0        0
##   2                        0      0        0      0     0     0        0
##   3                        0      0        0      0     0     0        0
##   4                        0      0        0      0     0     0        0
##   5                        0      0        0      0     0     0        0
##   6                        0      0        0      0     0     0        0
##   7                        0      0        0      0     0     0        0
##   8                        0      0        0      0     0     0        0
##   9                        0      0        0      0     0     0        0
##   10                       0      0        0      0     0     0        0
##   11                       0      0        0      0     0     0        0
##   12                       0      0        0      0     0     0        0
##   13                       0      0        0      0     0     0        0
##   14                       0      0        0      1     0     0        0
##   15                       0      0        0      0     0     0        0
##   16                       0      0        0      0     0     0        0
##   17                       0      0        0      0     0     0        0
##   18                       0      0        0      0     0     0        0
##   19                       0      0        0      0     0     0        0
##   20                       0      0        0      0     0     0        0
##   21                       0      0        0      1     0     0        0
##   22                       0      0        0      0     0     0        0
##   23                       0      0        0      0     0     0        0
##   24                       0      0        0      0     0     0        0
##   25                       0      0        0      0     0     0        0
##   26                       0      0        0      0     0     0        0
##   27                       0      0        0      2     0     0        0
##   28                       0      0        0      0     0     0        0
##   29                       0      0        0      2     0     0        0
##   30                       0      0        0      0     0     0        0
##   31                       0      0        0      0     0     0        0
##   32                       0      0        0      0     0     0        0
##   33                       0      0        0      0     0     0        0
##   34                       0      0        0      0     0     0        0
##   35                       0      0        0      0     0     0        0
##   36                       0      0        0      0     0     0        0
```

```
##    37                          0       0         0      2     0     0         0
##    38                          0       0         0      3     0     0         0
##    39                          0       0         0      1     0     0         0
##    40                          0       0         0      1     0     0         0
##    41                          0       0         0      1     0     0         0
##    42                          0       0         0      2     0     0         0
##    43                          0       0         0      1     0     0         0
##    44                          0       0         0      2     0     0         0
##    45                          0       0         0      1     0     0         0
##    46                          0       0         0      1     0     0         0
##    47                          0       0         0      0     0     0         0
##    48                          0       0         0      3     0     0         0
##    49                          0       0         0      3     0     0         0
##    50                          0       0         0     10     0     0         0
##    51                          0       0         0      3     0     0         0
##    52                          0       0         0      3     0     0         0
##    53                          0       0         0      3     0     0         0
##    54                          0       0         0      1     0     0         0
##    55                          0       0         0      2     0     0         0
##    56                          0       0         0      2     0     0         0
##    57                          0       0         0      0     0     0         0
##    58                          0       0         0      2     0     0         0
##    59                          0       0         0      1     0     0         0
##    60                          0       0         0      0     0     0         0
##    61                          0       0         0      1     0     0         0
##    62                          0       0         0      0     0     0         0
##    63                          0       0         0      0     0     0         0
##    64                          0       0         0      2     0     0         0
##    65                          0       0         0      1     0     0         0
##    66                          0       0         0      0     0     0         0
##    67                          0       0         0      1     0     0         0
##    68                          0       0         0      0     0     0         0
##    69                          0       0         0      2     0     0         0
##    70                          0       0         0      2     0     0         0
##    71                          0       0         0      1     0     0         0
##    72                          0       0         0      0     0     0         0
##    73                          0       0         0      0     0     0         0
##    74                          0       0         0      1     0     0         0
##    75                          0       0         0      2     0     0         0
##    76                          0       0         0      0     0     0         0
##    77                          0       0         0      1     0     0         0
##    78                          0       0         0      4     0     0         0
##    79                          0       0         0      0     0     0         0
##    80                          0       0         0      1     0     0         0
##    81                          0       0         0      4     0     0         0
##    82                          0       0         0      1     0     0         0
##    83                          0       0         0      1     0     0         0
##    84                          0       0         0      0     0     0         0
##    85                          0       0         0      1     0     0         0
##    86                          0       0         0      0     0     0         0
##    87                          0       0         0      1     0     0         0
##    88                          0       0         0      1     0     0         0
##    89                          0       0         0      1     0     0         0
##    90                          0       0         0      2     0     0         0
```

```
##   91                       0      0        0      0     0     0        0
##   92                       0      0        0      0     0     0        0
##   93                       0      0        0      0     0     0        0
##   94                       0      0        0      1     0     0        0
##   95                       0      0        0      1     0     0        0
##   96                       0      0        0      1     0     0        0
##   97                       0      0        0      0     0     0        0
##   98                       0      0        0      1     0     0        0
##   99                       0      0        0      2     0     0        0
##   100                      0      0        0     14     0     0        0
## 
##       Costa Rica Croatia Czech Republic Denmark Ecuador Egypt Estonia Fiji
##   1            0       0              0       0       0     0       0    0
##   2            0       0              0       0       0     0       0    0
##   3            0       0              0       0       0     0       0    0
##   4            0       0              0       0       0     0       0    0
##   5            0       0              0       0       0     0       0    0
##   6            0       0              0       0       0     0       0    0
##   7            0       0              0       0       0     0       0    0
##   8            0       0              0       0       0     0       0    0
##   9            0       0              0       0       0     0       0    0
##   10           0       0              0       0       0     0       0    0
##   11           0       0              0       0       0     0       0    0
##   12           0       0              0       0       0     0       0    0
##   13           0       0              0       0       0     0       0    0
##   14           0       0              0       0       0     0       0    0
##   15           0       0              0       0       0     0       0    0
##   16           0       0              0       0       0     0       0    0
##   17           0       0              0       0       0     0       0    0
##   18           0       0              0       0       0     0       0    0
##   19           0       0              0       0       0     0       0    0
##   20           0       0              0       0       0     0       0    0
##   21           0       0              0       0       0     0       0    0
##   22           0       0              0       0       0     0       0    0
##   23           0       0              0       0       0     0       0    0
##   24           0       0              0       0       0     0       0    0
##   25           0       0              0       0       0     0       0    0
##   26           0       0              0       0       0     0       0    0
##   27           0       0              0       0       0     0       0    0
##   28           0       0              0       0       0     0       0    0
##   29           0       0              0       0       0     0       0    0
##   30           0       0              0       0       0     0       0    0
##   31           0       0              0       0       0     0       0    0
##   32           0       0              0       0       0     0       0    0
##   33           0       0              0       0       0     0       0    0
##   34           0       0              0       0       0     0       0    0
##   35           0       0              0       0       0     0       0    0
##   36           0       0              0       0       0     0       0    0
##   37           0       0              0       0       0     0       0    0
##   38           0       0              0       0       0     0       0    0
##   39           0       0              0       0       0     0       0    0
##   40           0       0              0       0       0     0       0    0
##   41           0       0              0       0       0     0       0    0
##   42           0       0              0       0       0     0       0    0
```

```
##    43           0    0          0    0    0   0    0  0
##    44           0    0          0    0    0   0    0  0
##    45           0    0          0    0    0   0    0  0
##    46           0    0          0    0    0   0    0  0
##    47           0    0          0    0    0   0    0  0
##    48           0    0          0    0    0   0    0  0
##    49           0    0          0    0    0   0    0  0
##    50           0    0          0    0    0   0    0  0
##    51           0    0          0    0    0   0    0  0
##    52           0    0          0    0    0   0    0  0
##    53           0    0          0    0    0   0    0  0
##    54           0    0          0    0    0   0    0  0
##    55           0    0          0    0    0   0    0  0
##    56           0    0          0    0    0   0    0  0
##    57           0    0          0    0    0   0    0  0
##    58           0    0          0    0    0   0    0  0
##    59           0    0          0    0    0   0    0  0
##    60           0    0          0    0    0   0    0  0
##    61           0    0          0    0    0   0    0  0
##    62           0    0          0    0    0   0    0  0
##    63           0    0          0    0    0   0    0  0
##    64           0    0          0    0    0   0    0  0
##    65           0    0          0    0    0   0    0  0
##    66           0    0          0    0    0   0    0  0
##    67           0    0          0    0    0   0    0  0
##    68           0    0          0    0    0   0    0  0
##    69           0    0          0    0    0   0    0  0
##    70           0    0          0    0    0   0    0  0
##    71           0    0          0    0    0   0    0  0
##    72           0    0          0    0    0   0    0  0
##    73           0    0          0    0    0   0    0  0
##    74           0    0          0    0    0   0    0  0
##    75           0    0          0    0    0   0    0  0
##    76           0    0          0    0    0   0    0  0
##    77           0    0          0    0    0   0    0  0
##    78           0    0          0    0    0   0    0  0
##    79           0    0          0    0    0   0    0  0
##    80           0    0          0    0    0   0    0  0
##    81           0    0          0    0    0   0    0  0
##    82           0    0          0    0    0   0    0  0
##    83           0    0          0    0    0   0    0  0
##    84           0    0          0    0    0   0    0  0
##    85           0    0          0    0    0   0    0  0
##    86           0    0          0    0    0   0    0  0
##    87           0    0          0    0    0   0    0  0
##    88           0    0          0    0    0   0    0  0
##    89           0    0          0    0    0   0    0  0
##    90           0    0          0    0    0   0    0  0
##    91           0    0          0    0    0   0    0  0
##    92           0    0          0    0    0   0    0  0
##    93           0    0          0    0    0   0    0  0
##    94           0    0          0    0    0   0    0  0
##    95           0    0          0    0    0   0    0  0
##    96           0    0          0    0    0   0    0  0
```

```
##   97            0       0              0       0       0      0       0    0
##   98            0       0              0       0       0      0       0    0
##   99            0       0              0       0       0      0       0    0
##   100           0       0              0       0       0      0       0    0
##
##       Finland France Georgia Germany Ghana Greece Guatemala Guernsey Guinea
##   1         0      1       0       1     0      0         0        0      0
##   2         0      0       0       0     0      0         0        0      0
##   3         0      0       0       0     0      0         0        0      0
##   4         0      0       0       0     0      0         0        0      0
##   5         0      0       0       0     0      0         0        0      0
##   6         0      0       0       0     0      0         0        0      0
##   7         0      0       0       0     0      0         0        0      0
##   8         0      0       0       0     0      0         0        0      0
##   9         0      1       0       0     0      0         0        0      0
##   10        0      1       0       0     0      0         0        0      0
##   11        0      0       0       0     0      0         0        0      0
##   12        0      0       0       0     0      0         0        0      0
##   13        0      0       0       0     0      0         0        0      0
##   14        0      0       0       0     0      0         0        0      0
##   15        0      1       0       0     0      0         0        0      0
##   16        0      0       0       0     0      0         0        0      0
##   17        0      0       0       1     0      0         0        0      0
##   18        0      0       0       0     0      0         0        0      0
##   19        0      1       0       0     0      0         0        0      0
##   20        0      1       0       1     0      0         0        0      0
##   21        0      0       0       0     0      0         0        0      0
##   22        0      0       0       0     0      0         0        0      0
##   23        0      0       0       1     0      0         0        0      0
##   24        0      1       0       1     0      0         0        0      0
##   25        0      0       0       1     0      0         0        0      0
##   26        0      0       0       3     0      0         0        0      0
##   27        0      1       0       2     0      0         0        0      0
##   28        0      1       0       0     0      0         0        0      0
##   29        0      0       0       1     0      0         0        0      0
##   30        0      2       0       1     0      0         0        0      0
##   31        0      1       0       0     0      0         0        0      0
##   32        0      5       0       1     0      0         0        0      0
##   33        0      2       0       0     0      0         0        0      0
##   34        0      5       0       1     0      0         0        0      0
##   35        0      2       0       1     0      0         0        0      0
##   36        0      6       0       2     0      0         0        0      0
##   37        0      4       0       4     0      0         0        0      0
##   38        0      2       0       2     0      0         0        0      0
##   39        0      4       0       2     0      0         0        0      0
##   40        0      1       0       3     0      0         0        0      0
##   41        0      2       0       3     0      0         0        0      0
##   42        0      6       0       5     0      0         0        0      0
##   43        0      1       0       2     0      0         0        0      0
##   44        0      2       0       9     0      0         0        0      0
##   45        0      5       0       1     0      0         0        0      0
##   46        0      5       0       5     0      0         0        0      0
##   47        0      3       0       6     0      0         0        0      0
##   48        0      7       0       5     0      0         0        0      0
```

```
##   49         0      11         0        3     0       0          0        0      0
##   50         0      37         0       21     0       0          0        0      0
##   51         0      15         0       10     0       0          0        0      0
##   52         0       4         0        6     0       0          0        0      0
##   53         0      10         0        4     0       0          0        0      0
##   54         0      11         0        0     0       0          0        0      0
##   55         0       4         0        4     0       0          0        0      0
##   56         0       3         0        1     0       0          0        0      0
##   57         0       4         0        0     0       0          0        0      0
##   58         0      10         0        2     0       0          0        0      0
##   59         0       4         0        1     0       0          0        0      0
##   60         0       8         0        3     0       0          0        0      0
##   61         0       4         0        2     0       0          0        0      0
##   62         0       4         0        1     0       0          0        0      0
##   63         0       3         0        1     0       0          0        0      0
##   64         0       2         0        1     0       0          0        0      0
##   65         0       1         0        6     0       0          0        0      0
##   66         0       4         0        1     0       0          0        0      0
##   67         0       3         0        3     0       0          0        0      0
##   68         0       5         0        2     0       0          0        0      0
##   69         0       7         0        4     0       0          0        0      0
##   70         0       8         0        1     0       0          0        0      0
##   71         0       3         0        4     0       0          0        0      0
##   72         0       5         0        4     0       0          0        0      0
##   73         0       4         0        2     0       0          0        0      0
##   74         0       5         0        2     0       0          0        0      0
##   75         0       3         0        5     0       0          0        0      0
##   76         0       2         0        1     0       0          0        0      0
##   77         0       6         0        1     0       0          0        0      0
##   78         0       3         0        1     0       0          0        0      0
##   79         0       3         0        4     0       0          0        0      0
##   80         0       2         0        3     0       0          0        0      0
##   81         0       4         0        1     0       0          0        0      0
##   82         0       3         0        1     0       0          0        0      0
##   83         0       3         0        4     0       0          0        0      0
##   84         0       1         0        1     0       0          0        0      0
##   85         0       4         0        2     0       0          0        0      0
##   86         0       0         0        2     0       0          0        0      0
##   87         0       4         0        3     0       0          0        0      0
##   88         0       4         0        6     0       0          0        0      0
##   89         0       3         0        2     0       0          0        0      0
##   90         0       2         0        3     0       0          0        0      0
##   91         0       3         0        1     0       0          0        0      0
##   92         0       3         0        0     0       0          0        0      0
##   93         0       2         0        2     0       0          0        0      0
##   94         0       1         0        3     0       0          0        0      0
##   95         0       2         0        0     0       0          0        0      0
##   96         0       3         0        0     0       0          0        0      0
##   97         0       3         0        1     0       0          0        0      0
##   98         0       2         0        0     0       0          0        0      0
##   99         0       4         0        1     0       0          0        0      0
##   100        0      21         0       19     0       0          0        0      0
##
##       Guyana Hong Kong Hungary Iceland India Indonesia Iran Iraq Ireland Israel
```

```
## 1      0     0     0     0   0     0  0  0    0    0
## 2      0     0     0     0   0     0  0  0    0    0
## 3      0     0     0     0   0     0  0  0    0    0
## 4      0     0     0     0   0     0  0  0    0    0
## 5      0     0     0     0   0     0  0  0    0    0
## 6      0     0     0     0   0     0  0  0    0    0
## 7      0     0     0     0   0     0  0  0    0    0
## 8      0     0     0     0   0     0  0  0    0    0
## 9      0     0     0     0   0     0  0  0    0    0
## 10     0     0     0     0   0     0  0  0    0    0
## 11     0     0     0     0   0     0  0  0    0    0
## 12     0     0     0     0   0     0  0  0    0    0
## 13     0     0     0     0   0     0  0  0    0    0
## 14     0     0     0     0   0     0  0  0    0    0
## 15     0     0     0     0   0     0  0  0    0    0
## 16     0     0     0     0   0     0  0  0    0    0
## 17     0     0     0     0   0     0  0  0    0    0
## 18     0     0     0     0   0     0  0  0    0    0
## 19     0     0     0     0   0     0  0  0    0    0
## 20     0     0     0     0   0     0  0  0    0    0
## 21     0     0     0     0   0     0  0  0    0    0
## 22     0     0     0     0   0     0  0  0    0    0
## 23     0     0     0     0   0     0  0  0    0    0
## 24     0     0     0     0   0     0  0  0    0    0
## 25     0     0     0     0   0     0  0  0    0    0
## 26     0     0     0     0   0     0  0  0    0    0
## 27     0     0     0     0   0     0  0  0    0    0
## 28     0     0     0     0   0     0  0  0    0    0
## 29     0     0     0     0   0     0  0  0    0    0
## 30     0     0     0     0   0     0  0  0    0    0
## 31     0     0     0     0   0     0  0  0    0    0
## 32     0     0     0     0   0     0  0  0    0    0
## 33     0     0     0     0   0     0  0  0    0    0
## 34     0     0     0     0   0     0  0  0    0    0
## 35     0     0     0     0   0     0  0  0    0    0
## 36     0     0     0     0   0     0  0  0    0    0
## 37     0     0     0     0   0     0  0  0    0    0
## 38     0     0     0     0   0     0  0  0    0    0
## 39     0     0     0     0   0     0  0  0    0    0
## 40     0     0     0     0   0     0  0  0    0    0
## 41     0     0     0     0   0     0  0  0    0    0
## 42     0     0     0     0   0     0  0  0    0    0
## 43     0     0     0     0   0     0  0  0    0    0
## 44     0     0     0     0   0     0  0  0    0    0
## 45     0     0     0     0   0     0  0  0    0    0
## 46     0     0     0     0   0     0  0  0    0    0
## 47     0     0     0     0   0     0  0  0    0    0
## 48     0     0     0     0   0     0  0  0    0    0
## 49     0     0     0     0   0     0  0  0    0    0
## 50     0     0     0     0   0     0  0  0    0    0
## 51     0     0     0     0   0     0  0  0    0    0
## 52     0     0     0     0   0     0  0  0    0    0
## 53     0     0     0     0   0     0  0  0    0    0
## 54     0     0     0     0   0     0  0  0    0    0
```

```
##   55       0         0       0       0     0         0    0    0       0      0
##   56       0         0       0       0     0         0    0    0       0      0
##   57       0         0       0       0     0         0    0    0       0      0
##   58       0         0       0       0     0         0    0    0       0      0
##   59       0         0       0       0     0         0    0    0       0      0
##   60       0         0       0       0     0         0    0    0       0      0
##   61       0         0       0       0     0         0    0    0       0      0
##   62       0         0       0       0     0         0    0    0       0      0
##   63       0         0       0       0     0         0    0    0       0      0
##   64       0         0       0       0     0         0    0    0       0      0
##   65       0         0       0       0     0         0    0    0       0      0
##   66       0         0       0       0     0         0    0    0       0      0
##   67       0         0       0       0     0         0    0    0       0      0
##   68       0         0       0       0     0         0    0    0       0      0
##   69       0         0       0       0     0         0    0    0       0      0
##   70       0         0       0       0     0         0    0    0       0      0
##   71       0         0       0       0     0         0    0    0       0      0
##   72       0         0       0       0     0         0    0    0       0      0
##   73       0         0       0       0     0         0    0    0       0      0
##   74       0         0       0       0     0         0    0    0       0      0
##   75       0         0       0       0     0         0    0    0       0      0
##   76       0         0       0       0     0         0    0    0       0      0
##   77       0         0       0       0     0         0    0    0       0      0
##   78       0         0       0       0     0         0    0    0       0      0
##   79       0         0       0       0     0         0    0    0       0      0
##   80       0         0       0       0     0         0    0    0       0      0
##   81       0         0       0       0     0         0    0    0       0      0
##   82       0         0       0       0     0         0    0    0       0      0
##   83       0         0       0       0     0         0    0    0       0      0
##   84       0         0       0       0     0         0    0    0       0      0
##   85       0         0       0       0     0         0    0    0       0      0
##   86       0         0       0       0     0         0    0    0       0      0
##   87       0         0       0       0     0         0    0    0       0      0
##   88       0         0       0       0     0         0    0    0       0      0
##   89       0         0       0       0     0         0    0    0       0      0
##   90       0         0       0       0     0         0    0    0       0      0
##   91       0         0       0       0     0         0    0    0       0      0
##   92       0         0       0       0     0         0    0    0       0      0
##   93       0         0       0       0     0         0    0    0       0      0
##   94       0         0       0       0     0         0    0    0       0      0
##   95       0         0       0       0     0         0    0    0       0      0
##   96       0         0       0       0     0         0    0    0       0      0
##   97       0         0       0       0     0         0    0    0       0      0
##   98       0         0       0       0     0         0    0    0       0      0
##   99       0         0       0       0     0         0    0    0       0      0
##   100      0         0       0       0     0         0    0    0       0      0
##
##       Italy Japan Jersey Jordan Kazakhstan Kenya Kuwait Kyrgyzstan Lebanon
##   1       2     0      0      0          0     0      0          0       0
##   2       1     0      0      0          0     0      0          0       0
##   3       1     0      0      0          0     0      0          0       0
##   4       0     0      0      0          0     0      0          0       0
##   5       0     0      0      0          0     0      0          0       0
##   6       1     0      0      0          0     0      0          0       0
```

```
##   7        0    0     0     0          0    0     0          0       0
##   8        0    0     0     0          0    0     0          0       0
##   9        0    0     0     0          0    0     0          0       0
##   10       0    0     0     0          0    0     0          0       0
##   11       0    0     0     0          0    0     0          0       0
##   12       0    0     0     0          0    0     0          0       0
##   13       0    0     0     0          0    0     0          0       0
##   14       0    0     0     0          0    0     0          0       0
##   15       0    0     0     0          0    0     0          0       0
##   16       0    0     0     0          0    0     0          0       0
##   17       0    0     0     0          0    0     0          0       0
##   18       0    0     0     0          0    0     0          0       0
##   19       1    0     0     0          0    0     0          0       0
##   20       0    0     0     0          0    0     0          0       0
##   21       0    0     0     0          0    0     0          0       0
##   22       0    0     0     0          0    0     0          0       0
##   23       0    0     0     0          0    0     0          0       0
##   24       0    0     0     0          0    0     0          0       0
##   25       0    0     0     0          0    0     0          0       0
##   26       0    0     0     0          0    0     0          0       0
##   27       2    0     0     0          0    0     0          0       0
##   28       0    0     0     0          0    0     0          0       0
##   29       0    0     0     0          0    0     0          0       0
##   30       0    0     0     0          0    0     0          0       0
##   31       0    0     0     0          0    0     0          0       0
##   32       0    0     0     0          0    0     0          0       0
##   33       0    0     0     0          0    0     0          0       0
##   34       3    0     0     0          0    0     0          0       0
##   35       1    0     0     0          0    0     0          0       0
##   36       0    0     0     0          0    0     0          0       0
##   37       1    0     0     0          0    0     0          0       0
##   38       1    0     0     0          0    0     0          0       0
##   39       1    0     0     0          0    0     0          0       0
##   40       1    0     0     0          0    0     0          0       0
##   41       0    0     0     0          0    0     0          0       0
##   42       1    0     0     0          0    0     0          0       0
##   43       2    0     0     0          0    0     0          0       0
##   44       2    0     0     0          0    0     0          0       0
##   45       0    0     0     0          0    0     0          0       0
##   46       0    0     0     0          0    0     0          0       0
##   47       1    0     0     0          0    0     0          0       0
##   48       1    0     0     0          0    0     0          0       0
##   49       3    0     0     0          0    0     0          0       0
##   50      20    0     0     0          0    0     0          0       0
##   51       3    0     0     0          0    0     0          0       0
##   52       3    0     0     0          0    0     0          0       0
##   53       0    0     0     0          0    0     0          0       0
##   54       2    0     0     0          0    0     0          0       0
##   55       1    0     0     0          0    0     0          0       0
##   56       2    0     0     0          0    0     0          0       0
##   57       2    0     0     0          0    0     0          0       0
##   58       2    0     0     0          0    0     0          0       0
##   59       1    0     0     0          0    0     0          0       0
##   60       0    0     0     0          0    0     0          0       0
```

```
##   61      2     0      0      0          0     0      0          0       0
##   62      4     0      0      0          0     0      0          0       0
##   63      1     0      0      0          0     0      0          0       0
##   64      1     0      0      0          0     0      0          0       0
##   65      1     0      0      0          0     0      0          0       0
##   66      0     0      0      0          0     0      0          0       0
##   67      0     0      0      0          0     0      0          0       0
##   68      0     0      0      0          0     0      0          0       0
##   69      1     0      0      0          0     0      0          0       0
##   70      2     0      0      0          0     0      0          0       0
##   71      3     0      0      0          0     0      0          0       0
##   72      2     0      0      0          0     0      0          0       0
##   73      3     0      0      0          0     0      0          0       0
##   74      1     0      0      0          0     0      0          0       0
##   75      0     0      0      0          0     0      0          0       0
##   76      2     0      0      0          0     0      0          0       0
##   77      1     0      0      0          0     0      0          0       0
##   78      0     0      0      0          0     0      0          0       0
##   79      1     0      0      0          0     0      0          0       0
##   80      1     0      0      0          0     0      0          0       0
##   81      1     0      0      0          0     0      0          0       0
##   82      1     0      0      0          0     0      0          0       0
##   83      1     0      0      0          0     0      0          0       0
##   84      0     0      0      0          0     0      0          0       0
##   85      1     0      0      0          0     0      0          0       0
##   86      0     0      0      0          0     0      0          0       0
##   87      1     0      0      0          0     0      0          0       0
##   88      0     0      0      0          0     0      0          0       0
##   89      1     0      0      0          0     0      0          0       0
##   90      0     0      0      0          0     0      0          0       0
##   91      0     0      0      0          0     0      0          0       0
##   92      1     0      0      0          0     0      0          0       0
##   93      0     0      0      0          0     0      0          0       0
##   94      1     0      0      0          0     0      0          0       0
##   95      2     0      0      0          0     0      0          0       0
##   96      0     0      0      0          0     0      0          0       0
##   97      1     0      0      0          0     0      0          0       0
##   98      0     0      0      0          0     0      0          0       0
##   99      2     0      0      0          0     0      0          0       0
##   100    11     0      0      0          0     0      0          0       0
##
##       Lithuania Luxembourg Malaysia Malta Mauritius Mexico Monaco Morocco
##   1           0          0        0     0         0      0      0       0
##   2           0          0        0     0         0      0      0       0
##   3           0          0        0     0         0      0      0       0
##   4           0          0        0     0         0      0      0       0
##   5           0          0        0     0         0      0      0       0
##   6           0          0        0     0         0      0      0       0
##   7           0          0        0     0         0      0      0       0
##   8           0          0        0     0         0      0      0       0
##   9           0          0        0     0         0      0      0       0
##   10          0          0        0     0         0      0      0       0
##   11          0          0        0     0         0      0      0       0
##   12          0          0        0     0         0      0      0       0
```

```
## 13 0 0 0 0 0 0 0 0
## 14 0 0 0 0 0 0 0 0
## 15 0 0 0 0 0 0 0 0
## 16 0 0 0 0 0 0 0 0
## 17 0 0 0 0 0 0 0 0
## 18 0 0 0 0 0 0 0 0
## 19 0 0 0 0 0 0 0 0
## 20 0 0 0 0 0 0 0 0
## 21 0 0 0 0 0 0 0 0
## 22 0 0 0 0 0 0 0 0
## 23 0 0 0 0 0 0 0 0
## 24 0 0 0 0 0 0 0 0
## 25 0 0 0 0 0 0 0 0
## 26 0 0 0 0 0 0 0 0
## 27 0 0 0 0 0 0 0 0
## 28 0 0 0 0 0 0 0 0
## 29 0 0 0 0 0 0 0 0
## 30 0 0 0 0 0 0 0 0
## 31 0 0 0 0 0 0 0 0
## 32 0 0 0 0 0 0 0 0
## 33 0 0 0 0 0 0 0 0
## 34 0 0 0 0 0 0 0 0
## 35 0 0 0 0 0 0 0 0
## 36 0 0 0 0 0 0 0 0
## 37 0 0 0 0 0 0 0 0
## 38 0 0 0 0 0 0 0 0
## 39 0 0 0 0 0 0 0 0
## 40 0 0 0 0 0 0 0 0
## 41 0 0 0 0 0 0 0 0
## 42 0 0 0 0 0 0 0 0
## 43 0 0 0 0 0 0 0 0
## 44 0 0 0 0 0 0 0 0
## 45 0 0 0 0 0 0 0 0
## 46 0 0 0 0 0 0 0 0
## 47 0 0 0 0 0 0 0 0
## 48 0 0 0 0 0 0 0 0
## 49 0 0 0 0 0 0 0 0
## 50 0 0 0 0 0 0 0 0
## 51 0 0 0 0 0 0 0 0
## 52 0 0 0 0 0 0 0 0
## 53 0 0 0 0 0 0 0 0
## 54 0 0 0 0 0 0 0 0
## 55 0 0 0 0 0 0 0 0
## 56 0 0 0 0 0 0 0 0
## 57 0 0 0 0 0 0 0 0
## 58 0 0 0 0 0 0 0 0
## 59 0 0 0 0 0 0 0 0
## 60 0 0 0 0 0 0 0 0
## 61 0 0 0 0 0 0 0 0
## 62 0 0 0 0 0 0 0 0
## 63 0 0 0 0 0 0 0 0
## 64 0 0 0 0 0 0 0 0
## 65 0 0 0 0 0 0 0 0
## 66 0 0 0 0 0 0 0 0
```

```
##   67          0          0        0     0         0      0      0       0
##   68          0          0        0     0         0      0      0       0
##   69          0          0        0     0         0      0      0       0
##   70          0          0        0     0         0      0      0       0
##   71          0          0        0     0         0      0      0       0
##   72          0          0        0     0         0      0      0       0
##   73          0          0        0     0         0      0      0       0
##   74          0          0        0     0         0      0      0       0
##   75          0          0        0     0         0      0      0       0
##   76          0          0        0     0         0      0      0       0
##   77          0          0        0     0         0      0      0       0
##   78          0          0        0     0         0      0      0       0
##   79          0          0        0     0         0      0      0       0
##   80          0          0        0     0         0      0      0       0
##   81          0          0        0     0         0      0      0       0
##   82          0          0        0     0         0      0      0       0
##   83          0          0        0     0         0      0      0       0
##   84          0          0        0     0         0      0      0       0
##   85          0          0        0     0         0      0      0       0
##   86          0          0        0     0         0      0      0       0
##   87          0          0        0     0         0      0      0       0
##   88          0          0        0     0         0      0      0       0
##   89          0          0        0     0         0      0      0       0
##   90          0          0        0     0         0      0      0       0
##   91          0          0        0     0         0      0      0       0
##   92          0          0        0     0         0      0      0       0
##   93          0          0        0     0         0      0      0       0
##   94          0          0        0     0         0      0      0       0
##   95          0          0        0     0         0      0      0       0
##   96          0          0        0     0         0      0      0       0
##   97          0          0        0     0         0      0      0       0
##   98          0          0        0     0         0      0      0       0
##   99          0          0        0     0         0      0      0       0
##   100         0          0        0     0         0      0      0       0
##
##       Mozambique Namibia Nepal Netherlands New Zealand Nigeria North Macedonia
##   1             0       0     0           0           0       0               0
##   2             0       0     0           0           0       0               0
##   3             0       0     0           0           0       0               0
##   4             0       0     0           0           0       0               0
##   5             0       0     0           0           0       0               0
##   6             0       0     0           0           0       0               0
##   7             0       0     0           0           0       0               0
##   8             0       0     0           0           0       0               0
##   9             0       0     0           0           0       0               0
##   10            0       0     0           0           0       0               0
##   11            0       0     0           0           0       0               0
##   12            0       0     0           0           0       0               0
##   13            0       0     0           0           0       0               0
##   14            0       0     0           0           0       0               0
##   15            0       0     0           0           0       0               0
##   16            0       0     0           0           0       0               0
##   17            0       0     0           0           0       0               0
##   18            0       0     0           0           0       0               0
```

```
## 19    0    0    0    0    0    0    0
## 20    0    0    0    0    0    0    0
## 21    0    0    0    0    0    0    0
## 22    0    0    0    0    0    0    0
## 23    0    0    0    0    0    0    0
## 24    0    0    0    0    0    0    0
## 25    0    0    0    0    0    0    0
## 26    0    0    0    0    0    0    0
## 27    0    0    0    0    0    0    0
## 28    0    0    0    0    0    0    0
## 29    0    0    0    0    0    0    0
## 30    0    0    0    0    0    0    0
## 31    0    0    0    0    0    0    0
## 32    0    0    0    0    0    0    0
## 33    0    0    0    0    0    0    0
## 34    0    0    0    0    0    0    0
## 35    0    0    0    0    0    0    0
## 36    0    0    0    0    0    0    0
## 37    0    0    0    0    0    0    0
## 38    0    0    0    0    0    0    0
## 39    0    0    0    0    0    0    0
## 40    0    0    0    0    0    0    0
## 41    0    0    0    0    0    0    0
## 42    0    0    0    0    0    0    0
## 43    0    0    0    0    0    0    0
## 44    0    0    0    0    0    0    0
## 45    0    0    0    0    0    0    0
## 46    0    0    0    0    0    0    0
## 47    0    0    0    0    0    0    0
## 48    0    0    0    0    0    0    0
## 49    0    0    0    0    0    0    0
## 50    0    0    0    0    0    0    0
## 51    0    0    0    0    0    0    0
## 52    0    0    0    0    0    0    0
## 53    0    0    0    0    0    0    0
## 54    0    0    0    0    0    0    0
## 55    0    0    0    0    0    0    0
## 56    0    0    0    0    0    0    0
## 57    0    0    0    0    0    0    0
## 58    0    0    0    0    0    0    0
## 59    0    0    0    0    0    0    0
## 60    0    0    0    0    0    0    0
## 61    0    0    0    0    0    0    0
## 62    0    0    0    0    0    0    0
## 63    0    0    0    0    0    0    0
## 64    0    0    0    0    0    0    0
## 65    0    0    0    0    0    0    0
## 66    0    0    0    0    0    0    0
## 67    0    0    0    0    0    0    0
## 68    0    0    0    0    0    0    0
## 69    0    0    0    0    0    0    0
## 70    0    0    0    0    0    0    0
## 71    0    0    0    0    0    0    0
## 72    0    0    0    0    0    0    0
```

```
## 73              0        0      0            0            0        0                0
## 74              0        0      0            0            0        0                0
## 75              0        0      0            0            0        0                0
## 76              0        0      0            0            0        0                0
## 77              0        0      0            0            0        0                0
## 78              0        0      0            0            0        0                0
## 79              0        0      0            0            0        0                0
## 80              0        0      0            0            0        0                0
## 81              0        0      0            0            0        0                0
## 82              0        0      0            0            0        0                0
## 83              0        0      0            0            0        0                0
## 84              0        0      0            0            0        0                0
## 85              0        0      0            0            0        0                0
## 86              0        0      0            0            0        0                0
## 87              0        0      0            0            0        0                0
## 88              0        0      0            0            0        0                0
## 89              0        0      0            0            0        0                0
## 90              0        0      0            0            0        0                0
## 91              0        0      0            0            0        0                0
## 92              0        0      0            0            0        0                0
## 93              0        0      0            0            0        0                0
## 94              0        0      0            0            0        0                0
## 95              0        0      0            0            0        0                0
## 96              0        0      0            0            0        0                0
## 97              0        0      0            0            0        0                0
## 98              0        0      0            0            0        0                0
## 99              0        0      0            0            0        0                0
## 100             0        0      0            0            0        0                0
##
##         Norway Oman Pakistan Panama Papua New Guinea Paraguay Peru Philippines
## 1            0    0        0      0                0        0    1           0
## 2            0    0        0      0                0        0    0           0
## 3            0    0        0      0                0        0    0           0
## 4            0    0        0      0                0        0    0           0
## 5            0    0        0      0                0        0    1           0
## 6            0    0        0      0                0        0    0           0
## 7            0    0        0      0                0        0    1           0
## 8            0    0        0      0                0        0    0           0
## 9            0    0        0      0                0        0    3           0
## 10           0    0        0      0                0        0    2           0
## 11           0    0        0      0                0        0    1           0
## 12           0    0        0      0                0        0    0           0
## 13           0    0        0      0                0        0    1           0
## 14           0    0        0      0                0        0    4           0
## 15           0    0        0      0                0        0    1           0
## 16           0    0        0      0                0        0    1           0
## 17           0    0        0      0                0        0    2           0
## 18           0    0        0      0                0        0    1           0
## 19           0    0        0      0                0        0    0           0
## 20           0    0        0      0                0        0    1           0
## 21           0    0        0      0                0        0    0           0
## 22           0    0        0      0                0        0    0           0
## 23           0    0        0      0                0        0    0           0
## 24           0    0        0      0                0        0    1           0
```

```
##    25      0   0     0    0          0     0    0       0
##    26      0   0     0    0          0     0    1       0
##    27      0   0     0    0          0     0    2       0
##    28      0   0     0    0          0     0    3       0
##    29      0   0     0    0          0     0    1       0
##    30      0   0     0    0          0     0    3       0
##    31      0   0     0    0          0     0    5       0
##    32      0   0     0    0          0     0    2       0
##    33      0   0     0    0          0     0    2       0
##    34      0   0     0    0          0     0    5       0
##    35      0   0     0    0          0     0    6       0
##    36      0   0     0    0          0     0    4       0
##    37      0   0     0    0          0     0    6       0
##    38      0   0     0    0          0     0    6       0
##    39      0   0     0    0          0     0    5       0
##    40      0   0     0    0          0     0    5       0
##    41      0   0     0    0          0     0    3       0
##    42      0   0     0    0          0     0    2       0
##    43      0   0     0    0          0     0    4       0
##    44      0   0     0    0          0     0    2       0
##    45      0   0     0    0          0     0    4       0
##    46      0   0     0    0          0     0    3       0
##    47      0   0     0    0          0     0    9       0
##    48      0   0     0    0          0     0   10       0
##    49      0   0     0    0          0     0   29       0
##    50      0   0     0    0          0     0  263       0
##    51      0   0     0    0          0     0   28       0
##    52      0   0     0    0          0     0   17       0
##    53      0   0     0    0          0     0   14       0
##    54      0   0     0    0          0     0   10       0
##    55      0   0     0    0          0     0    5       0
##    56      0   0     0    0          0     0    1       0
##    57      0   0     0    0          0     0    3       0
##    58      0   0     0    0          0     0    4       0
##    59      0   0     0    0          0     0    2       0
##    60      0   0     0    0          0     0    2       0
##    61      0   0     0    0          0     0    3       0
##    62      0   0     0    0          0     0    2       0
##    63      0   0     0    0          0     0    0       0
##    64      0   0     0    0          0     0    4       0
##    65      0   0     0    0          0     0    7       0
##    66      0   0     0    0          0     0    3       0
##    67      0   0     0    0          0     0    4       0
##    68      0   0     0    0          0     0    7       0
##    69      0   0     0    0          0     0    8       0
##    70      0   0     0    0          0     0    6       0
##    71      0   0     0    0          0     0    3       0
##    72      0   0     0    0          0     0    3       0
##    73      0   0     0    0          0     0    3       0
##    74      0   0     0    0          0     0    3       0
##    75      0   0     0    0          0     0    5       0
##    76      0   0     0    0          0     0    3       0
##    77      0   0     0    0          0     0    5       0
##    78      0   0     0    0          0     0    4       0
```

```
##   79       0    0        0      0                0        0    3           0
##   80       0    0        0      0                0        0    3           0
##   81       0    0        0      0                0        0    4           0
##   82       0    0        0      0                0        0    2           0
##   83       0    0        0      0                0        0    2           0
##   84       0    0        0      0                0        0    3           0
##   85       0    0        0      0                0        0    3           0
##   86       0    0        0      0                0        0    5           0
##   87       0    0        0      0                0        0    6           0
##   88       0    0        0      0                0        0    6           0
##   89       0    0        0      0                0        0    6           0
##   90       0    0        0      0                0        0   12           0
##   91       0    0        0      0                0        0    8           0
##   92       0    0        0      0                0        0    7           0
##   93       0    0        0      0                0        0    9           0
##   94       0    0        0      0                0        0   11           0
##   95       0    0        0      0                0        0    7           0
##   96       0    0        0      0                0        0    8           0
##   97       0    0        0      0                0        0    7           0
##   98       0    0        0      0                0        0    6           0
##   99       0    0        0      0                0        0    5           0
##   100      0    0        0      0                0        0   36           0
##
##       Poland Portugal Puerto Rico Qatar Romania Russia Saudi Arabia Senegal
##   1        0        0           0     0       0      0            0       0
##   2        0        0           0     0       0      0            0       0
##   3        0        0           0     0       0      0            0       0
##   4        0        0           0     0       0      0            0       0
##   5        0        0           0     0       0      0            0       0
##   6        0        0           0     0       0      0            0       0
##   7        0        0           0     0       0      0            0       0
##   8        0        0           0     0       0      0            0       0
##   9        0        0           0     0       0      0            0       0
##   10       0        0           0     0       0      0            0       0
##   11       0        0           0     0       0      0            0       0
##   12       0        0           0     0       0      0            0       0
##   13       0        0           0     0       0      0            0       0
##   14       0        0           0     0       0      0            0       0
##   15       0        0           0     0       0      0            0       0
##   16       0        0           0     0       0      0            0       0
##   17       0        0           0     0       0      0            0       0
##   18       0        0           0     0       0      0            0       0
##   19       0        0           0     0       0      0            0       0
##   20       0        0           0     0       0      0            0       0
##   21       0        0           0     0       0      0            0       0
##   22       0        0           0     0       0      0            0       0
##   23       0        0           0     0       0      0            0       0
##   24       0        0           0     0       0      0            0       0
##   25       0        0           0     0       0      0            0       0
##   26       0        0           0     0       0      0            0       0
##   27       0        0           0     0       0      0            0       0
##   28       0        0           0     0       0      0            0       0
##   29       0        0           0     0       0      0            0       0
##   30       0        0           0     0       0      0            0       0
```

```
## 31 0 0 0 0 0 0 0 0
## 32 0 0 0 0 0 0 0 0
## 33 0 0 0 0 0 0 0 0
## 34 0 0 0 0 0 0 0 0
## 35 0 0 0 0 0 0 0 0
## 36 0 0 0 0 0 0 0 0
## 37 0 0 0 0 0 0 0 0
## 38 0 0 0 0 0 0 0 0
## 39 0 0 0 0 0 0 0 0
## 40 0 0 0 0 0 0 0 0
## 41 0 0 0 0 0 0 0 0
## 42 0 0 0 0 0 0 0 0
## 43 0 0 0 0 0 0 0 0
## 44 0 0 0 0 0 0 0 0
## 45 0 0 0 0 0 0 0 0
## 46 0 0 0 0 0 0 0 0
## 47 0 0 0 0 0 0 0 0
## 48 0 0 0 0 0 0 0 0
## 49 0 0 0 0 0 0 0 0
## 50 0 0 0 0 0 0 0 0
## 51 0 0 0 0 0 0 0 0
## 52 0 0 0 0 0 0 0 0
## 53 0 0 0 0 0 0 0 0
## 54 0 0 0 0 0 0 0 0
## 55 0 0 0 0 0 0 0 0
## 56 0 0 0 0 0 0 0 0
## 57 0 0 0 0 0 0 0 0
## 58 0 0 0 0 0 0 0 0
## 59 0 0 0 0 0 0 0 0
## 60 0 0 0 0 0 0 0 0
## 61 0 0 0 0 0 0 0 0
## 62 0 0 0 0 0 0 0 0
## 63 0 0 0 0 0 0 0 0
## 64 0 0 0 0 0 0 0 0
## 65 0 0 0 0 0 0 0 0
## 66 0 0 0 0 0 0 0 0
## 67 0 0 0 0 0 0 0 0
## 68 0 0 0 0 0 0 0 0
## 69 0 0 0 0 0 0 0 0
## 70 0 0 0 0 0 0 0 0
## 71 0 0 0 0 0 0 0 0
## 72 0 0 0 0 0 0 0 0
## 73 0 0 0 0 0 0 0 0
## 74 0 0 0 0 0 0 0 0
## 75 0 0 0 0 0 0 0 0
## 76 0 0 0 0 0 0 0 0
## 77 0 0 0 0 0 0 0 0
## 78 0 0 0 0 0 0 0 0
## 79 0 0 0 0 0 0 0 0
## 80 0 0 0 0 0 0 0 0
## 81 0 0 0 0 0 0 0 0
## 82 0 0 0 0 0 0 0 0
## 83 0 0 0 0 0 0 0 0
## 84 0 0 0 0 0 0 0 0
```

```
##   85       0        0           0     0       0      0            0       0
##   86       0        0           0     0       0      0            0       0
##   87       0        0           0     0       0      0            0       0
##   88       0        0           0     0       0      0            0       0
##   89       0        0           0     0       0      0            0       0
##   90       0        0           0     0       0      0            0       0
##   91       0        0           0     0       0      0            0       0
##   92       0        0           0     0       0      0            0       0
##   93       0        0           0     0       0      0            0       0
##   94       0        0           0     0       0      0            0       0
##   95       0        0           0     0       0      0            0       0
##   96       0        0           0     0       0      0            0       0
##   97       0        0           0     0       0      0            0       0
##   98       0        0           0     0       0      0            0       0
##   99       0        0           0     0       0      0            0       0
##   100      0        0           0     0       0      0            0       0
## 
##        Serbia Singapore Slovakia Slovenia Somalia South Africa South Korea Spain
##   1         0         0        0        0       0            0           0     0
##   2         0         0        0        0       0            0           0     0
##   3         0         0        0        0       0            0           0     0
##   4         0         0        0        0       0            0           0     0
##   5         0         0        0        0       0            0           0     0
##   6         0         0        0        0       0            0           0     0
##   7         0         0        0        0       0            0           0     0
##   8         0         0        0        0       0            0           0     0
##   9         0         0        0        0       0            0           0     0
##   10        0         0        0        0       0            0           0     0
##   11        0         0        0        0       0            0           0     0
##   12        0         0        0        0       0            0           0     0
##   13        0         0        0        0       0            0           0     0
##   14        0         0        0        0       0            0           0     0
##   15        0         0        0        0       0            0           0     0
##   16        0         0        0        0       0            0           0     0
##   17        0         0        0        0       0            0           0     0
##   18        0         0        0        0       0            0           0     0
##   19        0         0        0        0       0            0           0     0
##   20        0         0        0        0       0            0           0     0
##   21        0         0        0        0       0            0           0     0
##   22        0         0        0        0       0            0           0     0
##   23        0         0        0        0       0            0           0     0
##   24        0         0        0        0       0            0           0     0
##   25        0         0        0        0       0            0           0     0
##   26        0         0        0        0       0            0           0     0
##   27        0         0        0        0       0            0           0     0
##   28        0         0        0        0       0            0           0     0
##   29        0         0        0        0       0            0           0     0
##   30        0         0        0        0       0            0           0     0
##   31        0         0        0        0       0            0           0     0
##   32        0         0        0        0       0            0           0     0
##   33        0         0        0        0       0            0           0     0
##   34        0         0        0        0       0            0           0     0
##   35        0         0        0        0       0            0           0     0
##   36        0         0        0        0       0            0           0     0
```

```
## 37 0 0 0 0 0 0 0 0
## 38 0 0 0 0 0 0 0 0
## 39 0 0 0 0 0 0 0 0
## 40 0 0 0 0 0 0 0 0
## 41 0 0 0 0 0 0 0 0
## 42 0 0 0 0 0 0 0 0
## 43 0 0 0 0 0 0 0 0
## 44 0 0 0 0 0 0 0 0
## 45 0 0 0 0 0 0 0 0
## 46 0 0 0 0 0 0 0 0
## 47 0 0 0 0 0 0 0 0
## 48 0 0 0 0 0 0 0 0
## 49 0 0 0 0 0 0 0 0
## 50 0 0 0 0 0 0 0 0
## 51 0 0 0 0 0 0 0 0
## 52 0 0 0 0 0 0 0 0
## 53 0 0 0 0 0 0 0 0
## 54 0 0 0 0 0 0 0 0
## 55 0 0 0 0 0 0 0 0
## 56 0 0 0 0 0 0 0 0
## 57 0 0 0 0 0 0 0 0
## 58 0 0 0 0 0 0 0 0
## 59 0 0 0 0 0 0 0 0
## 60 0 0 0 0 0 0 0 0
## 61 0 0 0 0 0 0 0 0
## 62 0 0 0 0 0 0 0 0
## 63 0 0 0 0 0 0 0 0
## 64 0 0 0 0 0 0 0 0
## 65 0 0 0 0 0 0 0 0
## 66 0 0 0 0 0 0 0 0
## 67 0 0 0 0 0 0 0 0
## 68 0 0 0 0 0 0 0 0
## 69 0 0 0 0 0 0 0 0
## 70 0 0 0 0 0 0 0 0
## 71 0 0 0 0 0 0 0 0
## 72 0 0 0 0 0 0 0 0
## 73 0 0 0 0 0 0 0 0
## 74 0 0 0 0 0 0 0 0
## 75 0 0 0 0 0 0 0 0
## 76 0 0 0 0 0 0 0 0
## 77 0 0 0 0 0 0 0 0
## 78 0 0 0 0 0 0 0 0
## 79 0 0 0 0 0 0 0 0
## 80 0 0 0 0 0 0 0 0
## 81 0 0 0 0 0 0 0 0
## 82 0 0 0 0 0 0 0 0
## 83 0 0 0 0 0 0 0 0
## 84 0 0 0 0 0 0 0 0
## 85 0 0 0 0 0 0 0 0
## 86 0 0 0 0 0 0 0 0
## 87 0 0 0 0 0 0 0 0
## 88 0 0 0 0 0 0 0 0
## 89 0 0 0 0 0 0 0 0
## 90 0 0 0 0 0 0 0 0
```

```
## 91        0          0        0        0       0            0           0      0
## 92        0          0        0        0       0            0           0      0
## 93        0          0        0        0       0            0           0      0
## 94        0          0        0        0       0            0           0      0
## 95        0          0        0        0       0            0           0      0
## 96        0          0        0        0       0            0           0      0
## 97        0          0        0        0       0            0           0      0
## 98        0          0        0        0       0            0           0      0
## 99        0          0        0        0       0            0           0      0
## 100       0          0        0        0       0            0           0      0
##
##     Sri Lanka Sudan Suriname Sweden Switzerland Syria Taiwan Thailand
## 1           0     0        0      2           0     0      0        0
## 2           0     0        0      0           0     0      0        0
## 3           0     0        0      0           0     0      0        0
## 4           0     0        0      0           0     0      0        0
## 5           0     0        0      0           0     0      0        0
## 6           0     0        0      0           0     0      0        0
## 7           0     0        0      0           0     0      0        0
## 8           0     0        0      0           0     0      0        0
## 9           0     0        0      0           0     0      0        0
## 10          0     0        0      0           0     0      0        0
## 11          0     0        0      0           0     0      0        0
## 12          0     0        0      0           0     0      0        0
## 13          0     0        0      0           0     0      0        0
## 14          0     0        0      0           0     0      0        0
## 15          0     0        0      1           0     0      0        0
## 16          0     0        0      0           0     0      0        0
## 17          0     0        0      1           0     0      0        0
## 18          0     0        0      0           0     0      0        0
## 19          0     0        0      0           0     0      0        0
## 20          0     0        0      0           0     0      0        0
## 21          0     0        0      0           0     0      0        0
## 22          0     0        0      0           0     0      0        0
## 23          0     0        0      0           0     0      0        0
## 24          0     0        0      0           0     0      0        0
## 25          0     0        0      0           0     0      0        0
## 26          0     0        0      0           0     0      0        0
## 27          0     0        0      0           0     0      0        0
## 28          0     0        0      2           0     0      0        0
## 29          0     0        0      1           0     0      0        0
## 30          0     0        0      0           0     0      0        0
## 31          0     0        0      0           0     0      0        0
## 32          0     0        0      0           0     0      0        0
## 33          0     0        0      2           0     0      0        0
## 34          0     0        0      1           0     0      0        0
## 35          0     0        0      1           0     0      0        0
## 36          0     0        0      2           0     0      0        0
## 37          0     0        0      1           0     0      0        0
## 38          0     0        0      0           0     0      0        0
## 39          0     0        0      1           0     0      0        0
## 40          0     0        0      1           0     0      0        0
## 41          0     0        0      2           0     0      0        0
## 42          0     0        0      0           0     0      0        0
```

```
##   43          0     0       0    1         0     0     0       0
##   44          0     0       0    1         0     0     0       0
##   45          0     0       0    4         0     0     0       0
##   46          0     0       0    1         0     0     0       0
##   47          0     0       0    2         0     0     0       0
##   48          0     0       0    2         0     0     0       0
##   49          0     0       0    2         0     0     0       0
##   50          0     0       0   20         0     0     0       0
##   51          0     0       0    2         0     0     0       0
##   52          0     0       0    0         0     0     0       0
##   53          0     0       0    2         0     0     0       0
##   54          0     0       0    0         0     0     0       0
##   55          0     0       0    0         0     0     0       0
##   56          0     0       0    0         0     0     0       0
##   57          0     0       0    0         0     0     0       0
##   58          0     0       0    1         0     0     0       0
##   59          0     0       0    1         0     0     0       0
##   60          0     0       0    0         0     0     0       0
##   61          0     0       0    4         0     0     0       0
##   62          0     0       0    1         0     0     0       0
##   63          0     0       0    1         0     0     0       0
##   64          0     0       0    1         0     0     0       0
##   65          0     0       0    1         0     0     0       0
##   66          0     0       0    0         0     0     0       0
##   67          0     0       0    0         0     0     0       0
##   68          0     0       0    1         0     0     0       0
##   69          0     0       0    2         0     0     0       0
##   70          0     0       0    4         0     0     0       0
##   71          0     0       0    4         0     0     0       0
##   72          0     0       0    1         0     0     0       0
##   73          0     0       0    1         0     0     0       0
##   74          0     0       0    1         0     0     0       0
##   75          0     0       0    1         0     0     0       0
##   76          0     0       0    2         0     0     0       0
##   77          0     0       0    3         0     0     0       0
##   78          0     0       0    3         0     0     0       0
##   79          0     0       0    4         0     0     0       0
##   80          0     0       0    0         0     0     0       0
##   81          0     0       0    3         0     0     0       0
##   82          0     0       0    0         0     0     0       0
##   83          0     0       0    0         0     0     0       0
##   84          0     0       0    0         0     0     0       0
##   85          0     0       0    2         0     0     0       0
##   86          0     0       0    1         0     0     0       0
##   87          0     0       0    0         0     0     0       0
##   88          0     0       0    1         0     0     0       0
##   89          0     0       0    2         0     0     0       0
##   90          0     0       0    1         0     0     0       0
##   91          0     0       0    1         0     0     0       0
##   92          0     0       0    1         0     0     0       0
##   93          0     0       0    0         0     0     0       0
##   94          0     0       0    2         0     0     0       0
##   95          0     0       0    2         0     0     0       0
##   96          0     0       0    1         0     0     0       0
```

```
##   97          0     0        0      0           0     0      0        0
##   98          0     0        0      3           0     0      0        0
##   99          0     0        0      2           0     0      0        0
##   100         0     0        0     24           0     0      0        0
##
##       Trinidad and Tobago Tunisia Turkey UAE Uganda  UK Ukraine Uruguay USA
##   1                     0       0      3   0      0   2       0       0   2
##   2                     0       0      0   0      0   0       0       0   2
##   3                     0       0      0   0      0   0       0       0   0
##   4                     0       0      1   0      0   1       0       0   0
##   5                     0       0      0   0      0   0       0       0   0
##   6                     0       0      0   0      0   0       0       0   0
##   7                     0       0      0   0      0   0       0       0   0
##   8                     0       0      1   0      0   0       0       0   0
##   9                     0       0      0   0      0   0       0       0   0
##   10                    0       0      0   0      0   0       0       0   1
##   11                    0       0      0   0      0   0       0       0   0
##   12                    0       0      0   0      0   0       0       0   1
##   13                    0       0      0   0      0   0       0       0   1
##   14                    0       0      0   0      0   0       0       0   0
##   15                    0       0      0   0      0   0       0       0   0
##   16                    0       0      1   0      0   0       0       0   1
##   17                    0       0      0   0      0   0       0       0   0
##   18                    0       0      1   0      0   0       0       0   2
##   19                    0       0      1   0      0   2       0       0   0
##   20                    0       0      0   0      0   1       0       0   2
##   21                    0       0      0   0      0   0       0       0   1
##   22                    0       0      1   0      0   0       0       0   0
##   23                    0       0      1   0      0   0       0       0   1
##   24                    0       0      1   0      0   0       0       0   1
##   25                    0       0      2   0      0   1       0       0   3
##   26                    0       0      2   0      0   1       0       0   6
##   27                    0       0      0   0      0   1       0       0   6
##   28                    0       0      6   0      0   6       0       0   1
##   29                    0       0      6   0      0   5       0       0   2
##   30                    0       0      3   0      0   2       0       0   5
##   31                    0       0      5   0      0   3       0       0   4
##   32                    0       0      3   0      0   5       0       0   1
##   33                    0       0      5   0      0   1       0       0   3
##   34                    0       0      4   0      0   2       0       0   5
##   35                    0       0      4   0      0  11       0       0   4
##   36                    0       0      8   0      0  11       0       0   2
##   37                    0       0      8   0      0  16       0       0   8
##   38                    0       0      6   0      0  13       0       0   7
##   39                    0       0     11   0      0  15       0       0   9
##   40                    0       0     11   0      0  17       0       0   8
##   41                    0       0      6   0      0  19       0       0   6
##   42                    0       0      8   0      0  24       0       0   7
##   43                    0       0      6   0      0  28       0       0   7
##   44                    0       0      6   0      0  30       0       0   7
##   45                    0       0      4   0      0  24       0       0   8
##   46                    0       0     10   0      0  20       0       0   1
##   47                    0       0     16   0      0  24       0       0   6
##   48                    0       0     14   0      0  27       0       0   6
```

```
##   49                    0       0    38   0      0  43       0       0  11
##   50                    0       0   166   0      0 374       0       0  69
##   51                    0       0    36   0      0  74       0       0  13
##   52                    0       0    17   0      0  49       0       0   8
##   53                    0       0    14   0      0  32       0       0   3
##   54                    0       0     9   0      0  34       0       0   4
##   55                    0       0     9   0      0  26       0       0   1
##   56                    0       0     6   0      0  16       0       0   8
##   57                    0       0     5   0      0  21       0       0   4
##   58                    0       0    12   0      0  20       0       0   6
##   59                    0       0    11   0      0  28       0       0   5
##   60                    0       0     7   0      0  25       0       0   4
##   61                    0       0     5   0      0  20       0       0   4
##   62                    0       0    12   0      0  25       0       0   5
##   63                    0       0    15   0      0  19       0       0   7
##   64                    0       0    16   0      0  23       0       0   6
##   65                    0       0    11   0      0  30       0       0   2
##   66                    0       0    15   0      0  18       0       0   5
##   67                    0       0     7   0      0  30       0       0   5
##   68                    0       0    12   0      0  24       0       0   5
##   69                    0       0    15   0      0  34       0       0   5
##   70                    0       0    12   0      0  26       0       0  10
##   71                    0       0    20   0      0  34       0       0   8
##   72                    0       0    22   0      0  23       0       0   9
##   73                    0       0    17   0      0  21       0       0   5
##   74                    0       0    12   0      0  29       0       0   2
##   75                    0       0    15   0      0  23       0       0   9
##   76                    0       0    10   0      0  25       0       0   6
##   77                    0       0    14   0      0  19       0       0   8
##   78                    0       0     9   0      0  20       0       0   6
##   79                    0       0    10   0      0  19       0       0   4
##   80                    0       0    12   0      0  12       0       0   9
##   81                    0       0     7   0      0  17       0       0   6
##   82                    0       0     8   0      0  14       0       0   2
##   83                    0       0     5   0      0   8       0       0   5
##   84                    0       0     9   0      0   9       0       0   4
##   85                    0       0     7   0      0  12       0       0   6
##   86                    0       0     8   0      0  15       0       0   4
##   87                    0       0    10   0      0   9       0       0   5
##   88                    0       0     9   0      0  13       0       0   5
##   89                    0       0     6   0      0  14       0       0   5
##   90                    0       0    13   0      0  13       0       0   7
##   91                    0       0    13   0      0  14       0       0   5
##   92                    0       0     9   0      0   7       0       0   3
##   93                    0       0    12   0      0  19       0       0   1
##   94                    0       0    12   0      0   5       0       0   8
##   95                    0       0    12   0      0  11       0       0   4
##   96                    0       0    11   0      0  14       0       0   3
##   97                    0       0    11   0      0   9       0       0   5
##   98                    0       0    13   0      0  13       0       0   5
##   99                    0       0    19   0      0  20       0       0   5
##   100                   0       0   198   0      0 173       0       0  62
##
##       Vietnam Zambia
```

```
## 1 0 0
## 2 0 0
## 3 0 0
## 4 0 0
## 5 0 0
## 6 0 0
## 7 0 0
## 8 0 0
## 9 0 0
## 10 0 0
## 11 0 0
## 12 0 0
## 13 0 0
## 14 0 0
## 15 0 0
## 16 0 0
## 17 0 0
## 18 0 0
## 19 0 0
## 20 0 0
## 21 0 0
## 22 0 0
## 23 0 0
## 24 0 0
## 25 0 0
## 26 0 0
## 27 0 0
## 28 0 0
## 29 0 0
## 30 0 0
## 31 0 0
## 32 0 0
## 33 0 0
## 34 0 0
## 35 0 0
## 36 0 0
## 37 0 0
## 38 0 0
## 39 0 0
## 40 0 0
## 41 0 0
## 42 0 0
## 43 0 0
## 44 0 0
## 45 0 0
## 46 0 0
## 47 0 0
## 48 0 0
## 49 0 0
## 50 0 0
## 51 0 0
## 52 0 0
## 53 0 0
## 54 0 0
```

```
##   55         0       0
##   56         0       0
##   57         0       0
##   58         0       0
##   59         0       0
##   60         0       0
##   61         0       0
##   62         0       0
##   63         0       0
##   64         0       0
##   65         0       0
##   66         0       0
##   67         0       0
##   68         0       0
##   69         0       0
##   70         0       0
##   71         0       0
##   72         0       0
##   73         0       0
##   74         0       0
##   75         0       0
##   76         0       0
##   77         0       0
##   78         0       0
##   79         0       0
##   80         0       0
##   81         0       0
##   82         0       0
##   83         0       0
##   84         0       0
##   85         0       0
##   86         0       0
##   87         0       0
##   88         0       0
##   89         0       0
##   90         0       0
##   91         0       0
##   92         0       0
##   93         0       0
##   94         0       0
##   95         0       0
##   96         0       0
##   97         0       0
##   98         0       0
##   99         0       0
##   100        0       0
```

```
# Wellbeing in N>100 countries
table(DatasetB_N100$wellbeing, DatasetB_N100$country_now_Name)
```

```
##
##                Afghanistan Albania Algeria Argentina Armenia Australia Austria
##   7        0             0       0       0         0       0         1       0
##   9.51     0             0       0       0         0       0         0       0
```

```
##   11.25   0           0       0       0         0       0         1       0
##   12.4    0           0       0       0         0       0         0       0
##   13.33   0           0       0       0         0       0         0       0
##   14.08   0           0       0       0         0       0         0       0
##   14.75   0           0       0       0         0       0         0       0
##   15.32   0           0       0       0         0       0         1       0
##   15.84   0           0       0       0         0       0         2       0
##   16.36   0           0       0       0         0       0         5       0
##   16.88   0           0       0       0         0       0         2       0
##   17.43   0           0       0       0         0       0         8       0
##   17.98   0           0       0       0         0       0        10       0
##   18.59   0           0       0       0         0       0         8       0
##   19.25   0           0       0       0         0       0        18       0
##   19.98   0           0       0       0         0       0         4       0
##   20.73   0           0       0       0         0       0         5       0
##   21.54   0           0       0       0         0       0         8       0
##   22.35   0           0       0       0         0       0        11       0
##   23.21   0           0       0       0         0       0        13       0
##   24.11   0           0       0       0         0       0        18       0
##   25.03   0           0       0       0         0       0         4       0
##   26.02   0           0       0       0         0       0         2       0
##   27.03   0           0       0       0         0       0         4       0
##   28.13   0           0       0       0         0       0         4       0
##   29.31   0           0       0       0         0       0         2       0
##   30.7    0           0       0       0         0       0         0       0
##   32.55   0           0       0       0         0       0         1       0
##   35      0           0       0       0         0       0         3       0
##
##         Azerbaijan Bangladesh Belgium Belize Bermuda Bolivia
##   7              0          1       0      0       0       0
##   9.51           0          0       0      0       0       0
##   11.25          0          1       0      0       0       0
##   12.4           0          1       0      0       0       0
##   13.33          0          2       0      0       0       0
##   14.08          0          4       0      0       0       0
##   14.75          0          1       0      0       0       0
##   15.32          0          1       0      0       0       0
##   15.84          0          5       0      0       0       0
##   16.36          0          8       0      0       0       0
##   16.88          0          3       0      0       0       0
##   17.43          0          6       0      0       0       0
##   17.98          0          8       0      0       0       0
##   18.59          0         13       0      0       0       0
##   19.25          0         11       0      0       0       0
##   19.98          0         10       0      0       0       0
##   20.73          0         17       0      0       0       0
##   21.54          0         20       0      0       0       0
##   22.35          0         18       0      0       0       0
##   23.21          0         11       0      0       0       0
##   24.11          0         15       0      0       0       0
##   25.03          0         29       0      0       0       0
##   26.02          0         26       0      0       0       0
##   27.03          0         21       0      0       0       0
##   28.13          0         14       0      0       0       0
```

```
##   29.31          0           7       0      0       0       0
##   30.7           0          11       0      0       0       0
##   32.55          0           3       0      0       0       0
##   35             0           8       0      0       0       0
##        
##         Bosnia and Herzegovina Brasil Bulgaria Canada Chile China Colombia
##   7                          0      0        0      1     0     0        0
##   9.51                       0      0        0      0     0     0        0
##   11.25                      0      0        0      1     0     0        0
##   12.4                       0      0        0      1     0     0        0
##   13.33                      0      0        0      0     0     0        0
##   14.08                      0      0        0      2     0     0        0
##   14.75                      0      0        0      0     0     0        0
##   15.32                      0      0        0      3     0     0        0
##   15.84                      0      0        0      3     0     0        0
##   16.36                      0      0        0      5     0     0        0
##   16.88                      0      0        0      6     0     0        0
##   17.43                      0      0        0      5     0     0        0
##   17.98                      0      0        0     12     0     0        0
##   18.59                      0      0        0      6     0     0        0
##   19.25                      0      0        0     12     0     0        0
##   19.98                      0      0        0     10     0     0        0
##   20.73                      0      0        0      5     0     0        0
##   21.54                      0      0        0     12     0     0        0
##   22.35                      0      0        0      7     0     0        0
##   23.21                      0      0        0      1     0     0        0
##   24.11                      0      0        0      4     0     0        0
##   25.03                      0      0        0      5     0     0        0
##   26.02                      0      0        0      1     0     0        0
##   27.03                      0      0        0      1     0     0        0
##   28.13                      0      0        0      1     0     0        0
##   29.31                      0      0        0      1     0     0        0
##   30.7                       0      0        0      0     0     0        0
##   32.55                      0      0        0      0     0     0        0
##   35                         0      0        0      0     0     0        0
##        
##         Costa Rica Croatia Czech Republic Denmark Ecuador Egypt Estonia Fiji
##   7              0       0              0       0       0     0       0    0
##   9.51           0       0              0       0       0     0       0    0
##   11.25          0       0              0       0       0     0       0    0
##   12.4           0       0              0       0       0     0       0    0
##   13.33          0       0              0       0       0     0       0    0
##   14.08          0       0              0       0       0     0       0    0
##   14.75          0       0              0       0       0     0       0    0
##   15.32          0       0              0       0       0     0       0    0
##   15.84          0       0              0       0       0     0       0    0
##   16.36          0       0              0       0       0     0       0    0
##   16.88          0       0              0       0       0     0       0    0
##   17.43          0       0              0       0       0     0       0    0
##   17.98          0       0              0       0       0     0       0    0
##   18.59          0       0              0       0       0     0       0    0
##   19.25          0       0              0       0       0     0       0    0
##   19.98          0       0              0       0       0     0       0    0
##   20.73          0       0              0       0       0     0       0    0
```

```
##   21.54          0       0             0       0       0     0       0    0
##   22.35          0       0             0       0       0     0       0    0
##   23.21          0       0             0       0       0     0       0    0
##   24.11          0       0             0       0       0     0       0    0
##   25.03          0       0             0       0       0     0       0    0
##   26.02          0       0             0       0       0     0       0    0
##   27.03          0       0             0       0       0     0       0    0
##   28.13          0       0             0       0       0     0       0    0
##   29.31          0       0             0       0       0     0       0    0
##   30.7           0       0             0       0       0     0       0    0
##   32.55          0       0             0       0       0     0       0    0
##   35             0       0             0       0       0     0       0    0
##
##         Finland France Georgia Germany Ghana Greece Guatemala Guernsey Guinea
##   7           0      0       0       0     0      0         0        0      0
##   9.51        0      0       0       0     0      0         0        0      0
##   11.25       0      1       0       1     0      0         0        0      0
##   12.4        0      0       0       0     0      0         0        0      0
##   13.33       0      0       0       1     0      0         0        0      0
##   14.08       0      1       0       2     0      0         0        0      0
##   14.75       0      0       0       4     0      0         0        0      0
##   15.32       0      3       0       1     0      0         0        0      0
##   15.84       0      0       0       4     0      0         0        0      0
##   16.36       0      5       0       4     0      0         0        0      0
##   16.88       0     10       0       9     0      0         0        0      0
##   17.43       0     15       0       7     0      0         0        0      0
##   17.98       0     18       0      10     0      0         0        0      0
##   18.59       0     19       0      17     0      0         0        0      0
##   19.25       0     20       0      19     0      0         0        0      0
##   19.98       0     23       0      12     0      0         0        0      0
##   20.73       0     36       0      12     0      0         0        0      0
##   21.54       0     39       0      19     0      0         0        0      0
##   22.35       0     32       0      22     0      0         0        0      0
##   23.21       0     32       0      21     0      0         0        0      0
##   24.11       0     31       0      19     0      0         0        0      0
##   25.03       0     18       0      16     0      0         0        0      0
##   26.02       0     14       0       9     0      0         0        0      0
##   27.03       0     17       0       3     0      0         0        0      0
##   28.13       0      4       0       3     0      0         0        0      0
##   29.31       0      2       0       3     0      0         0        0      0
##   30.7        0      2       0       2     0      0         0        0      0
##   32.55       0      1       0       0     0      0         0        0      0
##   35          0      1       0       1     0      0         0        0      0
##
##         Guyana Hong Kong Hungary Iceland India Indonesia Iran Iraq Ireland
##   7          0         0       0       0     0         0    0    0       0
##   9.51       0         0       0       0     0         0    0    0       0
##   11.25      0         0       0       0     0         0    0    0       0
##   12.4       0         0       0       0     0         0    0    0       0
##   13.33      0         0       0       0     0         0    0    0       0
##   14.08      0         0       0       0     0         0    0    0       0
##   14.75      0         0       0       0     0         0    0    0       0
##   15.32      0         0       0       0     0         0    0    0       0
##   15.84      0         0       0       0     0         0    0    0       0
```

```
##   16.36      0         0       0       0     0         0    0    0       0
##   16.88      0         0       0       0     0         0    0    0       0
##   17.43      0         0       0       0     0         0    0    0       0
##   17.98      0         0       0       0     0         0    0    0       0
##   18.59      0         0       0       0     0         0    0    0       0
##   19.25      0         0       0       0     0         0    0    0       0
##   19.98      0         0       0       0     0         0    0    0       0
##   20.73      0         0       0       0     0         0    0    0       0
##   21.54      0         0       0       0     0         0    0    0       0
##   22.35      0         0       0       0     0         0    0    0       0
##   23.21      0         0       0       0     0         0    0    0       0
##   24.11      0         0       0       0     0         0    0    0       0
##   25.03      0         0       0       0     0         0    0    0       0
##   26.02      0         0       0       0     0         0    0    0       0
##   27.03      0         0       0       0     0         0    0    0       0
##   28.13      0         0       0       0     0         0    0    0       0
##   29.31      0         0       0       0     0         0    0    0       0
##   30.7       0         0       0       0     0         0    0    0       0
##   32.55      0         0       0       0     0         0    0    0       0
##   35         0         0       0       0     0         0    0    0       0
##
##         Israel Italy Japan Jersey Jordan Kazakhstan Kenya Kuwait Kyrgyzstan
##   7          0     1     0      0      0          0     0      0          0
##   9.51       0     0     0      0      0          0     0      0          0
##   11.25      0     0     0      0      0          0     0      0          0
##   12.4       0     1     0      0      0          0     0      0          0
##   13.33      0     0     0      0      0          0     0      0          0
##   14.08      0     1     0      0      0          0     0      0          0
##   14.75      0     2     0      0      0          0     0      0          0
##   15.32      0     2     0      0      0          0     0      0          0
##   15.84      0     4     0      0      0          0     0      0          0
##   16.36      0     2     0      0      0          0     0      0          0
##   16.88      0     5     0      0      0          0     0      0          0
##   17.43      0     8     0      0      0          0     0      0          0
##   17.98      0     4     0      0      0          0     0      0          0
##   18.59      0     6     0      0      0          0     0      0          0
##   19.25      0     7     0      0      0          0     0      0          0
##   19.98      0    10     0      0      0          0     0      0          0
##   20.73      0     6     0      0      0          0     0      0          0
##   21.54      0     8     0      0      0          0     0      0          0
##   22.35      0    13     0      0      0          0     0      0          0
##   23.21      0     7     0      0      0          0     0      0          0
##   24.11      0     7     0      0      0          0     0      0          0
##   25.03      0     8     0      0      0          0     0      0          0
##   26.02      0     7     0      0      0          0     0      0          0
##   27.03      0     1     0      0      0          0     0      0          0
##   28.13      0     1     0      0      0          0     0      0          0
##   29.31      0     0     0      0      0          0     0      0          0
##   30.7       0     1     0      0      0          0     0      0          0
##   32.55      0     0     0      0      0          0     0      0          0
##   35         0     0     0      0      0          0     0      0          0
##
##         Lebanon Lithuania Luxembourg Malaysia Malta Mauritius Mexico Monaco
##   7           0         0          0        0     0         0      0      0
```

```
##   9.51         0         0          0        0     0          0      0       0
##   11.25        0         0          0        0     0          0      0       0
##   12.4         0         0          0        0     0          0      0       0
##   13.33        0         0          0        0     0          0      0       0
##   14.08        0         0          0        0     0          0      0       0
##   14.75        0         0          0        0     0          0      0       0
##   15.32        0         0          0        0     0          0      0       0
##   15.84        0         0          0        0     0          0      0       0
##   16.36        0         0          0        0     0          0      0       0
##   16.88        0         0          0        0     0          0      0       0
##   17.43        0         0          0        0     0          0      0       0
##   17.98        0         0          0        0     0          0      0       0
##   18.59        0         0          0        0     0          0      0       0
##   19.25        0         0          0        0     0          0      0       0
##   19.98        0         0          0        0     0          0      0       0
##   20.73        0         0          0        0     0          0      0       0
##   21.54        0         0          0        0     0          0      0       0
##   22.35        0         0          0        0     0          0      0       0
##   23.21        0         0          0        0     0          0      0       0
##   24.11        0         0          0        0     0          0      0       0
##   25.03        0         0          0        0     0          0      0       0
##   26.02        0         0          0        0     0          0      0       0
##   27.03        0         0          0        0     0          0      0       0
##   28.13        0         0          0        0     0          0      0       0
##   29.31        0         0          0        0     0          0      0       0
##   30.7         0         0          0        0     0          0      0       0
##   32.55        0         0          0        0     0          0      0       0
##   35           0         0          0        0     0          0      0       0
##
##         Morocco Mozambique Namibia Nepal Netherlands New Zealand Nigeria
##   7           0          0       0     0           0           0       0
##   9.51        0          0       0     0           0           0       0
##   11.25       0          0       0     0           0           0       0
##   12.4        0          0       0     0           0           0       0
##   13.33       0          0       0     0           0           0       0
##   14.08       0          0       0     0           0           0       0
##   14.75       0          0       0     0           0           0       0
##   15.32       0          0       0     0           0           0       0
##   15.84       0          0       0     0           0           0       0
##   16.36       0          0       0     0           0           0       0
##   16.88       0          0       0     0           0           0       0
##   17.43       0          0       0     0           0           0       0
##   17.98       0          0       0     0           0           0       0
##   18.59       0          0       0     0           0           0       0
##   19.25       0          0       0     0           0           0       0
##   19.98       0          0       0     0           0           0       0
##   20.73       0          0       0     0           0           0       0
##   21.54       0          0       0     0           0           0       0
##   22.35       0          0       0     0           0           0       0
##   23.21       0          0       0     0           0           0       0
##   24.11       0          0       0     0           0           0       0
##   25.03       0          0       0     0           0           0       0
##   26.02       0          0       0     0           0           0       0
##   27.03       0          0       0     0           0           0       0
```

```
##   28.13       0          0       0     0           0           0       0
##   29.31       0          0       0     0           0           0       0
##   30.7        0          0       0     0           0           0       0
##   32.55       0          0       0     0           0           0       0
##   35          0          0       0     0           0           0       0
##
##         North Macedonia Norway Oman Pakistan Panama Papua New Guinea Paraguay
##   7                   0      0    0        0      0                0        0
##   9.51                0      0    0        0      0                0        0
##   11.25               0      0    0        0      0                0        0
##   12.4                0      0    0        0      0                0        0
##   13.33               0      0    0        0      0                0        0
##   14.08               0      0    0        0      0                0        0
##   14.75               0      0    0        0      0                0        0
##   15.32               0      0    0        0      0                0        0
##   15.84               0      0    0        0      0                0        0
##   16.36               0      0    0        0      0                0        0
##   16.88               0      0    0        0      0                0        0
##   17.43               0      0    0        0      0                0        0
##   17.98               0      0    0        0      0                0        0
##   18.59               0      0    0        0      0                0        0
##   19.25               0      0    0        0      0                0        0
##   19.98               0      0    0        0      0                0        0
##   20.73               0      0    0        0      0                0        0
##   21.54               0      0    0        0      0                0        0
##   22.35               0      0    0        0      0                0        0
##   23.21               0      0    0        0      0                0        0
##   24.11               0      0    0        0      0                0        0
##   25.03               0      0    0        0      0                0        0
##   26.02               0      0    0        0      0                0        0
##   27.03               0      0    0        0      0                0        0
##   28.13               0      0    0        0      0                0        0
##   29.31               0      0    0        0      0                0        0
##   30.7                0      0    0        0      0                0        0
##   32.55               0      0    0        0      0                0        0
##   35                  0      0    0        0      0                0        0
##
##         Peru Philippines Poland Portugal Puerto Rico Qatar Romania Russia
##   7        1           0      0        0           0     0       0      0
##   9.51     0           0      0        0           0     0       0      0
##   11.25    0           0      0        0           0     0       0      0
##   12.4     0           0      0        0           0     0       0      0
##   13.33    0           0      0        0           0     0       0      0
##   14.08    2           0      0        0           0     0       0      0
##   14.75    2           0      0        0           0     0       0      0
##   15.32    4           0      0        0           0     0       0      0
##   15.84    9           0      0        0           0     0       0      0
##   16.36    9           0      0        0           0     0       0      0
##   16.88    8           0      0        0           0     0       0      0
##   17.43   19           0      0        0           0     0       0      0
##   17.98   25           0      0        0           0     0       0      0
##   18.59   42           0      0        0           0     0       0      0
##   19.25   55           0      0        0           0     0       0      0
##   19.98   30           0      0        0           0     0       0      0
```

```
##   20.73   42           0      0        0           0     0       0      0
##   21.54   46           0      0        0           0     0       0      0
##   22.35   49           0      0        0           0     0       0      0
##   23.21   55           0      0        0           0     0       0      0
##   24.11   54           0      0        0           0     0       0      0
##   25.03   49           0      0        0           0     0       0      0
##   26.02   49           0      0        0           0     0       0      0
##   27.03   42           0      0        0           0     0       0      0
##   28.13   33           0      0        0           0     0       0      0
##   29.31   30           0      0        0           0     0       0      0
##   30.7    21           0      0        0           0     0       0      0
##   32.55   20           0      0        0           0     0       0      0
##   35      28           0      0        0           0     0       0      0
##
##         Saudi Arabia Senegal Serbia Singapore Slovakia Slovenia Somalia
##   7                0       0      0         0        0        0       0
##   9.51             0       0      0         0        0        0       0
##   11.25            0       0      0         0        0        0       0
##   12.4             0       0      0         0        0        0       0
##   13.33            0       0      0         0        0        0       0
##   14.08            0       0      0         0        0        0       0
##   14.75            0       0      0         0        0        0       0
##   15.32            0       0      0         0        0        0       0
##   15.84            0       0      0         0        0        0       0
##   16.36            0       0      0         0        0        0       0
##   16.88            0       0      0         0        0        0       0
##   17.43            0       0      0         0        0        0       0
##   17.98            0       0      0         0        0        0       0
##   18.59            0       0      0         0        0        0       0
##   19.25            0       0      0         0        0        0       0
##   19.98            0       0      0         0        0        0       0
##   20.73            0       0      0         0        0        0       0
##   21.54            0       0      0         0        0        0       0
##   22.35            0       0      0         0        0        0       0
##   23.21            0       0      0         0        0        0       0
##   24.11            0       0      0         0        0        0       0
##   25.03            0       0      0         0        0        0       0
##   26.02            0       0      0         0        0        0       0
##   27.03            0       0      0         0        0        0       0
##   28.13            0       0      0         0        0        0       0
##   29.31            0       0      0         0        0        0       0
##   30.7             0       0      0         0        0        0       0
##   32.55            0       0      0         0        0        0       0
##   35               0       0      0         0        0        0       0
##
##         South Africa South Korea Spain Sri Lanka Sudan Suriname Sweden
##   7                0           0     0         0     0        0      0
##   9.51             0           0     0         0     0        0      0
##   11.25            0           0     0         0     0        0      1
##   12.4             0           0     0         0     0        0      0
##   13.33            0           0     0         0     0        0      0
##   14.08            0           0     0         0     0        0      2
##   14.75            0           0     0         0     0        0      3
##   15.32            0           0     0         0     0        0      4
```

```
##    15.84             0           0     0         0     0        0      3
##    16.36             0           0     0         0     0        0      5
##    16.88             0           0     0         0     0        0      7
##    17.43             0           0     0         0     0        0      7
##    17.98             0           0     0         0     0        0      3
##    18.59             0           0     0         0     0        0      7
##    19.25             0           0     0         0     0        0     12
##    19.98             0           0     0         0     0        0      8
##    20.73             0           0     0         0     0        0      6
##    21.54             0           0     0         0     0        0      8
##    22.35             0           0     0         0     0        0      9
##    23.21             0           0     0         0     0        0     15
##    24.11             0           0     0         0     0        0     12
##    25.03             0           0     0         0     0        0      8
##    26.02             0           0     0         0     0        0      5
##    27.03             0           0     0         0     0        0      5
##    28.13             0           0     0         0     0        0      3
##    29.31             0           0     0         0     0        0      3
##    30.7              0           0     0         0     0        0      1
##    32.55             0           0     0         0     0        0      0
##    35                0           0     0         0     0        0      1
##
##          Switzerland Syria Taiwan Thailand Trinidad and Tobago Tunisia Turkey
##    7               0     0      0        0                   0       0      4
##    9.51            0     0      0        0                   0       0      1
##    11.25           0     0      0        0                   0       0      2
##    12.4            0     0      0        0                   0       0      2
##    13.33           0     0      0        0                   0       0      8
##    14.08           0     0      0        0                   0       0     14
##    14.75           0     0      0        0                   0       0     20
##    15.32           0     0      0        0                   0       0     24
##    15.84           0     0      0        0                   0       0     17
##    16.36           0     0      0        0                   0       0     34
##    16.88           0     0      0        0                   0       0     39
##    17.43           0     0      0        0                   0       0     45
##    17.98           0     0      0        0                   0       0     58
##    18.59           0     0      0        0                   0       0     59
##    19.25           0     0      0        0                   0       0     69
##    19.98           0     0      0        0                   0       0     84
##    20.73           0     0      0        0                   0       0     74
##    21.54           0     0      0        0                   0       0     94
##    22.35           0     0      0        0                   0       0     95
##    23.21           0     0      0        0                   0       0     94
##    24.11           0     0      0        0                   0       0     63
##    25.03           0     0      0        0                   0       0     91
##    26.02           0     0      0        0                   0       0     45
##    27.03           0     0      0        0                   0       0     29
##    28.13           0     0      0        0                   0       0     27
##    29.31           0     0      0        0                   0       0     23
##    30.7            0     0      0        0                   0       0     15
##    32.55           0     0      0        0                   0       0      8
##    35              0     0      0        0                   0       0     10
##
##          UAE Uganda  UK Ukraine Uruguay USA Vietnam Zambia
```

```
##   7       0      0   8       0       0   5       0      0
##   9.51    0      0   4       0       0   1       0      0
##   11.25   0      0   8       0       0   6       0      0
##   12.4    0      0  11       0       0   3       0      0
##   13.33   0      0  10       0       0   5       0      0
##   14.08   0      0  13       0       0   5       0      0
##   14.75   0      0  25       0       0  10       0      0
##   15.32   0      0  30       0       0  19       0      0
##   15.84   0      0  36       0       0  15       0      0
##   16.36   0      0  61       0       0  15       0      0
##   16.88   0      0  54       0       0  17       0      0
##   17.43   0      0  82       0       0  33       0      0
##   17.98   0      0 101       0       0  30       0      0
##   18.59   0      0 139       0       0  46       0      0
##   19.25   0      0 133       0       0  39       0      0
##   19.98   0      0 135       0       0  36       0      0
##   20.73   0      0 160       0       0  37       0      0
##   21.54   0      0 156       0       0  47       0      0
##   22.35   0      0 140       0       0  28       0      0
##   23.21   0      0 165       0       0  40       0      0
##   24.11   0      0 129       0       0  35       0      0
##   25.03   0      0 127       0       0  29       0      0
##   26.02   0      0  69       0       0   9       0      0
##   27.03   0      0  36       0       0   7       0      0
##   28.13   0      0  34       0       0   9       0      0
##   29.31   0      0  26       0       0   7       0      0
##   30.7    0      0  21       0       0   3       0      0
##   32.55   0      0  13       0       0   2       0      0
##   35      0      0  11       0       0   5       0      0
```

Dataset B, entire sample

```
# Bonding variables in entire sample
table(DatasetB_phase1$bond_country, DatasetB_phase1$country_now_Name)
```

```
##
##          Afghanistan Albania Algeria Argentina Armenia Australia Austria
##   0    4           1       1       2         6       2        94      14
##   1    1           1       0       1         5       1        41       0
##
##     Azerbaijan Bangladesh Belgium Belize Bermuda Bolivia Bosnia and Herzegovina
##   0          3        158      16      0       1       2                      1
##   1          0         97       2      1       0       1                      0
##
##     Brasil Bulgaria Canada Chile China Colombia Costa Rica Croatia
##   0     19        4     76     7     5       50          5       0
##   1      2        0     24     3     9       24          6       0
##
##     Czech Republic Denmark Ecuador Egypt Estonia Fiji Finland France Georgia
##   0              4      10       5     4       2    0       5    264       3
##   1              1       2       1     2       0    1       1     79       0
##
```

```
##     Germany Ghana Greece Guatemala Guernsey Guinea Guyana Hong Kong Hungary
##   0     194     3     11         2        1      1      1         5       3
##   1      24     1      6         1        1      0      0         3       1
##
##     Iceland India Indonesia Iran Iraq Ireland Israel Italy Japan Jersey Jordan
##   0       1    35         8   62    3      16      2    87     8      1      1
##   1       0    41         2    2    3       4      0    22     2      0      2
##
##     Kazakhstan Kenya Kuwait Kyrgyzstan Lebanon Lithuania Luxembourg Malaysia
##   0          1    16      0          0      16         9          8       15
##   1          0     3      1          0       5         4          1       11
##
##     Malta Mauritius Mexico Monaco Morocco Mozambique Namibia Nepal Netherlands
##   0     2         1     29      2       3          2       0     2          26
##   1     0         0      7      0       0          0       1     1           7
##
##     New Zealand Nigeria North Macedonia Norway Oman Pakistan Panama
##   0           9       7               2      3    3       11      1
##   1           7       3               0      3    0        6      0
##
##     Papua New Guinea Paraguay Peru Philippines Poland Portugal Puerto Rico
##   0                1        0  355           8      8        7           2
##   1                1        2  269           1      1        0           0
##
##     Qatar Romania Russia Saudi Arabia Senegal Serbia Singapore Slovakia
##   0     4      15     13            3       1      2         7        1
##   1     0       0      3            5       0      0         2        0
##
##     Slovenia Somalia South Africa South Korea Spain Sri Lanka Sudan Suriname
##   0        5       0           10           3    53         1     1        1
##   1        1       1            4           0    22         3     0        0
##
##     Sweden Switzerland Syria Taiwan Thailand Trinidad and Tobago Tunisia Turkey
##   0    104          20     1      0        9                   1       1    980
##   1     34           2     1      3        2                   0       2    131
##
##      UAE Uganda   UK Ukraine Uruguay  USA Vietnam Zambia
##   0   19      5 1525       2       2  481       1      2
##   1    7      0  393       0       1   55       0      0
```

```
table(DatasetB_phase1$bond_gvmt, DatasetB_phase1$country_now_Name)
```

```
##
##          Afghanistan Albania Algeria Argentina Armenia Australia Austria
##   0    4           2       1       3         8       3       125      14
##   1    1           0       0       0         3       0         9       0
##
##     Azerbaijan Bangladesh Belgium Belize Bermuda Bolivia Bosnia and Herzegovina
##   0          3        222      17      1       0       3                      1
##   1          0         19       0      0       1       0                      0
##
##     Brasil Bulgaria Canada Chile China Colombia Costa Rica Croatia
##   0     20        4     87    10     7       69          5       0
##   1      1        0     15     0     7        5          5       0
```

```
##
##     Czech Republic Denmark Ecuador Egypt Estonia Fiji Finland France Georgia
##   0              5      11       6     5       2    1       6    328       2
##   1              0       1       0     1       0    0       0     11       1
##
##     Germany Ghana Greece Guatemala Guernsey Guinea Guyana Hong Kong Hungary
##   0     204     4     15         2        1      1      1         7       4
##   1      11     0      2         1        1      0      0         0       0
##
##     Iceland India Indonesia Iran Iraq Ireland Israel Italy Japan Jersey Jordan
##   0       1    53        10   62    5      18      2    97    10      1      1
##   1       0    23         0    1    1       2      0    12     0      0      2
##
##     Kazakhstan Kenya Kuwait Kyrgyzstan Lebanon Lithuania Luxembourg Malaysia
##   0          1    18      0          0      20        12          6       20
##   1          0     1      0          0       1         1          2        3
##
##     Malta Mauritius Mexico Monaco Morocco Mozambique Namibia Nepal Netherlands
##   0     2         1     35      2       3          2       1     2          27
##   1     0         0      1      0       0          0       0     0           6
##
##     New Zealand Nigeria North Macedonia Norway Oman Pakistan Panama
##   0           9      10               2      4    3       13      1
##   1           7       0               0      2    0        3      0
##
##     Papua New Guinea Paraguay Peru Philippines Poland Portugal Puerto Rico
##   0                2        2  527           9      9        4           2
##   1                0        0   97           0      0        2           0
##
##     Qatar Romania Russia Saudi Arabia Senegal Serbia Singapore Slovakia
##   0     2      15     14            6       1      2         8        0
##   1     1       0      2            2       0      0         1        1
##
##     Slovenia Somalia South Africa South Korea Spain Sri Lanka Sudan Suriname
##   0        5       1           10           2    67         4     1        1
##   1        0       0            4           1     7         0     0        0
##
##     Sweden Switzerland Syria Taiwan Thailand Trinidad and Tobago Tunisia Turkey
##   0    126          20     0      2       11                   1       3   1013
##   1     12           2     1      1        0                   0       0     13
##
##      UAE Uganda   UK Ukraine Uruguay  USA Vietnam Zambia
##   0   19      5 1814       2       2  523       1      2
##   1    6      0  104       0       1   12       0      0
```

```
table(DatasetB_phase1$bond_mult, DatasetB_phase1$country_now_Name)
```

```
##
##          Afghanistan Albania Algeria Argentina Armenia Australia Austria
##   0    4           1       1       2         6       2        93      14
##   1    0           1       0       1         2       1        34       0
##   2    1           0       0       0         3       0         8       0
##
##     Azerbaijan Bangladesh Belgium Belize Bermuda Bolivia Bosnia and Herzegovina
```

```
##   0          3         158      16     0       0       2                      1
##   1          0          82       2     1       1       1                      0
##   2          0          17       0     0       0       0                      0
##
##     Brasil Bulgaria Canada Chile China Colombia Costa Rica Croatia
##   0     19        4     72     7     3       48          4       0
##   1      1        0     21     3     6       23          3       0
##   2      1        0      9     0     5        3          4       0
##
##     Czech Republic Denmark Ecuador Egypt Estonia Fiji Finland France Georgia
##   0              4      10       5     4       2    0       5    263       2
##   1              1       1       1     1       0    1       1     70       1
##   2              0       1       0     1       0    0       0     10       0
##
##     Germany Ghana Greece Guatemala Guernsey Guinea Guyana Hong Kong Hungary
##   0     190     3     11         2        1      1      1         5       3
##   1      21     1      4         0        0      0      0         3       1
##   2       7     0      2         1        1      0      0         0       0
##
##     Iceland India Indonesia Iran Iraq Ireland Israel Italy Japan Jersey Jordan
##   0       1    32         8   62    3      16      2    84     8      1      1
##   1       0    24         2    1    2       2      0    16     2      0      0
##   2       0    20         0    1    1       2      0     9     0      0      2
##
##     Kazakhstan Kenya Kuwait Kyrgyzstan Lebanon Lithuania Luxembourg Malaysia
##   0          1    16      0          0      16         9          7       14
##   1          0     2      1          0       4         3          1       10
##   2          0     1      0          0       1         1          1        2
##
##     Malta Mauritius Mexico Monaco Morocco Mozambique Namibia Nepal Netherlands
##   0     2         1     29      2       3          2       0     2          25
##   1     0         0      6      0       0          0       1     1           3
##   2     0         0      1      0       0          0       0     0           5
##
##     New Zealand Nigeria North Macedonia Norway Oman Pakistan Panama
##   0           6       7               2      3    3       11      1
##   1           6       3               0      1    0        3      0
##   2           4       0               0      2    0        3      0
##
##     Papua New Guinea Paraguay Peru Philippines Poland Portugal Puerto Rico
##   0                1        0  372           8      8        5           2
##   1                1        2  196           1      1        2           0
##   2                0        0   85           0      0        0           0
##
##     Qatar Romania Russia Saudi Arabia Senegal Serbia Singapore Slovakia
##   0     3      15     13            3       1      2         7        0
##   1     1       0      3            3       0      0         1        1
##   2     0       0      1            2       0      0         1        0
##
##     Slovenia Somalia South Africa South Korea Spain Sri Lanka Sudan Suriname
##   0        5       0            9           2    50         1     1        1
##   1        1       1            2           1    21         3     0        0
##   2        0       0            3           0     4         0     0        0
##
```

```
##     Sweden Switzerland Syria Taiwan Thailand Trinidad and Tobago Tunisia Turkey
##   0    104          19     1      0        9                   1       1    987
##   1     22           2     0      2        2                   0       2    120
##   2     12           1     1      1        0                   0       0     12
##
##      UAE Uganda   UK Ukraine Uruguay  USA Vietnam Zambia
##   0   18      5 1511       2       2  477       1      2
##   1    3      0  325       0       0   55       0      0
##   2    5      0   86       0       1    6       0      0
```

```r
# Health behaviours in entire sample
table(DatasetB_phase1$comply_self, DatasetB_phase1$country_now_Name)
```

```
##
##            Afghanistan Albania Algeria Argentina Armenia Australia Austria
##   1     0           0       0       0         0       0         0       0
##   2     0           0       0       0         0       0         0       0
##   3     0           0       0       0         0       0         0       0
##   4     0           0       0       0         0       0         0       0
##   5     0           0       0       0         0       0         0       0
##   6     0           0       0       0         0       0         0       0
##   7     0           0       0       0         0       0         0       0
##   8     0           0       0       0         0       0         0       0
##   9     0           0       0       0         0       0         0       0
##   10    0           0       0       0         1       0         0       0
##   11    0           0       0       0         0       0         0       0
##   12    0           0       0       0         0       0         0       0
##   13    0           0       0       0         0       0         0       0
##   14    0           0       0       0         0       0         0       0
##   15    1           0       0       0         0       0         0       0
##   16    0           0       0       0         0       0         1       0
##   17    0           0       0       0         0       0         0       0
##   18    0           0       0       0         0       0         0       0
##   19    0           0       0       0         0       0         0       0
##   20    0           0       0       0         0       0         0       0
##   21    0           0       0       0         0       0         1       0
##   22    0           0       0       0         0       0         0       0
##   23    0           0       0       0         0       0         0       0
##   24    0           0       0       0         0       0         0       0
##   25    0           0       0       0         0       0         1       0
##   26    0           0       0       0         0       0         0       0
##   27    0           0       0       0         0       0         1       0
##   28    0           0       0       0         0       0         0       0
##   29    0           0       0       0         0       0         0       0
##   30    0           0       0       0         0       0         1       0
##   31    0           0       1       0         0       0         0       0
##   32    0           0       0       0         0       0         1       0
##   33    0           0       0       0         0       0         0       0
##   34    0           0       0       0         0       0         0       0
##   35    0           0       0       0         0       0         1       0
##   36    0           0       0       0         0       0         2       0
##   37    0           0       0       0         1       0         2       0
##   38    0           0       0       0         0       0         1       0
##   39    0           0       0       0         0       0         2       0
```

```
##    40  0           0      0      0        0      0        0      1
##    41  0           0      0      0        0      0        3      0
##    42  0           0      0      0        0      0        2      0
##    43  0           0      0      0        0      0        0      0
##    44  0           0      0      0        0      0        1      0
##    45  0           0      0      1        0      0        1      0
##    46  0           0      0      0        0      0        0      0
##    47  0           0      0      0        0      0        0      0
##    48  0           0      0      0        0      0        1      0
##    49  0           0      0      0        0      1        4      1
##    50  2           1      0      0        2      1       29      5
##    51  0           0      0      1        1      0        2      0
##    52  0           0      0      0        1      0        3      0
##    53  0           0      0      0        0      0        5      0
##    54  0           0      0      0        0      0        1      0
##    55  0           0      0      0        0      0        4      0
##    56  0           0      0      0        0      0        1      0
##    57  1           0      0      0        1      0        0      0
##    58  0           0      0      0        0      0        1      0
##    59  0           0      0      0        0      0        1      0
##    60  0           0      0      0        0      0        1      0
##    61  0           0      0      0        0      0        1      0
##    62  0           0      0      0        0      0        1      0
##    63  0           0      0      0        1      0        2      1
##    64  0           0      0      0        0      0        1      0
##    65  0           0      0      0        0      0        3      0
##    66  0           0      0      1        0      0        3      0
##    67  0           0      0      0        0      0        0      1
##    68  1           0      0      0        0      0        3      0
##    69  0           0      0      0        0      0        2      0
##    70  0           1      0      0        0      0        3      0
##    71  0           0      0      0        0      0        1      0
##    72  0           0      0      0        0      0        3      1
##    73  0           0      0      0        0      0        0      0
##    74  0           0      0      0        0      0        2      0
##    75  0           0      0      0        0      0        2      0
##    76  0           0      0      0        0      0        1      1
##    77  0           0      0      0        0      0        0      1
##    78  0           0      0      0        0      0        0      0
##    79  0           0      0      0        0      0        1      0
##    80  0           0      0      0        0      1        1      0
##    81  0           0      0      0        0      0        0      0
##    82  0           0      0      0        0      0        0      0
##    83  0           0      0      0        0      0        0      0
##    84  0           0      0      0        0      0        4      0
##    85  0           0      0      0        0      0        0      0
##    86  0           0      0      0        0      0        2      0
##    87  0           0      0      0        0      0        0      0
##    88  0           0      0      0        0      0        0      0
##    89  0           0      0      0        0      0        1      0
##    90  0           0      0      0        0      0        1      0
##    91  0           0      0      0        1      0        0      0
##    92  0           0      0      0        1      0        0      0
##    93  0           0      0      0        0      0        5      0
```

```
##   94   0          0       0       0         0       0         0       0
##   95   0          0       0       0         0       0         3       0
##   96   0          0       0       0         0       0         1       0
##   97   0          0       0       0         0       0         2       0
##   98   0          0       0       0         0       0         0       0
##   99   0          0       0       0         0       0         0       0
##   100  1          0       0       0         1       0        12       2
##
##       Azerbaijan Bangladesh Belgium Belize Bermuda Bolivia
##   1            0          1       0      0       0       0
##   2            0          0       0      0       0       0
##   3            0          0       0      0       0       0
##   4            0          0       0      0       0       0
##   5            0          1       0      0       0       0
##   6            0          1       0      0       0       0
##   7            0          0       0      0       0       0
##   8            0          1       0      0       0       0
##   9            0          2       0      0       0       0
##   10           0          1       0      0       0       0
##   11           0          0       0      0       0       0
##   12           0          2       0      0       0       0
##   13           0          0       0      0       0       0
##   14           0          1       0      0       0       0
##   15           0          0       0      0       0       0
##   16           0          0       0      0       0       0
##   17           0          0       0      0       0       0
##   18           0          1       0      0       0       0
##   19           0          0       0      0       0       0
##   20           0          0       0      0       0       0
##   21           0          0       0      0       0       0
##   22           0          0       0      0       0       0
##   23           0          1       0      0       0       0
##   24           0          1       0      0       0       0
##   25           0          3       0      0       0       0
##   26           0          0       0      0       0       0
##   27           0          0       0      0       0       0
##   28           0          2       0      0       0       0
##   29           0          1       0      0       0       0
##   30           0          2       0      0       0       0
##   31           0          1       0      0       0       0
##   32           0          4       1      0       0       0
##   33           0          3       0      0       0       0
##   34           0          1       0      0       0       0
##   35           0          2       0      0       0       0
##   36           0          0       0      0       0       0
##   37           0          3       0      0       0       0
##   38           0          1       1      0       0       0
##   39           0          2       0      1       0       0
##   40           0          3       0      0       0       1
##   41           0          2       1      0       0       0
##   42           0          3       1      0       0       0
##   43           0          0       0      0       0       0
##   44           0          1       0      0       0       0
##   45           0          2       1      0       0       0
```

```
##   46          0          2       0      0       0       0
##   47          0          2       0      0       0       0
##   48          0          8       0      0       0       0
##   49          0         12       0      0       0       1
##   50          0         85       3      0       0       1
##   51          0         12       1      0       0       0
##   52          0         14       1      0       0       0
##   53          0          6       0      0       0       0
##   54          1          1       0      0       0       0
##   55          0          1       0      0       0       0
##   56          0          2       0      0       0       0
##   57          0          1       0      0       0       0
##   58          0          0       0      0       0       0
##   59          0          3       0      0       1       0
##   60          0          1       0      0       0       0
##   61          0          0       0      0       0       0
##   62          0          1       0      0       0       0
##   63          0          1       0      0       0       0
##   64          0          1       1      0       0       0
##   65          0          1       0      0       0       0
##   66          0          2       0      0       0       0
##   67          0          2       0      0       0       0
##   68          0          1       0      0       0       0
##   69          0          3       0      0       0       0
##   70          0          4       1      0       0       0
##   71          0          5       0      0       0       0
##   72          0          1       1      0       0       0
##   73          0          2       0      0       0       0
##   74          0          0       0      0       0       0
##   75          0          3       0      0       0       0
##   76          0          1       1      0       0       0
##   77          0          0       0      0       0       0
##   78          0          0       0      0       0       0
##   79          0          1       0      0       0       0
##   80          0          1       0      0       0       0
##   81          0          4       0      0       0       0
##   82          0          0       0      0       0       0
##   83          0          0       0      0       0       0
##   84          0          0       0      0       0       0
##   85          0          0       0      0       0       0
##   86          0          0       0      0       0       0
##   87          0          1       0      0       0       0
##   88          0          1       0      0       0       0
##   89          0          2       0      0       0       0
##   90          0          4       0      0       0       0
##   91          0          4       0      0       0       0
##   92          0          2       1      0       0       0
##   93          0          4       0      0       0       0
##   94          0          3       0      0       0       0
##   95          0          0       0      0       0       0
##   96          0          5       0      0       0       0
##   97          0          1       0      0       0       0
##   98          0          1       0      0       0       0
##   99          0          1       0      0       0       0
```

```
##   100           2          13        4       0       0       0
##
##       Bosnia and Herzegovina Brasil Bulgaria Canada Chile China Colombia
##   1                        0      0        0      0     0     0        0
##   2                        0      0        0      0     0     0        0
##   3                        0      0        0      0     0     0        0
##   4                        0      0        0      0     0     0        0
##   5                        0      0        0      0     0     0        0
##   6                        0      0        0      0     0     0        0
##   7                        0      0        0      0     0     0        0
##   8                        0      0        0      0     0     0        0
##   9                        0      0        0      0     0     0        0
##   10                       0      0        0      0     0     0        0
##   11                       0      0        0      0     0     0        0
##   12                       0      0        0      0     0     0        0
##   13                       0      0        0      0     0     0        0
##   14                       0      0        0      1     0     0        0
##   15                       0      0        0      0     0     0        0
##   16                       0      0        0      0     0     0        0
##   17                       0      0        0      0     0     0        0
##   18                       0      0        0      0     0     0        0
##   19                       0      0        0      0     0     0        0
##   20                       0      0        0      0     0     0        0
##   21                       0      0        0      1     0     0        0
##   22                       0      0        0      0     0     0        0
##   23                       0      0        0      0     0     0        0
##   24                       0      0        0      0     0     0        0
##   25                       0      0        0      0     0     0        0
##   26                       0      0        0      0     0     0        0
##   27                       0      0        0      2     0     0        0
##   28                       0      0        0      0     0     0        0
##   29                       0      0        0      2     0     0        1
##   30                       0      0        0      0     0     0        0
##   31                       0      0        0      0     0     0        1
##   32                       0      0        0      0     0     0        1
##   33                       0      1        1      0     0     0        2
##   34                       0      0        0      0     0     0        0
##   35                       0      1        0      0     0     0        1
##   36                       0      0        0      0     0     0        0
##   37                       0      0        0      2     0     0        1
##   38                       0      0        0      3     0     0        0
##   39                       0      0        0      1     0     0        1
##   40                       0      0        0      1     0     0        1
##   41                       0      0        0      1     0     0        1
##   42                       0      1        0      2     0     1        0
##   43                       0      0        0      1     0     0        1
##   44                       0      0        0      2     0     0        0
##   45                       0      0        0      1     0     1        1
##   46                       0      0        0      1     0     0        2
##   47                       0      0        0      0     0     0        0
##   48                       0      0        0      3     0     0        0
##   49                       0      1        0      3     0     0        0
##   50                       0      5        1     10     1     5       14
##   51                       0      0        0      3     0     0        4
```

```
##   52                       0      1        0      3     1     0        1
##   53                       0      0        0      3     0     0        2
##   54                       0      0        0      1     0     0        0
##   55                       0      1        0      2     0     0        0
##   56                       0      0        0      2     0     0        1
##   57                       0      0        0      0     0     0        0
##   58                       0      0        0      2     1     0        1
##   59                       0      1        0      1     0     0        1
##   60                       0      0        0      0     0     0        0
##   61                       0      0        0      1     0     1        0
##   62                       0      0        0      0     0     0        2
##   63                       0      0        0      0     0     0        1
##   64                       0      0        0      2     0     0        0
##   65                       1      0        0      1     0     0        2
##   66                       0      1        0      0     0     0        0
##   67                       0      1        0      1     0     0        0
##   68                       0      0        0      0     0     0        0
##   69                       0      0        0      2     0     0        2
##   70                       0      0        0      2     0     0        1
##   71                       0      1        0      1     0     0        2
##   72                       0      0        0      0     0     0        0
##   73                       0      0        0      0     0     0        2
##   74                       0      0        0      1     0     0        1
##   75                       0      0        0      2     0     2        0
##   76                       0      0        0      0     1     0        1
##   77                       0      0        0      1     1     0        0
##   78                       0      1        0      4     0     0        0
##   79                       0      0        0      0     0     0        2
##   80                       0      1        0      1     0     0        0
##   81                       0      0        0      4     0     0        1
##   82                       0      0        0      1     0     1        1
##   83                       0      0        0      1     0     0        0
##   84                       0      0        0      0     0     0        0
##   85                       0      0        0      1     0     1        2
##   86                       0      0        0      0     0     0        1
##   87                       0      1        0      1     0     0        0
##   88                       0      1        0      1     0     0        1
##   89                       0      0        0      1     1     0        0
##   90                       0      0        0      2     0     0        0
##   91                       0      0        0      0     0     0        2
##   92                       0      0        0      0     1     0        0
##   93                       0      0        0      0     0     0        0
##   94                       0      0        0      1     0     0        0
##   95                       0      0        0      1     0     1        2
##   96                       0      0        0      1     0     0        0
##   97                       0      0        0      0     0     0        3
##   98                       0      0        0      1     0     0        0
##   99                       0      1        1      2     0     0        2
##   100                      0      1        1     14     3     1        7
##
##       Costa Rica Croatia Czech Republic Denmark Ecuador Egypt Estonia Fiji
##   1            0       0              0       0       0     0       0    0
##   2            0       0              0       0       0     0       0    0
##   3            0       0              0       0       0     0       0    0
```

```
## 4           0    0       0    0    0    0     0  0
## 5           0    0       0    0    0    0     0  0
## 6           0    0       0    0    0    0     0  0
## 7           0    0       0    0    0    0     0  0
## 8           0    0       0    0    0    1     0  0
## 9           0    0       0    0    0    0     0  0
## 10          0    0       0    0    0    0     0  0
## 11          0    0       0    0    0    0     0  0
## 12          0    0       0    0    0    0     0  0
## 13          0    0       0    0    0    0     0  0
## 14          0    0       0    0    0    0     0  0
## 15          0    0       0    0    0    0     0  0
## 16          0    0       0    0    0    0     0  0
## 17          0    0       0    0    0    0     0  0
## 18          0    0       0    0    0    0     0  0
## 19          0    0       0    0    0    1     0  0
## 20          0    0       0    0    0    0     0  0
## 21          0    0       0    0    0    0     0  0
## 22          0    0       0    0    0    0     0  0
## 23          0    0       0    0    0    0     0  0
## 24          0    0       0    0    0    0     0  0
## 25          0    0       0    0    0    0     0  0
## 26          0    0       0    0    0    1     0  0
## 27          0    0       0    0    0    0     0  1
## 28          0    0       1    0    0    0     0  0
## 29          0    0       0    0    0    0     0  0
## 30          0    0       0    0    0    0     0  0
## 31          0    0       0    0    0    0     0  0
## 32          0    0       0    0    0    1     0  0
## 33          1    0       0    0    0    0     0  0
## 34          0    0       0    0    0    0     0  0
## 35          0    0       0    0    0    0     0  0
## 36          0    0       0    0    0    0     0  0
## 37          0    0       0    0    0    0     0  0
## 38          1    0       0    0    0    1     0  0
## 39          1    0       0    1    0    0     0  0
## 40          0    0       0    0    0    0     0  0
## 41          0    0       0    0    0    0     0  0
## 42          0    0       0    0    0    0     1  0
## 43          0    1       0    1    1    0     0  0
## 44          0    0       1    0    0    0     0  0
## 45          0    0       0    0    0    0     0  0
## 46          0    0       0    0    0    1     0  0
## 47          2    0       0    0    0    0     0  0
## 48          0    0       0    0    0    0     0  0
## 49          2    0       0    0    0    0     0  0
## 50          0    0       1    1    1    0     1  0
## 51          0    0       1    1    0    0     0  0
## 52          0    0       0    0    0    0     0  0
## 53          0    0       0    0    0    0     0  0
## 54          2    0       0    0    0    0     0  0
## 55          0    0       0    0    0    0     0  0
## 56          0    0       0    0    0    0     0  0
## 57          0    0       0    0    0    0     0  0
```

```
##   58           0       0               0       0       0     0       0    0
##   59           0       0               0       0       0     0       0    0
##   60           0       0               0       0       0     0       0    0
##   61           0       0               0       0       0     0       0    0
##   62           0       0               0       2       0     0       0    0
##   63           0       0               0       0       0     0       0    0
##   64           0       0               0       0       0     0       0    0
##   65           0       0               0       0       0     0       0    0
##   66           0       0               0       1       0     0       0    0
##   67           0       0               0       0       0     0       0    0
##   68           0       0               0       0       0     0       0    0
##   69           0       0               0       0       0     0       0    0
##   70           0       0               0       0       0     0       0    0
##   71           0       0               0       0       0     0       0    0
##   72           0       0               0       0       0     0       0    0
##   73           0       0               0       0       0     0       0    0
##   74           0       0               0       0       0     0       0    0
##   75           0       0               0       1       0     0       0    0
##   76           0       0               0       0       0     0       0    0
##   77           0       0               0       0       0     0       0    0
##   78           0       0               0       0       0     0       0    0
##   79           0       0               0       0       0     0       0    0
##   80           0       0               0       0       0     0       0    0
##   81           0       0               0       0       0     0       0    0
##   82           0       0               0       0       0     0       0    0
##   83           0       0               0       0       0     0       0    0
##   84           0       0               0       0       0     0       0    0
##   85           0       0               0       1       0     0       0    0
##   86           0       0               0       1       0     0       0    0
##   87           0       0               0       0       1     0       0    0
##   88           0       0               0       0       0     0       0    0
##   89           0       0               0       0       1     0       0    0
##   90           0       0               0       0       0     0       0    0
##   91           0       0               0       0       0     0       0    0
##   92           0       0               0       0       0     0       0    0
##   93           0       0               0       0       0     0       0    0
##   94           0       0               0       0       0     0       0    0
##   95           1       0               0       0       0     0       0    0
##   96           0       0               0       0       0     0       0    0
##   97           0       0               0       1       0     0       0    0
##   98           0       0               0       0       0     0       0    0
##   99           0       0               0       0       1     0       0    0
##   100          1       0               1       1       1     0       0    0
##
##        Finland France Georgia Germany Ghana Greece Guatemala Guernsey Guinea
##   1          0      1       0       1     0      0         0        0      0
##   2          0      0       0       0     0      0         0        0      0
##   3          0      0       0       0     0      0         0        0      0
##   4          0      0       0       0     0      0         0        0      0
##   5          0      0       0       0     0      0         0        0      0
##   6          0      0       0       0     0      0         0        0      0
##   7          0      0       0       0     0      0         0        0      0
##   8          0      0       0       0     0      0         0        0      0
##   9          0      1       0       0     0      0         0        0      0
```

```
##    10         0      1        0        0     0      0           0        0       0
##    11         0      0        0        0     0      0           0        0       0
##    12         0      0        0        0     0      0           0        0       0
##    13         0      0        0        0     0      0           0        0       0
##    14         0      0        0        0     0      0           0        0       0
##    15         0      1        0        0     0      0           0        0       0
##    16         0      0        0        0     0      0           0        0       0
##    17         0      0        0        1     0      0           0        0       0
##    18         0      0        0        0     0      0           0        0       0
##    19         0      1        0        0     0      0           0        0       0
##    20         0      1        0        1     0      0           0        0       0
##    21         0      0        0        0     0      0           0        0       0
##    22         0      0        0        0     0      0           0        0       0
##    23         0      0        0        1     0      0           0        0       0
##    24         0      1        0        1     0      0           0        0       0
##    25         0      0        0        1     0      0           0        0       0
##    26         0      0        0        3     0      0           0        0       0
##    27         0      1        0        2     0      0           0        0       0
##    28         0      1        0        0     0      0           0        0       0
##    29         0      0        0        1     0      0           0        0       0
##    30         0      2        0        1     0      0           0        0       0
##    31         0      1        0        0     0      0           0        0       0
##    32         0      5        0        1     0      2           0        0       0
##    33         0      2        0        0     0      1           0        0       0
##    34         0      5        0        1     0      0           0        0       0
##    35         0      2        0        1     0      0           0        0       0
##    36         0      6        0        2     0      0           0        0       0
##    37         0      4        0        4     0      0           0        0       0
##    38         0      2        0        2     0      0           0        0       0
##    39         0      4        0        2     0      0           0        0       0
##    40         0      1        0        3     0      0           0        0       0
##    41         0      2        0        3     0      0           0        0       0
##    42         0      6        0        5     0      0           0        0       0
##    43         0      1        0        2     0      0           0        0       0
##    44         0      2        1        9     0      0           0        0       0
##    45         0      5        0        1     0      0           0        0       0
##    46         0      5        0        5     0      1           0        0       0
##    47         0      3        0        6     0      0           0        0       0
##    48         1      7        0        5     0      0           0        0       0
##    49         0     11        0        3     0      0           0        0       0
##    50         0     37        0       21     0      3           1        0       0
##    51         0     15        0       10     0      1           1        0       0
##    52         0      4        1        6     0      1           0        0       0
##    53         0     10        0        4     0      1           0        0       0
##    54         0     11        0        0     0      0           0        0       0
##    55         1      4        0        4     0      0           0        0       0
##    56         0      3        0        1     0      0           0        0       0
##    57         0      4        0        0     0      0           0        0       0
##    58         0     10        0        2     0      0           0        0       0
##    59         0      4        0        1     0      0           0        0       0
##    60         0      8        0        3     0      2           0        0       0
##    61         0      4        0        2     0      0           0        0       0
##    62         0      4        0        1     0      0           0        0       0
##    63         0      3        0        1     0      1           0        0       0
```

```
##   64        1      2       0       1     0      0         0        0      0
##   65        0      1       1       6     0      0         0        0      0
##   66        0      4       0       1     0      0         0        0      0
##   67        0      3       0       3     0      1         0        1      0
##   68        0      5       0       2     1      0         0        0      0
##   69        0      7       0       4     0      0         0        0      0
##   70        0      8       0       1     0      0         0        0      0
##   71        0      3       0       4     0      0         0        0      0
##   72        0      5       0       4     0      0         0        0      0
##   73        0      4       0       2     0      0         0        0      0
##   74        0      5       0       2     0      1         0        0      0
##   75        0      3       0       5     1      0         0        0      0
##   76        0      2       0       1     0      1         0        1      0
##   77        0      6       0       1     0      0         0        0      0
##   78        0      3       0       1     1      0         0        0      0
##   79        0      3       0       4     0      0         0        0      0
##   80        1      2       0       3     0      0         0        0      0
##   81        0      4       0       1     0      0         0        0      0
##   82        0      3       0       1     0      0         0        0      0
##   83        1      3       0       4     0      0         0        0      0
##   84        0      1       0       1     0      0         0        0      0
##   85        0      4       0       2     0      0         0        0      0
##   86        0      0       0       2     0      0         0        0      0
##   87        0      4       0       3     0      0         0        0      0
##   88        0      4       0       6     0      0         0        0      0
##   89        0      3       0       2     0      0         0        0      0
##   90        0      2       0       3     0      0         0        0      0
##   91        1      3       0       1     0      0         0        0      0
##   92        0      3       0       0     1      0         0        0      0
##   93        0      2       0       2     0      0         0        0      0
##   94        0      1       0       3     0      0         0        0      0
##   95        0      2       0       0     0      0         0        0      0
##   96        0      3       0       0     0      0         0        0      0
##   97        0      3       0       1     0      0         0        0      0
##   98        0      2       0       0     0      0         0        0      0
##   99        0      4       0       1     0      0         0        0      1
##   100       0     21       0      19     0      1         1        0      0
##
##       Guyana Hong Kong Hungary Iceland India Indonesia Iran Iraq Ireland Israel
##   1        0         0       0       0     0         0    1    0       0      0
##   2        0         0       0       0     0         0    0    0       0      0
##   3        0         0       0       0     0         0    0    0       0      0
##   4        0         0       0       0     0         0    0    0       0      0
##   5        0         0       0       0     0         0    0    0       0      0
##   6        0         0       0       0     0         0    0    0       0      0
##   7        0         0       0       0     0         0    0    0       0      0
##   8        0         0       0       0     0         0    0    0       0      0
##   9        0         0       0       0     0         0    1    0       0      0
##   10       0         0       0       0     0         0    0    0       0      0
##   11       0         0       0       0     0         0    0    0       0      0
##   12       0         0       0       0     0         0    0    1       0      0
##   13       0         0       0       0     0         0    0    0       0      0
##   14       0         0       0       0     0         0    0    0       0      0
##   15       0         0       0       0     0         0    0    0       0      0
```

```
##   16       0       0      0      0    0        0   0   0      0     0
##   17       0       0      0      0    0        1   0   0      0     0
##   18       0       0      1      0    0        0   0   0      0     0
##   19       0       0      0      0    0        0   0   0      0     0
##   20       0       0      0      0    0        0   0   0      0     0
##   21       0       0      0      0    0        0   0   0      0     0
##   22       0       0      0      0    0        0   0   0      0     0
##   23       0       0      0      0    0        0   0   0      0     0
##   24       0       1      0      0    0        0   0   0      0     0
##   25       0       0      0      0    0        0   0   0      0     0
##   26       0       0      0      0    0        0   0   0      0     0
##   27       0       0      0      0    0        0   1   0      0     0
##   28       0       0      0      0    0        0   0   0      0     0
##   29       0       0      0      0    0        0   0   0      0     0
##   30       0       0      0      0    0        0   0   0      0     0
##   31       0       0      0      0    0        0   2   0      0     0
##   32       0       0      0      0    1        0   0   0      0     0
##   33       0       0      0      0    0        0   0   0      1     0
##   34       0       0      0      0    0        0   0   0      0     0
##   35       0       0      0      0    0        0   2   0      0     0
##   36       0       0      0      0    1        0   0   0      0     0
##   37       0       0      0      0    1        0   1   1      0     1
##   38       0       0      0      0    0        0   0   0      0     0
##   39       0       0      0      0    0        0   1   0      0     0
##   40       0       0      0      0    0        0   0   0      0     0
##   41       0       1      0      0    0        0   2   0      0     0
##   42       0       0      1      0    0        0   1   0      1     0
##   43       0       0      0      0    0        0   0   0      0     0
##   44       0       0      0      0    0        0   1   0      0     0
##   45       0       0      0      0    1        0   3   0      1     0
##   46       0       1      0      0    0        0   1   0      0     0
##   47       0       0      0      0    1        0   2   0      0     0
##   48       0       0      0      0    1        0   2   0      1     0
##   49       0       0      0      0    4        0   1   0      1     0
##   50       0       0      0      0   23        2   8   0      3     1
##   51       0       0      0      0    7        0   0   0      1     0
##   52       0       1      0      0    2        0   1   0      0     0
##   53       0       0      0      0    1        0   1   0      0     0
##   54       0       0      0      0    2        0   0   0      0     0
##   55       0       0      0      0    2        0   0   0      0     0
##   56       0       0      0      0    0        1   0   0      0     0
##   57       0       0      0      0    0        0   0   0      0     0
##   58       0       1      0      0    0        0   0   0      0     0
##   59       0       0      1      0    0        0   2   0      1     0
##   60       0       0      0      0    1        0   1   0      0     0
##   61       0       0      0      0    2        0   2   0      1     0
##   62       0       0      0      0    0        0   0   0      0     0
##   63       1       0      0      1    1        0   0   0      0     0
##   64       0       0      0      0    0        1   2   0      0     0
##   65       0       0      0      0    0        0   0   0      0     0
##   66       0       0      0      0    1        0   1   0      0     0
##   67       0       0      0      0    1        0   1   0      0     0
##   68       0       0      0      0    0        0   2   0      1     0
##   69       0       0      0      0    0        0   2   1      1     0
```

```
##   70        0         0       0       0     0         0    1    0       0      0
##   71        0         0       0       0     2         1    0    0       0      0
##   72        0         0       0       0     2         1    0    0       0      0
##   73        0         0       0       0     0         0    0    0       0      0
##   74        0         0       0       0     0         0    2    0       1      0
##   75        0         0       0       0     0         1    0    0       0      0
##   76        0         0       0       0     1         0    0    0       0      0
##   77        0         0       1       0     0         0    0    0       0      0
##   78        0         0       0       0     0         0    0    0       0      0
##   79        0         0       0       0     0         0    0    0       0      0
##   80        0         0       0       0     1         0    1    0       1      0
##   81        0         1       0       0     0         0    1    0       0      0
##   82        0         0       0       0     1         0    0    0       0      0
##   83        0         0       0       0     0         0    0    1       0      0
##   84        0         0       0       0     0         0    2    0       0      0
##   85        0         0       0       0     0         0    0    0       0      0
##   86        0         0       0       0     1         0    0    0       0      0
##   87        0         0       0       0     0         0    0    0       0      0
##   88        0         0       0       0     0         0    1    0       0      0
##   89        0         0       0       0     1         0    1    0       0      0
##   90        0         0       0       0     3         0    0    0       0      0
##   91        0         0       0       0     0         0    0    0       0      0
##   92        0         0       0       0     0         0    1    1       1      0
##   93        0         0       0       0     2         0    0    0       0      0
##   94        0         1       0       0     2         0    0    0       0      0
##   95        0         0       0       0     2         0    0    0       0      0
##   96        0         0       0       0     0         0    0    0       1      0
##   97        0         0       0       0     1         0    0    0       0      0
##   98        0         1       0       0     1         0    0    0       0      0
##   99        0         0       0       0     0         0    1    0       0      0
##   100       0         0       0       0     9         2    8    1       3      0
##
##        Italy Japan Jersey Jordan Kazakhstan Kenya Kuwait Kyrgyzstan Lebanon
##   1        2     0      0      0          0     0      0          0       1
##   2        1     0      0      0          0     0      0          0       0
##   3        1     0      0      0          0     0      0          0       0
##   4        0     0      0      0          0     0      0          0       0
##   5        0     0      0      0          0     0      0          0       0
##   6        1     0      0      0          0     0      0          0       0
##   7        0     0      0      0          0     0      0          0       0
##   8        0     0      0      0          0     0      0          0       0
##   9        0     0      0      0          0     0      0          0       0
##   10       0     0      0      0          0     0      0          0       0
##   11       0     0      0      0          0     0      0          0       0
##   12       0     0      0      0          0     0      0          0       0
##   13       0     0      0      0          0     0      0          0       0
##   14       0     0      0      0          0     0      0          0       0
##   15       0     0      0      0          0     0      0          0       0
##   16       0     0      0      0          0     0      0          0       0
##   17       0     0      0      0          0     0      0          0       0
##   18       0     0      0      0          0     0      0          0       0
##   19       1     0      0      0          0     0      0          0       0
##   20       0     0      0      0          0     0      0          0       0
##   21       0     0      0      0          0     0      0          0       0
```

```
##    22     0    0     0     0          0    0     0          0       0
##    23     0    0     0     0          0    1     0          0       0
##    24     0    0     0     0          0    0     0          0       0
##    25     0    0     0     0          0    0     0          0       0
##    26     0    0     0     0          0    0     0          0       0
##    27     2    0     0     0          0    1     0          0       0
##    28     0    0     0     0          0    0     0          0       0
##    29     0    0     0     0          0    0     0          0       0
##    30     0    0     0     0          0    0     0          0       0
##    31     0    0     0     0          0    0     0          0       0
##    32     0    0     0     0          0    0     0          0       0
##    33     0    0     0     0          0    0     0          0       0
##    34     3    0     0     0          0    0     0          0       0
##    35     1    0     0     0          0    0     0          0       0
##    36     0    0     0     0          0    0     0          0       0
##    37     1    0     0     0          0    1     0          0       0
##    38     1    0     0     0          0    0     0          0       2
##    39     1    0     0     0          0    0     0          0       0
##    40     1    0     0     0          0    0     0          0       1
##    41     0    0     0     0          0    0     0          0       0
##    42     1    0     0     0          0    0     0          0       0
##    43     2    1     0     0          0    0     0          0       1
##    44     2    0     0     0          0    0     0          0       0
##    45     0    0     0     0          0    0     0          0       0
##    46     0    1     0     0          0    0     0          0       0
##    47     1    0     0     0          0    0     0          0       0
##    48     1    0     0     0          0    1     0          0       0
##    49     3    1     0     0          1    1     0          0       1
##    50    20    2     0     2          0    3     0          1       6
##    51     3    0     0     0          0    2     0          0       2
##    52     3    0     0     0          0    1     0          0       0
##    53     0    1     0     0          0    0     0          0       0
##    54     2    0     0     0          0    0     0          0       0
##    55     1    0     0     0          0    0     0          0       0
##    56     2    0     0     0          0    2     0          0       0
##    57     2    0     0     0          0    0     0          0       0
##    58     2    0     0     0          0    0     0          0       1
##    59     1    0     0     0          0    0     0          0       0
##    60     0    0     0     0          0    0     0          0       0
##    61     2    0     0     0          0    0     0          0       0
##    62     4    0     0     0          0    0     0          0       0
##    63     1    0     0     0          0    0     0          0       0
##    64     1    0     0     0          0    0     0          0       0
##    65     1    0     0     0          0    0     0          0       0
##    66     0    0     0     0          0    0     0          0       0
##    67     0    0     0     0          0    0     0          0       0
##    68     0    1     0     0          0    0     0          0       0
##    69     1    1     0     0          0    1     0          0       1
##    70     2    0     0     0          0    0     0          0       0
##    71     3    0     0     0          0    0     0          0       0
##    72     2    0     0     0          0    0     0          0       0
##    73     3    0     0     0          0    0     0          0       0
##    74     1    0     1     0          0    0     0          0       0
##    75     0    0     0     0          0    1     0          0       0
```

```
##   76      2     0      0      0          0     0      0          0       0
##   77      1     0      0      0          0     0      0          0       0
##   78      0     0      0      0          0     0      0          0       0
##   79      1     0      0      0          0     0      0          0       0
##   80      1     1      0      0          0     0      0          0       0
##   81      1     0      0      0          0     0      0          0       0
##   82      1     0      0      0          0     0      0          0       0
##   83      1     0      0      0          0     0      0          0       0
##   84      0     0      0      0          0     0      0          0       0
##   85      1     0      0      0          0     0      0          0       0
##   86      0     0      0      0          0     0      0          0       0
##   87      1     0      0      0          0     0      0          0       0
##   88      0     0      0      0          0     1      0          0       0
##   89      1     0      0      0          0     0      0          0       0
##   90      0     0      0      0          0     1      0          0       1
##   91      0     0      0      0          0     0      0          0       0
##   92      1     0      0      0          0     0      0          0       0
##   93      0     0      0      0          0     0      1          0       1
##   94      1     0      0      0          0     1      0          0       0
##   95      2     0      0      0          0     0      0          0       0
##   96      0     0      0      0          0     0      0          0       1
##   97      1     0      0      0          0     0      0          0       0
##   98      0     0      0      0          0     0      0          0       0
##   99      2     0      0      0          0     0      0          0       0
##   100    11     1      0      1          0     1      0          0       2
##
##       Lithuania Luxembourg Malaysia Malta Mauritius Mexico Monaco Morocco
##   1           0          0        0     0         0      0      0       0
##   2           0          0        0     1         0      0      0       0
##   3           0          0        0     0         0      0      0       1
##   4           0          0        0     0         0      0      0       0
##   5           0          0        0     0         0      0      0       0
##   6           0          0        0     0         0      0      0       0
##   7           0          0        0     0         0      0      0       0
##   8           0          0        0     0         0      0      0       0
##   9           0          0        0     0         0      0      0       0
##   10          0          0        0     0         0      0      0       0
##   11          0          0        0     0         0      0      0       0
##   12          0          0        0     0         0      0      0       0
##   13          0          0        0     0         0      0      0       0
##   14          0          0        0     0         0      0      0       0
##   15          0          0        0     0         0      0      0       0
##   16          0          0        0     0         0      0      0       0
##   17          0          0        0     0         0      0      0       0
##   18          0          0        0     0         0      0      0       0
##   19          0          0        0     0         0      0      0       0
##   20          0          0        0     0         0      0      0       0
##   21          0          0        0     0         0      0      0       0
##   22          0          0        0     0         0      0      0       0
##   23          0          0        0     0         0      0      0       0
##   24          0          0        0     0         0      0      0       0
##   25          0          0        1     0         0      1      0       0
##   26          0          0        0     0         0      0      0       0
##   27          0          0        0     0         0      0      0       0
```

```
##    28          0          0        0     0        0      0      0       0
##    29          0          0        0     0        0      0      0       0
##    30          0          0        0     0        0      2      0       0
##    31          0          0        0     0        0      0      0       0
##    32          0          0        0     0        0      2      0       0
##    33          0          0        0     0        0      1      0       0
##    34          0          0        0     0        0      0      0       0
##    35          0          0        0     0        0      0      0       0
##    36          0          0        0     0        0      1      0       0
##    37          0          0        0     0        0      1      0       0
##    38          0          0        0     0        0      0      0       0
##    39          0          0        0     0        0      1      0       0
##    40          1          0        0     0        0      0      0       0
##    41          0          0        0     0        0      0      0       0
##    42          0          0        0     0        0      0      0       0
##    43          0          0        0     0        0      0      0       0
##    44          0          0        0     0        0      0      0       0
##    45          1          0        0     0        0      0      0       0
##    46          0          0        0     0        0      0      0       0
##    47          0          1        0     0        0      0      0       0
##    48          0          0        0     0        0      2      0       0
##    49          1          0        1     0        0      2      0       0
##    50          3          0        3     0        0      6      0       0
##    51          1          0        0     0        0      5      0       0
##    52          0          1        1     0        0      0      0       0
##    53          0          0        0     0        0      0      0       0
##    54          0          0        0     0        0      0      0       0
##    55          0          0        0     0        0      0      0       0
##    56          0          0        0     0        0      0      0       0
##    57          0          0        2     0        0      0      0       0
##    58          0          0        0     0        0      0      0       0
##    59          0          0        1     0        0      0      0       0
##    60          0          0        1     0        0      0      0       0
##    61          0          0        0     0        0      0      0       0
##    62          0          0        2     0        0      0      0       0
##    63          0          0        0     0        0      0      0       0
##    64          0          0        0     0        0      0      0       0
##    65          0          0        1     0        0      0      0       0
##    66          0          0        0     0        0      1      0       0
##    67          0          0        0     0        0      0      0       0
##    68          0          0        1     0        0      0      0       0
##    69          0          0        1     0        0      1      0       0
##    70          0          0        0     0        0      0      0       1
##    71          1          0        0     0        0      1      0       0
##    72          0          0        0     0        0      1      0       0
##    73          0          0        0     0        0      1      0       0
##    74          0          0        1     0        0      0      0       0
##    75          0          0        0     0        0      0      0       0
##    76          1          0        0     0        0      0      0       0
##    77          0          0        0     0        0      0      0       1
##    78          0          2        0     0        0      0      0       0
##    79          0          0        0     0        0      0      0       0
##    80          0          2        1     0        0      0      0       0
##    81          0          0        0     0        0      0      0       0
```

```
##   82         0          0        0     0         0      0      0       0
##   83         0          0        0     0         0      1      0       0
##   84         0          0        1     0         0      2      0       0
##   85         0          0        0     1         0      0      0       0
##   86         1          0        2     0         0      0      0       0
##   87         0          0        0     0         0      0      0       0
##   88         0          0        1     0         0      1      0       0
##   89         0          1        0     0         0      0      0       0
##   90         0          0        0     0         0      0      1       0
##   91         0          0        0     0         0      0      0       0
##   92         0          0        0     0         0      1      0       0
##   93         0          0        1     0         0      0      0       0
##   94         0          0        0     0         0      0      0       0
##   95         0          0        0     0         0      0      0       0
##   96         0          0        0     0         0      0      0       0
##   97         0          0        0     0         0      0      0       0
##   98         0          0        0     0         0      0      0       0
##   99         0          0        0     0         0      0      0       0
##   100        3          2        4     0         1      2      1       0
##
##       Mozambique Namibia Nepal Netherlands New Zealand Nigeria North Macedonia
##   1            0       0     0           0           0       0               0
##   2            0       0     0           0           0       0               0
##   3            0       0     0           0           0       1               0
##   4            0       0     0           0           0       0               0
##   5            0       0     0           0           0       0               0
##   6            0       0     0           0           0       0               0
##   7            0       0     0           0           0       0               0
##   8            1       0     0           0           0       0               0
##   9            0       0     0           0           0       0               0
##   10           0       0     0           0           0       0               0
##   11           0       0     0           0           0       0               0
##   12           0       0     0           0           0       0               0
##   13           0       0     0           0           0       0               0
##   14           0       0     0           0           0       0               0
##   15           0       0     0           0           0       0               0
##   16           0       0     0           0           0       0               0
##   17           0       0     0           0           0       0               0
##   18           0       0     0           0           0       0               0
##   19           0       0     0           0           0       0               0
##   20           0       0     0           0           0       0               0
##   21           0       0     0           0           0       0               0
##   22           0       0     0           0           0       0               0
##   23           0       0     0           0           0       0               0
##   24           0       0     0           1           0       0               0
##   25           0       0     0           0           0       0               0
##   26           0       0     0           0           0       0               0
##   27           0       0     0           0           0       0               0
##   28           0       0     0           0           0       0               0
##   29           0       0     0           2           0       0               0
##   30           0       0     0           0           0       0               0
##   31           0       0     0           0           0       0               0
##   32           0       0     0           0           0       0               0
##   33           0       0     1           0           0       0               0
```

```
##    34              0       0     0           0           0       0               0
##    35              0       0     0           0           0       0               0
##    36              0       0     0           0           0       0               0
##    37              0       0     0           2           0       0               0
##    38              0       0     0           0           0       0               0
##    39              0       0     0           0           0       0               0
##    40              0       0     0           0           0       0               0
##    41              0       0     0           1           1       0               0
##    42              0       0     0           0           0       0               0
##    43              0       0     0           0           1       0               0
##    44              0       0     0           0           0       0               0
##    45              0       0     0           1           0       0               0
##    46              0       0     0           0           0       0               0
##    47              0       0     0           0           0       0               0
##    48              0       0     0           0           0       1               0
##    49              0       0     0           1           0       3               0
##    50              1       0     0           7           2       4               0
##    51              0       0     0           2           1       0               0
##    52              0       0     0           0           0       0               0
##    53              0       0     0           0           0       0               0
##    54              0       0     1           1           1       0               0
##    55              0       0     0           1           0       0               0
##    56              0       0     0           0           0       0               0
##    57              0       0     0           0           0       0               0
##    58              0       0     0           1           0       0               0
##    59              0       0     0           0           0       0               0
##    60              0       0     0           0           1       0               0
##    61              0       0     0           0           0       0               0
##    62              0       0     0           0           1       0               0
##    63              0       0     0           0           1       0               0
##    64              0       0     0           1           0       0               0
##    65              0       0     0           0           0       0               0
##    66              0       0     1           0           0       0               0
##    67              0       0     0           1           0       0               0
##    68              0       0     0           1           0       0               0
##    69              0       0     0           0           0       0               0
##    70              0       0     0           1           0       0               0
##    71              0       0     0           1           0       0               0
##    72              0       0     0           0           0       0               0
##    73              0       0     0           0           1       0               0
##    74              0       0     0           1           0       0               0
##    75              0       0     0           0           0       0               0
##    76              0       0     0           0           0       0               0
##    77              0       0     0           0           1       0               0
##    78              0       0     0           0           1       0               0
##    79              0       0     0           0           1       0               0
##    80              0       0     0           0           0       0               0
##    81              0       0     0           0           0       0               0
##    82              0       0     0           0           0       0               0
##    83              0       0     0           0           0       0               0
##    84              0       0     0           0           0       0               0
##    85              0       0     0           0           0       0               0
##    86              0       0     0           0           0       0               0
##    87              0       0     0           2           0       0               0
```

```
##   88          0       0     0           1           1       0               0
##   89          0       0     0           0           0       0               0
##   90          0       1     0           0           0       0               0
##   91          0       0     0           0           2       0               0
##   92          0       0     0           2           0       1               0
##   93          0       0     0           0           0       0               0
##   94          0       0     0           0           0       0               1
##   95          0       0     0           0           0       0               0
##   96          0       0     0           0           0       0               0
##   97          0       0     0           1           0       0               0
##   98          0       0     0           0           0       0               1
##   99          0       0     0           0           0       0               0
##   100         0       0     0           1           0       0               0
##
##       Norway Oman Pakistan Panama Papua New Guinea Paraguay Peru Philippines
##   1        0    0        0      0                0        0    1           0
##   2        0    0        0      0                0        0    0           0
##   3        0    0        0      0                0        0    0           0
##   4        0    0        0      0                0        0    0           0
##   5        0    0        0      0                0        0    1           0
##   6        0    0        0      0                0        0    0           0
##   7        0    0        0      0                0        0    1           0
##   8        0    0        0      0                0        0    0           0
##   9        0    0        0      0                0        0    3           0
##   10       0    0        1      0                0        0    2           0
##   11       0    0        0      0                0        0    1           0
##   12       0    0        0      0                0        0    0           0
##   13       0    0        0      0                0        0    1           0
##   14       0    0        0      0                0        0    4           0
##   15       0    0        0      0                0        0    1           0
##   16       0    0        0      0                0        0    1           0
##   17       0    0        0      0                0        0    2           0
##   18       0    0        0      0                0        0    1           0
##   19       0    0        0      0                0        0    0           0
##   20       0    0        0      0                0        0    1           0
##   21       0    0        0      0                0        0    0           0
##   22       0    0        0      0                0        0    0           0
##   23       0    0        0      0                0        0    0           0
##   24       0    0        0      0                0        0    1           0
##   25       0    0        0      0                1        0    0           0
##   26       0    0        0      0                0        0    1           0
##   27       0    0        0      0                0        0    2           0
##   28       0    0        0      0                0        1    3           0
##   29       0    0        0      0                0        0    1           0
##   30       0    0        0      0                0        0    3           0
##   31       0    0        0      0                0        0    5           0
##   32       0    0        0      0                0        0    2           0
##   33       0    0        0      0                0        0    2           0
##   34       0    0        0      0                0        0    5           0
##   35       0    0        0      0                0        0    6           0
##   36       0    0        1      0                0        0    4           0
##   37       0    0        0      0                0        0    6           1
##   38       0    0        0      0                0        0    6           0
##   39       0    0        0      0                0        0    5           0
```

```
##   40         1    1        0       0                   0         0     5             0
##   41         0    0        0       0                   0         0     3             0
##   42         0    0        0       0                   0         0     2             0
##   43         0    0        0       0                   0         0     4             0
##   44         0    0        0       0                   0         0     2             0
##   45         0    0        0       0                   0         0     4             0
##   46         0    0        0       0                   0         0     3             0
##   47         0    0        0       0                   0         0     9             0
##   48         0    1        0       0                   0         0    10             0
##   49         0    0        0       0                   0         0    29             0
##   50         1    0        3       1                   0         0   263             0
##   51         0    0        0       0                   0         0    28             0
##   52         0    0        1       0                   0         0    17             1
##   53         0    0        0       0                   0         0    14             0
##   54         0    0        0       0                   0         0    10             0
##   55         0    0        0       0                   0         0     5             0
##   56         0    0        0       0                   0         0     1             0
##   57         0    0        0       0                   0         0     3             0
##   58         0    0        0       0                   0         0     4             0
##   59         0    0        0       0                   0         0     2             0
##   60         0    0        0       0                   0         0     2             0
##   61         0    0        1       0                   0         0     3             0
##   62         0    0        0       0                   0         0     2             0
##   63         0    0        0       0                   0         0     0             0
##   64         0    0        0       0                   0         0     4             0
##   65         0    0        1       0                   0         0     7             1
##   66         0    0        0       0                   0         0     3             0
##   67         0    0        0       0                   0         0     4             0
##   68         0    0        1       0                   1         0     7             0
##   69         0    0        0       0                   0         0     8             0
##   70         0    0        0       0                   0         0     6             0
##   71         0    0        0       0                   0         0     3             0
##   72         0    0        0       0                   0         0     3             0
##   73         0    1        0       0                   0         0     3             1
##   74         0    0        1       0                   0         0     3             0
##   75         0    0        1       0                   0         0     5             0
##   76         0    0        0       0                   0         0     3             0
##   77         0    0        1       0                   0         0     5             0
##   78         0    0        0       0                   0         0     4             0
##   79         0    0        0       0                   0         0     3             1
##   80         0    0        0       0                   0         0     3             0
##   81         0    0        1       0                   0         0     4             0
##   82         0    0        0       0                   0         0     2             1
##   83         0    0        0       0                   0         0     2             0
##   84         1    0        0       0                   0         0     3             0
##   85         0    0        0       0                   0         0     3             0
##   86         0    0        0       0                   0         0     5             0
##   87         1    0        0       0                   0         0     6             1
##   88         0    0        0       0                   0         0     6             0
##   89         0    0        0       0                   0         0     6             0
##   90         0    0        0       0                   0         0    12             0
##   91         0    0        0       0                   0         0     8             0
##   92         0    0        0       0                   0         0     7             0
##   93         0    0        0       0                   0         0     9             0
```

```
##   94       0    0        0      0                0        0   11           0
##   95       0    0        0      0                0        0    7           0
##   96       0    0        1      0                0        0    8           0
##   97       1    0        0      0                0        0    7           1
##   98       0    0        0      0                0        0    6           0
##   99       0    0        1      0                0        0    5           0
##   100      1    0        2      0                0        1   36           3
##
##       Poland Portugal Puerto Rico Qatar Romania Russia Saudi Arabia Senegal
##   1        0        0           0     0       0      0            0       1
##   2        0        0           0     0       0      0            0       0
##   3        0        0           0     0       0      0            0       0
##   4        0        0           0     0       0      0            0       0
##   5        0        0           0     0       0      0            0       0
##   6        0        0           0     0       0      0            0       0
##   7        0        0           0     0       0      0            0       0
##   8        0        0           0     0       0      0            0       0
##   9        0        0           0     0       0      0            0       0
##   10       0        0           0     0       0      0            0       0
##   11       0        0           0     0       0      0            0       0
##   12       0        0           0     0       0      0            0       0
##   13       0        0           0     0       0      0            0       0
##   14       0        0           0     0       0      0            0       0
##   15       0        0           0     0       0      0            0       0
##   16       0        0           0     0       0      0            0       0
##   17       0        0           0     0       0      0            0       0
##   18       0        0           0     0       0      0            0       0
##   19       0        0           0     0       0      0            0       0
##   20       0        0           0     0       0      0            0       0
##   21       0        0           0     0       0      0            0       0
##   22       0        0           0     0       0      0            0       0
##   23       0        0           0     0       1      0            0       0
##   24       0        0           0     0       0      0            0       0
##   25       0        0           0     0       0      0            0       0
##   26       0        0           0     0       1      0            0       0
##   27       0        0           0     0       0      1            0       0
##   28       0        0           0     0       0      0            0       0
##   29       0        0           0     0       0      0            0       0
##   30       0        0           0     0       0      0            0       0
##   31       0        0           0     0       0      0            0       0
##   32       0        0           0     0       0      1            0       0
##   33       0        0           0     0       1      0            1       0
##   34       0        1           0     0       1      0            0       0
##   35       0        0           0     0       0      0            0       0
##   36       0        0           0     0       0      0            0       0
##   37       0        0           0     1       0      0            0       0
##   38       0        0           0     0       0      0            0       0
##   39       0        0           0     0       0      0            0       0
##   40       0        0           0     0       0      0            0       0
##   41       0        0           0     0       0      0            0       0
##   42       1        0           0     0       0      0            0       0
##   43       0        0           0     0       0      0            0       0
##   44       0        0           0     0       0      0            0       0
##   45       0        0           0     0       0      0            0       0
```

```
##   46      0      0        0    0     0     0        0      0
##   47      0      0        0    0     0     0        0      0
##   48      0      0        0    1     0     0        0      0
##   49      0      1        0    0     0     1        1      0
##   50      2      2        0    0     0     5        1      0
##   51      0      1        0    0     1     1        1      0
##   52      0      0        0    0     1     0        0      0
##   53      0      0        0    0     0     0        0      0
##   54      0      0        0    0     0     0        0      0
##   55      0      0        1    0     0     0        0      0
##   56      0      0        0    1     0     0        0      0
##   57      0      0        0    0     1     1        0      0
##   58      0      0        0    0     0     1        0      0
##   59      0      0        0    0     1     0        0      0
##   60      0      0        0    0     1     0        0      0
##   61      1      0        0    0     0     0        0      0
##   62      0      0        0    0     0     0        0      0
##   63      0      0        0    0     0     0        0      0
##   64      0      0        0    0     0     0        0      0
##   65      0      0        0    0     0     0        0      0
##   66      0      0        0    0     0     0        0      0
##   67      0      0        0    0     0     0        0      0
##   68      0      0        0    0     0     0        0      0
##   69      0      0        1    0     0     1        0      0
##   70      0      0        0    0     0     1        0      0
##   71      0      0        0    0     0     0        0      0
##   72      0      0        0    0     2     0        1      0
##   73      0      0        0    0     0     0        0      0
##   74      0      0        0    0     0     0        1      0
##   75      0      0        0    0     1     0        0      0
##   76      0      0        0    0     0     0        0      0
##   77      0      0        0    0     0     0        0      0
##   78      0      0        0    1     0     0        0      0
##   79      0      0        0    0     1     1        0      0
##   80      0      0        0    0     0     0        0      0
##   81      0      0        0    0     0     0        0      0
##   82      1      0        0    0     0     0        1      0
##   83      0      0        0    0     0     0        1      0
##   84      0      0        0    0     0     0        0      0
##   85      0      0        0    0     0     0        0      0
##   86      1      0        0    0     0     0        0      0
##   87      0      0        0    0     0     1        0      0
##   88      0      0        0    0     0     1        0      0
##   89      0      0        0    0     0     0        0      0
##   90      0      0        0    0     0     1        0      0
##   91      1      0        0    0     0     0        0      0
##   92      0      0        0    0     0     0        0      0
##   93      0      1        0    0     0     0        0      0
##   94      0      0        0    0     0     0        0      0
##   95      0      0        0    0     0     0        0      0
##   96      0      1        0    0     0     0        0      0
##   97      0      0        0    0     2     0        0      0
##   98      0      0        0    0     0     0        0      0
##   99      0      0        0    0     0     0        0      0
```

```
## 100      2         0          0      0       0     0          0       0
##
##     Serbia Singapore Slovakia Slovenia Somalia South Africa South Korea Spain
## 1        0         0        0        0       0            0           0     0
## 2        0         0        0        0       0            0           0     0
## 3        0         0        0        0       0            0           0     0
## 4        0         0        0        0       0            0           0     0
## 5        0         0        0        0       0            0           0     0
## 6        0         0        0        0       0            0           0     0
## 7        0         0        0        0       0            0           0     0
## 8        0         0        0        0       0            0           0     0
## 9        0         0        0        0       0            0           0     0
## 10       0         0        0        0       0            0           0     0
## 11       0         0        0        0       0            0           0     0
## 12       0         0        0        0       0            0           0     0
## 13       0         0        0        0       0            0           0     0
## 14       0         0        0        0       0            0           0     0
## 15       0         0        0        0       0            0           0     0
## 16       0         0        0        0       0            0           0     0
## 17       0         0        0        0       0            0           0     0
## 18       0         0        0        0       0            0           0     0
## 19       0         0        0        0       0            0           0     0
## 20       0         0        0        0       0            0           0     0
## 21       0         0        0        0       0            0           0     1
## 22       0         0        0        0       0            0           0     0
## 23       0         0        0        0       0            0           0     0
## 24       0         0        0        0       0            0           0     0
## 25       0         0        0        0       0            0           0     0
## 26       0         0        0        0       0            0           0     0
## 27       0         0        0        0       0            0           0     0
## 28       0         0        0        0       0            0           0     0
## 29       0         0        0        0       0            0           1     0
## 30       0         0        0        0       0            0           1     0
## 31       0         0        0        0       0            0           0     0
## 32       0         0        0        0       0            0           0     0
## 33       0         0        0        0       0            0           0     0
## 34       0         0        0        0       0            0           0     1
## 35       0         0        0        0       0            0           0     1
## 36       0         0        0        0       0            0           0     0
## 37       0         0        0        0       0            0           0     0
## 38       0         0        0        0       0            0           0     0
## 39       0         0        0        0       0            0           1     0
## 40       0         0        0        0       0            1           0     1
## 41       0         0        0        0       0            1           0     0
## 42       0         0        0        0       0            0           0     2
## 43       0         0        0        0       0            2           0     0
## 44       0         0        0        0       0            0           0     1
## 45       0         0        0        0       0            0           0     2
## 46       0         0        0        0       0            0           0     2
## 47       0         0        0        0       1            0           0     1
## 48       0         0        0        0       0            0           0     1
## 49       0         1        0        0       0            0           0     1
## 50       0         3        0        3       0            4           0    13
## 51       0         0        1        1       0            0           0     3
```

```
##   52      0          0        0        0       0            2           0    5
##   53      0          0        0        0       0            0           0    2
##   54      0          0        0        1       0            0           0    1
##   55      0          0        0        0       0            0           0    0
##   56      0          0        0        0       0            0           0    1
##   57      0          0        0        0       0            0           0    1
##   58      0          0        0        0       0            0           0    1
##   59      0          1        0        0       0            0           0    2
##   60      0          0        0        0       0            0           0    0
##   61      0          0        0        0       0            0           0    0
##   62      0          0        0        0       0            0           0    1
##   63      0          0        0        0       0            0           0    0
##   64      0          0        0        0       0            0           0    1
##   65      0          0        0        0       0            0           0    0
##   66      0          0        0        0       0            0           0    2
##   67      0          0        0        0       0            0           0    1
##   68      0          0        0        0       0            0           0    3
##   69      0          0        0        0       0            0           0    1
##   70      0          0        0        0       0            0           0    0
##   71      0          0        0        0       0            0           0    0
##   72      0          0        0        0       0            0           0    0
##   73      0          0        0        0       0            0           0    1
##   74      0          0        0        0       0            2           0    1
##   75      0          0        0        0       0            0           0    0
##   76      0          0        0        0       0            1           0    2
##   77      0          0        0        0       0            0           0    0
##   78      0          1        0        0       0            0           0    1
##   79      0          0        0        0       0            0           0    1
##   80      0          0        0        0       0            0           0    1
##   81      0          0        0        0       0            0           0    0
##   82      0          0        0        0       0            0           0    1
##   83      0          0        0        0       0            0           0    0
##   84      0          1        0        0       0            0           0    1
##   85      0          1        0        0       0            0           0    0
##   86      0          0        0        0       0            0           0    0
##   87      0          0        0        0       0            0           0    1
##   88      0          0        0        0       0            0           0    0
##   89      0          0        0        0       0            0           0    0
##   90      0          1        0        0       0            0           0    1
##   91      0          0        0        0       0            1           0    0
##   92      0          0        0        0       0            0           0    2
##   93      0          0        0        0       0            0           0    0
##   94      0          0        0        0       0            0           0    0
##   95      0          0        0        0       0            0           0    0
##   96      0          0        0        0       0            0           0    1
##   97      0          0        0        0       0            0           0    0
##   98      0          0        0        0       0            0           0    0
##   99      0          0        0        0       0            0           0    0
##   100     2          0        0        1       0            0           0    9
##
##       Sri Lanka Sudan Suriname Sweden Switzerland Syria Taiwan Thailand
##   1           0     0        0      2           0     0      1        0
##   2           0     0        0      0           0     0      0        0
##   3           0     0        0      0           0     0      0        0
```

```
##   4        0   0     0   0       0   0   0     0
##   5        0   0     0   0       0   0   0     0
##   6        0   0     0   0       0   0   0     0
##   7        0   0     0   0       0   0   0     0
##   8        0   0     0   0       0   0   0     0
##   9        0   0     0   0       0   0   0     0
##   10       0   0     0   0       0   1   0     0
##   11       0   0     0   0       0   0   0     0
##   12       0   0     0   0       0   0   0     0
##   13       0   0     0   0       0   0   0     0
##   14       0   0     0   0       0   0   0     0
##   15       0   0     0   1       0   0   0     0
##   16       0   0     0   0       0   0   0     0
##   17       0   0     0   1       0   0   0     0
##   18       0   0     0   0       0   0   0     0
##   19       0   0     0   0       0   0   0     0
##   20       0   0     0   0       0   0   0     0
##   21       0   0     0   0       0   0   0     0
##   22       0   0     0   0       0   0   0     0
##   23       0   0     0   0       0   0   0     0
##   24       0   0     0   0       0   0   0     0
##   25       0   0     0   0       0   0   0     0
##   26       0   0     0   0       0   0   0     0
##   27       0   0     0   0       0   0   0     0
##   28       0   0     0   2       0   0   0     0
##   29       0   0     0   1       0   0   0     0
##   30       0   0     0   0       0   0   0     0
##   31       0   0     0   0       0   0   0     0
##   32       0   0     0   0       0   0   0     0
##   33       0   0     0   2       0   0   0     0
##   34       0   0     0   1       0   0   0     0
##   35       0   0     0   1       0   0   0     0
##   36       0   0     0   2       0   0   0     0
##   37       0   0     0   1       0   0   0     0
##   38       0   0     0   0       0   0   1     0
##   39       0   0     0   1       1   0   0     0
##   40       0   0     0   1       0   0   0     0
##   41       0   0     0   2       0   0   0     0
##   42       0   0     0   0       1   0   0     0
##   43       0   0     0   1       0   0   0     0
##   44       0   0     0   1       0   0   0     0
##   45       0   0     0   4       0   0   0     0
##   46       0   0     0   1       0   0   0     0
##   47       0   0     0   2       1   0   0     0
##   48       0   0     0   2       0   0   0     0
##   49       1   0     0   2       1   0   0     1
##   50       1   0     1  20       1   0   0     2
##   51       0   0     0   2       1   0   0     0
##   52       0   0     0   0       0   0   0     0
##   53       0   0     0   2       0   0   0     0
##   54       0   1     0   0       0   0   0     0
##   55       0   0     0   0       0   0   0     0
##   56       0   0     0   0       0   0   0     0
##   57       0   0     0   0       1   0   0     0
```

```
##   58          0     0        0      1           0     0      0        0
##   59          0     0        0      1           0     0      0        0
##   60          0     0        0      0           1     0      0        1
##   61          0     0        0      4           0     0      0        1
##   62          0     0        0      1           0     0      0        0
##   63          0     0        0      1           0     0      0        0
##   64          0     0        0      1           0     0      0        0
##   65          0     0        0      1           0     0      0        0
##   66          0     0        0      0           0     0      0        0
##   67          0     0        0      0           0     0      0        0
##   68          0     0        0      1           0     0      0        1
##   69          1     0        0      2           1     0      0        0
##   70          0     0        0      4           0     0      0        0
##   71          0     0        0      4           0     0      0        0
##   72          0     0        0      1           1     0      0        1
##   73          0     0        0      1           0     0      0        0
##   74          0     0        0      1           0     0      0        0
##   75          0     0        0      1           0     0      0        0
##   76          0     0        0      2           0     0      0        0
##   77          0     0        0      3           1     0      0        1
##   78          0     0        0      3           0     1      0        0
##   79          0     0        0      4           0     0      1        0
##   80          0     0        0      0           0     0      0        1
##   81          0     0        0      3           0     0      0        0
##   82          0     0        0      0           1     0      0        0
##   83          0     0        0      0           1     0      0        0
##   84          0     0        0      0           0     0      0        0
##   85          1     0        0      2           0     0      0        0
##   86          0     0        0      1           1     0      0        0
##   87          0     0        0      0           0     0      0        0
##   88          0     0        0      1           0     0      0        1
##   89          0     0        0      2           0     0      0        0
##   90          0     0        0      1           0     0      0        0
##   91          0     0        0      1           0     0      0        0
##   92          0     0        0      1           0     0      0        0
##   93          0     0        0      0           2     0      0        0
##   94          0     0        0      2           0     0      0        0
##   95          0     0        0      2           0     0      0        0
##   96          0     0        0      1           0     0      0        1
##   97          0     0        0      0           1     0      0        0
##   98          0     0        0      3           0     0      0        0
##   99          0     0        0      2           1     0      0        0
##   100         0     0        0     24           4     0      0        0
##
##       Trinidad and Tobago Tunisia Turkey UAE Uganda  UK Ukraine Uruguay USA
##   1                     0       0      3   0      0   2       0       0   2
##   2                     0       0      0   0      0   0       0       0   2
##   3                     0       0      0   0      0   0       0       0   0
##   4                     0       0      1   0      0   1       0       0   0
##   5                     0       0      0   0      0   0       0       0   0
##   6                     0       0      0   0      0   0       0       0   0
##   7                     0       0      0   0      0   0       0       0   0
##   8                     0       0      1   0      0   0       0       0   0
##   9                     0       0      0   0      0   0       0       0   0
```

```
##    10                 0       0     0   0     0   0       0       0   1
##    11                 0       0     0   0     0   0       0       0   0
##    12                 0       0     0   0     0   0       0       0   1
##    13                 0       0     0   0     0   0       0       0   1
##    14                 0       0     0   0     0   0       0       0   0
##    15                 0       0     0   0     0   0       0       0   0
##    16                 0       0     1   0     0   0       0       0   1
##    17                 0       0     0   0     0   0       0       0   0
##    18                 0       0     1   0     0   0       0       0   2
##    19                 0       0     1   0     0   2       0       0   0
##    20                 0       0     0   0     0   1       0       0   2
##    21                 0       0     0   0     0   0       0       0   1
##    22                 0       0     1   0     0   0       1       0   0
##    23                 0       0     1   0     0   0       0       0   1
##    24                 0       0     1   0     0   0       0       0   1
##    25                 0       0     2   0     0   1       0       0   3
##    26                 0       0     2   0     0   1       0       0   6
##    27                 0       0     0   0     0   1       0       0   6
##    28                 0       0     6   0     0   6       0       0   1
##    29                 0       0     6   0     0   5       0       0   2
##    30                 0       0     3   0     0   2       0       0   5
##    31                 0       0     5   0     0   3       0       0   4
##    32                 0       0     3   0     0   5       0       0   1
##    33                 0       0     5   0     0   1       0       0   3
##    34                 0       0     4   0     0   2       0       0   5
##    35                 0       0     4   0     0  11       0       0   4
##    36                 0       0     8   1     1  11       0       0   2
##    37                 0       0     8   0     0  16       0       1   8
##    38                 0       0     6   0     0  13       0       0   7
##    39                 0       0    11   0     0  15       0       0   9
##    40                 0       0    11   0     0  17       0       0   8
##    41                 0       0     6   0     0  19       0       0   6
##    42                 0       0     8   0     0  24       0       0   7
##    43                 0       0     6   1     0  28       0       0   7
##    44                 0       0     6   0     0  30       0       0   7
##    45                 0       0     4   0     0  24       0       0   8
##    46                 0       0    10   0     0  20       0       0   1
##    47                 0       0    16   0     0  24       0       0   6
##    48                 0       0    14   0     0  27       0       0   6
##    49                 0       0    38   0     1  43       0       0  11
##    50                 0       1   166   4     1 374       1       0  69
##    51                 1       0    36   2     0  74       0       0  13
##    52                 0       1    17   1     0  49       0       0   8
##    53                 0       0    14   0     0  32       0       0   3
##    54                 0       0     9   0     0  34       0       0   4
##    55                 0       0     9   0     0  26       0       0   1
##    56                 0       0     6   1     1  16       0       0   8
##    57                 0       0     5   1     0  21       0       0   4
##    58                 0       1    12   0     0  20       0       0   6
##    59                 0       0    11   1     0  28       0       0   5
##    60                 0       0     7   0     0  25       0       0   4
##    61                 0       0     5   0     0  20       0       0   4
##    62                 0       0    12   0     0  25       0       1   5
##    63                 0       0    15   1     0  19       0       0   7
```

```
##    64                       0        0      16   1        0   23         0        0    6
##    65                       0        0      11   1        0   30         0        0    2
##    66                       0        0      15   0        0   18         0        0    5
##    67                       0        0       7   0        0   30         0        0    5
##    68                       0        0      12   0        0   24         0        0    5
##    69                       0        0      15   1        0   34         0        0    5
##    70                       0        0      12   1        0   26         0        0   10
##    71                       0        0      20   1        0   34         0        0    8
##    72                       0        0      22   0        0   23         0        0    9
##    73                       0        0      17   0        0   21         0        0    5
##    74                       0        0      12   1        1   29         0        0    2
##    75                       0        0      15   0        0   23         0        0    9
##    76                       0        0      10   0        0   25         0        0    6
##    77                       0        0      14   0        0   19         0        1    8
##    78                       0        0       9   0        0   20         0        0    6
##    79                       0        0      10   0        0   19         0        0    4
##    80                       0        0      12   0        0   12         0        0    9
##    81                       0        0       7   0        0   17         0        0    6
##    82                       0        0       8   0        0   14         0        0    2
##    83                       0        0       5   0        0    8         0        0    5
##    84                       0        0       9   0        0    9         0        0    4
##    85                       0        0       7   0        0   12         0        0    6
##    86                       0        0       8   0        0   15         0        0    4
##    87                       0        0      10   0        0    9         0        0    5
##    88                       0        0       9   0        0   13         0        0    5
##    89                       0        0       6   0        0   14         0        0    5
##    90                       0        0      13   0        0   13         0        0    7
##    91                       0        0      13   0        0   14         0        0    5
##    92                       0        0       9   0        0    7         0        0    3
##    93                       0        0      12   0        0   19         0        0    1
##    94                       0        0      12   0        0    5         0        0    8
##    95                       0        0      12   1        0   11         0        0    4
##    96                       0        0      11   0        1   14         0        0    3
##    97                       0        0      11   0        0    9         0        0    5
##    98                       0        0      13   0        0   13         0        0    5
##    99                       0        0      19   0        0   20         0        0    5
##    100                      0        0     198   7        1  173         0        0   62
##
##        Vietnam Zambia
##    1         1      0
##    2         0      0
##    3         0      0
##    4         0      0
##    5         0      0
##    6         0      0
##    7         0      0
##    8         0      0
##    9         0      0
##    10        0      0
##    11        0      0
##    12        0      0
##    13        0      0
##    14        0      0
##    15        0      0
```

```
##   16         0       0
##   17         0       0
##   18         0       0
##   19         0       0
##   20         0       0
##   21         0       0
##   22         0       0
##   23         0       0
##   24         0       0
##   25         0       0
##   26         0       0
##   27         0       0
##   28         0       0
##   29         0       0
##   30         0       0
##   31         0       0
##   32         0       0
##   33         0       0
##   34         0       0
##   35         0       0
##   36         0       0
##   37         0       0
##   38         0       0
##   39         0       0
##   40         0       0
##   41         0       0
##   42         0       0
##   43         0       0
##   44         0       0
##   45         0       0
##   46         0       0
##   47         0       0
##   48         0       0
##   49         0       0
##   50         0       2
##   51         0       0
##   52         0       0
##   53         0       0
##   54         0       0
##   55         0       0
##   56         0       0
##   57         0       0
##   58         0       0
##   59         0       0
##   60         0       0
##   61         0       0
##   62         0       0
##   63         0       0
##   64         0       0
##   65         0       0
##   66         0       0
##   67         0       0
##   68         0       0
##   69         0       0
```

```
##   70         0      0
##   71         0      0
##   72         0      0
##   73         0      0
##   74         0      0
##   75         0      0
##   76         0      0
##   77         0      0
##   78         0      0
##   79         0      0
##   80         0      0
##   81         0      0
##   82         0      0
##   83         0      0
##   84         0      0
##   85         0      0
##   86         0      0
##   87         0      0
##   88         0      0
##   89         0      0
##   90         0      0
##   91         0      0
##   92         0      0
##   93         0      0
##   94         0      0
##   95         0      0
##   96         0      0
##   97         0      0
##   98         0      0
##   99         0      0
##   100        0      0
```

```
# Wellbeing in entire sample
table(DatasetB_phase1$wellbeing, DatasetB_phase1$country_now_Name)
```

```
##
##                Afghanistan Albania Algeria Argentina Armenia Australia Austria
##   7        0             0       0       0         0       0         1       2
##   9.51     0             0       0       0         0       0         0       0
##   11.25    0             0       0       0         1       0         1       0
##   12.4     0             0       0       0         0       0         0       0
##   13.33    1             0       0       0         0       0         0       0
##   14.08    0             1       0       0         0       0         0       0
##   14.75    0             0       0       0         0       0         0       0
##   15.32    0             0       0       0         0       0         1       0
##   15.84    0             0       0       0         0       0         2       0
##   16.36    0             0       0       0         0       0         5       1
##   16.88    1             0       0       0         0       0         2       1
##   17.43    0             0       0       0         0       0         8       0
##   17.98    0             0       0       1         0       0        10       0
##   18.59    0             0       0       0         1       1         8       1
##   19.25    0             0       0       0         1       0        18       1
##   19.98    0             0       0       0         0       0         4       0
##   20.73    2             1       0       0         2       0         5       3
```

```
##   21.54  0          0       0       0         1       0         8       1
##   22.35  1          0       0       0         0       1        11       0
##   23.21  0          0       1       0         2       0        13       1
##   24.11  0          0       0       1         2       0        18       0
##   25.03  0          0       0       0         1       0         4       2
##   26.02  0          0       0       1         0       1         2       0
##   27.03  0          0       0       0         0       0         4       0
##   28.13  0          0       0       0         0       0         4       0
##   29.31  0          0       0       0         0       0         2       1
##   30.7   0          0       0       0         0       0         0       0
##   32.55  0          0       0       0         0       0         1       0
##   35     1          0       0       0         0       0         3       0
##
##         Azerbaijan Bangladesh Belgium Belize Bermuda Bolivia
##   7              1          1       0      0       0       0
##   9.51           0          0       0      0       0       0
##   11.25          0          1       0      0       0       0
##   12.4           0          1       0      0       0       0
##   13.33          0          2       0      0       0       0
##   14.08          0          4       0      0       0       0
##   14.75          0          1       0      0       0       0
##   15.32          0          1       0      0       0       0
##   15.84          0          5       0      0       0       0
##   16.36          0          8       0      0       0       0
##   16.88          0          3       1      0       0       1
##   17.43          0          6       1      0       0       0
##   17.98          0          8       1      0       0       0
##   18.59          0         13       1      0       0       0
##   19.25          0         11       2      0       0       0
##   19.98          0         10       2      0       0       1
##   20.73          0         17       0      0       0       0
##   21.54          0         20       1      0       1       0
##   22.35          1         18       2      1       0       0
##   23.21          0         11       2      0       0       0
##   24.11          0         15       0      0       0       0
##   25.03          0         29       1      0       0       0
##   26.02          0         26       0      0       0       1
##   27.03          0         21       0      0       0       0
##   28.13          0         14       3      0       0       0
##   29.31          0          7       0      0       0       0
##   30.7           1         11       2      0       0       0
##   32.55          0          3       0      0       0       0
##   35             0          8       0      0       0       0
##
##         Bosnia and Herzegovina Brasil Bulgaria Canada Chile China Colombia
##   7                          0      1        0      1     0     0        0
##   9.51                       0      0        0      0     0     0        0
##   11.25                      0      0        0      1     0     0        0
##   12.4                       0      0        0      1     0     0        1
##   13.33                      0      0        0      0     0     0        0
##   14.08                      0      0        0      2     0     0        0
##   14.75                      0      0        0      0     0     0        1
##   15.32                      0      0        0      3     0     1        1
##   15.84                      0      1        0      3     0     0        1
```

```
##   16.36                      0      0        0      5     0     0        1
##   16.88                      0      0        0      6     1     0        0
##   17.43                      0      3        0      5     0     0        3
##   17.98                      0      1        0     12     1     0        1
##   18.59                      0      0        0      6     1     2        3
##   19.25                      0      4        1     12     0     1        5
##   19.98                      0      0        0     10     0     0        8
##   20.73                      0      1        0      5     2     2        5
##   21.54                      0      5        0     12     0     0        7
##   22.35                      0      2        0      7     1     1        4
##   23.21                      0      0        0      1     1     3        5
##   24.11                      1      1        0      4     0     0        6
##   25.03                      0      1        1      5     3     0        6
##   26.02                      0      0        1      1     0     0        2
##   27.03                      0      1        1      1     0     0        7
##   28.13                      0      0        0      1     0     1        2
##   29.31                      0      0        0      1     0     0        3
##   30.7                       0      0        0      0     0     1        1
##   32.55                      0      0        0      0     0     0        1
##   35                         0      0        0      0     0     2        2
##
##         Costa Rica Croatia Czech Republic Denmark Ecuador Egypt Estonia Fiji
##   7              0       0              0       0       0     0       0    0
##   9.51           0       0              0       0       0     0       0    0
##   11.25          0       0              0       0       0     0       0    0
##   12.4           0       0              0       0       0     0       0    0
##   13.33          0       0              0       0       0     0       0    0
##   14.08          0       0              0       0       0     0       0    0
##   14.75          0       0              0       0       0     0       0    0
##   15.32          0       0              0       1       0     0       0    0
##   15.84          0       0              0       0       0     0       0    0
##   16.36          0       0              0       0       0     0       0    0
##   16.88          0       0              0       0       0     0       0    0
##   17.43          0       1              0       1       0     0       0    0
##   17.98          0       0              1       1       0     1       0    0
##   18.59          0       0              0       2       0     0       0    0
##   19.25          0       0              0       0       2     0       0    0
##   19.98          1       0              1       0       0     0       0    0
##   20.73          0       0              1       1       1     2       1    0
##   21.54          0       0              0       0       1     0       0    0
##   22.35          2       0              1       2       0     0       0    0
##   23.21          0       0              0       3       0     1       0    1
##   24.11          4       0              0       0       1     0       1    0
##   25.03          0       0              0       1       0     1       0    0
##   26.02          0       0              1       0       0     1       0    0
##   27.03          2       0              0       0       0     0       0    0
##   28.13          0       0              0       0       1     0       0    0
##   29.31          2       0              0       0       0     0       0    0
##   30.7           0       0              0       0       0     0       0    0
##   32.55          0       0              0       0       0     0       0    0
##   35             0       0              0       0       0     0       0    0
##
##         Finland France Georgia Germany Ghana Greece Guatemala Guernsey Guinea
##   7           0      0       0       0     0      0         0        0      0
```

```
##   9.51         0      0      0      0     0      0        0        0      0
##   11.25        0      1      0      1     0      0        0        0      0
##   12.4         0      0      0      0     0      0        0        0      0
##   13.33        0      0      0      1     0      0        0        0      0
##   14.08        0      1      0      2     0      0        0        0      0
##   14.75        0      0      0      4     0      1        0        0      0
##   15.32        0      3      0      1     0      0        0        0      0
##   15.84        0      0      0      4     0      0        0        0      0
##   16.36        0      5      0      4     0      2        0        1      1
##   16.88        1     10      0      9     0      0        0        0      0
##   17.43        0     15      0      7     1      1        0        0      0
##   17.98        0     18      0     10     0      0        1        0      0
##   18.59        0     19      1     17     0      2        0        0      0
##   19.25        1     20      0     19     0      0        0        1      0
##   19.98        0     23      0     12     0      2        1        0      0
##   20.73        1     36      0     12     0      1        0        0      0
##   21.54        1     39      0     19     1      2        0        0      0
##   22.35        0     32      1     22     0      3        0        0      0
##   23.21        1     32      0     21     1      0        0        0      0
##   24.11        1     31      0     19     1      1        0        0      0
##   25.03        0     18      0     16     0      0        0        0      0
##   26.02        0     14      1      9     0      1        0        0      0
##   27.03        0     17      0      3     0      0        1        0      0
##   28.13        0      4      0      3     0      1        0        0      0
##   29.31        0      2      0      3     0      0        0        0      0
##   30.7         0      2      0      2     0      0        0        0      0
##   32.55        0      1      0      0     0      0        0        0      0
##   35           0      1      0      1     0      0        0        0      0
##
##         Guyana Hong Kong Hungary Iceland India Indonesia Iran Iraq Ireland
##   7          0         0       0       0     1         0    0    0       0
##   9.51       0         0       0       0     1         0    0    0       0
##   11.25      0         0       0       0     0         0    0    0       0
##   12.4       0         0       0       0     0         0    0    0       0
##   13.33      0         0       0       0     2         0    0    0       0
##   14.08      0         0       0       0     1         0    1    0       1
##   14.75      0         0       0       0     1         0    2    0       0
##   15.32      0         0       0       0     1         0    2    1       0
##   15.84      0         0       0       0     1         1    1    0       0
##   16.36      0         1       0       0     1         0    2    0       0
##   16.88      0         0       0       0     2         0    3    0       0
##   17.43      0         0       0       0     4         1    4    0       1
##   17.98      0         0       0       0     4         0    2    0       1
##   18.59      0         2       1       0     2         1    4    0       0
##   19.25      0         0       0       0     2         0    3    0       1
##   19.98      0         1       0       0     8         0    4    1       2
##   20.73      1         0       1       0     2         1    5    0       2
##   21.54      0         2       0       0     4         1    4    0       4
##   22.35      0         0       0       0     6         1    7    0       2
##   23.21      0         0       0       1     6         0    1    1       1
##   24.11      0         0       0       0     5         1    7    0       1
##   25.03      0         1       1       0     9         0    3    1       2
##   26.02      0         0       0       0     1         0    2    1       0
##   27.03      0         0       1       0     3         1    3    1       0
```

```
##    28.13       0         0       0       0     2         0     2    0        0
##    29.31       0         0       0       0     2         0     2    0        1
##    30.7        0         0       0       0     5         0     0    0        0
##    32.55       0         0       0       0     2         1     0    0        1
##    35          0         1       0       0     4         1     0    0        0
##
##          Israel Italy Japan Jersey Jordan Kazakhstan Kenya Kuwait Kyrgyzstan
##    7          0     1     0      0      0          0     0      0          0
##    9.51       0     0     0      0      0          0     0      0          0
##    11.25      0     0     0      0      0          0     0      0          0
##    12.4       0     1     0      0      0          0     0      0          0
##    13.33      0     0     0      0      0          0     0      0          0
##    14.08      0     1     0      0      0          0     0      0          0
##    14.75      0     2     0      0      0          0     0      0          0
##    15.32      0     2     0      0      0          0     0      0          0
##    15.84      1     4     2      0      0          0     1      0          0
##    16.36      0     2     0      0      0          0     1      0          0
##    16.88      0     5     2      0      1          0     1      1          0
##    17.43      0     8     0      0      0          1     0      0          0
##    17.98      0     4     1      0      0          0     2      0          0
##    18.59      0     6     0      0      0          0     0      0          0
##    19.25      0     7     0      0      0          0     1      0          0
##    19.98      0    10     2      0      0          0     0      0          0
##    20.73      0     6     1      0      0          0     3      0          0
##    21.54      0     8     0      0      0          0     0      0          0
##    22.35      0    13     0      1      0          0     2      0          0
##    23.21      0     7     0      0      1          0     1      0          0
##    24.11      1     7     2      0      0          0     3      0          0
##    25.03      0     8     0      0      0          0     0      0          0
##    26.02      0     7     0      0      0          0     1      0          1
##    27.03      0     1     0      0      0          0     1      0          0
##    28.13      0     1     0      0      0          0     0      0          0
##    29.31      0     0     0      0      0          0     1      0          0
##    30.7       0     1     0      0      0          0     0      0          0
##    32.55      0     0     0      0      0          0     0      0          0
##    35         0     0     0      0      1          0     1      0          0
##
##          Lebanon Lithuania Luxembourg Malaysia Malta Mauritius Mexico Monaco
##    7           0         0          0        0     0         0      0      0
##    9.51        0         0          0        0     0         0      0      0
##    11.25       0         0          0        0     0         0      0      0
##    12.4        0         0          0        0     0         0      0      0
##    13.33       0         0          0        1     0         0      0      0
##    14.08       0         0          0        1     0         0      1      0
##    14.75       0         0          0        2     0         0      0      0
##    15.32       0         0          0        1     0         0      0      0
##    15.84       0         1          1        1     0         0      0      0
##    16.36       0         0          1        2     0         0      1      0
##    16.88       1         0          0        0     0         0      1      0
##    17.43       2         1          0        0     0         0      1      0
##    17.98       1         0          0        1     0         0      2      0
##    18.59       1         2          0        2     0         0      3      0
##    19.25       1         0          0        2     0         0      1      0
##    19.98       1         2          0        2     0         0      1      0
```

```
##   20.73        0         1          0        2     1         0      6      0
##   21.54        2         2          1        4     0         0      2      1
##   22.35        4         1          1        0     0         0      2      0
##   23.21        1         0          2        2     0         0      3      0
##   24.11        3         1          0        0     0         1      2      0
##   25.03        2         2          1        2     0         0      3      0
##   26.02        1         0          0        0     0         0      4      0
##   27.03        1         0          1        0     1         0      1      0
##   28.13        0         0          0        0     0         0      2      1
##   29.31        0         0          1        0     0         0      0      0
##   30.7         0         0          0        1     0         0      0      0
##   32.55        0         0          0        0     0         0      0      0
##   35           0         0          0        0     0         0      0      0
##
##         Morocco Mozambique Namibia Nepal Netherlands New Zealand Nigeria
##   7           0          0       0     0           0           0       0
##   9.51        0          0       0     0           0           0       0
##   11.25       0          0       0     0           0           0       0
##   12.4        0          0       0     0           0           0       0
##   13.33       0          0       0     0           1           0       0
##   14.08       0          0       0     0           1           0       0
##   14.75       0          0       0     0           0           0       0
##   15.32       1          0       0     0           0           0       0
##   15.84       0          0       0     0           0           0       0
##   16.36       0          0       0     0           1           0       0
##   16.88       0          0       0     0           1           2       0
##   17.43       0          0       0     0           1           0       0
##   17.98       0          0       0     0           0           1       0
##   18.59       0          1       0     1           1           3       0
##   19.25       0          0       0     0           1           0       1
##   19.98       0          0       0     0           4           3       0
##   20.73       0          0       0     0           5           0       1
##   21.54       0          0       0     0           1           0       0
##   22.35       0          0       0     2           3           1       1
##   23.21       1          1       0     0           2           0       1
##   24.11       1          0       0     0           3           2       1
##   25.03       0          0       0     0           3           2       1
##   26.02       0          0       1     0           2           1       1
##   27.03       0          0       0     0           3           0       1
##   28.13       0          0       0     0           0           1       1
##   29.31       0          0       0     0           0           0       0
##   30.7        0          0       0     0           0           0       1
##   32.55       0          0       0     0           0           0       0
##   35          0          0       0     0           0           0       0
##
##         North Macedonia Norway Oman Pakistan Panama Papua New Guinea Paraguay
##   7                   0      0    0        0      0                0        0
##   9.51                0      0    0        0      0                0        0
##   11.25               0      0    0        0      0                0        0
##   12.4                0      0    0        0      0                0        0
##   13.33               0      0    0        0      0                0        0
##   14.08               1      0    0        0      0                0        0
##   14.75               0      0    0        0      0                0        0
##   15.32               0      1    0        0      0                0        0
```

```
##   15.84               0      0    0        1      0                0        0
##   16.36               0      0    0        1      0                0        0
##   16.88               0      1    0        1      0                0        0
##   17.43               0      0    0        0      0                0        0
##   17.98               0      0    1        2      0                0        0
##   18.59               0      0    1        1      0                0        0
##   19.25               0      0    0        3      0                0        0
##   19.98               0      0    0        3      0                0        0
##   20.73               0      2    0        0      0                1        0
##   21.54               1      0    0        1      1                1        1
##   22.35               0      0    0        2      0                0        0
##   23.21               0      1    0        1      0                0        0
##   24.11               0      1    1        1      0                0        0
##   25.03               0      0    0        0      0                0        0
##   26.02               0      0    0        0      0                0        0
##   27.03               0      0    0        0      0                0        1
##   28.13               0      0    0        0      0                0        0
##   29.31               0      0    0        0      0                0        0
##   30.7                0      0    0        0      0                0        0
##   32.55               0      0    0        0      0                0        0
##   35                  0      0    0        0      0                0        0
##
##         Peru Philippines Poland Portugal Puerto Rico Qatar Romania Russia
##   7        1           0      0        0           0     0       1      0
##   9.51     0           0      0        0           0     0       0      0
##   11.25    0           0      0        0           0     0       0      0
##   12.4     0           0      0        0           0     0       0      0
##   13.33    0           0      0        0           0     0       0      0
##   14.08    2           1      0        0           0     0       0      0
##   14.75    2           0      0        0           0     0       0      0
##   15.32    4           0      0        0           0     0       1      0
##   15.84    9           0      0        0           0     0       0      0
##   16.36    9           0      1        0           0     0       1      2
##   16.88    8           1      0        0           0     0       1      0
##   17.43   19           0      0        1           0     0       0      2
##   17.98   25           1      0        1           0     0       1      1
##   18.59   42           0      0        0           0     0       0      1
##   19.25   55           1      2        1           0     0       0      0
##   19.98   30           0      0        1           0     1       1      3
##   20.73   42           2      0        0           0     1       5      5
##   21.54   46           3      0        0           0     1       1      0
##   22.35   49           2      2        0           1     0       2      0
##   23.21   55           0      1        0           0     0       0      1
##   24.11   54           0      0        2           1     1       0      0
##   25.03   49           0      0        0           0     0       1      0
##   26.02   49           0      1        0           0     0       0      1
##   27.03   42           0      1        1           0     0       0      0
##   28.13   33           0      0        0           0     0       0      0
##   29.31   30           0      0        0           0     0       0      0
##   30.7    21           0      0        0           0     0       0      0
##   32.55   20           0      1        0           0     0       0      0
##   35      28           0      0        0           0     0       0      1
##
##         Saudi Arabia Senegal Serbia Singapore Slovakia Slovenia Somalia
```

```
##    7                0       0      0          0         0         0       0
##    9.51             0       0      0          0         0         0       0
##    11.25            0       0      0          0         0         0       0
##    12.4             0       0      0          0         0         0       0
##    13.33            0       0      0          0         0         0       0
##    14.08            0       0      0          0         0         0       0
##    14.75            0       0      0          0         0         0       0
##    15.32            0       0      0          0         0         0       0
##    15.84            1       0      0          0         0         0       0
##    16.36            0       0      0          0         0         0       0
##    16.88            0       0      1          1         0         1       0
##    17.43            0       0      0          0         0         0       0
##    17.98            0       0      0          1         1         0       0
##    18.59            1       0      0          1         0         1       0
##    19.25            1       0      0          1         0         0       0
##    19.98            1       0      0          1         0         0       0
##    20.73            1       0      0          1         0         0       0
##    21.54            0       0      0          0         0         2       0
##    22.35            0       0      1          0         0         0       1
##    23.21            1       0      0          1         0         1       0
##    24.11            0       0      0          1         0         0       0
##    25.03            0       1      0          0         0         0       0
##    26.02            0       0      0          0         0         0       0
##    27.03            0       0      0          0         0         0       0
##    28.13            0       0      0          0         0         1       0
##    29.31            0       0      0          1         0         0       0
##    30.7             1       0      0          0         0         0       0
##    32.55            0       0      0          0         0         0       0
##    35               1       0      0          0         0         0       0
##
##          South Africa South Korea Spain Sri Lanka Sudan Suriname Sweden
##    7                0           0     0         0     0        0      0
##    9.51             0           0     0         0     0        0      0
##    11.25            0           0     0         0     0        0      1
##    12.4             0           0     0         0     0        0      0
##    13.33            0           0     0         0     0        0      0
##    14.08            0           0     1         0     0        0      2
##    14.75            0           0     0         0     0        0      3
##    15.32            0           0     1         0     0        0      4
##    15.84            0           0     1         1     0        0      3
##    16.36            1           0     2         2     0        0      5
##    16.88            0           0     1         0     0        0      7
##    17.43            0           0     1         0     0        0      7
##    17.98            1           0     5         0     0        0      3
##    18.59            1           2     3         0     0        0      7
##    19.25            1           0    11         0     0        0     12
##    19.98            1           0     2         0     0        0      8
##    20.73            1           0     2         0     0        0      6
##    21.54            2           0     7         0     0        0      8
##    22.35            2           0     8         0     0        0      9
##    23.21            1           0     7         0     0        0     15
##    24.11            1           0     4         1     0        0     12
##    25.03            0           0     8         0     1        0      8
##    26.02            1           1     2         0     0        0      5
```

```
##   27.03            0           0     2         0     0        0      5
##   28.13            0           0     3         0     0        1      3
##   29.31            0           0     2         0     0        0      3
##   30.7             0           0     1         0     0        0      1
##   32.55            1           0     1         0     0        0      0
##   35               0           0     0         0     0        0      1
##
##         Switzerland Syria Taiwan Thailand Trinidad and Tobago Tunisia Turkey
##   7               0     0      0        0                   0       0      4
##   9.51            0     0      0        0                   0       0      1
##   11.25           0     0      0        0                   0       0      2
##   12.4            0     0      0        0                   0       1      2
##   13.33           0     0      0        0                   0       0      8
##   14.08           0     0      0        0                   0       0     14
##   14.75           0     0      0        0                   0       0     20
##   15.32           0     0      0        1                   0       1     24
##   15.84           0     0      0        0                   0       0     17
##   16.36           1     0      0        0                   0       0     34
##   16.88           0     0      0        0                   0       0     39
##   17.43           1     0      1        1                   0       0     45
##   17.98           1     0      0        0                   0       0     58
##   18.59           2     0      1        0                   0       0     59
##   19.25           2     0      0        0                   0       0     69
##   19.98           3     0      0        0                   0       0     84
##   20.73           0     0      0        2                   1       0     74
##   21.54           3     0      0        1                   0       0     94
##   22.35           2     0      0        0                   0       0     95
##   23.21           2     0      0        1                   0       0     94
##   24.11           1     0      0        1                   0       0     63
##   25.03           2     0      0        0                   0       1     91
##   26.02           1     0      0        1                   0       0     45
##   27.03           0     1      0        1                   0       0     29
##   28.13           1     0      0        0                   0       0     27
##   29.31           0     0      0        0                   0       0     23
##   30.7            0     0      0        2                   0       0     15
##   32.55           0     1      0        0                   0       0      8
##   35              0     0      1        0                   0       0     10
##
##         UAE Uganda  UK Ukraine Uruguay USA Vietnam Zambia
##   7       0      0   8       0       0   5       0      0
##   9.51    0      0   4       0       0   1       0      0
##   11.25   0      0   8       0       0   6       0      0
##   12.4    0      0  11       0       0   3       1      0
##   13.33   0      0  10       0       0   5       0      0
##   14.08   0      0  13       0       0   5       0      0
##   14.75   1      0  25       0       0  10       0      0
##   15.32   0      0  30       0       0  19       0      0
##   15.84   1      0  36       0       0  15       0      0
##   16.36   0      0  61       1       0  15       0      1
##   16.88   0      0  54       0       0  17       0      0
##   17.43   0      0  82       0       0  33       0      0
##   17.98   0      0 101       0       1  30       0      0
##   18.59   1      1 139       0       0  46       0      0
##   19.25   2      0 133       1       0  39       0      0
```

```
##   19.98   4      1 135      0       0  36       0      1
##   20.73   1      2 160      0       0  37       0      0
##   21.54   3      0 156      0       0  47       0      0
##   22.35   4      1 140      0       1  28       0      0
##   23.21   4      0 165      0       0  40       0      0
##   24.11   2      1 129      0       0  35       0      0
##   25.03   0      0 127      0       0  29       0      0
##   26.02   1      0  69      0       1   9       0      0
##   27.03   1      0  36      0       0   7       0      0
##   28.13   1      0  34      0       0   9       0      0
##   29.31   0      0  26      0       0   7       0      0
##   30.7    1      0  21      0       0   3       0      0
##   32.55   0      1  13      0       0   2       0      0
##   35      0      0  11      0       0   5       0      0
```

Main Analyses: Complete outputs of models reported in the paper

Study hypotheses

```
# Hypothesis 1a: Bonding (vs no bonding) will positively predict engagement in health behaviours.
# Hypothesis 1b: Bonding with multiple groups will positively predict engagement in health behaviours.
# Hypothesis 2a: Bonding (vs no bonding)  will positively predict mental health and wellbeing
# Hypothesis 2b: Bonding with multiple groups will positively predict mental health and wellbeing.
```

Table S5A: Hypothesis 1a, Dataset A: Does bonding (vs no bonding) predict health behaviours?

Hypothesis 1a, Dataset A models

```
# Distancing
summary(bond_distancing_A<- lme(Pro_Distancing_scaled ~ bond_family + bond_friend +
                                 bond_country + bond_humanity  +
                                demo_age + demo_gender + demo_education + GDP,
                                (~1|Country_Name), data=DatasetA, na.action=na.exclude))
```

```
## Linear mixed-effects model fit by REML
##   Data: DatasetA
##        AIC     BIC    logLik
##   18368.74 18463.7 -9170.369
##
## Random effects:
##  Formula: ~1 | Country_Name
##         (Intercept)  Residual
## StdDev:   0.8367055 0.9697492
##
## Fixed effects:  Pro_Distancing_scaled ~ bond_family + bond_friend + bond_country +      bond_humanit
##                                 Value  Std.Error   DF   t-value p-value
## (Intercept)                -0.5447652 0.17724938 6452 -3.073439  0.0021
```

```
## bond_family1                  0.0879132 0.02951936 6452  2.978153  0.0029
## bond_friend1                 -0.0449372 0.04342510 6452 -1.034821  0.3008
## bond_country1                 0.0194740 0.04305131 6452  0.452344  0.6510
## bond_humanity1                0.0408196 0.03280964 6452  1.244134  0.2135
## demo_age                      0.0008720 0.00098685 6452  0.883579  0.3770
## demo_genderFemale             0.1555361 0.02530527 6452  6.146392  0.0000
## demo_genderOther/non-binary   0.0408152 0.12900480 6452  0.316385  0.7517
## demo_educationundergraduate   0.1176155 0.03061402 6452  3.841883  0.0001
## demo_educationpostgraduate    0.0900160 0.03336625 6452  2.697814  0.0070
## demo_educationNA             -0.1357636 0.14552114 6452 -0.932947  0.3509
## GDP                          -0.0000008 0.00000406   69 -0.186456  0.8526
##  Correlation:
##                              (Intr) bnd_fm1 bnd_fr1 bnd_c1 bnd_h1 demo_g dm_gnF
## bond_family1                 -0.005
## bond_friend1                  0.006 -0.235
## bond_country1                -0.009 -0.086  -0.088
## bond_humanity1               -0.023  0.004  -0.064  -0.296
## demo_age                     -0.176 -0.179  -0.013  -0.072 -0.012
## demo_genderFemale            -0.069 -0.060  -0.061   0.018 -0.017 -0.021
## demo_genderOther/non-binary -0.011  0.020  -0.022   0.005 -0.018  0.032  0.113
## demo_educationundergraduate -0.085  0.056  -0.006   0.012 -0.028 -0.079 -0.020
## demo_educationpostgraduate  -0.069  0.033   0.035   0.029 -0.060 -0.169 -0.042
## demo_educationNA             -0.037 -0.008  -0.041   0.007 -0.027 -0.019 -0.011
## GDP                          -0.698  0.007  -0.010   0.004  0.010  0.001 -0.001
##                              dm_O/- dm_dctnn dm_dctnp dm_dNA
## bond_family1
## bond_friend1
## bond_country1
## bond_humanity1
## demo_age
## demo_genderFemale
## demo_genderOther/non-binary
## demo_educationundergraduate  0.018
## demo_educationpostgraduate   0.009  0.582
## demo_educationNA              0.007  0.120    0.117
## GDP                          -0.031 -0.010   -0.009    0.012
##
## Standardized Within-Group Residuals:
##        Min          Q1         Med          Q3         Max
## -6.7366096 -0.2038657  0.3441812  0.5714576  2.5019374
##
## Number of Observations: 6533
## Number of Groups: 71
```

```
anova(bond_distancing_A,type="marginal")
```

```
##                 numDF denDF   F-value p-value
## (Intercept)         1  6452  9.446028  0.0021
## bond_family         1  6452  8.869393  0.0029
## bond_friend         1  6452  1.070856  0.3008
## bond_country        1  6452  0.204615  0.6510
## bond_humanity       1  6452  1.547870  0.2135
## demo_age            1  6452  0.780712  0.3770
## demo_gender         2  6452 18.961603  <.0001
```

```
## demo_education     3  6452  5.720112  0.0007
## GDP                1    69  0.034766  0.8526
```

```r
r2_nakagawa(bond_distancing_A)
```

```
## # R2 for Mixed Models
##
##   Conditional R2: 0.431
##      Marginal R2: 0.007
```

```r
eta_squared(bond_distancing_A)
```

```
## # Effect Size for ANOVA
##
## Parameter      | Eta2 (partial) |       95% CI
## ----------------------------------------------
## bond_family    |       1.95e-03 | [0.00, 1.00]
## bond_friend    |       4.51e-05 | [0.00, 1.00]
## bond_country   |       1.16e-04 | [0.00, 1.00]
## bond_humanity  |       3.51e-04 | [0.00, 1.00]
## demo_age       |       3.43e-04 | [0.00, 1.00]
## demo_gender    |       6.03e-03 | [0.00, 1.00]
## demo_education |       2.65e-03 | [0.00, 1.00]
## GDP            |       5.04e-04 | [0.00, 1.00]
##
## - One-sided CIs: upper bound fixed at [1.00].
```

```r
# Hygiene
summary(bond_hygiene_A<- lme(Pro_Hygiene_scaled ~ bond_family + bond_friend +
                                 bond_country + bond_humanity  +
                                demo_age + demo_gender + demo_education + GDP,
                                (~1|Country_Name), data=DatasetA, na.action=na.exclude))
```

```
## Linear mixed-effects model fit by REML
##   Data: DatasetA
##       AIC      BIC    logLik
##   17897.8 17992.72 -8934.898
##
## Random effects:
##  Formula: ~1 | Country_Name
##         (Intercept)  Residual
## StdDev:   0.2212911 0.9444003
##
## Fixed effects:  Pro_Hygiene_scaled ~ bond_family + bond_friend + bond_country +      bond_humanity +
##                                 Value  Std.Error   DF   t-value p-value
## (Intercept)                -0.2997817 0.09489681 6436 -3.159028  0.0016
## bond_family1                0.3103720 0.02870762 6436 10.811486  0.0000
## bond_friend1                0.0653228 0.04229642 6436  1.544404  0.1225
## bond_country1              -0.0208586 0.04182196 6436 -0.498747  0.6180
## bond_humanity1              0.0297216 0.03188855 6436  0.932047  0.3513
## demo_age                    0.0049207 0.00095843 6436  5.134140  0.0000
## demo_genderFemale           0.2616766 0.02458592 6436 10.643352  0.0000
```

```
## demo_genderOther/non-binary -0.0446892 0.12495058 6436 -0.357655  0.7206
## demo_educationundergraduate -0.0119032 0.02979803 6436 -0.399463  0.6896
## demo_educationpostgraduate  -0.0966228 0.03243420 6436 -2.979040  0.0029
## demo_educationNA             0.3369301 0.14049778 6436  2.398117  0.0165
## GDP                         -0.0000048 0.00000199   70 -2.433356  0.0175
##  Correlation:
##                             (Intr) bnd_fm1 bnd_fr1 bnd_c1 bnd_h1 demo_g dm_gnF
## bond_family1                -0.018
## bond_friend1                 0.007 -0.234
## bond_country1               -0.012 -0.087  -0.089
## bond_humanity1              -0.042  0.005  -0.065  -0.296
## demo_age                    -0.322 -0.182  -0.013  -0.073 -0.014
## demo_genderFemale           -0.117 -0.060  -0.061   0.018 -0.017 -0.021
## demo_genderOther/non-binary -0.027  0.020  -0.022   0.006 -0.018  0.033  0.113
## demo_educationundergraduate -0.162  0.055  -0.006   0.011 -0.028 -0.080 -0.023
## demo_educationpostgraduate  -0.124  0.034   0.034   0.030 -0.060 -0.168 -0.045
## demo_educationNA            -0.051 -0.008  -0.040   0.007 -0.025 -0.018 -0.012
## GDP                         -0.747  0.025  -0.014   0.005  0.022 -0.004 -0.012
##                             dm_O/- dm_dctnn dm_dctnp dm_dNA
## bond_family1
## bond_friend1
## bond_country1
## bond_humanity1
## demo_age
## demo_genderFemale
## demo_genderOther/non-binary
## demo_educationundergraduate  0.017
## demo_educationpostgraduate   0.008  0.583
## demo_educationNA             0.007  0.121    0.117
## GDP                         -0.034 -0.012   -0.012    0.016
##
## Standardized Within-Group Residuals:
##        Min         Q1        Med         Q3        Max
## -3.5243607 -0.6445210  0.0642938  0.7085250  2.5584225
##
## Number of Observations: 6518
## Number of Groups: 72
```

```
anova.lme(bond_hygiene_A,type="marginal")
```

```
##               numDF denDF   F-value p-value
## (Intercept)       1  6436   9.97946  0.0016
## bond_family       1  6436 116.88823  <.0001
## bond_friend       1  6436   2.38518  0.1225
## bond_country      1  6436   0.24875  0.6180
## bond_humanity     1  6436   0.86871  0.3513
## demo_age          1  6436  26.35940  <.0001
## demo_gender       2  6436  57.86987  <.0001
## demo_education    3  6436   6.22958  0.0003
## GDP               1    70   5.92122  0.0175
```

```
r2_nakagawa(bond_hygiene_A)
```

```
## # R2 for Mixed Models
##
##   Conditional R2: 0.110
##      Marginal R2: 0.062
```

```
eta_squared(bond_hygiene_A)
```

```
## # Effect Size for ANOVA
##
## Parameter      | Eta2 (partial) |       95% CI
## -----------------------------------------------
## bond_family    |           0.03 | [0.02, 1.00]
## bond_friend    |       1.00e-03 | [0.00, 1.00]
## bond_country   |       1.47e-06 | [0.00, 1.00]
## bond_humanity  |       2.04e-04 | [0.00, 1.00]
## demo_age       |       3.90e-03 | [0.00, 1.00]
## demo_gender    |           0.02 | [0.01, 1.00]
## demo_education |       2.95e-03 | [0.00, 1.00]
## GDP            |           0.08 | [0.01, 1.00]
##
## - One-sided CIs: upper bound fixed at [1.00].
```

```
# Masking
summary(bond_masking_A<- lme(Pro_MaskWearing_scaled ~ bond_family + bond_friend +
                                 bond_country + bond_humanity  +
                                demo_age + demo_gender + demo_education + GDP,
                                (~1|Country_Name), data=DatasetA, na.action=na.exclude))
```

```
## Linear mixed-effects model fit by REML
##   Data: DatasetA
##        AIC      BIC    logLik
##   17761.68 17856.62 -8866.842
##
## Random effects:
##  Formula: ~1 | Country_Name
##         (Intercept)  Residual
## StdDev:   0.4316374 0.9308937
##
## Fixed effects:  Pro_MaskWearing_scaled ~ bond_family + bond_friend + bond_country +      bond_humani
##                                 Value  Std.Error   DF   t-value p-value
## (Intercept)                 0.1034552 0.12255446 6443  0.844157  0.3986
## bond_family1                0.1243062 0.02832205 6443  4.389027  0.0000
## bond_friend1               -0.0316124 0.04169114 6443 -0.758252  0.4483
## bond_country1              -0.0005623 0.04136204 6443 -0.013595  0.9892
## bond_humanity1              0.0477578 0.03148127 6443  1.517022  0.1293
## demo_age                    0.0023085 0.00094755 6443  2.436253  0.0149
## demo_genderFemale           0.0411331 0.02427063 6443  1.694769  0.0902
## demo_genderOther/non-binary  0.0098005 0.12454986 6443  0.078687  0.9373
## demo_educationundergraduate  0.0194798 0.02938726 6443  0.662867  0.5074
## demo_educationpostgraduate  -0.0584236 0.03203657 6443 -1.823654  0.0683
## demo_educationNA            0.3448342 0.13901338 6443  2.480583  0.0131
## GDP                        -0.0000054 0.00000268   69 -1.997442  0.0497
##  Correlation:
```

```
##                              (Intr) bnd_fm1 bnd_fr1 bnd_c1 bnd_h1 demo_g dm_gnF
## bond_family1                 -0.012
## bond_friend1                  0.007 -0.235
## bond_country1                -0.011 -0.088  -0.088
## bond_humanity1               -0.032  0.005  -0.064  -0.298
## demo_age                     -0.242 -0.180  -0.013  -0.072 -0.013
## demo_genderFemale            -0.096 -0.059  -0.061   0.017 -0.016 -0.021
## demo_genderOther/non-binary -0.017  0.020  -0.022   0.006 -0.018  0.032  0.112
## demo_educationundergraduate -0.123  0.056  -0.006   0.012 -0.028 -0.080 -0.020
## demo_educationpostgraduate   -0.099  0.034   0.035   0.029 -0.060 -0.170 -0.043
## demo_educationNA             -0.048 -0.008  -0.041   0.007 -0.026 -0.019 -0.011
## GDP                          -0.725  0.015  -0.014   0.005  0.015 -0.002 -0.003
##                              dm_O/- dm_dctnn dm_dctnp dm_dNA
## bond_family1
## bond_friend1
## bond_country1
## bond_humanity1
## demo_age
## demo_genderFemale
## demo_genderOther/non-binary
## demo_educationundergraduate  0.020
## demo_educationpostgraduate   0.009  0.583
## demo_educationNA             0.007  0.121    0.117
## GDP                          -0.036 -0.010   -0.010    0.017
##
## Standardized Within-Group Residuals:
##        Min         Q1        Med         Q3        Max
## -1.7935243 -0.6649986 -0.2866702  0.5679022  3.3278912
##
## Number of Observations: 6524
## Number of Groups: 71
```

```
anova.lme(bond_masking_A,type="marginal")
```

```
##                 numDF denDF   F-value p-value
## (Intercept)         1  6443  0.712601  0.3986
## bond_family         1  6443 19.263561  <.0001
## bond_friend         1  6443  0.574947  0.4483
## bond_country        1  6443  0.000185  0.9892
## bond_humanity       1  6443  2.301354  0.1293
## demo_age            1  6443  5.935327  0.0149
## demo_gender         2  6443  1.442343  0.2364
## demo_education      3  6443  4.829597  0.0023
## GDP                 1    69  3.989773  0.0497
```

```
r2_nakagawa(bond_masking_A)
```

```
## # R2 for Mixed Models
##
##   Conditional R2: 0.191
##      Marginal R2: 0.018
```

```r
eta_squared(bond_masking_A)
```

```
## # Effect Size for ANOVA
##
## Parameter      | Eta2 (partial) |       95% CI
## -----------------------------------------------
## bond_family    |       4.23e-03 | [0.00, 1.00]
## bond_friend    |       1.07e-05 | [0.00, 1.00]
## bond_country   |       6.92e-05 | [0.00, 1.00]
## bond_humanity  |       3.72e-04 | [0.00, 1.00]
## demo_age       |       7.25e-04 | [0.00, 1.00]
## demo_gender    |       4.11e-04 | [0.00, 1.00]
## demo_education |       2.28e-03 | [0.00, 1.00]
## GDP            |           0.05 | [0.00, 1.00]
##
## - One-sided CIs: upper bound fixed at [1.00].
```

Hypothesis 1a, Dataset A model check for assumptions and fit

```r
# Test homogeneity of variance for the bonding variables and models with/out random effect
# Distancing
summary(bond_distancing_A_noRand<- lm(Pro_Distancing_scaled ~ bond_family + bond_friend +
                                 bond_country + bond_humanity  +
                                demo_age + demo_gender + demo_education + GDP,
                                data=DatasetA, na.action=na.exclude))
```

```
##
## Call:
## lm(formula = Pro_Distancing_scaled ~ bond_family + bond_friend +
##     bond_country + bond_humanity + demo_age + demo_gender + demo_education +
##     GDP, data = DatasetA, na.action = na.exclude)
##
## Residuals:
##     Min      1Q  Median      3Q     Max
## -6.7615 -0.1427  0.4325  0.5740  1.0301
##
## Coefficients:
##                               Estimate Std. Error t value Pr(>|t|)
## (Intercept)                 -2.738e-01  4.946e-02  -5.536 3.21e-08 ***
## bond_family1                 7.114e-02  2.987e-02   2.382 0.017255 *
## bond_friend1                -4.371e-02  4.411e-02  -0.991 0.321750
## bond_country1               -1.986e-02  4.299e-02  -0.462 0.644189
## bond_humanity1               5.630e-02  3.317e-02   1.697 0.089710 .
## demo_age                    -1.066e-03  9.607e-04  -1.110 0.267208
## demo_genderFemale            1.457e-01  2.549e-02   5.717 1.13e-08 ***
## demo_genderOther/non-binary  3.907e-02  1.306e-01   0.299 0.764900
## demo_educationundergraduate  1.519e-01  3.045e-02   4.988 6.27e-07 ***
## demo_educationpostgraduate   1.246e-01  3.330e-02   3.743 0.000184 ***
## demo_educationNA            -1.845e-01  1.467e-01  -1.258 0.208560
## GDP                          2.567e-06  6.503e-07   3.947 7.98e-05 ***
## ---
```

```
## Signif. codes:  0 '***' 0.001 '**' 0.01 '*' 0.05 '.' 0.1 ' ' 1
##
## Residual standard error: 0.9901 on 6521 degrees of freedom
##   (56 observations deleted due to missingness)
## Multiple R-squared:  0.01415,    Adjusted R-squared:  0.01249
## F-statistic: 8.511 on 11 and 6521 DF,  p-value: 4.27e-15
```

```
bond_distancing_A<- lme(Pro_Distancing_scaled ~ bond_family + bond_friend +
                                 bond_country + bond_humanity  +
                                demo_age + demo_gender + demo_education + GDP,
                                (~1|Country_Name), data=DatasetA, na.action=na.exclude)
anova(bond_distancing_A, bond_distancing_A_noRand)
```

```
##                          Model df      AIC      BIC    logLik   Test  L.Ratio
## bond_distancing_A            1 14 18368.74 18463.70 -9170.369
## bond_distancing_A_noRand     2 13 18509.09 18597.26 -9241.543 1 vs 2 142.3469
##                          p-value
## bond_distancing_A
## bond_distancing_A_noRand  <.0001
```

```
leveneTest(residuals(bond_distancing_A) ~ DatasetA$bond_family)
```

```
## Levene's Test for Homogeneity of Variance (center = median)
##         Df F value Pr(>F)
## group    1  0.5295 0.4669
##       6531
```

```
leveneTest(residuals(bond_distancing_A) ~ DatasetA$bond_friend)
```

```
## Levene's Test for Homogeneity of Variance (center = median)
##         Df F value Pr(>F)
## group    1   1.622 0.2029
##       6531
```

```
leveneTest(residuals(bond_distancing_A) ~ DatasetA$bond_country)
```

```
## Levene's Test for Homogeneity of Variance (center = median)
##         Df F value Pr(>F)
## group    1   0.779 0.3775
##       6531
```

```
leveneTest(residuals(bond_distancing_A) ~ DatasetA$bond_humanity)
```

```
## Levene's Test for Homogeneity of Variance (center = median)
##         Df F value Pr(>F)
## group    1  0.7545 0.3851
##       6531
```

```r
# Hygiene
summary(bond_hygiene_A_noRand<- lm(Pro_Hygiene_scaled ~ bond_family + bond_friend +
                                 bond_country + bond_humanity  +
                                demo_age + demo_gender + demo_education + GDP,
                            data=DatasetA, na.action=na.exclude))
```

```
##
## Call:
## lm(formula = Pro_Hygiene_scaled ~ bond_family + bond_friend +
##     bond_country + bond_humanity + demo_age + demo_gender + demo_education +
##     GDP, data = DatasetA, na.action = na.exclude)
##
## Residuals:
##     Min      1Q  Median      3Q     Max
## -3.3234 -0.6293  0.0655  0.7100  2.1246
##
## Coefficients:
##                              Estimate Std. Error t value Pr(>|t|)
## (Intercept)                -3.584e-01  4.897e-02  -7.319 2.81e-13 ***
## bond_family1                2.954e-01  2.958e-02   9.988  < 2e-16 ***
## bond_friend1                7.491e-02  4.371e-02   1.714   0.0866 .
## bond_country1              -8.491e-02  4.255e-02  -1.996   0.0460 *
## bond_humanity1              5.847e-02  3.280e-02   1.782   0.0747 .
## demo_age                    1.728e-03  9.520e-04   1.815   0.0696 .
## demo_genderFemale           2.494e-01  2.524e-02   9.881  < 2e-16 ***
## demo_genderOther/non-binary -4.581e-02  1.292e-01  -0.355   0.7229
## demo_educationundergraduate  4.531e-02  3.017e-02   1.502   0.1332
## demo_educationpostgraduate  -5.906e-02  3.297e-02  -1.791   0.0733 .
## demo_educationNA            2.373e-01  1.451e-01   1.635   0.1020
## GDP                         1.344e-06  6.434e-07   2.089   0.0368 *
## ---
## Signif. codes:  0 '***' 0.001 '**' 0.01 '*' 0.05 '.' 0.1 ' ' 1
##
## Residual standard error: 0.9791 on 6506 degrees of freedom
##   (71 observations deleted due to missingness)
## Multiple R-squared:  0.04329,    Adjusted R-squared:  0.04167
## F-statistic: 26.76 on 11 and 6506 DF,  p-value: < 2.2e-16
```

```r
bond_hygiene_A<- lme(Pro_Hygiene_scaled ~ bond_family + bond_friend +
                                 bond_country + bond_humanity  +
                                demo_age + demo_gender + demo_education + GDP,
                                (~1|Country_Name), data=DatasetA, na.action=na.exclude)
anova(bond_hygiene_A, bond_hygiene_A_noRand)
```

```
##                       Model df      AIC      BIC    logLik   Test  L.Ratio
## bond_hygiene_A            1 14 17897.79 17992.72 -8934.898
## bond_hygiene_A_noRand     2 13 18321.12 18409.26 -9147.559 1 vs 2 425.3231
##                       p-value
## bond_hygiene_A
## bond_hygiene_A_noRand  <.0001
```

```r
leveneTest(residuals(bond_hygiene_A) ~ DatasetA$bond_family)
```

```
## Levene's Test for Homogeneity of Variance (center = median)
##         Df F value Pr(>F)
## group    1  1.6835 0.1945
##       6516
```

```r
leveneTest(residuals(bond_hygiene_A) ~ DatasetA$bond_friend)
```

```
## Levene's Test for Homogeneity of Variance (center = median)
##         Df F value Pr(>F)
## group    1  0.2542 0.6142
##       6516
```

```r
leveneTest(residuals(bond_hygiene_A) ~ DatasetA$bond_country) # sig.
```

```
## Levene's Test for Homogeneity of Variance (center = median)
##         Df F value   Pr(>F)
## group    1  7.5596 0.005986 **
##       6516
## ---
## Signif. codes:  0 '***' 0.001 '**' 0.01 '*' 0.05 '.' 0.1 ' ' 1
```

```r
leveneTest(residuals(bond_hygiene_A) ~ DatasetA$bond_humanity)
```

```
## Levene's Test for Homogeneity of Variance (center = median)
##         Df F value  Pr(>F)
## group    1  2.8237 0.09293 .
##       6516
## ---
## Signif. codes:  0 '***' 0.001 '**' 0.01 '*' 0.05 '.' 0.1 ' ' 1
```

```r
# Masking
summary(bond_masking_A_noRand<- lm(Pro_MaskWearing_scaled ~ bond_family + bond_friend +
                                 bond_country + bond_humanity  +
                                demo_age + demo_gender + demo_education + GDP,
                                data=DatasetA, na.action=na.exclude))
```

```
##
## Call:
## lm(formula = Pro_MaskWearing_scaled ~ bond_family + bond_friend +
##     bond_country + bond_humanity + demo_age + demo_gender + demo_education +
##     GDP, data = DatasetA, na.action = na.exclude)
##
## Residuals:
##     Min      1Q  Median      3Q     Max
## -1.4262 -0.7832 -0.3624  0.6422  2.8260
##
## Coefficients:
##                                 Estimate Std. Error t value Pr(>|t|)
```

```
## (Intercept)                   -4.387e-02  4.937e-02  -0.889 0.374241
## bond_family1                   1.836e-01  2.980e-02   6.161 7.68e-10 ***
## bond_friend1                  -4.669e-02  4.403e-02  -1.060 0.289005
## bond_country1                 -1.100e-01  4.296e-02  -2.560 0.010480 *
## bond_humanity1                 1.351e-01  3.309e-02   4.082 4.51e-05 ***
## demo_age                       4.306e-03  9.605e-04   4.483 7.49e-06 ***
## demo_genderFemale              9.455e-02  2.543e-02   3.718 0.000203 ***
## demo_genderOther/non-binary -9.945e-03  1.313e-01  -0.076 0.939634
## demo_educationundergraduate -1.124e-01  3.040e-02  -3.698 0.000219 ***
## demo_educationpostgraduate  -1.706e-01  3.324e-02  -5.131 2.96e-07 ***
## demo_educationNA               3.991e-01  1.463e-01   2.728 0.006392 **
## GDP                           -3.344e-06  6.483e-07  -5.158 2.57e-07 ***
## ---
## Signif. codes:  0 '***' 0.001 '**' 0.01 '*' 0.05 '.' 0.1 ' ' 1
##
## Residual standard error: 0.987 on 6512 degrees of freedom
##   (65 observations deleted due to missingness)
## Multiple R-squared:  0.02692,    Adjusted R-squared:  0.02528
## F-statistic: 16.38 on 11 and 6512 DF,  p-value: < 2.2e-16
```

```
bond_masking_A<- lme(Pro_MaskWearing_scaled ~ bond_family + bond_friend +
                                 bond_country + bond_humanity  +
                                demo_age + demo_gender + demo_education + GDP,
                                (~1|Country_Name), data=DatasetA, na.action=na.exclude)
anova(bond_masking_A, bond_masking_A_noRand)
```

```
##                       Model df      AIC      BIC    logLik   Test  L.Ratio
## bond_masking_A            1 14 17761.68 17856.62 -8866.842
## bond_masking_A_noRand     2 13 18443.60 18531.76 -9208.800 1 vs 2 683.9166
##                       p-value
## bond_masking_A
## bond_masking_A_noRand  <.0001
```

```
leveneTest(residuals(bond_masking_A) ~ DatasetA$bond_family) # sig.
```

```
## Levene's Test for Homogeneity of Variance (center = median)
##         Df F value   Pr(>F)
## group    1   26.69 2.46e-07 ***
##       6522
## ---
## Signif. codes:  0 '***' 0.001 '**' 0.01 '*' 0.05 '.' 0.1 ' ' 1
```

```
leveneTest(residuals(bond_masking_A) ~ DatasetA$bond_friend)
```

```
## Levene's Test for Homogeneity of Variance (center = median)
##         Df F value Pr(>F)
## group    1  3.5524 0.0595 .
##       6522
## ---
## Signif. codes:  0 '***' 0.001 '**' 0.01 '*' 0.05 '.' 0.1 ' ' 1
```

```r
leveneTest(residuals(bond_masking_A) ~ DatasetA$bond_country)
```

```
## Levene's Test for Homogeneity of Variance (center = median)
##         Df F value Pr(>F)
## group    1  0.3641 0.5462
##       6522
```

```r
leveneTest(residuals(bond_masking_A) ~ DatasetA$bond_humanity) # sig.
```

```
## Levene's Test for Homogeneity of Variance (center = median)
##         Df F value    Pr(>F)
## group    1  12.962 0.0003202 ***
##       6522
## ---
## Signif. codes:  0 '***' 0.001 '**' 0.01 '*' 0.05 '.' 0.1 ' ' 1
```

Table S5B: Hypothesis 1a, Dataset B: Does bonding (vs no bonding) predict health behaviours?

Hypothesis 1a, Dataset B model

```r
# Distancing
summary(bond_distancing_B <- lme(comply_self_scale ~ bond_country + bond_gvmt  +
                                   age + gender + education + GDP,
                                     random=list(country_now_Name=pdDiag(form=~ bond_country)),
                                     weights=varIdent(form=~ 1|bond_country),
                                     data=DatasetB_phase1, na.action=na.exclude, method="REML"))
```

```
## Linear mixed-effects model fit by REML
##   Data: DatasetB_phase1
##        AIC      BIC    logLik
##   17562.52 17656.92 -8767.258
##
## Random effects:
##  Formula: ~bond_country | country_now_Name
##  Structure: Diagonal
##         (Intercept) bond_country1  Residual
## StdDev:   0.1621149    0.07508225 0.9374759
##
## Variance function:
##  Structure: Different standard deviations per stratum
##  Formula: ~1 | bond_country
##  Parameter estimates:
##       1       0
## 1.00000 1.04313
## Fixed effects:  comply_self_scale ~ bond_country + bond_gvmt + age + gender +      education + GDP
##                              Value  Std.Error   DF   t-value p-value
## (Intercept)            -0.24931177 0.06288330 6164 -3.964674  0.0001
## bond_country1          -0.01175592 0.04023832 6164 -0.292157  0.7702
## bond_gvmt1              0.08327983 0.05238559 6164  1.589747  0.1119
```

```
## age                         0.00108244 0.00096721 6164  1.119136  0.2631
## genderwoman                 0.06408049 0.02755035 6164  2.325941  0.0201
## gendernb                    0.06288756 0.13181553 6164  0.477088  0.6333
## gendernone                  0.26645158 0.14875243 6164  1.791242  0.0733
## educationundergraduate      0.05346938 0.03644172 6164  1.467257  0.1424
## educationpostgraduate       0.10830740 0.03916621 6164  2.765327  0.0057
## GDP                         0.00000378 0.00000130  105  2.908153  0.0044
##  Correlation:
##                        (Intr) bnd_c1 bnd_g1 age    gndrwm gndrnb gndrnn edctnn
## bond_country1          -0.083
## bond_gvmt1             -0.037 -0.303
## age                    -0.381 -0.142 -0.007
## genderwoman            -0.260 -0.023  0.037  0.084
## gendernb               -0.076  0.008  0.019  0.054  0.151
## gendernone             -0.069  0.015 -0.013  0.030  0.124  0.029
## educationundergraduate -0.354  0.005  0.011 -0.180 -0.028 -0.004  0.005
## educationpostgraduate  -0.306  0.035  0.007 -0.253 -0.045 -0.003  0.007  0.722
## GDP                    -0.494  0.062 -0.022 -0.085 -0.081 -0.025 -0.004  0.020
##                        edctnp
## bond_country1
## bond_gvmt1
## age
## genderwoman
## gendernb
## gendernone
## educationundergraduate
## educationpostgraduate
## GDP                    -0.009
##
## Standardized Within-Group Residuals:
##        Min         Q1        Med         Q3        Max
## -3.1126430 -0.6832448 -0.3039266  0.7687691  2.1395836
##
## Number of Observations: 6279
## Number of Groups: 107
```

```
anova.lme(bond_distancing_B,type="marginal")
```

```
##             numDF denDF   F-value p-value
## (Intercept)     1  6164 15.718637  0.0001
## bond_country    1  6164  0.085356  0.7702
## bond_gvmt       1  6164  2.527295  0.1119
## age             1  6164  1.252465  0.2631
## gender          3  6164  2.571072  0.0524
## education       2  6164  4.116389  0.0163
## GDP             1   105  8.457351  0.0044
```

```
r2_nakagawa(bond_distancing_B)
```

```
## [1] NA
```

```r
eta_squared(bond_distancing_B)
```

```
## # Effect Size for ANOVA
##
## Parameter    | Eta2 (partial) |       95% CI
## ---------------------------------------------
## bond_country |       4.56e-06 | [0.00, 1.00]
## bond_gvmt    |       4.09e-04 | [0.00, 1.00]
## age          |       6.11e-04 | [0.00, 1.00]
## gender       |       1.54e-03 | [0.00, 1.00]
## education    |       1.39e-03 | [0.00, 1.00]
## GDP          |           0.07 | [0.01, 1.00]
##
## - One-sided CIs: upper bound fixed at [1.00].
```

Hypothesis 1a, Dataset B model check for assumptions and fit

```r
# Test homogeneity of variance for the bonding variables
summary(bond_distancing_B_noRand<- lm(comply_self_scale ~ bond_country + bond_gvmt  +
                                   age + gender + education + GDP,
                               data=DatasetB_phase1, na.action=na.exclude, method="REML"))
```

```
##
## Call:
## lm(formula = comply_self_scale ~ bond_country + bond_gvmt + age +
##     gender + education + GDP, data = DatasetB_phase1, na.action = na.exclude,
##     method = "REML")
##
## Residuals:
##     Min      1Q  Median      3Q     Max
## -3.0614 -0.6542 -0.3488  0.7498  1.8292
##
## Coefficients:
##                          Estimate Std. Error t value Pr(>|t|)
## (Intercept)            -1.401e-01  4.531e-02  -3.091 0.002004 **
## bond_country1          -7.877e-02  3.315e-02  -2.376 0.017543 *
## bond_gvmt1              6.140e-02  5.341e-02   1.150 0.250321
## age                     3.109e-03  9.337e-04   3.330 0.000873 ***
## genderwoman             5.224e-02  2.733e-02   1.912 0.055972 .
## gendernb                7.159e-02  1.322e-01   0.542 0.588008
## gendernone              2.372e-01  1.496e-01   1.586 0.112779
## educationundergraduate  5.169e-02  3.669e-02   1.409 0.158855
## educationpostgraduate   8.220e-02  3.880e-02   2.118 0.034192 *
## GDP                    -2.910e-07  6.416e-07  -0.454 0.650158
## ---
## Signif. codes:  0 '***' 0.001 '**' 0.01 '*' 0.05 '.' 0.1 ' ' 1
##
## Residual standard error: 0.9802 on 6269 degrees of freedom
##   (396 observations deleted due to missingness)
## Multiple R-squared:  0.004874,   Adjusted R-squared:  0.003445
## F-statistic: 3.412 on 9 and 6269 DF,  p-value: 0.0003404
```

```r
summary(bond_distancing_B<- lme(comply_self_scale ~ bond_country + bond_gvmt  +
                                  age + gender + education + GDP,
                                  (~1|country_now_Name), data=DatasetB_phase1, na.action=na.exclude, me
```

```
## Linear mixed-effects model fit by REML
##   Data: DatasetB_phase1
##        AIC      BIC    logLik
##   17563.3 17644.22 -8769.651
##
## Random effects:
##  Formula: ~1 | country_now_Name
##         (Intercept)  Residual
## StdDev:   0.1614897 0.9697239
##
## Fixed effects:  comply_self_scale ~ bond_country + bond_gvmt + age + gender +      education + GDP
##                              Value  Std.Error   DF   t-value p-value
## (Intercept)             -0.25114339 0.06239619 6164 -4.024980  0.0001
## bond_country1           -0.02293517 0.03359434 6164 -0.682709  0.4948
## bond_gvmt1               0.07936684 0.05348294 6164  1.483965  0.1379
## age                      0.00118503 0.00096415 6164  1.229097  0.2191
## genderwoman              0.06471591 0.02755705 6164  2.348434  0.0189
## gendernb                 0.05975248 0.13112576 6164  0.455688  0.6486
## gendernone               0.25711711 0.14821678 6164  1.734737  0.0828
## educationundergraduate   0.05384261 0.03648754 6164  1.475644  0.1401
## educationpostgraduate    0.10801815 0.03917854 6164  2.757075  0.0058
## GDP                      0.00000380 0.00000129  105  2.935167  0.0041
##  Correlation:
##                         (Intr) bnd_c1 bnd_g1 age    gndrwm gndrnb gndrnn edctnn
## bond_country1           -0.036
## bond_gvmt1              -0.041 -0.359
## age                     -0.378 -0.193 -0.003
## genderwoman             -0.261 -0.035  0.035  0.084
## gendernb                -0.077  0.008  0.019  0.055  0.152
## gendernone              -0.069  0.016 -0.014  0.030  0.125  0.029
## educationundergraduate -0.358  0.012  0.009 -0.180 -0.029 -0.004  0.004
## educationpostgraduate  -0.311  0.051  0.006 -0.252 -0.046 -0.004  0.007  0.722
## GDP                     -0.492  0.061 -0.020 -0.089 -0.082 -0.025 -0.004  0.021
##                         edctnp
## bond_country1
## bond_gvmt1
## age
## genderwoman
## gendernb
## gendernone
## educationundergraduate
## educationpostgraduate
## GDP                     -0.008
##
## Standardized Within-Group Residuals:
##        Min         Q1        Med          Q3         Max
## -3.1379194 -0.6774675 -0.3074765  0.7689827  2.1083948
##
## Number of Observations: 6279
```

```
## Number of Groups: 107
```

```
anova(bond_distancing_B,bond_distancing_B_noRand)
```

```
##                          Model df      AIC      BIC    logLik   Test  L.Ratio
## bond_distancing_B            1 12 17563.30 17644.22 -8769.651
## bond_distancing_B_noRand     2 11 17654.36 17728.54 -8816.182 1 vs 2 93.06079
##                          p-value
## bond_distancing_B
## bond_distancing_B_noRand  <.0001
```

```
leveneTest(residuals(bond_distancing_B) ~ DatasetB_phase1$bond_country) # sig.
```

```
## Levene's Test for Homogeneity of Variance (center = median)
##         Df F value    Pr(>F)
## group    1  12.386 0.0004357 ***
##       6277
## ---
## Signif. codes:  0 '***' 0.001 '**' 0.01 '*' 0.05 '.' 0.1 ' ' 1
```

```
leveneTest(residuals(bond_distancing_B) ~ DatasetB_phase1$bond_gvmt)
```

```
## Levene's Test for Homogeneity of Variance (center = median)
##         Df F value Pr(>F)
## group    1   0.138 0.7103
##       6277
```

```
# Selection between model with homogeneous vs heterogeneous variances and models with/out random effect
summary(bond_distancing_B_homo <- lme(comply_self_scale ~ 1,
                                     random=list(country_now_Name=pdDiag(form=~factor(bond_country))),
                                     data=DatasetB_phase1, na.action=na.exclude, method="REML"))
```

```
## Linear mixed-effects model fit by REML
##   Data: DatasetB_phase1
##        AIC   BIC    logLik
##   18010.91 18038 -9001.454
##
## Random effects:
##  Formula: ~factor(bond_country) | country_now_Name
##  Structure: Diagonal
##         (Intercept) factor(bond_country)1  Residual
## StdDev:   0.1629491            0.06100984 0.9703176
##
## Fixed effects:  comply_self_scale ~ 1
##                   Value  Std.Error   DF   t-value p-value
## (Intercept) 0.003942032 0.03183196 6349 0.1238388  0.9014
##
## Standardized Within-Group Residuals:
##        Min         Q1        Med         Q3        Max
## -3.1819648 -0.6450772 -0.3130424  0.7810537  2.0122561
##
## Number of Observations: 6462
## Number of Groups: 113
```

```r
summary(bond_distancing_B_hetero <- lme(comply_self_scale ~ 1,
                                        random=list(country_now_Name=pdDiag(form=~ factor(bond_country))),
                                        weights=varIdent(form=~ 1|bond_country),
                                        data=DatasetB_phase1, na.action=na.exclude, method="REML"))
```

```
## Linear mixed-effects model fit by REML
##   Data: DatasetB_phase1
##        AIC      BIC   logLik
##   18009.68 18043.55 -8999.84
##
## Random effects:
##  Formula: ~factor(bond_country) | country_now_Name
##  Structure: Diagonal
##         (Intercept) factor(bond_country)1  Residual
## StdDev:   0.1628744            0.06423804 0.9783389
##
## Variance function:
##  Structure: Different standard deviations per stratum
##  Formula: ~1 | bond_country
##  Parameter estimates:
##         0         1
## 1.0000000 0.9621318
## Fixed effects:  comply_self_scale ~ 1
##                   Value  Std.Error   DF   t-value p-value
## (Intercept) 0.004063916 0.03181337 6349 0.1277424  0.8984
##
## Standardized Within-Group Residuals:
##        Min         Q1        Med         Q3        Max
## -3.1556437 -0.6659297 -0.3116782  0.7747167  2.0739797
##
## Number of Observations: 6462
## Number of Groups: 113
```

```r
VarCorr(bond_distancing_B_homo)
```

```
## country_now_Name = pdDiag(factor(bond_country))
##                       Variance    StdDev
## (Intercept)           0.026552425 0.16294915
## factor(bond_country)1 0.003722201 0.06100984
## Residual              0.941516162 0.97031756
```

```r
VarCorr(bond_distancing_B_hetero)
```

```
## country_now_Name = pdDiag(factor(bond_country))
##                       Variance    StdDev
## (Intercept)           0.026528071 0.16287440
## factor(bond_country)1 0.004126526 0.06423804
## Residual              0.957147022 0.97833891
```

```r
anova(bond_distancing_B_homo,bond_distancing_B_hetero)  # No significant difference. Heterogeneous mode
```

```
##                          Model df      AIC      BIC    logLik   Test  L.Ratio
## bond_distancing_B_homo       1  4 18010.91 18038.00 -9001.454
## bond_distancing_B_hetero     2  5 18009.68 18043.55 -8999.840 1 vs 2 3.228216
##                          p-value
## bond_distancing_B_homo
## bond_distancing_B_hetero  0.0724
```

Table S6A: Hypothesis 1b, Dataset A: Does bonding with multiple groups predict health behaviours?

Hypothesis 1b, Dataset A model

```
# Distancing
summary(bond_mult_distancing_A <- lme(Pro_Distancing_scaled ~ bond_mult  +
                                 demo_age + demo_gender + demo_education + GDP,
                                 (~1|Country_Name), data=DatasetA, na.action=na.exclude))
```

```
## Linear mixed-effects model fit by REML
##   Data: DatasetA
##        AIC      BIC    logLik
##   18412.15 18507.14 -9192.076
##
## Random effects:
##  Formula: ~1 | Country_Name
##         (Intercept)  Residual
## StdDev:   0.8293175 0.9707966
##
## Fixed effects:  Pro_Distancing_scaled ~ bond_mult + demo_age + demo_gender +      demo_education + GI
##                                 Value  Std.Error   DF   t-value p-value
## (Intercept)                -0.5522210 0.17643426 6464 -3.129897  0.0018
## bond_mult1                  0.0761238 0.02802346 6464  2.716432  0.0066
## bond_mult2                  0.0666455 0.04109986 6464  1.621550  0.1049
## bond_mult3                  0.1116060 0.06750836 6464  1.653217  0.0983
## bond_mult4                  0.0454349 0.11142114 6464  0.407776  0.6835
## demo_age                    0.0011026 0.00098055 6464  1.124464  0.2609
## demo_genderFemale           0.1540986 0.02525572 6464  6.101533  0.0000
## demo_genderOther/non-binary -0.0012908 0.12801145 6464 -0.010084  0.9920
## demo_educationundergraduate  0.1171603 0.03057913 6464  3.831382  0.0001
## demo_educationpostgraduate   0.0899418 0.03327893 6464  2.702665  0.0069
## demo_educationNA            -0.1679029 0.14274085 6464 -1.176278  0.2395
## GDP                         -0.0000008 0.00000403   69 -0.199327  0.8426
##  Correlation:
##                             (Intr) bnd_m1 bnd_m2 bnd_m3 bnd_m4 demo_g dm_gnF
## bond_mult1                  -0.044
## bond_mult2                  -0.011  0.258
## bond_mult3                  -0.008  0.167  0.131
## bond_mult4                  -0.001  0.110  0.090  0.073
## demo_age                    -0.178 -0.072 -0.110 -0.118 -0.118
## demo_genderFemale           -0.069 -0.030 -0.059 -0.052 -0.030 -0.023
## demo_genderOther/non-binary -0.011 -0.005 -0.012  0.003 -0.002  0.035  0.114
## demo_educationundergraduate -0.087  0.017  0.004  0.027  0.008 -0.073 -0.018
## demo_educationpostgraduate  -0.071  0.005  0.001  0.015  0.033 -0.166 -0.040
```

```
## demo_educationNA            -0.037 -0.008 -0.007 -0.057 -0.020 -0.018 -0.015
## GDP                         -0.698  0.008  0.001  0.007  0.004  0.002 -0.002
##                             dm_O/- dm_dctnn dm_dctnp dm_dNA
## bond_mult1
## bond_mult2
## bond_mult3
## bond_mult4
## demo_age
## demo_genderFemale
## demo_genderOther/non-binary
## demo_educationundergraduate  0.016
## demo_educationpostgraduate   0.006  0.581
## demo_educationNA             0.005  0.121    0.119
## GDP                         -0.031 -0.009   -0.008    0.012
##
## Standardized Within-Group Residuals:
##        Min         Q1        Med         Q3        Max
## -6.7202936 -0.2115388  0.3507508  0.5680169  2.4782858
##
## Number of Observations: 6545
## Number of Groups: 71
```

```
anova.lme(bond_mult_distancing_A,type="marginal")
```

```
##                 numDF denDF   F-value p-value
## (Intercept)         1  6464  9.796253  0.0018
## bond_mult           4  6464  2.392834  0.0484
## demo_age            1  6464  1.264419  0.2609
## demo_gender         2  6464 18.867276  <.0001
## demo_education      3  6464  5.961308  0.0005
## GDP                 1    69  0.039731  0.8426
```

```
r2_nakagawa(bond_mult_distancing_A)
```

```
## # R2 for Mixed Models
##
##   Conditional R2: 0.426
##      Marginal R2: 0.007
```

```
eta_squared(bond_mult_distancing_A)
```

```
## # Effect Size for ANOVA
##
## Parameter      | Eta2 (partial) |       95% CI
## ----------------------------------------------
## bond_mult      |       2.02e-03 | [0.00, 1.00]
## demo_age       |       4.64e-04 | [0.00, 1.00]
## demo_gender    |       5.97e-03 | [0.00, 1.00]
## demo_education |       2.76e-03 | [0.00, 1.00]
## GDP            |       5.75e-04 | [0.00, 1.00]
##
## - One-sided CIs: upper bound fixed at [1.00].
```

```r
# Hygiene
summary(bond_mult_hygiene_A <- lme(Pro_Hygiene_scaled ~ bond_mult  +
                                demo_age + demo_gender + demo_education + GDP,
                                (~1|Country_Name), data=DatasetA, na.action=na.exclude))
```

```
## Linear mixed-effects model fit by REML
##   Data: DatasetA
##        AIC      BIC   logLik
##   17977.46 18072.41 -8974.73
##
## Random effects:
##  Formula: ~1 | Country_Name
##         (Intercept)  Residual
## StdDev:   0.2263891 0.9479781
##
## Fixed effects:  Pro_Hygiene_scaled ~ bond_mult + demo_age + demo_gender + demo_education +      GDP
##                                   Value  Std.Error   DF   t-value p-value
## (Intercept)                  -0.3264495 0.09610503 6448 -3.396799  0.0007
## bond_mult1                    0.1751898 0.02732221 6448  6.411994  0.0000
## bond_mult2                    0.2146639 0.04015769 6448  5.345525  0.0000
## bond_mult3                    0.3788628 0.06569953 6448  5.766598  0.0000
## bond_mult4                    0.3118747 0.10838951 6448  2.877351  0.0040
## demo_age                      0.0058067 0.00095483 6448  6.081365  0.0000
## demo_genderFemale             0.2708026 0.02460753 6448 11.004866  0.0000
## demo_genderOther/non-binary -0.0783665 0.12434645 6448 -0.630227  0.5286
## demo_educationundergraduate -0.0235215 0.02984738 6448 -0.788060  0.4307
## demo_educationpostgraduate  -0.1090943 0.03244030 6448 -3.362925  0.0008
## demo_educationNA              0.3348416 0.13825541 6448  2.421906  0.0155
## GDP                          -0.0000049 0.00000201   70 -2.451162  0.0167
##  Correlation:
##                              (Intr) bnd_m1 bnd_m2 bnd_m3 bnd_m4 demo_g dm_gnF
## bond_mult1                   -0.086
## bond_mult2                   -0.026  0.257
## bond_mult3                   -0.013  0.167  0.130
## bond_mult4                   -0.007  0.110  0.089  0.071
## demo_age                     -0.322 -0.075 -0.111 -0.121 -0.121
## demo_genderFemale            -0.116 -0.028 -0.060 -0.052 -0.030 -0.023
## demo_genderOther/non-binary -0.026 -0.003 -0.011  0.004 -0.002  0.036  0.114
## demo_educationundergraduate -0.161  0.015  0.005  0.028  0.005 -0.074 -0.021
## demo_educationpostgraduate  -0.125  0.005  0.001  0.017  0.033 -0.165 -0.042
## demo_educationNA             -0.050 -0.008 -0.006 -0.055 -0.020 -0.017 -0.016
## GDP                          -0.745  0.023  0.005  0.017  0.021 -0.003 -0.012
##                              dm_O/- dm_dctnn dm_dctnp dm_dNA
## bond_mult1
## bond_mult2
## bond_mult3
## bond_mult4
## demo_age
## demo_genderFemale
## demo_genderOther/non-binary
## demo_educationundergraduate  0.016
## demo_educationpostgraduate   0.005  0.582
## demo_educationNA              0.005  0.122    0.119
```

```
## GDP                         -0.034 -0.012   -0.011    0.015
##
## Standardized Within-Group Residuals:
##         Min          Q1         Med          Q3         Max
## -3.54888578 -0.63313293  0.06716649  0.71770171  2.57616036
##
## Number of Observations: 6530
## Number of Groups: 72
```

```
anova.lme(bond_mult_hygiene_A,type="marginal")
```

```
##                  numDF denDF  F-value p-value
## (Intercept)          1  6448 11.53824  0.0007
## bond_mult            4  6448 19.59981  <.0001
## demo_age             1  6448 36.98299  <.0001
## demo_gender          2  6448 62.35062  <.0001
## demo_education       3  6448  6.99157  0.0001
## GDP                  1    70  6.00819  0.0167
```

```
r2_nakagawa(bond_mult_hygiene_A)
```

```
## # R2 for Mixed Models
##
##   Conditional R2: 0.105
##      Marginal R2: 0.054
```

```
eta_squared(bond_mult_hygiene_A)
```

```
## # Effect Size for ANOVA
##
## Parameter      | Eta2 (partial) |       95% CI
## ----------------------------------------------
## bond_mult      |           0.02 | [0.01, 1.00]
## demo_age       |       5.50e-03 | [0.00, 1.00]
## demo_gender    |           0.02 | [0.01, 1.00]
## demo_education |       3.30e-03 | [0.00, 1.00]
## GDP            |           0.08 | [0.01, 1.00]
##
## - One-sided CIs: upper bound fixed at [1.00].
```

```
# Masking
summary(bond_mult_masking_A <- lme(Pro_MaskWearing_scaled ~ bond_mult  +
                                 demo_age + demo_gender + demo_education + GDP,
                                 random=list(Country_Name=pdDiag(form=~ factor(bond_mult))),
                                 weights=varIdent(form=~ 1|bond_mult), data=DatasetA, na.action=na.exclu
```

```
## Linear mixed-effects model fit by REML
##   Data: DatasetA
##       AIC      BIC    logLik
##   17786.6 17935.83 -8871.298
##
```

```
## Random effects:
##  Formula: ~factor(bond_mult) | Country_Name
##  Structure: Diagonal
##         (Intercept) factor(bond_mult)1 factor(bond_mult)2 factor(bond_mult)3
## StdDev:   0.4432964       0.0001231827         0.06045033          0.1795014
##         factor(bond_mult)4  Residual
## StdDev:          0.1941804 0.8973406
##
## Variance function:
##  Structure: Different standard deviations per stratum
##  Formula: ~1 | bond_mult
##  Parameter estimates:
##        0        1        4        2        3
## 1.000000 1.063405 1.099629 1.127843 1.078735
## Fixed effects:  Pro_MaskWearing_scaled ~ bond_mult + demo_age + demo_gender +      demo_education + [illegible]
##                                   Value  Std.Error   DF    t-value p-value
## (Intercept)                  0.10371713 0.12372020 6455  0.8383201  0.4019
## bond_mult1                   0.05977117 0.02699307 6455  2.2143155  0.0268
## bond_mult2                   0.12266423 0.04681045 6455  2.6204454  0.0088
## bond_mult3                   0.08867481 0.08868000 6455  0.9999415  0.3174
## bond_mult4                   0.10943901 0.13614227 6455  0.8038577  0.4215
## demo_age                     0.00266255 0.00094255 6455  2.8248319  0.0047
## demo_genderFemale            0.04412185 0.02408406 6455  1.8319937  0.0670
## demo_genderOther/non-binary -0.00714291 0.12237260 6455 -0.0583702  0.9535
## demo_educationundergraduate  0.01736594 0.02920059 6455  0.5947119  0.5521
## demo_educationpostgraduate  -0.06198662 0.03184231 6455 -1.9466749  0.0516
## demo_educationNA             0.27606899 0.13805326 6455  1.9997281  0.0456
## GDP                         -0.00000534 0.00000270   69 -1.9770220  0.0520
##  Correlation:
##                             (Intr) bnd_m1 bnd_m2 bnd_m3 bnd_m4 demo_g dm_gnF
## bond_mult1                  -0.058
## bond_mult2                  -0.017  0.200
## bond_mult3                  -0.020  0.110  0.074
## bond_mult4                  -0.008  0.080  0.055  0.038
## demo_age                    -0.241 -0.073 -0.095 -0.086 -0.095
## demo_genderFemale           -0.095 -0.028 -0.049 -0.037 -0.026 -0.022
## demo_genderOther/non-binary -0.017 -0.005 -0.010  0.004 -0.001  0.034  0.113
## demo_educationundergraduate -0.121  0.017  0.005  0.025  0.008 -0.077 -0.019
## demo_educationpostgraduate  -0.098  0.005  0.001  0.015  0.027 -0.171 -0.042
## demo_educationNA            -0.046 -0.009 -0.004 -0.039 -0.011 -0.020 -0.014
## GDP                         -0.722  0.015  0.002  0.014  0.011 -0.002 -0.003
##                             dm_O/- dm_dctnn dm_dctnp dm_dNA
## bond_mult1
## bond_mult2
## bond_mult3
## bond_mult4
## demo_age
## demo_genderFemale
## demo_genderOther/non-binary
## demo_educationundergraduate  0.018
## demo_educationpostgraduate   0.006  0.582
## demo_educationNA             0.006  0.121    0.118
## GDP                         -0.037 -0.010   -0.008    0.015
##
```

```
## Standardized Within-Group Residuals:
##        Min         Q1        Med         Q3        Max 
## -1.7571302 -0.6702419 -0.2871091  0.5656257  3.4510100 
## 
## Number of Observations: 6536
## Number of Groups: 71
```

```
anova.lme(bond_mult_masking_A,type="marginal")
```

```
##                 numDF denDF  F-value p-value
## (Intercept)         1  6455 0.702781  0.4019
## bond_mult           4  6455 2.632207  0.0325
## demo_age            1  6455 7.979675  0.0047
## demo_gender         2  6455 1.713663  0.1803
## demo_education      3  6455 4.258885  0.0052
## GDP                 1    69 3.908616  0.0520
```

```
r2_nakagawa(bond_mult_masking_A)
```

```
## [1] NA
```

```
eta_squared(bond_mult_masking_A)
```

```
## # Effect Size for ANOVA
## 
## Parameter      | Eta2 (partial) |       95% CI
## ----------------------------------------------
## bond_mult      |       2.26e-03 | [0.00, 1.00]
## demo_age       |       9.97e-04 | [0.00, 1.00]
## demo_gender    |       4.96e-04 | [0.00, 1.00]
## demo_education |       2.00e-03 | [0.00, 1.00]
## GDP            |           0.05 | [0.00, 1.00]
## 
## - One-sided CIs: upper bound fixed at [1.00].
```

Hypothesis 1b, Dataset A model check for assumptions and fit

```
# Test homogeneity of variance for the bonding variables and models with/out random effect
# Distancing
bond_mult_distancing_A_noRand <- lm(Pro_Distancing_scaled ~ bond_mult  +
                                demo_age + demo_gender + demo_education + GDP,
                                data=DatasetA, na.action=na.exclude)
bond_mult_distancing_A_homo <- lme(Pro_Distancing_scaled ~ bond_mult  +
                                demo_age + demo_gender + demo_education + GDP,
                                (~1|Country_Name), data=DatasetA, na.action=na.exclude)
anova(bond_mult_distancing_A_homo,bond_mult_distancing_A_noRand)
```

```
##                               Model df      AIC      BIC    logLik   Test
## bond_mult_distancing_A_homo       1 14 18412.15 18507.14 -9192.076
```

```
## bond_mult_distancing_A_noRand     2 13 18555.25 18643.45 -9264.626 1 vs 2
##                                L.Ratio p-value
## bond_mult_distancing_A_homo
## bond_mult_distancing_A_noRand 145.1014  <.0001
```

```
leveneTest(residuals(bond_mult_distancing_A_homo) ~ DatasetA$bond_mult)
```

```
## Levene's Test for Homogeneity of Variance (center = median)
##         Df F value Pr(>F)
## group    4  0.5527  0.697
##       6540
```

```
# Hygiene
bond_mult_hygiene_A_noRand <- lm(Pro_Hygiene_scaled ~ bond_mult  +
                                demo_age + demo_gender + demo_education + GDP,
                                data=DatasetA, na.action=na.exclude)
bond_mult_hygiene_A_homo <- lme(Pro_Hygiene_scaled ~ bond_mult  +
                                demo_age + demo_gender + demo_education + GDP,
                                (~1|Country_Name), data=DatasetA, na.action=na.exclude)
anova(bond_mult_hygiene_A_homo,bond_mult_hygiene_A_noRand)
```

```
##                            Model df      AIC      BIC    logLik   Test  L.Ratio
## bond_mult_hygiene_A_homo       1 14 17977.46 18072.41 -8974.730
## bond_mult_hygiene_A_noRand     2 13 18399.91 18488.08 -9186.954 1 vs 2 424.4486
##                            p-value
## bond_mult_hygiene_A_homo
## bond_mult_hygiene_A_noRand  <.0001
```

```
leveneTest(residuals(bond_mult_hygiene_A_homo) ~ DatasetA$bond_mult)
```

```
## Levene's Test for Homogeneity of Variance (center = median)
##         Df F value  Pr(>F)
## group    4  2.2149 0.06483 .
##       6525
## ---
## Signif. codes:  0 '***' 0.001 '**' 0.01 '*' 0.05 '.' 0.1 ' ' 1
```

```
# Masking
# Selection between model with homogeneous vs heterogeneous variances for bond_country
bond_mult_masking_A_noRand <- lm(Pro_MaskWearing_scaled ~ bond_mult  +
                                demo_age + demo_gender + demo_education + GDP,
                                data=DatasetA, na.action=na.exclude)
bond_mult_masking_A_homo <- lme(Pro_MaskWearing_scaled ~ bond_mult  +
                                demo_age + demo_gender + demo_education + GDP,
                                (~1|Country_Name), data=DatasetA, na.action=na.exclude)
anova(bond_mult_masking_A_homo,bond_mult_masking_A_noRand)
```

```
##                            Model df      AIC      BIC    logLik   Test  L.Ratio
## bond_mult_masking_A_homo       1 14 17797.56 17892.53 -8884.782
## bond_mult_masking_A_noRand     2 13 18501.85 18590.03 -9237.923 1 vs 2 706.2827
##                            p-value
## bond_mult_masking_A_homo
## bond_mult_masking_A_noRand  <.0001
```

```r
leveneTest(residuals(bond_mult_masking_A_homo) ~ DatasetA$bond_mult) # sig.
```

```
## Levene's Test for Homogeneity of Variance (center = median)
##         Df F value    Pr(>F)
## group    4  7.4914 5.126e-06 ***
##       6531
## ---
## Signif. codes:  0 '***' 0.001 '**' 0.01 '*' 0.05 '.' 0.1 ' ' 1
```

```r
bond_mult_masking_A_hetero <- lme(Pro_MaskWearing_scaled ~ bond_mult  +
                                demo_age + demo_gender + demo_education + GDP,
                                random=list(Country_Name=pdDiag(form=~ factor(bond_mult))),
                                weights=varIdent(form=~ 1|bond_mult), data=DatasetA, na.action=na.exclu
VarCorr(bond_mult_masking_A_homo)
```

```
## Country_Name = pdLogChol(1)
##             Variance  StdDev
## (Intercept) 0.1898341 0.4356995
## Residual    0.8673651 0.9313244
```

```r
VarCorr(bond_mult_masking_A_hetero)
```

```
## Country_Name = pdDiag(factor(bond_mult))
##                     Variance     StdDev
## (Intercept)         1.965117e-01 0.4432964386
## factor(bond_mult)1 1.517398e-08 0.0001231827
## factor(bond_mult)2 3.654243e-03 0.0604503325
## factor(bond_mult)3 3.222076e-02 0.1795014074
## factor(bond_mult)4 3.770601e-02 0.1941803617
## Residual            8.052201e-01 0.8973405859
```

```r
anova(bond_mult_masking_A_homo,bond_mult_masking_A_hetero) # Heterogeneous model is significantly bette
```

```
##                            Model df      AIC      BIC    logLik   Test  L.Ratio
## bond_mult_masking_A_homo       1 14 17797.56 17892.53 -8884.782
## bond_mult_masking_A_hetero     2 22 17786.60 17935.83 -8871.298 1 vs 2 26.96618
##                            p-value
## bond_mult_masking_A_homo
## bond_mult_masking_A_hetero   7e-04
```

Table S6B: Hypothesis 1b, Dataset B: Does bonding with multiple groups predict health behaviours?

Hypothesis 1b, Dataset B model

```r
# Distancing
summary(bond_mult_distancing_B <- lme(comply_self_scale ~ bond_mult  +
                                age + gender + education + GDP,
                                random=list(country_now_Name=pdDiag(form=~ factor(bond_mult))),
                                weights=varIdent(form=~ 1|bond_mult), data=DatasetB_phase1, na.action=na
```

```
## Linear mixed-effects model fit by REML
##   Data: DatasetB_phase1
##       AIC      BIC    logLik
##   18167.6 18276.05 -9067.802
##
## Random effects:
##  Formula: ~factor(bond_mult) | country_now_Name
##  Structure: Diagonal
##         (Intercept) factor(bond_mult)1 factor(bond_mult)2  Residual
## StdDev:   0.1595424         0.03798872          0.1187827 0.9274601
##
## Variance function:
##  Structure: Different standard deviations per stratum
##  Formula: ~1 | bond_mult
##  Parameter estimates:
##        1        0        2
## 1.000000 1.054612 1.050100
## Fixed effects:  comply_self_scale ~ bond_mult + age + gender + education + GDP
##                              Value  Std.Error   DF   t-value p-value
## (Intercept)            -0.23245202 0.06161552 6381 -3.772621  0.0002
## bond_mult1             -0.00681488 0.03453393 6381 -0.197339  0.8436
## bond_mult2              0.08521207 0.06698791 6381  1.272051  0.2034
## age                     0.00055791 0.00094579 6381  0.589890  0.5553
## genderwoman             0.05698528 0.02695260 6381  2.114278  0.0345
## gendernb                0.00749849 0.12945528 6381  0.057923  0.9538
## gendernone              0.26690599 0.14687449 6381  1.817239  0.0692
## educationundergraduate  0.06725670 0.03565827 6381  1.886146  0.0593
## educationpostgraduate   0.11476950 0.03850183 6381  2.980885  0.0029
## GDP                     0.00000382 0.00000128  106  2.984969  0.0035
##  Correlation:
##                        (Intr) bnd_m1 bnd_m2 age    gndrwm gndrnb gndrnn edctnn
## bond_mult1             -0.065
## bond_mult2             -0.061  0.128
## age                    -0.376 -0.172 -0.086
## genderwoman            -0.260 -0.025  0.013  0.081
## gendernb               -0.078  0.014  0.018  0.053  0.150
## gendernone             -0.070  0.005  0.002  0.032  0.124  0.029
## educationundergraduate -0.352  0.007  0.008 -0.181 -0.029 -0.001  0.007
## educationpostgraduate  -0.306  0.044  0.023 -0.253 -0.044 -0.001  0.009  0.719
## GDP                    -0.497  0.050  0.016 -0.082 -0.077 -0.023 -0.004  0.019
##                        edctnp
## bond_mult1
## bond_mult2
## age
## genderwoman
## gendernb
## gendernone
## educationundergraduate
## educationpostgraduate
## GDP                    -0.012
##
## Standardized Within-Group Residuals:
##        Min         Q1        Med         Q3        Max
## -3.1250081 -0.6775161 -0.3014223  0.7667837  2.1674173
```

```
##
## Number of Observations: 6497
## Number of Groups: 108
```

```
anova.lme(bond_mult_distancing_B,type="marginal")
```

```
##             numDF denDF   F-value p-value
## (Intercept)     1  6381 14.232673  0.0002
## bond_mult       2  6381  0.875023  0.4169
## age             1  6381  0.347970  0.5553
## gender          3  6381  2.335285  0.0718
## education       2  6381  4.511524  0.0110
## GDP             1   106  8.910043  0.0035
```

```
r2_nakagawa(bond_mult_distancing_B)
```

```
## [1] NA
```

```
eta_squared(bond_mult_distancing_B)
```

```
## # Effect Size for ANOVA
##
## Parameter | Eta2 (partial) |       95% CI
## ------------------------------------------
## bond_mult |       2.63e-04 | [0.00, 1.00]
## age       |       3.42e-04 | [0.00, 1.00]
## gender    |       1.34e-03 | [0.00, 1.00]
## education |       1.46e-03 | [0.00, 1.00]
## GDP       |           0.08 | [0.02, 1.00]
##
## - One-sided CIs: upper bound fixed at [1.00].
```

Hypothesis 1b, Dataset B model check for assumptions and fit

```
# Test homogeneity of variance for the bonding variables and models with/out random effect
bond_mult_distancing_B_noRand <- lm(comply_self_scale ~ bond_mult  +
                                age + gender + education + GDP,
                                data=DatasetB_phase1, na.action=na.exclude)
bond_mult_distancing_B_homo <- lme(comply_self_scale ~ bond_mult  +
                                age + gender + education + GDP,
                                (~1|country_now_Name), data=DatasetB_phase1, na.action=na.exclude)
anova(bond_mult_distancing_B_homo, bond_mult_distancing_B_noRand)
```

```
##                               Model df      AIC      BIC    logLik   Test
## bond_mult_distancing_B_homo       1 12 18165.78 18247.11 -9070.891
## bond_mult_distancing_B_noRand     2 11 18272.73 18347.28 -9125.363 1 vs 2
##                                L.Ratio p-value
## bond_mult_distancing_B_homo
## bond_mult_distancing_B_noRand 108.9447  <.0001
```

```r
leveneTest(residuals(bond_mult_distancing_B_homo) ~ DatasetB_phase1$bond_mult) # sig.
```

```
## Levene's Test for Homogeneity of Variance (center = median)
##         Df F value   Pr(>F)
## group    2  6.6499 0.001303 **
##       6494
## ---
## Signif. codes:  0 '***' 0.001 '**' 0.01 '*' 0.05 '.' 0.1 ' ' 1
```

```r
bond_mult_distancing_B_hetero <- lme(comply_self_scale ~ bond_mult  +
                                age + gender + education + GDP,
                                random=list(country_now_Name=pdDiag(form=~ factor(bond_mult))),
                                weights=varIdent(form=~ 1|bond_mult), data=DatasetB_phase1, na.action=na


# Selection between model with homogeneous vs heterogeneous variances for bond_country
VarCorr(bond_mult_distancing_B_homo)
```

```
## country_now_Name = pdLogChol(1)
##             Variance   StdDev
## (Intercept) 0.02569392 0.1602932
## Residual    0.93990724 0.9694881
```

```r
VarCorr(bond_mult_distancing_B_hetero)
```

```
## country_now_Name = pdDiag(factor(bond_mult))
##                    Variance    StdDev
## (Intercept)        0.025453787 0.15954243
## factor(bond_mult)1 0.001443143 0.03798872
## factor(bond_mult)2 0.014109320 0.11878266
## Residual           0.860182191 0.92746008
```

```r
anova(bond_mult_distancing_B_homo,bond_mult_distancing_B_hetero) # No significant difference between mo
```

```
##                               Model df      AIC      BIC    logLik   Test
## bond_mult_distancing_B_homo       1 12 18165.78 18247.11 -9070.891
## bond_mult_distancing_B_hetero     2 16 18167.60 18276.04 -9067.802 1 vs 2
##                                L.Ratio p-value
## bond_mult_distancing_B_homo
## bond_mult_distancing_B_hetero 6.176941  0.1863
```

Table S7A: Hypothesis 2a, Dataset A: Does bonding (vs no bonding) predict mental health?

Hypothesis 2a, Dataset A model

```r
# Anxiety
summary(bond_anx_A <- lme(anx_sum1_scaled ~ bond_family + bond_friend +
                                bond_country + bond_humanity  +
                              demo_age + demo_gender + demo_education + GDP,
                                   random=list(Country_Name=pdDiag(form=~ factor(bond_country))),
                                   weights=varIdent(form=~ 1|bond_country),
                                   data=DatasetA, na.action=na.exclude, method="REML"))
```

```
## Linear mixed-effects model fit by REML
##   Data: DatasetA
##        AIC      BIC    logLik
##   17699.62 17808.17 -8833.809
##
## Random effects:
##  Formula: ~factor(bond_country) | Country_Name
##  Structure: Diagonal
##         (Intercept) factor(bond_country)1  Residual
## StdDev:   0.2318764             0.1408103 0.9351949
##
## Variance function:
##  Structure: Different standard deviations per stratum
##  Formula: ~1 | bond_country
##  Parameter estimates:
##         0         1
## 1.0000000 0.8903249
## Fixed effects:  anx_sum1_scaled ~ bond_family + bond_friend + bond_country +      bond_humanity + der
##                                 Value  Std.Error   DF   t-value p-value
## (Intercept)                 0.0028194 0.09521667 6463  0.029611  0.9764
## bond_family1               -0.0910565 0.02793414 6463 -3.259685  0.0011
## bond_friend1               -0.1127361 0.04085393 6463 -2.759491  0.0058
## bond_country1              -0.1936566 0.05572576 6463 -3.475172  0.0005
## bond_humanity1             -0.0615969 0.03086387 6463 -1.995762  0.0460
## demo_age                   -0.0086199 0.00093186 6463 -9.250212  0.0000
## demo_genderFemale           0.4603266 0.02399425 6463 19.184871  0.0000
## demo_genderOther/non-binary  0.5430390 0.12269992 6463  4.425748  0.0000
## demo_educationundergraduate -0.0276393 0.02905092 6463 -0.951409  0.3414
## demo_educationpostgraduate  -0.0297292 0.03163853 6463 -0.939653  0.3474
## demo_educationNA            0.1263946 0.13665622 6463  0.924909  0.3550
## GDP                        -0.0000013 0.00000201   70 -0.636368  0.5266
##  Correlation:
##                             (Intr) bnd_fm1 bnd_fr1 bnd_c1 bnd_h1 demo_g dm_gnF
## bond_family1               -0.017
## bond_friend1                0.007 -0.238
## bond_country1              -0.044 -0.062  -0.066
## bond_humanity1             -0.040  0.004  -0.068  -0.220
## demo_age                   -0.312 -0.185  -0.011  -0.046 -0.016
## demo_genderFemale          -0.114 -0.060  -0.059   0.012 -0.018 -0.025
## demo_genderOther/non-binary -0.025  0.019  -0.020   0.002 -0.017  0.032  0.112
## demo_educationundergraduate -0.159  0.055  -0.004   0.012 -0.029 -0.076 -0.021
## demo_educationpostgraduate  -0.124  0.035   0.034   0.018 -0.060 -0.163 -0.043
## demo_educationNA           -0.050 -0.009  -0.040   0.015 -0.023 -0.017 -0.012
## GDP                        -0.746  0.025  -0.015   0.014  0.020 -0.004 -0.011
##                             dm_O/- dm_dctnn dm_dctnp dm_dNA
```

```
## bond_family1
## bond_friend1
## bond_country1
## bond_humanity1
## demo_age
## demo_genderFemale
## demo_genderOther/non-binary
## demo_educationundergraduate  0.016
## demo_educationpostgraduate   0.006  0.582
## demo_educationNA             0.006  0.120    0.117
## GDP                         -0.033 -0.012   -0.012    0.016
##
## Standardized Within-Group Residuals:
##         Min          Q1         Med          Q3         Max
## -2.56812961 -0.74789053 -0.07810434  0.65981789  3.64868776
##
## Number of Observations: 6545
## Number of Groups: 72
```

```
anova.lme(bond_anx_A,type="marginal")
```

```
##                 numDF denDF   F-value p-value
## (Intercept)         1  6463   0.00088  0.9764
## bond_family         1  6463  10.62555  0.0011
## bond_friend         1  6463   7.61479  0.0058
## bond_country        1  6463  12.07682  0.0005
## bond_humanity       1  6463   3.98306  0.0460
## demo_age            1  6463  85.56641  <.0001
## demo_gender         2  6463 186.65218  <.0001
## demo_education      3  6463   0.76297  0.5147
## GDP                 1    70   0.40496  0.5266
```

```
r2_nakagawa(bond_anx_A)
```

```
## [1] NA
```

```
eta_squared(bond_anx_A)
```

```
## # Effect Size for ANOVA
##
## Parameter      | Eta2 (partial) |       95% CI
## -----------------------------------------------
## bond_family    |       3.77e-03 | [0.00, 1.00]
## bond_friend    |       7.20e-04 | [0.00, 1.00]
## bond_country   |       3.29e-03 | [0.00, 1.00]
## bond_humanity  |       5.25e-04 | [0.00, 1.00]
## demo_age       |           0.01 | [0.01, 1.00]
## demo_gender    |           0.05 | [0.05, 1.00]
## demo_education |       3.61e-04 | [0.00, 1.00]
## GDP            |       5.75e-03 | [0.00, 1.00]
##
## - One-sided CIs: upper bound fixed at [1.00].
```

```r
# Depression
var_str <- varComb(varIdent(form=~ 1|bond_family), varIdent(form=~ 1|bond_friend), varIdent(form=~ 1|bon
summary(bond_dep_A <- lme(dep_sum1_scaled ~ bond_family + bond_friend +
                                 bond_country + bond_humanity +
                            demo_age + demo_gender + demo_education + GDP,
                          random=list(Country_Name=pdDiag(form=~
                                                   bond_family + bond_friend + bond_country)),
                                 weights=var_str, data=DatasetA, na.action=na.exclude, method="REML
```

```
## Linear mixed-effects model fit by REML
##   Data: DatasetA
##        AIC      BIC    logLik
##   18120.79 18256.49 -9040.394
##
## Random effects:
##  Formula: ~bond_family + bond_friend + bond_country | Country_Name
##  Structure: Diagonal
##         (Intercept) bond_family1 bond_friend1 bond_country1  Residual
## StdDev:   0.1936641   0.04087177   0.04010227     0.1025794 0.9745105
##
## Combination of variance functions:
##  Structure: Different standard deviations per stratum
##  Formula: ~1 | bond_family
##  Parameter estimates:
##         0         1
## 1.0000000 0.9709529
##  Structure: Different standard deviations per stratum
##  Formula: ~1 | bond_friend
##  Parameter estimates:
##         0         1
## 1.0000000 0.9676842
##  Structure: Different standard deviations per stratum
##  Formula: ~1 | bond_country
##  Parameter estimates:
##         0         1
## 1.0000000 0.9053137
## Fixed effects:  dep_sum1_scaled ~ bond_family + bond_friend + bond_country +      bond_humanity + der
##                                   Value  Std.Error   DF   t-value p-value
## (Intercept)                   0.2600186 0.09123669 6465  2.849934  0.0044
## bond_family1                 -0.1665435 0.03177922 6465 -5.240642  0.0000
## bond_friend1                 -0.2606379 0.04310684 6465 -6.046325  0.0000
## bond_country1                -0.1459998 0.05009892 6465 -2.914231  0.0036
## bond_humanity1               -0.1401334 0.03183201 6465 -4.402278  0.0000
## demo_age                     -0.0055155 0.00095958 6465 -5.747848  0.0000
## demo_genderFemale             0.2236946 0.02477108 6465  9.030472  0.0000
## demo_genderOther/non-binary   0.5350000 0.12717206 6465  4.206899  0.0000
## demo_educationundergraduate  -0.1158486 0.02997331 6465 -3.865059  0.0001
## demo_educationpostgraduate   -0.1356394 0.03260169 6465 -4.160502  0.0000
## demo_educationNA              0.0265069 0.13942464 6465  0.190116  0.8492
## GDP                          -0.0000030 0.00000190   70 -1.599605  0.1142
##  Correlation:
##                              (Intr) bnd_fm1 bnd_fr1 bnd_c1 bnd_h1 demo_g dm_gnF
## bond_family1                 -0.032
```

```
## bond_friend1                  0.004 -0.208
## bond_country1                -0.032 -0.071  -0.074
## bond_humanity1               -0.043  0.004  -0.066  -0.250
## demo_age                     -0.334 -0.172  -0.012  -0.057 -0.018
## demo_genderFemale            -0.121 -0.054  -0.059   0.015 -0.018 -0.025
## demo_genderOther/non-binary -0.028  0.019  -0.019   0.003 -0.018  0.033  0.112
## demo_educationundergraduate -0.172  0.046  -0.005   0.013 -0.029 -0.075 -0.020
## demo_educationpostgraduate  -0.132  0.032   0.033   0.023 -0.060 -0.162 -0.042
## demo_educationNA            -0.050 -0.007  -0.038   0.014 -0.025 -0.017 -0.013
## GDP                          -0.749  0.032  -0.015   0.009  0.024 -0.007 -0.014
##                              dm_O/- dm_dctnn dm_dctnp dm_dNA
## bond_family1
## bond_friend1
## bond_country1
## bond_humanity1
## demo_age
## demo_genderFemale
## demo_genderOther/non-binary
## demo_educationundergraduate  0.016
## demo_educationpostgraduate   0.007  0.581
## demo_educationNA             0.006  0.121    0.118
## GDP                          -0.032 -0.012   -0.012    0.015
##
## Standardized Within-Group Residuals:
##        Min          Q1         Med          Q3         Max
## -2.5150094 -0.7546344 -0.0719684  0.6654330  4.2592413
##
## Number of Observations: 6547
## Number of Groups: 72
```

```
anova.lme(bond_dep_A,type="marginal")
```

```
##                 numDF denDF  F-value p-value
## (Intercept)         1  6465  8.12213  0.0044
## bond_family         1  6465 27.46433  <.0001
## bond_friend         1  6465 36.55804  <.0001
## bond_country        1  6465  8.49274  0.0036
## bond_humanity       1  6465 19.38006  <.0001
## demo_age            1  6465 33.03776  <.0001
## demo_gender         2  6465 45.95414  <.0001
## demo_education      3  6465  7.03733  0.0001
## GDP                 1    70  2.55874  0.1142
```

```
r2_nakagawa(bond_dep_A)
```

```
## [1] NA
```

```
eta_squared(bond_dep_A)
```

```
## # Effect Size for ANOVA
##
## Parameter      | Eta2 (partial) |       95% CI
```

```
## ---------------------------------------------
## bond_family    |       9.41e-03 | [0.01, 1.00]
## bond_friend    |       5.99e-03 | [0.00, 1.00]
## bond_country   |       3.25e-03 | [0.00, 1.00]
## bond_humanity  |       3.18e-03 | [0.00, 1.00]
## demo_age       |       6.15e-03 | [0.00, 1.00]
## demo_gender    |           0.01 | [0.01, 1.00]
## demo_education |       3.29e-03 | [0.00, 1.00]
## GDP            |           0.04 | [0.00, 1.00]
##
## - One-sided CIs: upper bound fixed at [1.00].
```

Hypothesis 2a, Dataset A model check for assumptions and fit

```r
# Anxiety
# Test homogeneity of variance for the bonding variables and models with/out random effect
summary(bond_anx_A_noRand <- lm(anx_sum1_scaled ~ bond_family + bond_friend +
                                 bond_country + bond_humanity  +
                                demo_age + demo_gender + demo_education + GDP,
                                     data=DatasetA, na.action=na.exclude, method="REML"))
```

```
##
## Call:
## lm(formula = anx_sum1_scaled ~ bond_family + bond_friend + bond_country +
##     bond_humanity + demo_age + demo_gender + demo_education +
##     GDP, data = DatasetA, na.action = na.exclude, method = "REML")
##
## Residuals:
##     Min      1Q  Median      3Q     Max
## -2.4297 -0.7177 -0.0873  0.6218  3.3039
##
## Coefficients:
##                                Estimate Std. Error t value Pr(>|t|)
## (Intercept)                   1.880e-01  4.772e-02   3.940 8.24e-05 ***
## bond_family1                 -9.336e-02  2.881e-02  -3.241   0.0012 **
## bond_friend1                 -1.026e-01  4.256e-02  -2.410   0.0160 *
## bond_country1                -2.786e-01  4.147e-02  -6.718 1.99e-11 ***
## bond_humanity1               -9.221e-03  3.201e-02  -0.288   0.7733
## demo_age                     -1.103e-02  9.260e-04 -11.906  < 2e-16 ***
## demo_genderFemale             4.598e-01  2.460e-02  18.692  < 2e-16 ***
## demo_genderOther/non-binary   5.501e-01  1.261e-01   4.361 1.31e-05 ***
## demo_educationundergraduate  -2.023e-02  2.937e-02  -0.689   0.4910
## demo_educationpostgraduate   -2.953e-02  3.213e-02  -0.919   0.3580
## demo_educationNA              7.247e-02  1.417e-01   0.511   0.6091
## GDP                           1.047e-06  6.273e-07   1.669   0.0952 .
## ---
## Signif. codes:  0 '***' 0.001 '**' 0.01 '*' 0.05 '.' 0.1 ' ' 1
##
## Residual standard error: 0.9561 on 6533 degrees of freedom
##   (44 observations deleted due to missingness)
## Multiple R-squared:  0.0885, Adjusted R-squared:  0.08697
## F-statistic: 57.67 on 11 and 6533 DF,  p-value: < 2.2e-16
```

```r
summary(bond_anx_A <- lme(anx_sum1_scaled ~ bond_family + bond_friend +
                                bond_country + bond_humanity  +
                               demo_age + demo_gender + demo_education + GDP, (~1|Country_Name),
                         data=DatasetA, na.action=na.exclude, method="REML"))
```

```
## Linear mixed-effects model fit by REML
##   Data: DatasetA
##        AIC      BIC    logLik
##   17714.89 17809.87 -8843.445
##
## Random effects:
##  Formula: ~1 | Country_Name
##         (Intercept)  Residual
## StdDev:   0.2334546 0.9258271
##
## Fixed effects:  anx_sum1_scaled ~ bond_family + bond_friend + bond_country +      bond_humanity + de[illegible]
##                                  Value  Std.Error   DF   t-value p-value
## (Intercept)                  0.0078640 0.09545474 6463  0.082385  0.9343
## bond_family1                -0.0936932 0.02807713 6463 -3.336994  0.0009
## bond_friend1                -0.1082464 0.04134548 6463 -2.618094  0.0089
## bond_country1               -0.1709127 0.04091382 6463 -4.177382  0.0000
## bond_humanity1              -0.0661739 0.03123948 6463 -2.118279  0.0342
## demo_age                    -0.0086079 0.00093689 6463 -9.187744  0.0000
## demo_genderFemale            0.4620356 0.02406193 6463 19.201931  0.0000
## demo_genderOther/non-binary  0.5433548 0.12251756 6463  4.434914  0.0000
## demo_educationundergraduate -0.0304430 0.02913008 6463 -1.045071  0.2960
## demo_educationpostgraduate  -0.0313728 0.03173391 6463 -0.988621  0.3229
## demo_educationNA             0.1372318 0.13775340 6463  0.996214  0.3192
## GDP                         -0.0000014 0.00000201   70 -0.698240  0.4873
##  Correlation:
##                             (Intr) bnd_fm1 bnd_fr1 bnd_c1 bnd_h1 demo_g dm_gnF
## bond_family1                -0.017
## bond_friend1                 0.007 -0.235
## bond_country1               -0.012 -0.087  -0.089
## bond_humanity1              -0.041  0.004  -0.065  -0.298
## demo_age                    -0.313 -0.182  -0.013  -0.074 -0.013
## demo_genderFemale           -0.115 -0.060  -0.060   0.018 -0.017 -0.022
## demo_genderOther/non-binary -0.026  0.020  -0.022   0.006 -0.018  0.033  0.113
## demo_educationundergraduate -0.157  0.055  -0.006   0.012 -0.029 -0.079 -0.021
## demo_educationpostgraduate  -0.121  0.034   0.035   0.030 -0.060 -0.168 -0.043
## demo_educationNA            -0.051 -0.008  -0.040   0.007 -0.025 -0.018 -0.011
## GDP                         -0.746  0.024  -0.015   0.005  0.021 -0.003 -0.010
##                             dm_O/- dm_dctnn dm_dctnp dm_dNA
## bond_family1
## bond_friend1
## bond_country1
## bond_humanity1
## demo_age
## demo_genderFemale
## demo_genderOther/non-binary
## demo_educationundergraduate  0.017
## demo_educationpostgraduate   0.008  0.582
## demo_educationNA             0.007  0.121    0.117
```

```
## GDP                          -0.034 -0.012   -0.012    0.016
##
## Standardized Within-Group Residuals:
##         Min          Q1         Med          Q3         Max
## -2.59161407 -0.74493421 -0.07792846  0.65632604  3.59758529
##
## Number of Observations: 6545
## Number of Groups: 72
```

```
anova(bond_anx_A,bond_anx_A_noRand)
```

```
##                   Model df      AIC      BIC    logLik   Test  L.Ratio p-value
## bond_anx_A            1 14 17714.89 17809.88 -8843.445
## bond_anx_A_noRand     2 13 18086.42 18174.62 -9030.211 1 vs 2 373.5318  <.0001
```

```
leveneTest(residuals(bond_anx_A) ~ DatasetA$bond_family)
```

```
## Levene's Test for Homogeneity of Variance (center = median)
##         Df F value Pr(>F)
## group    1  2.3057 0.1289
##       6543
```

```
leveneTest(residuals(bond_anx_A) ~ DatasetA$bond_friend)
```

```
## Levene's Test for Homogeneity of Variance (center = median)
##         Df F value Pr(>F)
## group    1  0.1299 0.7185
##       6543
```

```
leveneTest(residuals(bond_anx_A) ~ DatasetA$bond_country) # sig.
```

```
## Levene's Test for Homogeneity of Variance (center = median)
##         Df F value   Pr(>F)
## group    1  9.7079 0.001843 **
##       6543
## ---
## Signif. codes:  0 '***' 0.001 '**' 0.01 '*' 0.05 '.' 0.1 ' ' 1
```

```
leveneTest(residuals(bond_anx_A) ~ DatasetA$bond_humanity)
```

```
## Levene's Test for Homogeneity of Variance (center = median)
##         Df F value Pr(>F)
## group    1  0.3062 0.5801
##       6543
```

```
# Selection between model with homogeneous vs heterogeneous variances for bond_country
summary(bond_anx_A_homo <- lme(anx_sum1_scaled ~ 1,
                                     random=list(Country_Name=pdDiag(form=~factor(bond_country))),
                                     data=DatasetA, na.action=na.exclude, method="REML"))
```

```
## Linear mixed-effects model fit by REML
##   Data: DatasetA
##        AIC      BIC    logLik
##   18152.81 18179.97 -9072.405
##
## Random effects:
##  Formula: ~factor(bond_country) | Country_Name
##  Structure: Diagonal
##         (Intercept) factor(bond_country)1  Residual
## StdDev:   0.2477494             0.2807644 0.9575876
##
## Fixed effects:  anx_sum1_scaled ~ 1
##                  Value  Std.Error   DF   t-value p-value
## (Intercept) -0.1759285 0.05193209 6493 -3.387665   7e-04
##
## Standardized Within-Group Residuals:
##        Min         Q1        Med         Q3        Max
## -2.2460037 -0.7448997 -0.1042003  0.6826604  3.3479919
##
## Number of Observations: 6567
## Number of Groups: 74
```

```r
summary(bond_anx_A_hetero <- lme(anx_sum1_scaled ~ 1,
                                     random=list(Country_Name=pdDiag(form=~ factor(bond_country))),
                                     weights=varIdent(form=~ 1|bond_country),
                                     data=DatasetA, na.action=na.exclude, method="REML"))
```

```
## Linear mixed-effects model fit by REML
##   Data: DatasetA
##        AIC      BIC    logLik
##   18138.96 18172.91 -9064.481
##
## Random effects:
##  Formula: ~factor(bond_country) | Country_Name
##  Structure: Diagonal
##         (Intercept) factor(bond_country)1  Residual
## StdDev:   0.2471319             0.2912353 0.9682154
##
## Variance function:
##  Structure: Different standard deviations per stratum
##  Formula: ~1 | bond_country
##  Parameter estimates:
##         0         1
## 1.0000000 0.8886574
## Fixed effects:  anx_sum1_scaled ~ 1
##                 Value  Std.Error   DF   t-value p-value
## (Intercept) -0.179872 0.05182887 6493 -3.470497   5e-04
##
## Standardized Within-Group Residuals:
##        Min         Q1        Med         Q3        Max
## -2.2211758 -0.7364447 -0.1026795  0.6752081  3.5023316
##
## Number of Observations: 6567
## Number of Groups: 74
```

```r
VarCorr(bond_anx_A_homo)
```

```
## Country_Name = pdDiag(factor(bond_country))
##                        Variance   StdDev
## (Intercept)            0.06137976 0.2477494
## factor(bond_country)1 0.07882866 0.2807644
## Residual               0.91697406 0.9575876
```

```r
VarCorr(bond_anx_A_hetero)
```

```
## Country_Name = pdDiag(factor(bond_country))
##                        Variance   StdDev
## (Intercept)            0.06107418 0.2471319
## factor(bond_country)1 0.08481800 0.2912353
## Residual               0.93744113 0.9682154
```

```r
anova(bond_anx_A_homo,bond_anx_A_hetero) # Heterogeneous model is significantly better (lower AIC)
```

```
##                    Model df      AIC      BIC    logLik   Test  L.Ratio p-value
## bond_anx_A_homo        1  4 18152.81 18179.97 -9072.405
## bond_anx_A_hetero      2  5 18138.96 18172.91 -9064.481 1 vs 2 15.84914   1e-04
```

```r
# Depression
# Test homogeneity of variance for the bonding variables and models with/out random effect
summary(bond_dep_A_noRand <- lm(dep_sum1_scaled ~ bond_family + bond_friend +
                                 bond_country + bond_humanity  +
                                demo_age + demo_gender + demo_education + GDP,
                                data=DatasetA, na.action=na.exclude, method="REML"))
```

```
##
## Call:
## lm(formula = dep_sum1_scaled ~ bond_family + bond_friend + bond_country +
##     bond_humanity + demo_age + demo_gender + demo_education +
##     GDP, data = DatasetA, na.action = na.exclude, method = "REML")
##
## Residuals:
##     Min      1Q  Median      3Q     Max
## -2.1904 -0.7465 -0.1030  0.6466  3.8270
##
## Coefficients:
##                                 Estimate Std. Error t value Pr(>|t|)
## (Intercept)                    3.362e-01  4.863e-02   6.913 5.19e-12 ***
## bond_family1                  -1.368e-01  2.937e-02  -4.657 3.27e-06 ***
## bond_friend1                  -2.766e-01  4.338e-02  -6.375 1.95e-10 ***
## bond_country1                 -2.141e-01  4.227e-02  -5.065 4.19e-07 ***
## bond_humanity1                -1.001e-01  3.264e-02  -3.068  0.00216 **
## demo_age                      -4.329e-03  9.439e-04  -4.586 4.59e-06 ***
## demo_genderFemale              2.541e-01  2.507e-02  10.137  < 2e-16 ***
## demo_genderOther/non-binary    5.496e-01  1.286e-01   4.275 1.94e-05 ***
## demo_educationundergraduate   -1.853e-01  2.993e-02  -6.192 6.31e-10 ***
## demo_educationpostgraduate    -2.140e-01  3.274e-02  -6.536 6.80e-11 ***
```

```
## demo_educationNA              3.412e-02  1.444e-01   0.236  0.81323
## GDP                          -1.837e-06  6.394e-07  -2.873  0.00408 **
## ---
## Signif. codes:  0 '***' 0.001 '**' 0.01 '*' 0.05 '.' 0.1 ' ' 1
##
## Residual standard error: 0.9745 on 6535 degrees of freedom
##   (42 observations deleted due to missingness)
## Multiple R-squared:  0.05153,    Adjusted R-squared:  0.04994
## F-statistic: 32.28 on 11 and 6535 DF,  p-value: < 2.2e-16
```

```
summary(bond_dep_A <- lme(dep_sum1_scaled ~ bond_family + bond_friend +
                                 bond_country + bond_humanity  +
                                demo_age + demo_gender + demo_education + GDP,
                                (~1|Country_Name), data=DatasetA, na.action=na.exclude, method="REML"))
```

```
## Linear mixed-effects model fit by REML
##   Data: DatasetA
##        AIC      BIC    logLik
##   18128.44 18223.43 -9050.222
##
## Random effects:
##  Formula: ~1 | Country_Name
##         (Intercept)  Residual
## StdDev:   0.1973156 0.9557705
##
## Fixed effects:  dep_sum1_scaled ~ bond_family + bond_friend + bond_country +      bond_humanity + de
##                                   Value  Std.Error   DF   t-value p-value
## (Intercept)                   0.2684959 0.09179795 6465  2.924857  0.0035
## bond_family1                 -0.1709066 0.02898433 6465 -5.896517  0.0000
## bond_friend1                 -0.2586300 0.04267281 6465 -6.060768  0.0000
## bond_country1                -0.1357465 0.04221411 6465 -3.215666  0.0013
## bond_humanity1               -0.1454558 0.03225550 6465 -4.509488  0.0000
## demo_age                     -0.0055148 0.00096635 6465 -5.706844  0.0000
## demo_genderFemale             0.2231715 0.02482176 6465  8.990963  0.0000
## demo_genderOther/non-binary   0.5432994 0.12640153 6465  4.298202  0.0000
## demo_educationundergraduate -0.1179943 0.03005529 6465 -3.925910  0.0001
## demo_educationpostgraduate   -0.1382584 0.03273017 6465 -4.224188  0.0000
## demo_educationNA              0.0153110 0.14209939 6465  0.107748  0.9142
## GDP                          -0.0000032 0.00000191   70 -1.687316  0.0960
##  Correlation:
##                             (Intr) bnd_fm1 bnd_fr1 bnd_c1 bnd_h1 demo_g dm_gnF
## bond_family1                -0.019
## bond_friend1                 0.005 -0.235
## bond_country1               -0.012 -0.088  -0.089
## bond_humanity1              -0.044  0.004  -0.065  -0.298
## demo_age                    -0.335 -0.183  -0.013  -0.074 -0.014
## demo_genderFemale           -0.121 -0.060  -0.059   0.018 -0.017 -0.022
## demo_genderOther/non-binary -0.028  0.020  -0.022   0.006 -0.018  0.033  0.112
## demo_educationundergraduate -0.170  0.055  -0.006   0.012 -0.029 -0.079 -0.020
## demo_educationpostgraduate  -0.129  0.035   0.034   0.030 -0.060 -0.168 -0.043
## demo_educationNA            -0.051 -0.008  -0.040   0.007 -0.025 -0.018 -0.011
## GDP                         -0.748  0.027  -0.014   0.005  0.023 -0.006 -0.014
##                             dm_O/- dm_dctnn dm_dctnp dm_dNA
## bond_family1
```

```
## bond_friend1
## bond_country1
## bond_humanity1
## demo_age
## demo_genderFemale
## demo_genderOther/non-binary
## demo_educationundergraduate  0.017
## demo_educationpostgraduate   0.008  0.583
## demo_educationNA             0.007  0.121    0.117
## GDP                         -0.033 -0.012   -0.012    0.016
##
## Standardized Within-Group Residuals:
##         Min          Q1         Med          Q3         Max
## -2.59440125 -0.75675464 -0.06984577  0.66769719  4.11143362
##
## Number of Observations: 6547
## Number of Groups: 72
```

```
anova(bond_dep_A, bond_dep_A_noRand)
```

```
##                   Model df      AIC      BIC    logLik   Test  L.Ratio p-value
## bond_dep_A            1 14 18128.44 18223.43 -9050.222
## bond_dep_A_noRand     2 13 18341.03 18429.23 -9157.513 1 vs 2 214.5831  <.0001
```

```
leveneTest(residuals(bond_dep_A) ~ DatasetA$bond_family) # sig.
```

```
## Levene's Test for Homogeneity of Variance (center = median)
##         Df F value   Pr(>F)
## group    1  8.3111 0.003953 **
##       6545
## ---
## Signif. codes:  0 '***' 0.001 '**' 0.01 '*' 0.05 '.' 0.1 ' ' 1
```

```
leveneTest(residuals(bond_dep_A) ~ DatasetA$bond_friend) # sig.
```

```
## Levene's Test for Homogeneity of Variance (center = median)
##         Df F value   Pr(>F)
## group    1  8.1253 0.004379 **
##       6545
## ---
## Signif. codes:  0 '***' 0.001 '**' 0.01 '*' 0.05 '.' 0.1 ' ' 1
```

```
leveneTest(residuals(bond_dep_A) ~ DatasetA$bond_country) # sig.
```

```
## Levene's Test for Homogeneity of Variance (center = median)
##         Df F value    Pr(>F)
## group    1   16.48 4.975e-05 ***
##       6545
## ---
## Signif. codes:  0 '***' 0.001 '**' 0.01 '*' 0.05 '.' 0.1 ' ' 1
```

```r
leveneTest(residuals(bond_dep_A) ~ DatasetA$bond_humanity)
```

```
## Levene's Test for Homogeneity of Variance (center = median)
##         Df F value Pr(>F)
## group    1  2.1059 0.1468
##       6545
```

```r
# Selection between model with homogeneous vs heterogeneous variances for bond_family, bond_friend, bon
var_str <- varComb(varIdent(form=~ 1|bond_family), varIdent(form=~ 1|bond_friend), varIdent(form=~ 1|bon
summary(bond_dep_A_homo <- lme(dep_sum1_scaled ~ 1,
                              random=list(Country_Name=pdDiag(form=~ bond_family+ bond_friend+ bond_cou
                                    data=DatasetA, na.action=na.exclude, method="REML"))
```

```
## Linear mixed-effects model fit by REML
##   Data: DatasetA
##        AIC      BIC    logLik
##   18309.75 18350.49 -9148.877
##
## Random effects:
##  Formula: ~bond_family + bond_friend + bond_country | Country_Name
##  Structure: Diagonal
##         (Intercept) bond_family1 bond_friend1 bond_country1  Residual
## StdDev:   0.2236025    0.1931732    0.2524316     0.2271916 0.9673407
##
## Fixed effects:  dep_sum1_scaled ~ 1
##                   Value  Std.Error   DF   t-value p-value
## (Intercept) -0.09983293 0.04924208 6491 -2.027391  0.0427
##
## Standardized Within-Group Residuals:
##        Min         Q1        Med         Q3        Max
## -2.2009706 -0.7470473 -0.1107059  0.6331853  3.7375263
##
## Number of Observations: 6565
## Number of Groups: 74
```

```r
summary(bond_dep_A_hetero <- lme(dep_sum1_scaled ~ 1,
                                    random=list(Country_Name=pdDiag(form=~ bond_family+ bond_friend+ bo
                                    weights=var_str, data=DatasetA, na.action=na.exclude, method="REML
```

```
## Linear mixed-effects model fit by REML
##   Data: DatasetA
##        AIC     BIC    logLik
##   18296.99 18358.1 -9139.497
##
## Random effects:
##  Formula: ~bond_family + bond_friend + bond_country | Country_Name
##  Structure: Diagonal
##         (Intercept) bond_family1 bond_friend1 bond_country1  Residual
## StdDev:   0.2230392    0.1946742    0.2608229     0.2371868 0.9876194
##
## Combination of variance functions:
##  Structure: Different standard deviations per stratum
```

```
##  Formula: ~1 | bond_family
##  Parameter estimates:
##         0         1
## 1.0000000 0.9663695
##  Structure: Different standard deviations per stratum
##  Formula: ~1 | bond_friend
##  Parameter estimates:
##         0         1
## 1.0000000 0.9700494
##  Structure: Different standard deviations per stratum
##  Formula: ~1 | bond_country
##  Parameter estimates:
##         0         1
## 1.0000000 0.9056227
## Fixed effects:  dep_sum1_scaled ~ 1
##                  Value  Std.Error   DF   t-value p-value
## (Intercept) -0.1028801 0.04920162 6491 -2.090989  0.0366
##
## Standardized Within-Group Residuals:
##        Min          Q1         Med          Q3         Max
## -2.1545797 -0.7381155 -0.1097344  0.6347211  3.7748074
##
## Number of Observations: 6565
## Number of Groups: 74
```

```
VarCorr(bond_dep_A_homo)
```

```
## Country_Name = pdDiag(bond_family + bond_friend + bond_country)
##               Variance   StdDev
## (Intercept)   0.04999808 0.2236025
## bond_family1  0.03731587 0.1931732
## bond_friend1  0.06372171 0.2524316
## bond_country1 0.05161601 0.2271916
## Residual      0.93574797 0.9673407
```

```
VarCorr(bond_dep_A_hetero)
```

```
## Country_Name = pdDiag(bond_family + bond_friend + bond_country)
##               Variance   StdDev
## (Intercept)   0.04974650 0.2230392
## bond_family1  0.03789803 0.1946742
## bond_friend1  0.06802858 0.2608229
## bond_country1 0.05625757 0.2371868
## Residual      0.97539204 0.9876194
```

```
anova(bond_dep_A_homo,bond_dep_A_hetero) # Heterogeneous model is significantly better (lower AIC)
```

```
##                   Model df      AIC      BIC    logLik   Test  L.Ratio p-value
## bond_dep_A_homo       1  6 18309.75 18350.49 -9148.877
## bond_dep_A_hetero     2  9 18296.99 18358.10 -9139.497 1 vs 2 18.75937   3e-04
```

Table S7B: Hypothesis 2a, Dataset B: Does bonding (vs no bonding) predict wellbeing?

Hypothesis 2a, Dataset B model

```r
# Wellbeing
var_str <- varComb(varIdent(form=~ 1|bond_country), varIdent(form=~ 1|bond_gvmt))
summary(bond_wellbeing_B <- lme(wellbeing_scale ~ bond_country + bond_gvmt  +
                                        age + gender + education + GDP,
                                     random=list(country_now_Name=pdDiag(form=~ bond_country+bond_gvmt)
                                     weights=var_str,
                                     data=DatasetB_phase1, na.action=na.exclude, method="REML"))
```

```
## Linear mixed-effects model fit by REML
##   Data: DatasetB_phase1
##        AIC      BIC    logLik
##   16959.48 17067.37 -8463.738
##
## Random effects:
##  Formula: ~bond_country + bond_gvmt | country_now_Name
##  Structure: Diagonal
##         (Intercept) bond_country1   bond_gvmt1  Residual
## StdDev:   0.1963958     0.1389683 0.0002591058 0.9639341
##
## Combination of variance functions:
##  Structure: Different standard deviations per stratum
##  Formula: ~1 | bond_country
##  Parameter estimates:
##         1         0
## 1.0000000 0.9367443
##  Structure: Different standard deviations per stratum
##  Formula: ~1 | bond_gvmt
##  Parameter estimates:
##        0        1
## 1.000000 1.097826
## Fixed effects:  wellbeing_scale ~ bond_country + bond_gvmt + age + gender + education +      GDP
##                             Value  Std.Error   DF   t-value p-value
## (Intercept)            -0.5511644 0.06412719 6164 -8.594863  0.0000
## bond_country1           0.2699748 0.04943697 6164  5.460989  0.0000
## bond_gvmt1              0.2464324 0.05678749 6164  4.339554  0.0000
## age                     0.0142592 0.00092498 6164 15.415666  0.0000
## genderwoman            -0.1544721 0.02624043 6164 -5.886796  0.0000
## gendernb               -0.2676343 0.12277696 6164 -2.179841  0.0293
## gendernone             -0.3460239 0.14016177 6164 -2.468747  0.0136
## educationundergraduate  0.0698759 0.03473813 6164  2.011505  0.0443
## educationpostgraduate   0.1449821 0.03732543 6164  3.884271  0.0001
## GDP                    -0.0000021 0.00000138  105 -1.514485  0.1329
##  Correlation:
##                        (Intr) bnd_c1 bnd_g1 age    gndrwm gndrnb gndrnn edctnn
## bond_country1          -0.097
## bond_gvmt1             -0.028 -0.245
## age                    -0.361 -0.101 -0.009
```

```
## genderwoman            -0.245 -0.012  0.032  0.085
## gendernb               -0.073  0.006  0.017  0.056  0.155
## gendernone             -0.064  0.011 -0.012  0.030  0.126  0.030
## educationundergraduate -0.327  0.002  0.010 -0.185 -0.030 -0.006  0.004
## educationpostgraduate  -0.284  0.025  0.008 -0.259 -0.048 -0.005  0.006  0.723
## GDP                    -0.519  0.055 -0.022 -0.069 -0.070 -0.020 -0.002  0.016
##                        edctnp
## bond_country1
## bond_gvmt1
## age
## genderwoman
## gendernb
## gendernone
## educationundergraduate
## educationpostgraduate
## GDP                    -0.009
##
## Standardized Within-Group Residuals:
##         Min          Q1         Med          Q3         Max
## -4.81904925 -0.67465273 -0.05767896  0.59595858  4.07080814
##
## Number of Observations: 6279
## Number of Groups: 107
```

```
anova.lme(bond_wellbeing_B,type="marginal")
```

```
##              numDF denDF   F-value p-value
## (Intercept)      1  6164  73.87167  <.0001
## bond_country     1  6164  29.82241  <.0001
## bond_gvmt        1  6164  18.83173  <.0001
## age              1  6164 237.64277  <.0001
## gender           3  6164  13.09366  <.0001
## education        2  6164   8.20789  0.0003
## GDP              1   105   2.29367  0.1329
```

```
r2_nakagawa(bond_wellbeing_B)
```

```
## [1] NA
```

```
eta_squared(bond_wellbeing_B)
```

```
## # Effect Size for ANOVA
##
## Parameter    | Eta2 (partial) |       95% CI
## ---------------------------------------------
## bond_country |           0.01 | [0.01, 1.00]
## bond_gvmt    |       3.51e-03 | [0.00, 1.00]
## age          |           0.05 | [0.04, 1.00]
## gender       |       6.23e-03 | [0.00, 1.00]
## education    |       2.62e-03 | [0.00, 1.00]
## GDP          |           0.02 | [0.00, 1.00]
##
## - One-sided CIs: upper bound fixed at [1.00].
```

Hypothesis 2a, Dataset B model check for assumptions and fit

```r
# Test homogeneity of variance for the bonding variables and models with/out random effect
summary(bond_wellbeing_B_noRand <- lm(wellbeing_scale ~ bond_country + bond_gvmt  +
                                 age + gender + education + GDP,
                                 data=DatasetB_phase1, na.action=na.exclude, method="REML"))
```

```
##
## Call:
## lm(formula = wellbeing_scale ~ bond_country + bond_gvmt + age +
##     gender + education + GDP, data = DatasetB_phase1, na.action = na.exclude,
##     method = "REML")
##
## Residuals:
##     Min      1Q  Median      3Q     Max
## -3.8602 -0.6324 -0.0589  0.5604  3.6345
##
## Coefficients:
##                          Estimate Std. Error t value Pr(>|t|)
## (Intercept)            -3.217e-01  4.345e-02  -7.403 1.51e-13 ***
## bond_country1           3.496e-01  3.180e-02  10.995  < 2e-16 ***
## bond_gvmt1              2.696e-01  5.122e-02   5.263 1.46e-07 ***
## age                     1.186e-02  8.955e-04  13.247  < 2e-16 ***
## genderwoman            -1.704e-01  2.621e-02  -6.500 8.67e-11 ***
## gendernb               -3.287e-01  1.267e-01  -2.594  0.00952 **
## gendernone             -3.457e-01  1.434e-01  -2.410  0.01597 *
## educationundergraduate  6.107e-02  3.518e-02   1.736  0.08265 .
## educationpostgraduate   1.161e-01  3.721e-02   3.119  0.00182 **
## GDP                    -6.346e-06  6.153e-07 -10.313  < 2e-16 ***
## ---
## Signif. codes:  0 '***' 0.001 '**' 0.01 '*' 0.05 '.' 0.1 ' ' 1
##
## Residual standard error: 0.94 on 6269 degrees of freedom
##   (396 observations deleted due to missingness)
## Multiple R-squared:  0.0998, Adjusted R-squared:  0.09851
## F-statistic: 77.23 on 9 and 6269 DF,  p-value: < 2.2e-16
```

```r
summary(bond_wellbeing_B <- lme(wellbeing_scale ~ bond_country + bond_gvmt  +
                                 age + gender + education + GDP, (~1|country_now_Name),
                                 data=DatasetB_phase1, na.action=na.exclude, method="REML"))
```

```
## Linear mixed-effects model fit by REML
##   Data: DatasetB_phase1
##        AIC      BIC    logLik
##   16982.11 17063.03 -8479.055
##
## Random effects:
##  Formula: ~1 | country_now_Name
##         (Intercept)  Residual
## StdDev:   0.1955565 0.9248278
##
## Fixed effects:  wellbeing_scale ~ bond_country + bond_gvmt + age + gender + education +       GDP
```

```
##                             Value  Std.Error   DF   t-value p-value
## (Intercept)            -0.5438673 0.06353000 6164 -8.560796  0.0000
## bond_country1           0.2775811 0.03208196 6164  8.652250  0.0000
## bond_gvmt1              0.2391052 0.05110723 6164  4.678499  0.0000
## age                     0.0144023 0.00092128 6164 15.632938  0.0000
## genderwoman            -0.1601093 0.02631521 6164 -6.084287  0.0000
## gendernb               -0.2693887 0.12511700 6164 -2.153094  0.0313
## gendernone             -0.3328291 0.14141649 6164 -2.353538  0.0186
## educationundergraduate  0.0650355 0.03482212 6164  1.867650  0.0619
## educationpostgraduate   0.1423452 0.03742920 6164  3.803052  0.0001
## GDP                    -0.0000023 0.00000137  105 -1.683521  0.0952
##  Correlation:
##                        (Intr) bnd_c1 bnd_g1 age    gndrwm gndrnb gndrnn edctnn
## bond_country1          -0.033
## bond_gvmt1             -0.038 -0.357
## age                    -0.356 -0.194 -0.004
## genderwoman            -0.246 -0.035  0.035  0.086
## gendernb               -0.073  0.008  0.018  0.056  0.152
## gendernone             -0.065  0.016 -0.014  0.030  0.125  0.029
## educationundergraduate -0.336  0.012  0.009 -0.180 -0.029 -0.005  0.004
## educationpostgraduate  -0.296  0.050  0.006 -0.252 -0.046 -0.004  0.007  0.722
## GDP                    -0.515  0.054 -0.022 -0.077 -0.072 -0.020 -0.002  0.017
##                        edctnp
## bond_country1
## bond_gvmt1
## age
## genderwoman
## gendernb
## gendernone
## educationundergraduate
## educationpostgraduate
## GDP                    -0.007
##
## Standardized Within-Group Residuals:
##         Min          Q1         Med          Q3         Max
## -4.64407395 -0.67315188 -0.05850194  0.60401588  3.97507368
##
## Number of Observations: 6279
## Number of Groups: 107
```

```
anova(bond_wellbeing_B, bond_wellbeing_B_noRand)
```

```
##                         Model df      AIC      BIC    logLik   Test  L.Ratio
## bond_wellbeing_B            1 12 16982.11 17063.03 -8479.055
## bond_wellbeing_B_noRand     2 11 17130.35 17204.53 -8554.175 1 vs 2 150.2382
##                         p-value
## bond_wellbeing_B
## bond_wellbeing_B_noRand  <.0001
```

```
leveneTest(residuals(bond_wellbeing_B) ~ DatasetB_phase1$bond_country) # sig.
```

```
## Levene's Test for Homogeneity of Variance (center = median)
##         Df F value    Pr(>F)
```

```
## group    1  18.569 1.663e-05 ***
##       6277
## ---
## Signif. codes:  0 '***' 0.001 '**' 0.01 '*' 0.05 '.' 0.1 ' ' 1
```

```
leveneTest(residuals(bond_wellbeing_B) ~ DatasetB_phase1$bond_gvmt) # sig.
```

```
## Levene's Test for Homogeneity of Variance (center = median)
##         Df F value    Pr(>F)
## group    1  12.561 0.0003967 ***
##       6277
## ---
## Signif. codes:  0 '***' 0.001 '**' 0.01 '*' 0.05 '.' 0.1 ' ' 1
```

```
# Selection between model with homogeneous vs heterogeneous variances for bond_country and bond_gvmt
var_str <- varComb(varIdent(form=~ 1|bond_country), varIdent(form=~ 1|bond_gvmt))
summary(bond_wellbeing_B_homo <- lme(wellbeing_scale ~ 1,
                                     random=list(country_now_Name=pdDiag(form=~bond_country+bond_gvmt))
                                     data=DatasetB_phase1, na.action=na.exclude, method="REML"))
```

```
## Linear mixed-effects model fit by REML
##   Data: DatasetB_phase1
##        AIC      BIC   logLik
##   17345.56 17379.29 -8667.78
##
## Random effects:
##  Formula: ~bond_country + bond_gvmt | country_now_Name
##  Structure: Diagonal
##         (Intercept) bond_country1 bond_gvmt1  Residual
## StdDev:   0.1792804     0.4140032  0.3530829 0.9497174
##
## Fixed effects:  wellbeing_scale ~ 1
##                   Value  Std.Error   DF   t-value p-value
## (Intercept) -0.08678218 0.03441842 6179 -2.521387  0.0117
##
## Standardized Within-Group Residuals:
##        Min         Q1        Med         Q3        Max
## -4.1400563 -0.6694166 -0.0398822  0.6370689  3.7033819
##
## Number of Observations: 6291
## Number of Groups: 112
```

```
summary(bond_wellbeing_B_hetero <- lme(wellbeing_scale ~ 1,
                                     random=list(country_now_Name=pdDiag(form=~ bond_country+bond_gvmt)
                                     weights=var_str,
                                     data=DatasetB_phase1, na.action=na.exclude, method="REML"))
```

```
## Linear mixed-effects model fit by REML
##   Data: DatasetB_phase1
##        AIC      BIC    logLik
##   17324.08 17371.31 -8655.041
##
```

```
## Random effects:
##  Formula: ~bond_country + bond_gvmt | country_now_Name
##  Structure: Diagonal
##         (Intercept) bond_country1 bond_gvmt1 Residual
## StdDev:   0.1799733     0.3993436  0.2778503 0.929672
##
## Combination of variance functions:
##  Structure: Different standard deviations per stratum
##  Formula: ~1 | bond_country
##  Parameter estimates:
##        0        1
## 1.000000 1.057656
##  Structure: Different standard deviations per stratum
##  Formula: ~1 | bond_gvmt
##  Parameter estimates:
##        0        1
## 1.000000 1.127398
## Fixed effects:  wellbeing_scale ~ 1
##                   Value  Std.Error   DF   t-value p-value
## (Intercept) -0.08951078 0.03425918 6179 -2.612753   0.009
##
## Standardized Within-Group Residuals:
##         Min          Q1         Med          Q3         Max
## -4.12735759 -0.68398882 -0.02915613  0.65066693  3.78339740
##
## Number of Observations: 6291
## Number of Groups: 112
```

```
VarCorr(bond_wellbeing_B_homo)
```

```
## country_now_Name = pdDiag(bond_country + bond_gvmt)
##               Variance   StdDev
## (Intercept)   0.03214146 0.1792804
## bond_country1 0.17139862 0.4140032
## bond_gvmt1    0.12466754 0.3530829
## Residual      0.90196317 0.9497174
```

```
VarCorr(bond_wellbeing_B_hetero)
```

```
## country_now_Name = pdDiag(bond_country + bond_gvmt)
##               Variance   StdDev
## (Intercept)   0.03239039 0.1799733
## bond_country1 0.15947529 0.3993436
## bond_gvmt1    0.07720077 0.2778503
## Residual      0.86428995 0.9296720
```

```
anova(bond_wellbeing_B_homo,bond_wellbeing_B_hetero) # Heterogeneous model is significantly better (low
```

```
##                         Model df      AIC      BIC    logLik   Test  L.Ratio
## bond_wellbeing_B_homo       1  5 17345.56 17379.29 -8667.780
## bond_wellbeing_B_hetero     2  7 17324.08 17371.31 -8655.041 1 vs 2 25.47747
##                         p-value
## bond_wellbeing_B_homo
## bond_wellbeing_B_hetero  <.0001
```

Table S8A: Hypothesis 2b, Dataset A: Does bonding with multiple groups predict mental health?

Hypothesis 2b, Dataset A model

```
# Anxiety
summary(bond_mult_anx_A <- lme(anx_sum1_scaled ~ bond_mult  +
                                demo_age + demo_gender + demo_education + GDP,
                                (~1|Country_Name), data=DatasetA, na.action=na.exclude))
```

```
## Linear mixed-effects model fit by REML
##   Data: DatasetA
##        AIC      BIC    logLik
##   17746.15 17841.16 -8859.074
##
## Random effects:
##  Formula: ~1 | Country_Name
##         (Intercept)  Residual
## StdDev:   0.2375083 0.9259253
##
## Fixed effects:  anx_sum1_scaled ~ bond_mult + demo_age + demo_gender + demo_education +      GDP
##                                  Value  Std.Error   DF   t-value p-value
## (Intercept)                  0.0134211 0.09624908 6475  0.139441  0.8891
## bond_mult1                  -0.1339047 0.02664830 6475 -5.024888  0.0000
## bond_mult2                  -0.2126759 0.03911758 6475 -5.436837  0.0000
## bond_mult3                  -0.2973131 0.06391507 6475 -4.651690  0.0000
## bond_mult4                  -0.3769852 0.10526485 6475 -3.581302  0.0003
## demo_age                    -0.0085980 0.00093006 6475 -9.244486  0.0000
## demo_genderFemale            0.4621824 0.02399504 6475 19.261583  0.0000
## demo_genderOther/non-binary  0.5330370 0.12147724 6475  4.387958  0.0000
## demo_educationundergraduate -0.0291088 0.02907059 6475 -1.001316  0.3167
## demo_educationpostgraduate  -0.0282964 0.03162274 6475 -0.894813  0.3709
## demo_educationNA             0.1820697 0.13505378 6475  1.348127  0.1777
## GDP                         -0.0000014 0.00000202   70 -0.704838  0.4832
##  Correlation:
##                             (Intr) bnd_m1 bnd_m2 bnd_m3 bnd_m4 demo_g dm_gnF
## bond_mult1                  -0.084
## bond_mult2                  -0.025  0.257
## bond_mult3                  -0.011  0.167  0.130
## bond_mult4                  -0.006  0.110  0.090  0.072
## demo_age                    -0.313 -0.074 -0.110 -0.123 -0.121
## demo_genderFemale           -0.114 -0.029 -0.059 -0.051 -0.030 -0.024
## demo_genderOther/non-binary -0.025 -0.003 -0.011  0.004 -0.002  0.036  0.114
## demo_educationundergraduate -0.157  0.016  0.004  0.027  0.007 -0.073 -0.019
## demo_educationpostgraduate  -0.122  0.005  0.001  0.015  0.034 -0.165 -0.041
## demo_educationNA            -0.050 -0.008 -0.007 -0.055 -0.019 -0.016 -0.015
## GDP                         -0.744  0.022  0.004  0.016  0.020 -0.002 -0.011
##                             dm_O/- dm_dctnn dm_dctnp dm_dNA
## bond_mult1
## bond_mult2
## bond_mult3
## bond_mult4
```

```
## demo_age
## demo_genderFemale
## demo_genderOther/non-binary
## demo_educationundergraduate  0.016
## demo_educationpostgraduate   0.005  0.581
## demo_educationNA             0.005  0.122    0.119
## GDP                         -0.034 -0.012   -0.011    0.015
##
## Standardized Within-Group Residuals:
##         Min          Q1         Med          Q3         Max
## -2.60811648 -0.74114433 -0.07723895  0.65564566  3.59348328
##
## Number of Observations: 6557
## Number of Groups: 72
```

```
anova.lme(bond_mult_anx_A,type="marginal")
```

```
##                  numDF denDF   F-value p-value
## (Intercept)          1  6475   0.01944  0.8891
## bond_mult            4  6475  15.65270  <.0001
## demo_age             1  6475  85.46053  <.0001
## demo_gender          2  6475 187.95278  <.0001
## demo_education       3  6475   1.14010  0.3313
## GDP                  1    70   0.49680  0.4832
```

```
r2_nakagawa(bond_mult_anx_A)
```

```
## # R2 for Mixed Models
##
##   Conditional R2: 0.129
##      Marginal R2: 0.072
```

```
eta_squared(bond_mult_anx_A)
```

```
## # Effect Size for ANOVA
##
## Parameter      | Eta2 (partial) |       95% CI
## -----------------------------------------------
## bond_mult      |           0.01 | [0.01, 1.00]
## demo_age       |           0.01 | [0.01, 1.00]
## demo_gender    |           0.05 | [0.05, 1.00]
## demo_education |       5.37e-04 | [0.00, 1.00]
## GDP            |       7.05e-03 | [0.00, 1.00]
##
## - One-sided CIs: upper bound fixed at [1.00].
```

```
# Depression
summary(bond_mult_dep_A <- lme(dep_sum1_scaled ~ bond_mult  +
                                 demo_age + demo_gender + demo_education + GDP,
                                 random=list(Country_Name=pdDiag(form=~ factor(bond_mult))),
                                 weights=varIdent(form=~ 1|bond_mult),data=DatasetA, na.action=na.exclude
```

```
## Linear mixed-effects model fit by REML
##   Data: DatasetA
##        AIC      BIC    logLik
##   18156.03 18305.34 -9056.015
##
## Random effects:
##  Formula: ~factor(bond_mult) | Country_Name
##  Structure: Diagonal
##         (Intercept) factor(bond_mult)1 factor(bond_mult)2 factor(bond_mult)3
## StdDev:      0.1984       2.980597e-05       0.0001094949       0.0003762482
##         factor(bond_mult)4  Residual
## StdDev:          0.1351186 0.9752519
##
## Variance function:
##  Structure: Different standard deviations per stratum
##  Formula: ~1 | bond_mult
##  Parameter estimates:
##         0         1         4         2         3
## 1.0000000 0.9765285 0.8901030 0.9042453 0.9309116
## Fixed effects:  dep_sum1_scaled ~ bond_mult + demo_age + demo_gender + demo_education +      GDP
##                                  Value  Std.Error   DF    t-value p-value
## (Intercept)                  0.2746124 0.09218658 6476   2.978876  0.0029
## bond_mult1                  -0.1966743 0.02761584 6476  -7.121793  0.0000
## bond_mult2                  -0.3864548 0.03802451 6476 -10.163307  0.0000
## bond_mult3                  -0.4485376 0.06304136 6476  -7.114973  0.0000
## bond_mult4                  -0.6766726 0.11326960 6476  -5.974001  0.0000
## demo_age                    -0.0055287 0.00095299 6476  -5.801411  0.0000
## demo_genderFemale            0.2207383 0.02472235 6476   8.928693  0.0000
## demo_genderOther/non-binary  0.5077028 0.12565632 6476   4.040408  0.0001
## demo_educationundergraduate -0.1126459 0.02994362 6476  -3.761935  0.0002
## demo_educationpostgraduate  -0.1292046 0.03251558 6476  -3.973621  0.0001
## demo_educationNA             0.0507289 0.13698211 6476   0.370333  0.7111
## GDP                         -0.0000032 0.00000191   70  -1.664829  0.1004
##  Correlation:
##                             (Intr) bnd_m1 bnd_m2 bnd_m3 bnd_m4 demo_g dm_gnF
## bond_mult1                  -0.095
## bond_mult2                  -0.033  0.281
## bond_mult3                  -0.015  0.180  0.149
## bond_mult4                  -0.013  0.106  0.091  0.070
## demo_age                    -0.335 -0.074 -0.117 -0.128 -0.116
## demo_genderFemale           -0.120 -0.029 -0.063 -0.053 -0.030 -0.026
## demo_genderOther/non-binary -0.028 -0.003 -0.012  0.004 -0.001  0.036  0.113
## demo_educationundergraduate -0.171  0.016  0.004  0.028  0.007 -0.071 -0.017
## demo_educationpostgraduate  -0.132  0.006  0.001  0.016  0.032 -0.161 -0.039
## demo_educationNA            -0.050 -0.008 -0.006 -0.056 -0.014 -0.017 -0.016
## GDP                         -0.747  0.026  0.007  0.019  0.025 -0.004 -0.014
##                             dm_O/- dm_dctnn dm_dctnp dm_dNA
## bond_mult1
## bond_mult2
## bond_mult3
## bond_mult4
## demo_age
## demo_genderFemale
## demo_genderOther/non-binary
```

```
## demo_educationundergraduate  0.016
## demo_educationpostgraduate   0.005  0.581
## demo_educationNA             0.005  0.123    0.120
## GDP                         -0.033 -0.012   -0.010    0.014
##
## Standardized Within-Group Residuals:
##         Min          Q1         Med          Q3         Max
## -2.55549335 -0.76006921 -0.07397742  0.67399218  4.36252446
##
## Number of Observations: 6558
## Number of Groups: 72
```

```
anova.lme(bond_mult_dep_A,type="marginal")
```

```
##                 numDF denDF  F-value p-value
## (Intercept)         1  6476  8.87370  0.0029
## bond_mult           4  6476 42.23049  <.0001
## demo_age            1  6476 33.65637  <.0001
## demo_gender         2  6476 44.50249  <.0001
## demo_education      3  6476  6.64102  0.0002
## GDP                 1    70  2.77166  0.1004
```

```
r2_nakagawa(bond_mult_dep_A)
```

```
## [1] NA
```

```
eta_squared(bond_mult_dep_A)
```

```
## # Effect Size for ANOVA
##
## Parameter      | Eta2 (partial) |       95% CI
## ----------------------------------------------
## bond_mult      |           0.03 | [0.02, 1.00]
## demo_age       |       6.16e-03 | [0.00, 1.00]
## demo_gender    |           0.01 | [0.01, 1.00]
## demo_education |       3.10e-03 | [0.00, 1.00]
## GDP            |           0.04 | [0.00, 1.00]
##
## - One-sided CIs: upper bound fixed at [1.00].
```

Hypothesis 2b, Dataset A model check for assumptions and fit

```
# Anxiety
# Test homogeneity of variance for the bonding variables and models with/out random effect
bond_mult_anx_A_noRand <- lm(anx_sum1_scaled ~ bond_mult  +
                                demo_age + demo_gender + demo_education + GDP,
                             data=DatasetA, na.action=na.exclude)
bond_mult_anx_A_homo <- lme(anx_sum1_scaled ~ bond_mult  +
                                demo_age + demo_gender + demo_education + GDP,
                                (~1|Country_Name), data=DatasetA, na.action=na.exclude)
anova(bond_mult_anx_A_homo, bond_mult_anx_A_noRand)
```

```
##                         Model df      AIC      BIC    logLik   Test  L.Ratio
## bond_mult_anx_A_homo       1 14 17746.15 17841.16 -8859.074
## bond_mult_anx_A_noRand     2 13 18139.27 18227.49 -9056.636 1 vs 2 395.1233
##                         p-value
## bond_mult_anx_A_homo
## bond_mult_anx_A_noRand  <.0001
```

```
leveneTest(residuals(bond_mult_anx_A) ~ DatasetA$bond_mult)
```

```
## Levene's Test for Homogeneity of Variance (center = median)
##         Df F value Pr(>F)
## group    4  0.6283 0.6423
##       6552
```

```
# Depression
# Test homogeneity of variance for the bonding variables and models with/out random effect
summary(bond_mult_dep_A_noRand <- lm(dep_sum1_scaled ~ bond_mult  +
                                demo_age + demo_gender + demo_education + GDP,
                                data=DatasetA, na.action=na.exclude))
```

```
##
## Call:
## lm(formula = dep_sum1_scaled ~ bond_mult + demo_age + demo_gender +
##     demo_education + GDP, data = DatasetA, na.action = na.exclude)
##
## Residuals:
##     Min      1Q  Median      3Q     Max
## -2.1959 -0.7497 -0.1033  0.6485  3.7646
##
## Coefficients:
##                               Estimate Std. Error t value Pr(>|t|)
## (Intercept)                  3.427e-01  4.917e-02   6.970 3.49e-12 ***
## bond_mult1                  -1.710e-01  2.790e-02  -6.131 9.24e-10 ***
## bond_mult2                  -3.613e-01  4.099e-02  -8.814  < 2e-16 ***
## bond_mult3                  -4.536e-01  6.705e-02  -6.766 1.44e-11 ***
## bond_mult4                  -6.942e-01  1.099e-01  -6.317 2.84e-10 ***
## demo_age                    -4.287e-03  9.342e-04  -4.589 4.54e-06 ***
## demo_genderFemale            2.529e-01  2.501e-02  10.113  < 2e-16 ***
## demo_genderOther/non-binary  5.249e-01  1.275e-01   4.116 3.90e-05 ***
## demo_educationundergraduate -1.853e-01  2.987e-02  -6.202 5.93e-10 ***
## demo_educationpostgraduate  -2.092e-01  3.264e-02  -6.408 1.58e-10 ***
## demo_educationNA             6.211e-02  1.416e-01   0.439  0.66102
## GDP                         -1.858e-06  6.394e-07  -2.906  0.00367 **
## ---
## Signif. codes:  0 '***' 0.001 '**' 0.01 '*' 0.05 '.' 0.1 ' ' 1
##
## Residual standard error: 0.975 on 6546 degrees of freedom
##   (31 observations deleted due to missingness)
## Multiple R-squared:  0.04988,    Adjusted R-squared:  0.04829
## F-statistic: 31.24 on 11 and 6546 DF,  p-value: < 2.2e-16
```

```r
summary(bond_mult_dep_A_homo <- lme(dep_sum1_scaled ~ bond_mult  +
                                demo_age + demo_gender + demo_education + GDP,
                                (~1|Country_Name), data=DatasetA, na.action=na.exclude))
```

```
## Linear mixed-effects model fit by REML
##   Data: DatasetA
##        AIC      BIC    logLik
##   18154.04 18249.05 -9063.021
##
## Random effects:
##  Formula: ~1 | Country_Name
##         (Intercept)  Residual
## StdDev:   0.1987686 0.9556372
##
## Fixed effects:  dep_sum1_scaled ~ bond_mult + demo_age + demo_gender + demo_education +      GDP
##                                   Value  Std.Error   DF   t-value p-value
## (Intercept)                   0.2802414 0.09225449 6476  3.037699  0.0024
## bond_mult1                   -0.1963031 0.02749191 6476 -7.140395  0.0000
## bond_mult2                   -0.3857163 0.04038899 6476 -9.550037  0.0000
## bond_mult3                   -0.4474971 0.06594151 6476 -6.786273  0.0000
## bond_mult4                   -0.6372323 0.10857830 6476 -5.868873  0.0000
## demo_age                     -0.0055242 0.00095904 6476 -5.760092  0.0000
## demo_genderFemale             0.2194497 0.02474813 6476  8.867323  0.0000
## demo_genderOther/non-binary   0.5216685 0.12529453 6476  4.163538  0.0000
## demo_educationundergraduate -0.1160633 0.02999233 6476 -3.869766  0.0001
## demo_educationpostgraduate   -0.1342683 0.03261771 6476 -4.116423  0.0000
## demo_educationNA              0.0318211 0.13927866 6476  0.228471  0.8193
## GDP                          -0.0000033 0.00000191   70 -1.720735  0.0897
##  Correlation:
##                              (Intr) bnd_m1 bnd_m2 bnd_m3 bnd_m4 demo_g dm_gnF
## bond_mult1                   -0.091
## bond_mult2                   -0.028  0.257
## bond_mult3                   -0.013  0.167  0.130
## bond_mult4                   -0.007  0.110  0.089  0.071
## demo_age                     -0.336 -0.075 -0.111 -0.123 -0.122
## demo_genderFemale            -0.120 -0.030 -0.059 -0.051 -0.030 -0.024
## demo_genderOther/non-binary -0.028 -0.003 -0.011  0.004 -0.002  0.036  0.114
## demo_educationundergraduate -0.170  0.016  0.004  0.027  0.006 -0.073 -0.018
## demo_educationpostgraduate   -0.130  0.006  0.002  0.016  0.034 -0.165 -0.040
## demo_educationNA             -0.051 -0.008 -0.006 -0.055 -0.019 -0.016 -0.015
## GDP                          -0.747  0.026  0.007  0.018  0.024 -0.005 -0.014
##                              dm_O/- dm_dctnn dm_dctnp dm_dNA
## bond_mult1
## bond_mult2
## bond_mult3
## bond_mult4
## demo_age
## demo_genderFemale
## demo_genderOther/non-binary
## demo_educationundergraduate  0.016
## demo_educationpostgraduate   0.005  0.582
## demo_educationNA             0.006  0.122    0.119
## GDP                          -0.033 -0.012   -0.010    0.015
```

```
##
## Standardized Within-Group Residuals:
##         Min          Q1         Med          Q3         Max
## -2.60546264 -0.76390311 -0.07493976  0.67258462  4.05756346
##
## Number of Observations: 6558
## Number of Groups: 72
```

```
anova(bond_mult_dep_A_homo, bond_mult_dep_A_noRand)
```

```
##                        Model df      AIC      BIC    logLik   Test  L.Ratio
## bond_mult_dep_A_homo       1 14 18154.04 18249.05 -9063.021
## bond_mult_dep_A_noRand     2 13 18375.33 18463.56 -9174.664 1 vs 2 223.2869
##                        p-value
## bond_mult_dep_A_homo
## bond_mult_dep_A_noRand  <.0001
```

```
leveneTest(residuals(bond_mult_dep_A_homo) ~ DatasetA$bond_mult)
```

```
## Levene's Test for Homogeneity of Variance (center = median)
##         Df F value    Pr(>F)
## group    4   5.007 0.0004993 ***
##       6553
## ---
## Signif. codes:  0 '***' 0.001 '**' 0.01 '*' 0.05 '.' 0.1 ' ' 1
```

```
summary(bond_mult_dep_A_hetero <- lme(dep_sum1_scaled ~ bond_mult  +
                                 demo_age + demo_gender + demo_education + GDP,
                                 random=list(Country_Name=pdDiag(form=~ factor(bond_mult))),
                                 weights=varIdent(form=~ 1|bond_mult),data=DatasetA, na.action=na.exclud
```

```
## Linear mixed-effects model fit by REML
##   Data: DatasetA
##        AIC      BIC    logLik
##   18156.03 18305.34 -9056.015
##
## Random effects:
##  Formula: ~factor(bond_mult) | Country_Name
##  Structure: Diagonal
##         (Intercept) factor(bond_mult)1 factor(bond_mult)2 factor(bond_mult)3
## StdDev:      0.1984       2.980597e-05       0.0001094949       0.0003762482
##         factor(bond_mult)4  Residual
## StdDev:          0.1351186 0.9752519
##
## Variance function:
##  Structure: Different standard deviations per stratum
##  Formula: ~1 | bond_mult
##  Parameter estimates:
##         0         1         4         2         3
## 1.0000000 0.9765285 0.8901030 0.9042453 0.9309116
## Fixed effects:  dep_sum1_scaled ~ bond_mult + demo_age + demo_gender + demo_education +      GDP
##                                  Value  Std.Error   DF    t-value p-value
```

```
## (Intercept)                  0.2746124 0.09218658 6476   2.978876  0.0029
## bond_mult1                  -0.1966743 0.02761584 6476  -7.121793  0.0000
## bond_mult2                  -0.3864548 0.03802451 6476 -10.163307  0.0000
## bond_mult3                  -0.4485376 0.06304136 6476  -7.114973  0.0000
## bond_mult4                  -0.6766726 0.11326960 6476  -5.974001  0.0000
## demo_age                    -0.0055287 0.00095299 6476  -5.801411  0.0000
## demo_genderFemale            0.2207383 0.02472235 6476   8.928693  0.0000
## demo_genderOther/non-binary  0.5077028 0.12565632 6476   4.040408  0.0001
## demo_educationundergraduate -0.1126459 0.02994362 6476  -3.761935  0.0002
## demo_educationpostgraduate  -0.1292046 0.03251558 6476  -3.973621  0.0001
## demo_educationNA             0.0507289 0.13698211 6476   0.370333  0.7111
## GDP                         -0.0000032 0.00000191   70  -1.664829  0.1004
##  Correlation:
##                             (Intr) bnd_m1 bnd_m2 bnd_m3 bnd_m4 demo_g dm_gnF
## bond_mult1                  -0.095
## bond_mult2                  -0.033  0.281
## bond_mult3                  -0.015  0.180  0.149
## bond_mult4                  -0.013  0.106  0.091  0.070
## demo_age                    -0.335 -0.074 -0.117 -0.128 -0.116
## demo_genderFemale           -0.120 -0.029 -0.063 -0.053 -0.030 -0.026
## demo_genderOther/non-binary -0.028 -0.003 -0.012  0.004 -0.001  0.036  0.113
## demo_educationundergraduate -0.171  0.016  0.004  0.028  0.007 -0.071 -0.017
## demo_educationpostgraduate  -0.132  0.006  0.001  0.016  0.032 -0.161 -0.039
## demo_educationNA            -0.050 -0.008 -0.006 -0.056 -0.014 -0.017 -0.016
## GDP                         -0.747  0.026  0.007  0.019  0.025 -0.004 -0.014
##                             dm_O/- dm_dctnn dm_dctnp dm_dNA
## bond_mult1
## bond_mult2
## bond_mult3
## bond_mult4
## demo_age
## demo_genderFemale
## demo_genderOther/non-binary
## demo_educationundergraduate  0.016
## demo_educationpostgraduate   0.005  0.581
## demo_educationNA             0.005  0.123    0.120
## GDP                         -0.033 -0.012   -0.010    0.014
##
## Standardized Within-Group Residuals:
##         Min          Q1         Med          Q3         Max
## -2.55549335 -0.76006921 -0.07397742  0.67399218  4.36252446
##
## Number of Observations: 6558
## Number of Groups: 72
```

```
VarCorr(bond_mult_dep_A_homo)
```

```
## Country_Name = pdLogChol(1)
##             Variance   StdDev
## (Intercept) 0.03950897 0.1987686
## Residual    0.91324243 0.9556372
```

```r
VarCorr(bond_mult_dep_A_hetero)
```

```
## Country_Name = pdDiag(factor(bond_mult))
##                     Variance     StdDev
## (Intercept)         3.936258e-02 1.984000e-01
## factor(bond_mult)1 8.883961e-10 2.980597e-05
## factor(bond_mult)2 1.198913e-08 1.094949e-04
## factor(bond_mult)3 1.415627e-07 3.762482e-04
## factor(bond_mult)4 1.825705e-02 1.351186e-01
## Residual           9.511163e-01 9.752519e-01
```

```r
anova(bond_mult_dep_A_homo,bond_mult_dep_A_hetero) # No significant difference between models. Heteroge
```

```
##                          Model df      AIC      BIC    logLik   Test  L.Ratio
## bond_mult_dep_A_homo         1 14 18154.04 18249.05 -9063.021
## bond_mult_dep_A_hetero       2 22 18156.03 18305.33 -9056.015 1 vs 2 14.01217
##                          p-value
## bond_mult_dep_A_homo
## bond_mult_dep_A_hetero  0.0814
```

Table S8B: Hypothesis 2b, Dataset B: Does bonding with multiple groups predict wellbeing?

Hypothesis 2b, Dataset B model

```r
# Wellbeing
summary(bond_mult_wellbeing_B <- lme(wellbeing_scale ~ bond_mult  +
                                age + gender + education + GDP,
                                random=list(country_now_Name=pdDiag(form=~ factor(bond_mult))),
                                weights=varIdent(form=~ 1|bond_mult), data=DatasetB_phase1, na.action=na
```

```
## Linear mixed-effects model fit by REML
##   Data: DatasetB_phase1
##        AIC     BIC    logLik
##   17536.46 17644.9 -8752.228
##
## Random effects:
##  Formula: ~factor(bond_mult) | country_now_Name
##  Structure: Diagonal
##         (Intercept) factor(bond_mult)1 factor(bond_mult)2  Residual
## StdDev:   0.2003617          0.1262106         0.05430643 0.9535381
##
## Variance function:
##  Structure: Different standard deviations per stratum
##  Formula: ~1 | bond_mult
##  Parameter estimates:
##         1         0         2
## 1.0000000 0.9477848 1.1346890
## Fixed effects:  wellbeing_scale ~ bond_mult + age + gender + education + GDP
##                              Value  Std.Error   DF   t-value p-value
```

```
## (Intercept)            -0.5526359 0.06362244 6381 -8.686178  0.0000
## bond_mult1              0.2564828 0.04729141 6381  5.423454  0.0000
## bond_mult2              0.5147199 0.06396153 6381  8.047335  0.0000
## age                     0.0144540 0.00090545 6381 15.963347  0.0000
## genderwoman            -0.1500427 0.02566432 6381 -5.846353  0.0000
## gendernb               -0.2922577 0.12064701 6381 -2.422419  0.0154
## gendernone             -0.3237939 0.13842669 6381 -2.339100  0.0194
## educationundergraduate  0.0677278 0.03396674 6381  1.993945  0.0462
## educationpostgraduate   0.1406880 0.03666714 6381  3.836897  0.0001
## GDP                    -0.0000022 0.00000138  106 -1.621904  0.1078
##  Correlation:
##                        (Intr) bnd_m1 bnd_m2 age    gndrwm gndrnb gndrnn edctnn
## bond_mult1             -0.093
## bond_mult2             -0.046  0.093
## age                    -0.355 -0.105 -0.085
## genderwoman            -0.242 -0.007  0.013  0.082
## gendernb               -0.074  0.011  0.017  0.056  0.153
## gendernone             -0.065  0.003  0.000  0.032  0.125  0.030
## educationundergraduate -0.322  0.000  0.011 -0.185 -0.030 -0.003  0.007
## educationpostgraduate  -0.281  0.026  0.024 -0.259 -0.046 -0.003  0.008  0.720
## GDP                    -0.525  0.042  0.014 -0.065 -0.065 -0.018 -0.002  0.015
##                        edctnp
## bond_mult1
## bond_mult2
## age
## genderwoman
## gendernb
## gendernone
## educationundergraduate
## educationpostgraduate
## GDP                    -0.011
##
## Standardized Within-Group Residuals:
##         Min          Q1         Med          Q3         Max
## -4.79230140 -0.67512017 -0.05782256  0.60372490  4.06418943
##
## Number of Observations: 6497
## Number of Groups: 108
```

```
anova.lme(bond_mult_wellbeing_B,type="marginal")
```

```
##             numDF denDF   F-value p-value
## (Intercept)     1  6381  75.44969  <.0001
## bond_mult       2  6381  43.40212  <.0001
## age             1  6381 254.82844  <.0001
## gender          3  6381  13.04725  <.0001
## education       2  6381   7.97157  0.0003
## GDP             1   106   2.63057  0.1078
```

```
r2_nakagawa(bond_mult_wellbeing_B)
```

```
## [1] NA
```

```
eta_squared(bond_mult_wellbeing_B)
```

```
## # Effect Size for ANOVA
##
## Parameter | Eta2 (partial) |       95% CI
## -----------------------------------------
## bond_mult |           0.02 | [0.02, 1.00]
## age       |           0.05 | [0.04, 1.00]
## gender    |       6.02e-03 | [0.00, 1.00]
## education |       2.45e-03 | [0.00, 1.00]
## GDP       |           0.02 | [0.00, 1.00]
##
## - One-sided CIs: upper bound fixed at [1.00].
```

Hypothesis 2b, Dataset B model check for assumptions and fit

```
# Test homogeneity of variance for the bonding variables and models with/out random effect
bond_mult_wellbeing_B_noRand <- lm(wellbeing_scale ~ bond_mult  +
                                age + gender + education + GDP,
                                data=DatasetB_phase1, na.action=na.exclude)

bond_mult_wellbeing_B_homo <- lme(wellbeing_scale ~ bond_mult  +
                                age + gender + education + GDP,
                                (~1|country_now_Name), data=DatasetB_phase1, na.action=na.exclude)
anova(bond_mult_wellbeing_B_homo, bond_mult_wellbeing_B_noRand)
```

```
##                              Model df      AIC      BIC    logLik   Test
## bond_mult_wellbeing_B_homo       1 12 17558.67 17640.00 -8767.337
## bond_mult_wellbeing_B_noRand     2 11 17722.63 17797.18 -8850.313 1 vs 2
##                               L.Ratio p-value
## bond_mult_wellbeing_B_homo
## bond_mult_wellbeing_B_noRand 165.9521  <.0001
```

```
leveneTest(residuals(bond_mult_wellbeing_B_homo) ~ DatasetB_phase1$bond_mult) # sig.
```

```
## Levene's Test for Homogeneity of Variance (center = median)
##         Df F value    Pr(>F)
## group    2  11.569 9.648e-06 ***
##       6494
## ---
## Signif. codes:  0 '***' 0.001 '**' 0.01 '*' 0.05 '.' 0.1 ' ' 1
```

```
bond_mult_wellbeing_B_hetero <- lme(wellbeing_scale ~ bond_mult  +
                                age + gender + education + GDP,
                                random=list(country_now_Name=pdDiag(form=~ factor(bond_mult))),
                                weights=varIdent(form=~ 1|bond_mult), data=DatasetB_phase1, na.action=na


# Selection between model with homogeneous vs heterogeneous variances for bond_country
VarCorr(bond_mult_wellbeing_B_homo)
```

```
## country_now_Name = pdLogChol(1)
##             Variance   StdDev
## (Intercept) 0.04087878 0.2021850
## Residual    0.85384743 0.9240387
```

```
VarCorr(bond_mult_wellbeing_B_hetero)
```

```
## country_now_Name = pdDiag(factor(bond_mult))
##                    Variance    StdDev
## (Intercept)        0.040144823 0.20036173
## factor(bond_mult)1 0.015929113 0.12621059
## factor(bond_mult)2 0.002949189 0.05430643
## Residual           0.909234911 0.95353810
```

```
anova(bond_mult_wellbeing_B_homo,bond_mult_wellbeing_B_hetero) #  Heterogenous model significantly bett
```

```
##                              Model df      AIC     BIC    logLik   Test
## bond_mult_wellbeing_B_homo       1 12 17558.67 17640.0 -8767.337
## bond_mult_wellbeing_B_hetero     2 16 17536.46 17644.9 -8752.228 1 vs 2
##                               L.Ratio p-value
## bond_mult_wellbeing_B_homo
## bond_mult_wellbeing_B_hetero 30.21711  <.0001
```

Supplementary Analyses

Table S9A: Bonding variables as random slopes, Dataset A

Hypothesis 1a, Dataset A

None of Hypothesis 1a models converged for Dataset A.

```
# Distancing
summary(bond_distancing_A_slope<- lme(Pro_Distancing_scaled ~ bond_family + bond_friend +
                                  bond_country + bond_humanity  +
                                 demo_age + demo_gender + demo_education + GDP,
                                 (~bond_family + bond_friend +
                                  bond_country + bond_humanity|Country_Name), data=DatasetA, na.action=

# Hygiene
summary(bond_hygiene_A_slope<- lme(Pro_Hygiene_scaled ~ bond_family + bond_friend +
                                  bond_country + bond_humanity  +
                                  demo_age + demo_gender + demo_education + GDP,
                                  (~bond_family + bond_friend +
                                  bond_country + bond_humanity|Country_Name), data=DatasetA, na.action=

# Masking
summary(bond_masking_A_slope<- lme(Pro_MaskWearing_scaled ~ bond_family + bond_friend +
                               bond_country + bond_humanity  +
                               demo_age + demo_gender + demo_education + GDP,
                               (~bond_family + bond_friend +
                                  bond_country + bond_humanity|Country_Name), data=DatasetA, na.action=
```

Hypothesis 1b, Dataset A

None of Hypothesis 1b models converged for Dataset A.

```
# Distancing
summary(bond_mult_distancing_A_slope<- lme(Pro_Distancing_scaled ~ bond_mult  +
                                demo_age + demo_gender + demo_education + GDP,
                                (~bond_mult|Country_Name), data=DatasetA, na.action=na.exclude))
anova.lme(bond_mult_distancing_A_slope,type="marginal")

# Hygiene
summary(bond_mult_hygiene_A_slope<- lme(Pro_Hygiene_scaled ~ bond_mult  +
                                 demo_age + demo_gender + demo_education + GDP,
                                 (~bond_mult|Country_Name), data=DatasetA, na.action=na.exclude))
anova.lme(bond_mult_hygiene_A_slope,type="marginal")

# Masking
summary(bond_mult_masking_A_slope<- lme(Pro_MaskWearing_scaled ~ bond_mult  +
                              demo_age + demo_gender + demo_education + GDP,
                              (~bond_mult|Country_Name), data=DatasetA, na.action=na.exclude))
anova.lme(bond_mult_masking_A_slope,type="marginal")
```

Hypothesis 2a, Dataset A

None of Hypothesis 2a models converged for Dataset A.

```
# Anxiety
summary(bond_anx_A_slope<- lme(anx_sum1_scaled ~ bond_family + bond_friend +
                                  bond_country + bond_humanity  +
                                 demo_age + demo_gender + demo_education + GDP,
                                 (~bond_family + bond_friend +
                                  bond_country + bond_humanity|Country_Name), data=DatasetA, na.action=

# Depression
summary(bond_dep_A_slope<- lme(dep_sum1_scaled ~ bond_family + bond_friend +
                                  bond_country + bond_humanity  +
                                  demo_age + demo_gender + demo_education + GDP,
                                  (~bond_family + bond_friend +
                                  bond_country + bond_humanity|Country_Name), data=DatasetA, na.action=
```

Hypothesis 2b, Dataset A

None of Hypothesis 2b models converged for Dataset A.

```
# Anxiety
summary(bond_mult_anx_A_slope<- lme(anx_sum1_scaled ~ bond_mult  +
                                demo_age + demo_gender + demo_education + GDP,
                                (~bond_mult|Country_Name), data=DatasetA, na.action=na.exclude))
anova.lme(bond_mult_anx_A_slope,type="marginal")

# Depression
summary(bond_mult_dep_A_slope<- lme(dep_sum1_scaled ~ bond_mult  +
```

```
                                  demo_age + demo_gender + demo_education + GDP,
                                  (~bond_mult|Country_Name), data=DatasetA, na.action=na.exclude))
anova.lme(bond_mult_dep_A_slope,type="marginal")
```

Table S9B: Bonding variables as random slopes, Dataset B

Hypothesis 1a, Dataset B

Hypothesis 1a model converged for Dataset B; findings hold the same.

```
# Distancing
summary(bond_distancing_B_slope<- lme(comply_self_scale ~ bond_country + bond_gvmt  +
                                 age + gender + education + GDP,
                                 (~bond_country + bond_gvmt|country_now_Name), data=DatasetB_phase1, na.a
```

```
## Linear mixed-effects model fit by REML
##   Data: DatasetB_phase1
##        AIC      BIC   logLik
##   17567.74 17682.38 -8766.87
##
## Random effects:
##  Formula: ~bond_country + bond_gvmt | country_now_Name
##  Structure: General positive-definite, Log-Cholesky parametrization
##              StdDev     Corr
## (Intercept)   0.17154785 (Intr) bnd_c1
## bond_country1 0.06734002 -0.572
## bond_gvmt1    0.11720347 -0.549  0.990
## Residual      0.96878546
##
## Fixed effects:  comply_self_scale ~ bond_country + bond_gvmt + age + gender +      education + GDP
##                               Value  Std.Error   DF   t-value p-value
## (Intercept)            -0.25816527 0.06302021 6164 -4.096547  0.0000
## bond_country1          -0.00977855 0.03803841 6164 -0.257070  0.7971
## bond_gvmt1              0.10769113 0.06108724 6164  1.762907  0.0780
## age                     0.00109411 0.00096461 6164  1.134257  0.2567
## genderwoman             0.06581603 0.02753878 6164  2.389940  0.0169
## gendernb                0.06508798 0.13103302 6164  0.496730  0.6194
## gendernone              0.26828308 0.14815767 6164  1.810794  0.0702
## educationundergraduate  0.05368515 0.03646863 6164  1.472091  0.1410
## educationpostgraduate   0.11043455 0.03916366 6164  2.819822  0.0048
## GDP                     0.00000388 0.00000127  105  3.053803  0.0029
##  Correlation:
##                        (Intr) bnd_c1 bnd_g1 age    gndrwm gndrnb gndrnn edctnn
## bond_country1          -0.147
## bond_gvmt1             -0.141 -0.070
## age                    -0.373 -0.161  0.002
## genderwoman            -0.259 -0.030  0.033  0.083
## gendernb               -0.076  0.008  0.017  0.054  0.152
## gendernone             -0.067  0.013 -0.010  0.029  0.125  0.029
## educationundergraduate -0.351  0.005  0.005 -0.181 -0.028 -0.005  0.005
## educationpostgraduate  -0.307  0.045  0.010 -0.254 -0.045 -0.003  0.007  0.722
## GDP                    -0.472  0.051 -0.023 -0.099 -0.083 -0.024 -0.004  0.019
```

```
##                          edctnp
## bond_country1
## bond_gvmt1
## age
## genderwoman
## gendernb
## gendernone
## educationundergraduate
## educationpostgraduate
## GDP                      -0.006
##
## Standardized Within-Group Residuals:
##        Min         Q1        Med         Q3        Max
## -3.1484721 -0.6825605 -0.3034880  0.7668776  2.1299374
##
## Number of Observations: 6279
## Number of Groups: 107
```

```
anova.lme(bond_distancing_B_slope,type="marginal")
```

```
##               numDF denDF   F-value p-value
## (Intercept)       1  6164 16.781696  <.0001
## bond_country      1  6164  0.066085  0.7971
## bond_gvmt         1  6164  3.107841  0.0780
## age               1  6164  1.286538  0.2567
## gender            3  6164  2.683127  0.0451
## education         2  6164  4.307795  0.0135
## GDP               1   105  9.325713  0.0029
```

Hypothesis 1b, Dataset B

Hypothesis 1b model did not converge for Dataset B.

```
# Distancing
summary(bond_mult_distancing_B_slope<- lme(comply_self_scale ~ bond_mult  +
                                age + gender + education + GDP,
                                (~bond_mult|country_now_Name), data=DatasetB_phase1, na.action=na.exclu
anova.lme(bond_mult_distancing_B_slope,type="marginal")
```

Hypothesis 2a, Dataset B

Hypothesis 2a model converged for Dataset B; findings hold the same.

```
# Wellbeing
summary(bond_wellbeing_B_slope<- lme(wellbeing_scale ~ bond_country + bond_gvmt  +
                                age + gender + education + GDP,
                                (~bond_country + bond_gvmt|country_now_Name), data=DatasetB_phase1, na.a
```

```
## Linear mixed-effects model fit by REML
##   Data: DatasetB_phase1
##        AIC      BIC    logLik
```

```
##   16983.28 17097.92 -8474.641
##
## Random effects:
##  Formula: ~bond_country + bond_gvmt | country_now_Name
##  Structure: General positive-definite, Log-Cholesky parametrization
##               StdDev     Corr
## (Intercept)   0.19820210 (Intr) bnd_c1
## bond_country1 0.17594407  0.089
## bond_gvmt1    0.08305346 -0.662 -0.792
## Residual      0.92298683
##
## Fixed effects:  wellbeing_scale ~ bond_country + bond_gvmt + age + gender + education +      GDP
##                             Value  Std.Error   DF   t-value p-value
## (Intercept)            -0.5416513 0.06427268 6164 -8.427396  0.0000
## bond_country1           0.2665301 0.05357774 6164  4.974643  0.0000
## bond_gvmt1              0.2679385 0.05410733 6164  4.951982  0.0000
## age                     0.0142229 0.00092750 6164 15.334588  0.0000
## genderwoman            -0.1583189 0.02631608 6164 -6.016054  0.0000
## gendernb               -0.2657648 0.12492096 6164 -2.127464  0.0334
## gendernone             -0.3254586 0.14123285 6164 -2.304412  0.0212
## educationundergraduate  0.0667935 0.03478635 6164  1.920106  0.0549
## educationpostgraduate   0.1460074 0.03741188 6164  3.902702  0.0001
## GDP                    -0.0000023 0.00000137  105 -1.644520  0.1031
##  Correlation:
##                        (Intr) bnd_c1 bnd_g1 age    gndrwm gndrnb gndrnn edctnn
## bond_country1          -0.093
## bond_gvmt1             -0.084 -0.425
## age                    -0.359 -0.088 -0.016
## genderwoman            -0.245 -0.007  0.027  0.085
## gendernb               -0.072  0.007  0.017  0.055  0.152
## gendernone             -0.066  0.012 -0.014  0.030  0.126  0.029
## educationundergraduate -0.328  0.001  0.015 -0.182 -0.028 -0.005  0.005
## educationpostgraduate  -0.287  0.021  0.020 -0.256 -0.046 -0.004  0.007  0.722
## GDP                    -0.511  0.065 -0.038 -0.075 -0.071 -0.020  0.000  0.015
##                        edctnp
## bond_country1
## bond_gvmt1
## age
## genderwoman
## gendernb
## gendernone
## educationundergraduate
## educationpostgraduate
## GDP                    -0.007
##
## Standardized Within-Group Residuals:
##         Min          Q1         Med          Q3         Max
## -4.71744463 -0.67205749 -0.05586223  0.60108912  3.98599512
##
## Number of Observations: 6279
## Number of Groups: 107
```

```
anova.lme(bond_wellbeing_B_slope,type="marginal")
```

```
##                 numDF denDF   F-value p-value
## (Intercept)         1  6164  71.02100  <.0001
## bond_country        1  6164  24.74707  <.0001
## bond_gvmt           1  6164  24.52213  <.0001
## age                 1  6164 235.14960  <.0001
## gender              3  6164  13.36400  <.0001
## education           2  6164   8.45623  0.0002
## GDP                 1   105   2.70444  0.1031
```

Hypothesis 2b, Dataset B

Hypothesis 2b model did not converge for Dataset B.

```
# Wellbeing
summary(bond_mult_wellbeing_B_slope<- lme(wellbeing_scale ~ bond_mult  +
                                age + gender + education + GDP,
                                (~bond_mult|country_now_Name), data=DatasetB_phase1, na.action=na.exclu
anova.lme(bond_mult_wellbeing_B_slope,type="marginal")
```

Table S10A: Continuous bonding variables, Dataset A

Hypothesis 1a, Dataset A

```
# Distancing
summary(bond_cont_distancing_A<- lme(Pro_Distancing_scaled ~ Fusion_Family1 + Fusion_Friends1 +
                                  Fusion_Country1 + Fusion_Humanity1  +
                                  demo_age + demo_gender + demo_education + GDP,
                                (~1|Country_Name), data=DatasetA, na.action=na.exclude))
```

```
## Linear mixed-effects model fit by REML
##   Data: DatasetA
##       AIC      BIC   logLik
##   18371.5 18466.46 -9171.75
##
## Random effects:
##  Formula: ~1 | Country_Name
##         (Intercept)  Residual
## StdDev:   0.8279912 0.9693328
##
## Fixed effects:  Pro_Distancing_scaled ~ Fusion_Family1 + Fusion_Friends1 + Fusion_Country1 +      Fu
##                                  Value Std.Error   DF   t-value p-value
## (Intercept)                 -0.6878449 0.18159464 6452 -3.787804  0.0002
## Fusion_Family1               0.0277568 0.01161782 6452  2.389158  0.0169
## Fusion_Friends1             -0.0068022 0.01155765 6452 -0.588545  0.5562
## Fusion_Country1             -0.0087109 0.01237697 6452 -0.703797  0.4816
## Fusion_Humanity1             0.0364960 0.01115199 6452  3.272601  0.0011
## demo_age                     0.0009654 0.00098758 6452  0.977529  0.3283
## demo_genderFemale            0.1510950 0.02528488 6452  5.975707  0.0000
## demo_genderOther/non-binary  0.0472630 0.12910964 6452  0.366069  0.7143
## demo_educationundergraduate  0.1119046 0.03058515 6452  3.658789  0.0003
## demo_educationpostgraduate   0.0803527 0.03333574 6452  2.410406  0.0160
```

```
## demo_educationNA            -0.1396889 0.14526902 6452 -0.961588  0.3363
## GDP                         -0.0000006 0.00000403   69 -0.141191  0.8881
##  Correlation:
##                              (Intr) Fsn_Fm1 Fsn_Fr1 Fsn_C1 Fsn_H1 demo_g dm_gnF
## Fusion_Family1               -0.130
## Fusion_Friends1              -0.079 -0.243
## Fusion_Country1              -0.022 -0.164  -0.155
## Fusion_Humanity1             -0.102 -0.023  -0.108  -0.364
## demo_age                     -0.132 -0.132   0.024  -0.128 -0.006
## demo_genderFemale            -0.045 -0.062  -0.028   0.018 -0.047 -0.023
## demo_genderOther/non-binary -0.016  0.056  -0.024   0.009 -0.016  0.025  0.110
## demo_educationundergraduate -0.082  0.038  -0.015  -0.005 -0.035 -0.070 -0.016
## demo_educationpostgraduate  -0.060  0.006   0.007   0.013 -0.075 -0.156 -0.034
## demo_educationNA            -0.032 -0.001  -0.007   0.004 -0.020 -0.023 -0.014
## GDP                         -0.680  0.011  -0.006  -0.005  0.017  0.001 -0.003
##                              dm_O/- dm_dctnn dm_dctnp dm_dNA
## Fusion_Family1
## Fusion_Friends1
## Fusion_Country1
## Fusion_Humanity1
## demo_age
## demo_genderFemale
## demo_genderOther/non-binary
## demo_educationundergraduate  0.019
## demo_educationpostgraduate   0.010  0.581
## demo_educationNA             0.006  0.121    0.119
## GDP                         -0.031 -0.010   -0.010    0.012
##
## Standardized Within-Group Residuals:
##        Min         Q1        Med         Q3        Max
## -6.7893401 -0.2058475  0.3466587  0.5602388  2.4595283
##
## Number of Observations: 6533
## Number of Groups: 71
```

```
anova.lme(bond_cont_distancing_A,type="marginal") # Humanity sig.
```

```
##                  numDF denDF   F-value p-value
## (Intercept)          1  6452 14.347461  0.0002
## Fusion_Family1       1  6452  5.708077  0.0169
## Fusion_Friends1      1  6452  0.346385  0.5562
## Fusion_Country1      1  6452  0.495330  0.4816
## Fusion_Humanity1     1  6452 10.709919  0.0011
## demo_age             1  6452  0.955562  0.3283
## demo_gender          2  6452 17.897402  <.0001
## demo_education       3  6452  5.193719  0.0014
## GDP                  1    69  0.019935  0.8881
```

```
# Hygiene
summary(bond_cont_hygiene_A<- lme(Pro_Hygiene_scaled ~ Fusion_Family1 + Fusion_Friends1 +
                                  Fusion_Country1 + Fusion_Humanity1  +
                                  demo_age + demo_gender + demo_education + GDP,
                                (~1|Country_Name), data=DatasetA, na.action=na.exclude))
```

```
## Linear mixed-effects model fit by REML
##   Data: DatasetA
##        AIC   BIC    logLik
##   17907.07 18002 -8939.535
##
## Random effects:
##  Formula: ~1 | Country_Name
##         (Intercept)  Residual
## StdDev:   0.2169677 0.9444546
##
## Fixed effects:  Pro_Hygiene_scaled ~ Fusion_Family1 + Fusion_Friends1 + Fusion_Country1 +      Fusio
##                                  Value  Std.Error   DF   t-value p-value
## (Intercept)                 -0.6954232 0.10377579 6436 -6.701208  0.0000
## Fusion_Family1               0.1201054 0.01130116 6436 10.627706  0.0000
## Fusion_Friends1              0.0118428 0.01126187 6436  1.051585  0.2930
## Fusion_Country1             -0.0215131 0.01201024 6436 -1.791230  0.0733
## Fusion_Humanity1             0.0205670 0.01084310 6436  1.896782  0.0579
## demo_age                     0.0054495 0.00095925 6436  5.681059  0.0000
## demo_genderFemale            0.2616373 0.02457579 6436 10.646140  0.0000
## demo_genderOther/non-binary  0.0100219 0.12511697 6436  0.080100  0.9362
## demo_educationundergraduate -0.0217980 0.02978337 6436 -0.731886  0.4643
## demo_educationpostgraduate  -0.1169884 0.03241546 6436 -3.609029  0.0003
## demo_educationNA             0.3648254 0.14033152 6436  2.599740  0.0094
## GDP                         -0.0000047 0.00000197   70 -2.357679  0.0212
##  Correlation:
##                             (Intr) Fsn_Fm1 Fsn_Fr1 Fsn_C1 Fsn_H1 demo_g dm_gnF
## Fusion_Family1              -0.222
## Fusion_Friends1             -0.136 -0.244
## Fusion_Country1             -0.040 -0.164  -0.156
## Fusion_Humanity1            -0.173 -0.023  -0.107  -0.363
## demo_age                    -0.227 -0.132   0.024  -0.130 -0.006
## demo_genderFemale           -0.070 -0.060  -0.029   0.017 -0.046 -0.023
## demo_genderOther/non-binary -0.036  0.057  -0.022   0.010 -0.016  0.025  0.110
## demo_educationundergraduate -0.145  0.038  -0.014  -0.007 -0.034 -0.071 -0.019
## demo_educationpostgraduate  -0.100  0.006   0.008   0.013 -0.074 -0.156 -0.037
## demo_educationNA            -0.041 -0.003  -0.006   0.005 -0.018 -0.022 -0.015
## GDP                         -0.690  0.026  -0.010  -0.013  0.037 -0.003 -0.014
##                             dm_O/- dm_dctnn dm_dctnp dm_dNA
## Fusion_Family1
## Fusion_Friends1
## Fusion_Country1
## Fusion_Humanity1
## demo_age
## demo_genderFemale
## demo_genderOther/non-binary
## demo_educationundergraduate  0.018
## demo_educationpostgraduate   0.008  0.583
## demo_educationNA             0.006  0.122    0.120
## GDP                         -0.033 -0.013   -0.014    0.016
##
## Standardized Within-Group Residuals:
##         Min          Q1         Med          Q3         Max
## -3.39789045 -0.63816715  0.05822122  0.69966888  2.44182131
##
```

```
## Number of Observations: 6518
## Number of Groups: 72
```

```
anova.lme(bond_cont_hygiene_A,type="marginal")
```

```
##                    numDF denDF   F-value p-value
## (Intercept)            1  6436  44.90619  <.0001
## Fusion_Family1         1  6436 112.94814  <.0001
## Fusion_Friends1        1  6436   1.10583  0.2930
## Fusion_Country1        1  6436   3.20850  0.0733
## Fusion_Humanity1       1  6436   3.59778  0.0579
## demo_age               1  6436  32.27443  <.0001
## demo_gender            2  6436  57.26690  <.0001
## demo_education         3  6436   8.19181  <.0001
## GDP                    1    70   5.55865  0.0212
```

```
# Masking
summary(bond_cont_masking_A<- lme(Pro_MaskWearing_scaled ~ Fusion_Family1 + Fusion_Friends1 +
                                   Fusion_Country1 + Fusion_Humanity1  +
                                   demo_age + demo_gender + demo_education + GDP,
                                 (~1|Country_Name), data=DatasetA, na.action=na.exclude))
```

```
## Linear mixed-effects model fit by REML
##   Data: DatasetA
##        AIC      BIC    logLik
##   17770.61 17865.55 -8871.303
##
## Random effects:
##  Formula: ~1 | Country_Name
##         (Intercept)  Residual
## StdDev:   0.4191847 0.9310013
##
## Fixed effects:  Pro_MaskWearing_scaled ~ Fusion_Family1 + Fusion_Friends1 + Fusion_Country1 +      Fu
##                                  Value  Std.Error   DF   t-value p-value
## (Intercept)                  0.0545995 0.12834183 6443  0.425423  0.6705
## Fusion_Family1               0.0405305 0.01114780 6443  3.635739  0.0003
## Fusion_Friends1             -0.0176734 0.01108710 6443 -1.594052  0.1110
## Fusion_Country1             -0.0378597 0.01187495 6443 -3.188202  0.0014
## Fusion_Humanity1             0.0224757 0.01069919 6443  2.100690  0.0357
## demo_age                     0.0030800 0.00094860 6443  3.246936  0.0012
## demo_genderFemale            0.0427719 0.02426193 6443  1.762921  0.0780
## demo_genderOther/non-binary  0.0217387 0.12468860 6443  0.174344  0.8616
## demo_educationundergraduate  0.0161263 0.02937495 6443  0.548982  0.5830
## demo_educationpostgraduate  -0.0649019 0.03202174 6443 -2.026808  0.0427
## demo_educationNA             0.3545534 0.13883510 6443  2.553773  0.0107
## GDP                         -0.0000054 0.00000264   69 -2.042300  0.0449
##  Correlation:
##                             (Intr) Fsn_Fm1 Fsn_Fr1 Fsn_C1 Fsn_H1 demo_g dm_gnF
## Fusion_Family1              -0.179
## Fusion_Friends1             -0.106 -0.243
## Fusion_Country1             -0.031 -0.165  -0.155
## Fusion_Humanity1            -0.138 -0.022  -0.108  -0.364
## demo_age                    -0.179 -0.131   0.025  -0.130 -0.006
```

```
## demo_genderFemale           -0.062 -0.060  -0.027   0.017 -0.046 -0.024
## demo_genderOther/non-binary -0.025  0.054  -0.021   0.009 -0.017  0.025  0.109
## demo_educationundergraduate -0.115  0.039  -0.014  -0.006 -0.036 -0.071 -0.016
## demo_educationpostgraduate  -0.084  0.007   0.007   0.013 -0.075 -0.157 -0.036
## demo_educationNA            -0.040 -0.002  -0.006   0.004 -0.019 -0.022 -0.014
## GDP                         -0.692  0.020  -0.010  -0.008  0.025 -0.002 -0.005
##                             dm_O/- dm_dctnn dm_dctnp dm_dNA
## Fusion_Family1
## Fusion_Friends1
## Fusion_Country1
## Fusion_Humanity1
## demo_age
## demo_genderFemale
## demo_genderOther/non-binary
## demo_educationundergraduate  0.021
## demo_educationpostgraduate   0.009  0.582
## demo_educationNA             0.006  0.122    0.120
## GDP                         -0.035 -0.011   -0.011    0.016
##
## Standardized Within-Group Residuals:
##        Min         Q1        Med         Q3        Max
## -1.8233113 -0.6780140 -0.2929958  0.5703616  3.4131762
##
## Number of Observations: 6524
## Number of Groups: 71
```

```
anova.lme(bond_cont_masking_A,type="marginal") # Country, Humanity sig.
```

```
##                  numDF denDF   F-value p-value
## (Intercept)          1  6443  0.180985  0.6705
## Fusion_Family1       1  6443 13.218598  0.0003
## Fusion_Friends1      1  6443  2.541001  0.1110
## Fusion_Country1      1  6443 10.164630  0.0014
## Fusion_Humanity1     1  6443  4.412898  0.0357
## demo_age             1  6443 10.542593  0.0012
## demo_gender          2  6443  1.554102  0.2115
## demo_education       3  6443  5.278790  0.0012
## GDP                  1    69  4.170989  0.0449
```

Hypothesis 2a, Dataset A

```
# Anxiety
summary(bond_cont_anx_A <- lme(anx_sum1_scaled ~
                                 Fusion_Family1 + Fusion_Friends1 + Fusion_Country1 + Fusion_Humanity1 +
                                 demo_age + demo_gender + demo_education + GDP, (~1|Country_Name), data=
```

```
## Linear mixed-effects model fit by REML
##   Data: DatasetA
##        AIC      BIC    logLik
##   17567.81 17662.79 -8769.905
##
```

```
## Random effects:
##  Formula: ~1 | Country_Name
##         (Intercept)  Residual
## StdDev:   0.2278157 0.9148487
##
## Fixed effects:  anx_sum1_scaled ~ Fusion_Family1 + Fusion_Friends1 + Fusion_Country1 +      Fusion_Hu
##                                  Value  Std.Error   DF   t-value p-value
## (Intercept)                    0.4887270 0.10284026 6463  4.752293  0.0000
## Fusion_Family1               -0.0287766 0.01093175 6463 -2.632386  0.0085
## Fusion_Friends1              -0.0628841 0.01087531 6463 -5.782285  0.0000
## Fusion_Country1              -0.1065206 0.01162235 6463 -9.165152  0.0000
## Fusion_Humanity1             -0.0067567 0.01049099 6463 -0.644053  0.5196
## demo_age                     -0.0074303 0.00092663 6463 -8.018668  0.0000
## demo_genderFemale             0.4693457 0.02376655 6463 19.748163  0.0000
## demo_genderOther/non-binary   0.5091631 0.12121982 6463  4.200329  0.0000
## demo_educationundergraduate -0.0185485 0.02877179 6463 -0.644676  0.5192
## demo_educationpostgraduate  -0.0158137 0.03134068 6463 -0.504576  0.6139
## demo_educationNA              0.1180975 0.13595553 6463  0.868648  0.3851
## GDP                          -0.0000014 0.00000198   70 -0.699394  0.4866
##  Correlation:
##                             (Intr) Fsn_Fm1 Fsn_Fr1 Fsn_C1 Fsn_H1 demo_g dm_gnF
## Fusion_Family1              -0.217
## Fusion_Friends1             -0.131 -0.243
## Fusion_Country1             -0.039 -0.165  -0.155
## Fusion_Humanity1            -0.168 -0.023  -0.109  -0.363
## demo_age                    -0.221 -0.132   0.024  -0.130 -0.008
## demo_genderFemale           -0.070 -0.061  -0.026   0.018 -0.046 -0.024
## demo_genderOther/non-binary -0.034  0.057  -0.022   0.010 -0.016  0.025  0.109
## demo_educationundergraduate -0.141  0.038  -0.014  -0.007 -0.036 -0.070 -0.017
## demo_educationpostgraduate  -0.098  0.008   0.007   0.013 -0.075 -0.155 -0.036
## demo_educationNA            -0.041 -0.002  -0.006   0.004 -0.018 -0.021 -0.014
## GDP                         -0.692  0.025  -0.011  -0.013  0.036 -0.002 -0.013
##                             dm_O/- dm_dctnn dm_dctnp dm_dNA
## Fusion_Family1
## Fusion_Friends1
## Fusion_Country1
## Fusion_Humanity1
## demo_age
## demo_genderFemale
## demo_genderOther/non-binary
## demo_educationundergraduate  0.018
## demo_educationpostgraduate   0.008  0.582
## demo_educationNA             0.006  0.122    0.119
## GDP                         -0.033 -0.013   -0.014    0.016
##
## Standardized Within-Group Residuals:
##         Min          Q1         Med          Q3         Max
## -2.63422795 -0.74328428 -0.07049267  0.64807347  3.55552943
##
## Number of Observations: 6545
## Number of Groups: 72
```

```
anova.lme(bond_cont_anx_A,type="marginal") # Humanity n.s.
```

```
##                   numDF denDF   F-value p-value
## (Intercept)           1  6463  22.58429  <.0001
## Fusion_Family1        1  6463   6.92946  0.0085
## Fusion_Friends1       1  6463  33.43482  <.0001
## Fusion_Country1       1  6463  84.00001  <.0001
## Fusion_Humanity1      1  6463   0.41480  0.5196
## demo_age              1  6463  64.29903  <.0001
## demo_gender           2  6463 197.10627  <.0001
## demo_education        3  6463   0.45765  0.7119
## GDP                   1    70   0.48915  0.4866
```

```
# Depression
summary(bond_cont_dep_A <- lme(dep_sum1_scaled ~
                                 Fusion_Family1 + Fusion_Friends1 + Fusion_Country1 + Fusion_Humanity1
                                 demo_age + demo_gender + demo_education + GDP, (~1|Country_Name), data
```

```
## Linear mixed-effects model fit by REML
##   Data: DatasetA
##        AIC      BIC    logLik
##   17834.97 17929.96 -8903.487
##
## Random effects:
##  Formula: ~1 | Country_Name
##         (Intercept)  Residual
## StdDev:   0.1938358 0.9338697
##
## Fixed effects:  dep_sum1_scaled ~ Fusion_Family1 + Fusion_Friends1 + Fusion_Country1 +      Fusion_Hu
##                                  Value  Std.Error   DF   t-value p-value
## (Intercept)                  1.1101056 0.09954282 6465 11.152040  0.0000
## Fusion_Family1              -0.0835763 0.01115810 6465 -7.490190  0.0000
## Fusion_Friends1             -0.1081366 0.01109635 6465 -9.745239  0.0000
## Fusion_Country1             -0.0914521 0.01185072 6465 -7.717008  0.0000
## Fusion_Humanity1            -0.0476196 0.01070673 6465 -4.447633  0.0000
## demo_age                    -0.0039762 0.00094510 6465 -4.207112  0.0000
## demo_genderFemale            0.2376917 0.02424472 6465  9.803855  0.0000
## demo_genderOther/non-binary  0.4745371 0.12367053 6465  3.837108  0.0001
## demo_educationundergraduate -0.1013241 0.02935496 6465 -3.451686  0.0006
## demo_educationpostgraduate  -0.1089851 0.03196381 6465 -3.409639  0.0007
## demo_educationNA            -0.0237940 0.13868596 6465 -0.171568  0.8638
## GDP                         -0.0000036 0.00000187   70 -1.900909  0.0614
##  Correlation:
##                             (Intr) Fsn_Fm1 Fsn_Fr1 Fsn_C1 Fsn_H1 demo_g dm_gnF
## Fusion_Family1              -0.228
## Fusion_Friends1             -0.139 -0.243
## Fusion_Country1             -0.042 -0.166  -0.155
## Fusion_Humanity1            -0.177 -0.022  -0.109  -0.363
## demo_age                    -0.233 -0.133   0.024  -0.130 -0.008
## demo_genderFemale           -0.072 -0.062  -0.026   0.018 -0.047 -0.025
## demo_genderOther/non-binary -0.037  0.057  -0.021   0.010 -0.016  0.025  0.109
## demo_educationundergraduate -0.149  0.038  -0.014  -0.007 -0.036 -0.070 -0.017
## demo_educationpostgraduate  -0.103  0.008   0.007   0.013 -0.075 -0.155 -0.035
## demo_educationNA            -0.040 -0.002  -0.006   0.005 -0.018 -0.021 -0.014
## GDP                         -0.688  0.027  -0.011  -0.014  0.039 -0.004 -0.016
##                             dm_O/- dm_dctnn dm_dctnp dm_dNA
```

```
## Fusion_Family1
## Fusion_Friends1
## Fusion_Country1
## Fusion_Humanity1
## demo_age
## demo_genderFemale
## demo_genderOther/non-binary
## demo_educationundergraduate  0.018
## demo_educationpostgraduate   0.008  0.582
## demo_educationNA             0.006  0.122    0.119
## GDP                         -0.032 -0.013   -0.014    0.016
##
## Standardized Within-Group Residuals:
##         Min          Q1         Med          Q3         Max
## -2.82434138 -0.74696774 -0.07813648  0.65961875  4.26392731
##
## Number of Observations: 6547
## Number of Groups: 72
```

```
anova.lme(bond_cont_dep_A,type="marginal") # Humanity n.s.
```

```
##                   numDF denDF   F-value p-value
## (Intercept)           1  6465 124.36800  <.0001
## Fusion_Family1        1  6465  56.10295  <.0001
## Fusion_Friends1       1  6465  94.96968  <.0001
## Fusion_Country1       1  6465  59.55221  <.0001
## Fusion_Humanity1      1  6465  19.78144  <.0001
## demo_age              1  6465  17.69979  <.0001
## demo_gender           2  6465  51.93394  <.0001
## demo_education        3  6465   5.00150  0.0018
## GDP                   1    70   3.61346  0.0614
```

Table S10B: Continuous bonding variables, Dataset B

Hypothesis 1a, Dataset B

```
# Distancing
summary(bond_cont_distancing_B <- lme(comply_self_scale ~ ios_country + ios_gvmt  +
                                   age + gender + education + GDP, (~1|country_now_Name),
                                     data=DatasetB_phase1, na.action=na.exclude, method="REML"))
```

```
## Linear mixed-effects model fit by REML
##   Data: DatasetB_phase1
##        AIC      BIC    logLik
##   17562.62 17643.54 -8769.311
##
## Random effects:
##  Formula: ~1 | country_now_Name
##         (Intercept)  Residual
## StdDev:   0.1594725 0.9693083
##
```

```
## Fixed effects:  comply_self_scale ~ ios_country + ios_gvmt + age + gender + education +      GDP
##                               Value  Std.Error   DF   t-value p-value
## (Intercept)             -0.20425016 0.06424373 6164 -3.179301  0.0015
## ios_country             -0.03247853 0.01144056 6164 -2.838893  0.0045
## ios_gvmt                 0.01228851 0.01188690 6164  1.033786  0.3013
## age                      0.00169089 0.00096499 6164  1.752242  0.0798
## genderwoman              0.06594794 0.02754539 6164  2.394156  0.0167
## gendernb                 0.05092173 0.13118485 6164  0.388168  0.6979
## gendernone               0.25519866 0.14816390 6164  1.722408  0.0850
## educationundergraduate   0.05245285 0.03646620 6164  1.438396  0.1504
## educationpostgraduate    0.10387119 0.03912682 6164  2.654731  0.0080
## GDP                      0.00000371 0.00000129  105  2.884779  0.0048
##  Correlation: 
##                         (Intr) is_cnt is_gvm age    gndrwm gndrnb gndrnn edctnn
## ios_country             -0.217                                                 
## ios_gvmt                -0.022 -0.481                                          
## age                     -0.322 -0.179 -0.018                                   
## genderwoman             -0.248 -0.039  0.038  0.084                            
## gendernb                -0.082  0.009  0.038  0.051  0.153                     
## gendernone              -0.072  0.010  0.013  0.028  0.125  0.030              
## educationundergraduate -0.349  0.009 -0.001 -0.179 -0.029 -0.005  0.004       
## educationpostgraduate   -0.309  0.034  0.003 -0.249 -0.046 -0.003  0.007  0.722
## GDP                     -0.473  0.039 -0.071 -0.077 -0.083 -0.028 -0.006  0.020
##                         edctnp
## ios_country                   
## ios_gvmt                      
## age                           
## genderwoman                   
## gendernb                      
## gendernone                    
## educationundergraduate       
## educationpostgraduate         
## GDP                     -0.011
## 
## Standardized Within-Group Residuals:
##        Min         Q1        Med         Q3        Max 
## -3.1896424 -0.6805329 -0.3074357  0.7670324  2.1473511 
## 
## Number of Observations: 6279
## Number of Groups: 107
```

```
anova.lme(bond_cont_distancing_B,type="marginal") # Country sig.
```

```
##              numDF denDF   F-value p-value
## (Intercept)      1  6164 10.107954  0.0015
## ios_country      1  6164  8.059311  0.0045
## ios_gvmt         1  6164  1.068713  0.3013
## age              1  6164  3.070353  0.0798
## gender           3  6164  2.595633  0.0507
## education        2  6164  3.764141  0.0232
## GDP              1   105  8.321952  0.0048
```

Hypothesis 2a, Dataset B

```r
# Wellbeing
summary(bond_cont_wellbeing_B <- lme(wellbeing_scale ~ ios_country + ios_gvmt  +
                                   age + gender + education + GDP, (~1|country_now_Name),
                                     data=DatasetB_phase1, na.action=na.exclude, method="REML"))
```

```
## Linear mixed-effects model fit by REML
##   Data: DatasetB_phase1
##        AIC      BIC    logLik
##   16867.16 16948.08 -8421.579
##
## Random effects:
##  Formula: ~1 | country_now_Name
##         (Intercept)  Residual
## StdDev:   0.1946763 0.9159785
##
## Fixed effects:  wellbeing_scale ~ ios_country + ios_gvmt + age + gender + education +      GDP
##                             Value  Std.Error   DF    t-value p-value
## (Intercept)            -0.7480738 0.06483206 6164 -11.538639  0.0000
## ios_country             0.1183987 0.01082349 6164  10.939050  0.0000
## ios_gvmt                0.0615380 0.01127983 6164   5.455579  0.0000
## age                     0.0134192 0.00091379 6164  14.685262  0.0000
## genderwoman            -0.1614105 0.02606626 6164  -6.192316  0.0000
## gendernb               -0.2166882 0.12403536 6164  -1.746987  0.0807
## gendernone             -0.2913828 0.14007701 6164  -2.080162  0.0376
## educationundergraduate  0.0623089 0.03448512 6164   1.806835  0.0708
## educationpostgraduate   0.1414942 0.03704301 6164   3.819727  0.0001
## GDP                    -0.0000033 0.00000137  105  -2.430869  0.0168
##  Correlation:
##                        (Intr) is_cnt is_gvm age    gndrwm gndrnb gndrnn edctnn
## ios_country            -0.202
## ios_gvmt               -0.022 -0.479
## age                    -0.303 -0.179 -0.020
## genderwoman            -0.234 -0.039  0.038  0.085
## gendernb               -0.078  0.008  0.039  0.051  0.153
## gendernone             -0.068  0.010  0.013  0.028  0.125  0.030
## educationundergraduate -0.328  0.009 -0.001 -0.179 -0.029 -0.005  0.004
## educationpostgraduate  -0.294  0.034  0.003 -0.248 -0.046 -0.003  0.007  0.722
## GDP                    -0.499  0.036 -0.066 -0.066 -0.072 -0.023 -0.003  0.016
##                        edctnp
## ios_country
## ios_gvmt
## age
## genderwoman
## gendernb
## gendernone
## educationundergraduate
## educationpostgraduate
## GDP                    -0.009
##
## Standardized Within-Group Residuals:
##        Min         Q1        Med         Q3        Max
```

```
## -4.3588813 -0.6654129 -0.0705161  0.5913088  4.2567581
##
## Number of Observations: 6279
## Number of Groups: 107
```

```r
anova.lme(bond_cont_wellbeing_B,type="marginal")
```

```
##             numDF denDF   F-value p-value
## (Intercept)     1  6164 133.14019  <.0001
## ios_country     1  6164 119.66282  <.0001
## ios_gvmt        1  6164  29.76334  <.0001
## age             1  6164 215.65691  <.0001
## gender          3  6164  13.56772  <.0001
## education       2  6164   8.24066  0.0003
## GDP             1   105   5.90912  0.0168
```

Table S11A: Main analyses only within N>100 countries, Dataset A

Hypothesis 1a, Dataset A

```r
# Distancing
summary(bond_distancing_A100 <- lmer(Pro_Distancing_scaled ~ bond_family +
                                       bond_friend + bond_country + bond_humanity  +
                                      demo_age + demo_gender + demo_education + GDP + Country_Name +
                                    (1|Country_Name), data=DatasetA_N100, na.action=na.exclude))
```

```
## Linear mixed model fit by REML. t-tests use Satterthwaite's method [
## lmerModLmerTest]
## Formula: Pro_Distancing_scaled ~ bond_family + bond_friend + bond_country +
##     bond_humanity + demo_age + demo_gender + demo_education +
##     GDP + Country_Name + (1 | Country_Name)
##    Data: DatasetA_N100
##
## REML criterion at convergence: 16369.6
##
## Scaled residuals:
##     Min      1Q  Median      3Q     Max
## -6.7526 -0.2068  0.3463  0.5733  1.1812
##
## Random effects:
##  Groups       Name        Variance Std.Dev.
##  Country_Name (Intercept) 0.0000   0.0000
##  Residual                 0.9364   0.9677
## Number of obs: 5871, groups:  Country_Name, 11
##
## Fixed effects:
##                             Estimate Std. Error         df t value Pr(>|t|)
## (Intercept)               -1.445e+00  2.755e-01  5.850e+03  -5.245 1.62e-07
## bond_family1               9.370e-02  3.062e-02  5.850e+03   3.061 0.002220
## bond_friend1              -1.960e-02  4.545e-02  5.850e+03  -0.431 0.666283
## bond_country1              3.276e-02  4.522e-02  5.850e+03   0.724 0.468853
```

```
## bond_humanity1                3.252e-02  3.413e-02  5.850e+03   0.953 0.340683
## demo_age                      5.350e-04  1.037e-03  5.850e+03   0.516 0.605931
## demo_genderFemale             1.562e-01  2.659e-02  5.850e+03   5.874 4.48e-09
## demo_genderOther/non-binary   1.141e-01  1.387e-01  5.850e+03   0.823 0.410584
## demo_educationundergraduate   1.378e-01  3.186e-02  5.850e+03   4.325 1.55e-05
## demo_educationpostgraduate    1.026e-01  3.494e-02  5.850e+03   2.938 0.003321
## demo_educationNA             -8.530e-02  1.556e-01  5.850e+03  -0.548 0.583687
## GDP                           2.204e-05  4.403e-06  5.850e+03   5.005 5.76e-07
## Country_NameBrazil            1.123e+00  2.392e-01  5.850e+03   4.696 2.71e-06
## Country_NameCroatia           8.355e-01  2.180e-01  5.850e+03   3.832 0.000128
## Country_NameFinland          -2.373e-01  9.270e-02  5.850e+03  -2.560 0.010495
## Country_NameFrance            6.085e-01  1.211e-01  5.850e+03   5.024 5.20e-07
## Country_NameGermany          -4.601e-02  9.100e-02  5.850e+03  -0.506 0.613125
## Country_NameItaly             2.667e-01  1.309e-01  5.850e+03   2.038 0.041630
## Country_NameNew Zealand       4.621e-01  1.164e-01  5.850e+03   3.970 7.26e-05
## Country_NamePortugal          6.751e-01  1.758e-01  5.850e+03   3.841 0.000124
## Country_NameUK                3.544e-01  9.401e-02  5.850e+03   3.770 0.000165
##
## (Intercept)                  ***
## bond_family1                 **
## bond_friend1
## bond_country1
## bond_humanity1
## demo_age
## demo_genderFemale            ***
## demo_genderOther/non-binary
## demo_educationundergraduate ***
## demo_educationpostgraduate  **
## demo_educationNA
## GDP                          ***
## Country_NameBrazil           ***
## Country_NameCroatia          ***
## Country_NameFinland          *
## Country_NameFrance           ***
## Country_NameGermany
## Country_NameItaly            *
## Country_NameNew Zealand      ***
## Country_NamePortugal         ***
## Country_NameUK               ***
## ---
## Signif. codes:  0 '***' 0.001 '**' 0.01 '*' 0.05 '.' 0.1 ' ' 1
## fit warnings:
## fixed-effect model matrix is rank deficient so dropping 1 column / coefficient
## Some predictor variables are on very different scales: consider rescaling
## optimizer (nloptwrap) convergence code: 0 (OK)
## boundary (singular) fit: see ?isSingular
```

```
anova(bond_distancing_A100)
```

```
## Type III Analysis of Variance Table with Satterthwaite's method
##                 Sum Sq Mean Sq NumDF DenDF F value    Pr(>F)
## bond_family      8.771  8.7707     1  5850  9.3667 0.0022196 **
## bond_friend      0.174  0.1742     1  5850  0.1860 0.6662835
## bond_country     0.491  0.4914     1  5850  0.5247 0.4688533
```

```
## bond_humanity    0.850  0.8502     1  5850  0.9080 0.3406828
## demo_age         0.249  0.2492     1  5850  0.2662 0.6059310
## demo_gender     32.346 16.1730     2  5850 17.2720 3.318e-08 ***
## demo_education  18.926  6.3086     3  5850  6.7373 0.0001559 ***
## GDP             23.454 23.4542     1  5850 25.0480 5.756e-07 ***
## Country_Name   136.455 15.1617     9  5850 16.1920 < 2.2e-16 ***
## ---
## Signif. codes:  0 '***' 0.001 '**' 0.01 '*' 0.05 '.' 0.1 ' ' 1
```

```
# Hygiene
summary(bond_hygiene_A100 <- lmer(Pro_Hygiene_scaled ~ bond_family + bond_friend +
                                   bond_country + bond_humanity +
                                   demo_age + demo_gender + demo_education + GDP + Country_Name +
                                   (1|Country_Name), data=DatasetA_N100, na.action=na.exclude))
```

```
## Linear mixed model fit by REML. t-tests use Satterthwaite's method [
## lmerModLmerTest]
## Formula: Pro_Hygiene_scaled ~ bond_family + bond_friend + bond_country +
##     bond_humanity + demo_age + demo_gender + demo_education +
##     GDP + Country_Name + (1 | Country_Name)
##    Data: DatasetA_N100
##
## REML criterion at convergence: 15964.8
##
## Scaled residuals:
##     Min      1Q  Median      3Q     Max
## -3.5831 -0.6422  0.0675  0.7050  2.6146
##
## Random effects:
##  Groups       Name        Variance Std.Dev.
##  Country_Name (Intercept) 0.0000   0.000
##  Residual                 0.8799   0.938
## Number of obs: 5856, groups:  Country_Name, 11
##
## Fixed effects:
##                               Estimate Std. Error         df t value Pr(>|t|)
## (Intercept)                 -2.201e+00  2.671e-01  5.835e+03  -8.242  < 2e-16
## bond_family1                 3.179e-01  2.972e-02  5.835e+03  10.696  < 2e-16
## bond_friend1                 6.934e-02  4.417e-02  5.835e+03   1.570  0.11654
## bond_country1                1.432e-02  4.389e-02  5.835e+03   0.326  0.74419
## bond_humanity1               1.828e-02  3.309e-02  5.835e+03   0.552  0.58070
## demo_age                     5.496e-03  1.007e-03  5.835e+03   5.458 5.02e-08
## demo_genderFemale            2.668e-01  2.581e-02  5.835e+03  10.337  < 2e-16
## demo_genderOther/non-binary -9.682e-02  1.344e-01  5.835e+03  -0.720  0.47141
## demo_educationundergraduate  1.996e-03  3.095e-02  5.835e+03   0.064  0.94859
## demo_educationpostgraduate  -8.858e-02  3.393e-02  5.835e+03  -2.611  0.00906
## demo_educationNA             3.941e-01  1.509e-01  5.835e+03   2.612  0.00903
## GDP                          3.284e-05  4.270e-06  5.835e+03   7.691 1.71e-14
## Country_NameBrazil           1.782e+00  2.319e-01  5.835e+03   7.684 1.80e-14
## Country_NameCroatia          1.476e+00  2.114e-01  5.835e+03   6.984 3.19e-12
## Country_NameFinland         -2.617e-01  8.986e-02  5.835e+03  -2.912  0.00360
## Country_NameFrance           1.552e-01  1.177e-01  5.835e+03   1.319  0.18725
## Country_NameGermany         -7.480e-02  8.821e-02  5.835e+03  -0.848  0.39652
## Country_NameItaly            2.878e-01  1.270e-01  5.835e+03   2.267  0.02342
```

```
## Country_NameNew Zealand      2.375e-01  1.128e-01  5.835e+03   2.105  0.03536
## Country_NamePortugal         1.102e+00  1.704e-01  5.835e+03   6.468 1.07e-10
## Country_NameUK               4.089e-01  9.113e-02  5.835e+03   4.487 7.38e-06
##
## (Intercept)                 ***
## bond_family1                ***
## bond_friend1
## bond_country1
## bond_humanity1
## demo_age                    ***
## demo_genderFemale           ***
## demo_genderOther/non-binary
## demo_educationundergraduate
## demo_educationpostgraduate  **
## demo_educationNA            **
## GDP                         ***
## Country_NameBrazil          ***
## Country_NameCroatia         ***
## Country_NameFinland         **
## Country_NameFrance
## Country_NameGermany
## Country_NameItaly           *
## Country_NameNew Zealand     *
## Country_NamePortugal        ***
## Country_NameUK              ***
## ---
## Signif. codes:  0 '***' 0.001 '**' 0.01 '*' 0.05 '.' 0.1 ' ' 1
## fit warnings:
## fixed-effect model matrix is rank deficient so dropping 1 column / coefficient
## Some predictor variables are on very different scales: consider rescaling
## optimizer (nloptwrap) convergence code: 0 (OK)
## boundary (singular) fit: see ?isSingular
```

```r
anova(bond_hygiene_A100)
```

```
## Type III Analysis of Variance Table with Satterthwaite's method
##                Sum Sq Mean Sq NumDF DenDF  F value    Pr(>F)
## bond_family    100.66 100.662     1  5835 114.4052 < 2.2e-16 ***
## bond_friend      2.17   2.168     1  5835   2.4640 0.1165371
## bond_country     0.09   0.094     1  5835   0.1065 0.7441889
## bond_humanity    0.27   0.268     1  5835   0.3051 0.5806953
## demo_age        26.21  26.210     1  5835  29.7880 5.018e-08 ***
## demo_gender     97.05  48.523     2  5835  55.1474 < 2.2e-16 ***
## demo_education  16.06   5.352     3  5835   6.0829 0.0003959 ***
## GDP             52.04  52.039     1  5835  59.1440 1.709e-14 ***
## Country_Name   400.07  44.452     9  5835  50.5204 < 2.2e-16 ***
## ---
## Signif. codes:  0 '***' 0.001 '**' 0.01 '*' 0.05 '.' 0.1 ' ' 1
```

```r
# Masking
summary(bond_masking_A100 <- lmer(Pro_MaskWearing_scaled ~ bond_family + bond_friend +
                                    bond_country + bond_humanity  +
                                    demo_age + demo_gender + demo_education + GDP + Country_Name +
                                    (1|Country_Name), data=DatasetA_N100, na.action=na.exclude))
```

```
## Linear mixed model fit by REML. t-tests use Satterthwaite's method [
## lmerModLmerTest]
## Formula: Pro_MaskWearing_scaled ~ bond_family + bond_friend + bond_country +
##     bond_humanity + demo_age + demo_gender + demo_education +
##     GDP + Country_Name + (1 | Country_Name)
##    Data: DatasetA_N100
##
## REML criterion at convergence: 16034
##
## Scaled residuals:
##     Min      1Q  Median      3Q     Max
## -1.7693 -0.6815 -0.3060  0.5926  3.1777
##
## Random effects:
##  Groups       Name        Variance Std.Dev.
##  Country_Name (Intercept) 0.001071 0.03272
##  Residual                 0.887470 0.94206
## Number of obs: 5863, groups:  Country_Name, 11
##
## Fixed effects:
##                              Estimate Std. Error         df t value Pr(>|t|)
## (Intercept)                -3.864e+00  3.568e-01  5.842e+03 -10.829  < 2e-16
## bond_family1                1.399e-01  2.983e-02  5.842e+03   4.689 2.81e-06
## bond_friend1               -1.428e-02  4.431e-02  5.842e+03  -0.322  0.74733
## bond_country1               1.119e-02  4.414e-02  5.842e+03   0.253  0.79992
## bond_humanity1              3.462e-02  3.325e-02  5.842e+03   1.041  0.29772
## demo_age                    2.808e-03  1.010e-03  5.842e+03   2.779  0.00548
## demo_genderFemale           5.801e-02  2.590e-02  5.842e+03   2.239  0.02516
## demo_genderOther/non-binary -9.563e-03  1.363e-01  5.842e+03  -0.070  0.94409
## demo_educationundergraduate  3.889e-02  3.104e-02  5.842e+03   1.253  0.21036
## demo_educationpostgraduate  -6.366e-02  3.406e-02  5.842e+03  -1.869  0.06168
## demo_educationNA            3.298e-01  1.515e-01  5.842e+03   2.176  0.02958
## GDP                         6.153e-05  5.920e-06  5.842e+03  10.393  < 2e-16
## Country_NameBrazil          3.365e+00  3.137e-01  5.842e+03  10.728  < 2e-16
## Country_NameCroatia         2.855e+00  2.789e-01  5.842e+03  10.237  < 2e-16
## Country_NameFinland         2.635e-01  1.043e-01  5.842e+03   2.527  0.01154
## Country_NameFrance          1.008e+00  1.454e-01  5.842e+03   6.935 4.51e-12
## Country_NameGermany         6.035e-01  1.073e-01  5.842e+03   5.625 1.94e-08
## Country_NameItaly           2.276e+00  1.694e-01  5.842e+03  13.433  < 2e-16
## Country_NameNew Zealand     8.759e-01  1.363e-01  5.842e+03   6.425 1.43e-10
## Country_NamePortugal        2.193e+00  2.270e-01  5.842e+03   9.661  < 2e-16
## Country_NameUK              8.979e-01  1.200e-01  5.842e+03   7.485 8.22e-14
##
## (Intercept)                ***
## bond_family1               ***
## bond_friend1
## bond_country1
## bond_humanity1
## demo_age                   **
## demo_genderFemale          *
## demo_genderOther/non-binary
## demo_educationundergraduate
```

```
## demo_educationpostgraduate  .
## demo_educationNA            *
## GDP                         ***
## Country_NameBrazil          ***
## Country_NameCroatia         ***
## Country_NameFinland         *
## Country_NameFrance          ***
## Country_NameGermany         ***
## Country_NameItaly           ***
## Country_NameNew Zealand     ***
## Country_NamePortugal        ***
## Country_NameUK              ***
## ---
## Signif. codes:  0 '***' 0.001 '**' 0.01 '*' 0.05 '.' 0.1 ' ' 1
## fit warnings:
## fixed-effect model matrix is rank deficient so dropping 1 column / coefficient
## Some predictor variables are on very different scales: consider rescaling
## optimizer (nloptwrap) convergence code: 0 (OK)
## unable to evaluate scaled gradient
##  Hessian is numerically singular: parameters are not uniquely determined
```

```
anova(bond_masking_A100)
```

```
## Type III Analysis of Variance Table with Satterthwaite's method
##                  Sum Sq Mean Sq NumDF DenDF  F value    Pr(>F)
## bond_family     19.513  19.513     1  5842  21.9878 2.806e-06 ***
## bond_friend      0.092   0.092     1  5842   0.1038 0.7473325
## bond_country     0.057   0.057     1  5842   0.0642 0.7999157
## bond_humanity    0.963   0.963     1  5842   1.0846 0.2977150
## demo_age         6.852   6.852     1  5842   7.7209 0.0054760 **
## demo_gender      4.537   2.269     2  5842   2.5563 0.0776810 .
## demo_education  14.534   4.845     3  5842   5.4592 0.0009585 ***
## GDP             95.859  95.859     1  5842 108.0143 < 2.2e-16 ***
## Country_Name   293.070  32.563     9  5842  36.6923 < 2.2e-16 ***
## ---
## Signif. codes:  0 '***' 0.001 '**' 0.01 '*' 0.05 '.' 0.1 ' ' 1
```

Hypothesis 1b, Dataset A

```
# Distancing
summary(bond_mult_distancing_A100 <- lmer(Pro_Distancing_scaled ~ bond_mult +
                                           demo_age + demo_gender + demo_education + GDP +
                                    (1|Country_Name), data=DatasetA_N100, na.action=na.exclude))
```

```
## Linear mixed model fit by REML. t-tests use Satterthwaite's method [
## lmerModLmerTest]
## Formula: Pro_Distancing_scaled ~ bond_mult + demo_age + demo_gender +
##     demo_education + GDP + (1 | Country_Name)
##    Data: DatasetA_N100
##
## REML criterion at convergence: 16416
```

```
##
## Scaled residuals:
##     Min      1Q  Median      3Q     Max
## -6.7360 -0.2093  0.3560  0.5655  1.1625
##
## Random effects:
##  Groups       Name        Variance Std.Dev.
##  Country_Name (Intercept) 0.0398   0.1995
##  Residual                 0.9385   0.9688
## Number of obs: 5883, groups:  Country_Name, 11
##
## Fixed effects:
##                               Estimate Std. Error         df t value Pr(>|t|)
## (Intercept)                 -2.752e-01  1.609e-01  9.599e+00  -1.710  0.11924
## bond_mult1                   7.797e-02  2.926e-02  5.867e+03   2.665  0.00773
## bond_mult2                   7.380e-02  4.291e-02  5.867e+03   1.720  0.08549
## bond_mult3                   1.356e-01  6.986e-02  5.866e+03   1.941  0.05231
## bond_mult4                   1.139e-01  1.197e-01  5.870e+03   0.952  0.34130
## demo_age                     6.918e-04  1.029e-03  5.833e+03   0.673  0.50122
## demo_genderFemale            1.553e-01  2.652e-02  5.869e+03   5.858 4.94e-09
## demo_genderOther/non-binary  6.425e-02  1.374e-01  5.862e+03   0.468  0.64003
## demo_educationundergraduate  1.382e-01  3.178e-02  5.870e+03   4.348 1.40e-05
## demo_educationpostgraduate   1.039e-01  3.482e-02  5.870e+03   2.985  0.00285
## demo_educationNA            -1.263e-01  1.522e-01  5.864e+03  -0.830  0.40661
## GDP                          3.358e-08  3.844e-06  8.268e+00   0.009  0.99324
##
## (Intercept)
## bond_mult1                  **
## bond_mult2                  .
## bond_mult3                  .
## bond_mult4
## demo_age
## demo_genderFemale           ***
## demo_genderOther/non-binary
## demo_educationundergraduate ***
## demo_educationpostgraduate  **
## demo_educationNA
## GDP
## ---
## Signif. codes:  0 '***' 0.001 '**' 0.01 '*' 0.05 '.' 0.1 ' ' 1
##
## Correlation of Fixed Effects:
##             (Intr) bnd_m1 bnd_m2 bnd_m3 bnd_m4 demo_g dm_gnF dm_O/- dm_dctnn
## bond_mult1  -0.059
## bond_mult2  -0.029  0.263
## bond_mult3  -0.011  0.170  0.134
## bond_mult4  -0.007  0.109  0.090  0.072
## demo_age    -0.198 -0.074 -0.107 -0.124 -0.116
## dem_gndrFml -0.057 -0.033 -0.062 -0.055 -0.022 -0.025
## dm_gndrOt/- -0.020 -0.007 -0.012  0.002 -0.002  0.035  0.110
## dm_dctnndrg -0.106  0.024  0.004  0.026  0.002 -0.068 -0.017  0.016
## dm_dctnpstg -0.076  0.009  0.006  0.019  0.030 -0.168 -0.041  0.006  0.569
## demo_dctnNA -0.016 -0.006 -0.010 -0.064 -0.026 -0.018 -0.024  0.004  0.115
## GDP         -0.879  0.016  0.016  0.013  0.018 -0.018 -0.024 -0.008 -0.002
```

```
##              dm_dctnp dm_dNA
## bond_mult1
## bond_mult2
## bond_mult3
## bond_mult4
## demo_age
## dem_gndrFml
## dm_gndrOt/-
## dm_dctnndrg
## dm_dctnpstg
## demo_dctnNA  0.112
## GDP          0.005    0.001
## fit warnings:
## Some predictor variables are on very different scales: consider rescaling
```

```
anova(bond_mult_distancing_A100)
```

```
## Type III Analysis of Variance Table with Satterthwaite's method
##                 Sum Sq Mean Sq NumDF  DenDF F value    Pr(>F)
## bond_mult        9.858  2.4645     4 5866.6  2.6260   0.03285 *
## demo_age         0.425  0.4246     1 5833.5  0.4524   0.50122
## demo_gender     32.236 16.1181     2 5866.0 17.1738 3.658e-08 ***
## demo_education 19.889  6.6297     3 5865.4  7.0639 9.774e-05 ***
## GDP              0.000  0.0001     1    8.3  0.0001   0.99324
## ---
## Signif. codes:  0 '***' 0.001 '**' 0.01 '*' 0.05 '.' 0.1 ' ' 1
```

```
# Hygiene
summary(bond_mult_hygiene_A100 <- lmer(Pro_Hygiene_scaled ~ bond_mult +
                                    demo_age + demo_gender + demo_education + GDP +
                                    (1|Country_Name), data=DatasetA_N100, na.action=na.exclude))
```

```
## Linear mixed model fit by REML. t-tests use Satterthwaite's method [
## lmerModLmerTest]
## Formula:
## Pro_Hygiene_scaled ~ bond_mult + demo_age + demo_gender + demo_education +
##     GDP + (1 | Country_Name)
##    Data: DatasetA_N100
##
## REML criterion at convergence: 16048.4
##
## Scaled residuals:
##     Min      1Q  Median      3Q     Max
## -3.5974 -0.6401  0.0690  0.7166  2.6140
##
## Random effects:
##  Groups       Name        Variance Std.Dev.
##  Country_Name (Intercept) 0.07215  0.2686
##  Residual                 0.88689  0.9417
## Number of obs: 5868, groups:  Country_Name, 11
##
## Fixed effects:
##                                Estimate Std. Error         df t value Pr(>|t|)
```

```
## (Intercept)                 -3.908e-01  2.103e-01  9.774e+00  -1.858  0.09346
## bond_mult1                   1.754e-01  2.848e-02  5.850e+03   6.159 7.81e-10
## bond_mult2                   2.494e-01  4.183e-02  5.850e+03   5.963 2.63e-09
## bond_mult3                   3.977e-01  6.793e-02  5.849e+03   5.855 5.02e-09
## bond_mult4                   3.043e-01  1.165e-01  5.853e+03   2.613  0.00900
## demo_age                     6.324e-03  1.003e-03  5.853e+03   6.308 3.03e-10
## demo_genderFemale            2.749e-01  2.582e-02  5.852e+03  10.647  < 2e-16
## demo_genderOther/non-binary -1.403e-01  1.336e-01  5.848e+03  -1.051  0.29341
## demo_educationundergraduate -1.128e-02  3.097e-02  5.855e+03  -0.364  0.71586
## demo_educationpostgraduate  -1.017e-01  3.392e-02  5.856e+03  -2.999  0.00272
## demo_educationNA             3.830e-01  1.480e-01  5.848e+03   2.588  0.00969
## GDP                         -4.212e-06  5.109e-06  9.013e+00  -0.824  0.43094
##
## (Intercept)                 .
## bond_mult1                  ***
## bond_mult2                  ***
## bond_mult3                  ***
## bond_mult4                  **
## demo_age                    ***
## demo_genderFemale           ***
## demo_genderOther/non-binary
## demo_educationundergraduate
## demo_educationpostgraduate  **
## demo_educationNA            **
## GDP
## ---
## Signif. codes:  0 '***' 0.001 '**' 0.01 '*' 0.05 '.' 0.1 ' ' 1
##
## Correlation of Fixed Effects:
##             (Intr) bnd_m1 bnd_m2 bnd_m3 bnd_m4 demo_g dm_gnF dm_O/- dm_dctnn
## bond_mult1  -0.044
## bond_mult2  -0.022  0.263
## bond_mult3  -0.008  0.171  0.134
## bond_mult4  -0.006  0.109  0.090  0.073
## demo_age    -0.147 -0.074 -0.106 -0.124 -0.115
## dem_gndrFml -0.042 -0.032 -0.063 -0.056 -0.023 -0.024
## dm_gndrOt/- -0.015 -0.007 -0.012  0.002 -0.002  0.035  0.110
## dm_dctnndrg -0.078  0.022  0.005  0.027 -0.001 -0.070 -0.020  0.016
## dm_dctnpstg -0.057  0.007  0.006  0.020  0.029 -0.170 -0.044  0.006  0.569
## demo_dctnNA -0.012 -0.006 -0.010 -0.063 -0.026 -0.019 -0.024  0.004  0.115
## GDP         -0.896  0.011  0.012  0.010  0.014 -0.014 -0.017 -0.006 -0.002
##             dm_dctnp dm_dNA
## bond_mult1
## bond_mult2
## bond_mult3
## bond_mult4
## demo_age
## dem_gndrFml
## dm_gndrOt/-
## dm_dctnndrg
## dm_dctnpstg
## demo_dctnNA  0.113
## GDP          0.004    0.001
## fit warnings:
```

```
## Some predictor variables are on very different scales: consider rescaling
```

```
anova(bond_mult_hygiene_A100)
```

```
## Type III Analysis of Variance Table with Satterthwaite's method
##                 Sum Sq Mean Sq NumDF  DenDF F value    Pr(>F)
## bond_mult       71.224  17.806     4 5850.2 20.0770 < 2.2e-16 ***
## demo_age        35.293  35.293     1 5852.9 39.7947 3.028e-10 ***
## demo_gender    104.959  52.479     2 5849.9 59.1725 < 2.2e-16 ***
## demo_education  17.537   5.846     3 5853.0  6.5913 0.0001919 ***
## GDP              0.603   0.603     1    9.0  0.6798 0.4309430
## ---
## Signif. codes:  0 '***' 0.001 '**' 0.01 '*' 0.05 '.' 0.1 ' ' 1
```

```
# Masking
summary(bond_mult_masking_A100 <- lmer(Pro_MaskWearing_scaled ~ bond_mult +
                                  demo_age + demo_gender + demo_education + GDP +
                                  (1|Country_Name), data=DatasetA_N100, na.action=na.exclude))
```

```
## Linear mixed model fit by REML. t-tests use Satterthwaite's method [
## lmerModLmerTest]
## Formula: Pro_MaskWearing_scaled ~ bond_mult + demo_age + demo_gender +
##     demo_education + GDP + (1 | Country_Name)
##    Data: DatasetA_N100
##
## REML criterion at convergence: 16080.3
##
## Scaled residuals:
##     Min      1Q  Median      3Q     Max
## -1.8644 -0.6863 -0.3038  0.5941  3.1393
##
## Random effects:
##  Groups       Name        Variance Std.Dev.
##  Country_Name (Intercept) 0.09235  0.3039
##  Residual                 0.88851  0.9426
## Number of obs: 5875, groups:  Country_Name, 11
##
## Fixed effects:
##                              Estimate Std. Error         df t value Pr(>|t|)
## (Intercept)                -7.079e-02  2.361e-01  9.721e+00  -0.300  0.77066
## bond_mult1                  6.575e-02  2.849e-02  5.857e+03   2.308  0.02106
## bond_mult2                  1.353e-01  4.178e-02  5.857e+03   3.239  0.00121
## bond_mult3                  1.253e-01  6.823e-02  5.856e+03   1.837  0.06632
## bond_mult4                  1.857e-01  1.165e-01  5.859e+03   1.594  0.11106
## demo_age                    3.166e-03  1.003e-03  5.863e+03   3.157  0.00160
## demo_genderFemale           6.136e-02  2.583e-02  5.858e+03   2.376  0.01755
## demo_genderOther/non-binary -3.396e-02  1.350e-01  5.855e+03  -0.252  0.80139
## demo_educationundergraduate  3.041e-02  3.096e-02  5.861e+03   0.982  0.32613
## demo_educationpostgraduate  -6.764e-02  3.394e-02  5.862e+03  -1.993  0.04630
## demo_educationNA             2.793e-01  1.481e-01  5.855e+03   1.885  0.05946
## GDP                         -5.929e-06  5.762e-06  9.118e+00  -1.029  0.32999
##
## (Intercept)
```

```
## bond_mult1                 *
## bond_mult2                 **
## bond_mult3                 .
## bond_mult4
## demo_age                   **
## demo_genderFemale          *
## demo_genderOther/non-binary
## demo_educationundergraduate
## demo_educationpostgraduate  *
## demo_educationNA            .
## GDP
## ---
## Signif. codes:  0 '***' 0.001 '**' 0.01 '*' 0.05 '.' 0.1 ' ' 1
##
## Correlation of Fixed Effects:
##             (Intr) bnd_m1 bnd_m2 bnd_m3 bnd_m4 demo_g dm_gnF dm_O/- dm_dctnn
## bond_mult1  -0.039
## bond_mult2  -0.019  0.262
## bond_mult3  -0.007  0.169  0.133
## bond_mult4  -0.005  0.109  0.090  0.071
## demo_age    -0.131 -0.073 -0.106 -0.121 -0.116
## dem_gndrFml -0.038 -0.032 -0.061 -0.056 -0.022 -0.025
## dm_gndrOt/- -0.013 -0.008 -0.012  0.002 -0.002  0.034  0.108
## dm_dctnndrg -0.070  0.023  0.003  0.027  0.002 -0.068 -0.017  0.019
## dm_dctnpstg -0.051  0.007  0.005  0.018  0.030 -0.169 -0.043  0.007  0.569
## demo_dctnNA -0.010 -0.006 -0.010 -0.064 -0.026 -0.019 -0.024  0.005  0.115
## GDP         -0.900  0.010  0.010  0.009  0.012 -0.012 -0.015 -0.005 -0.002
##             dm_dctnp dm_dNA
## bond_mult1
## bond_mult2
## bond_mult3
## bond_mult4
## demo_age
## dem_gndrFml
## dm_gndrOt/-
## dm_dctnndrg
## dm_dctnpstg
## demo_dctnNA  0.113
## GDP          0.003    0.001
## fit warnings:
## Some predictor variables are on very different scales: consider rescaling
```

```
anova(bond_mult_masking_A100)
```

```
## Type III Analysis of Variance Table with Satterthwaite's method
##                 Sum Sq Mean Sq NumDF  DenDF F value   Pr(>F)
## bond_mult      13.7864  3.4466     4 5856.7  3.8791 0.003772 **
## demo_age        8.8543  8.8543     1 5862.5  9.9653 0.001603 **
## demo_gender     5.2474  2.6237     2 5856.5  2.9529 0.052267 .
## demo_education 12.8376  4.2792     3 5859.6  4.8161 0.002373 **
## GDP             0.9408  0.9408     1    9.1  1.0589 0.329994
## ---
## Signif. codes:  0 '***' 0.001 '**' 0.01 '*' 0.05 '.' 0.1 ' ' 1
```

Hypothesis 2a, Dataset A

```r
# Anxiety
summary(bond_anx_A100 <- lmer(anx_sum1_scaled ~ bond_family + bond_friend +
                                     bond_country + bond_humanity  +
                                     demo_age + demo_gender + demo_education + GDP + Country_Name +
                                   (1|Country_Name), data=DatasetA_N100, na.action=na.exclude))
```

```
## Linear mixed model fit by REML. t-tests use Satterthwaite's method [
## lmerModLmerTest]
## Formula: anx_sum1_scaled ~ bond_family + bond_friend + bond_country +
##     bond_humanity + demo_age + demo_gender + demo_education +
##     GDP + Country_Name + (1 | Country_Name)
##    Data: DatasetA_N100
##
## REML criterion at convergence: 15894.1
##
## Scaled residuals:
##     Min      1Q  Median      3Q     Max
## -2.6093 -0.7427 -0.0701  0.6561  3.6060
##
## Random effects:
##  Groups       Name        Variance Std.Dev.
##  Country_Name (Intercept) 0.0000   0.0000
##  Residual                 0.8585   0.9266
## Number of obs: 5883, groups:  Country_Name, 11
##
## Fixed effects:
##                               Estimate Std. Error         df t value Pr(>|t|)
## (Intercept)                 -2.763e+00  2.637e-01  5.862e+03 -10.479  < 2e-16
## bond_family1                -1.002e-01  2.928e-02  5.862e+03  -3.421 0.000629
## bond_friend1                -1.205e-01  4.349e-02  5.862e+03  -2.771 0.005599
## bond_country1               -1.436e-01  4.324e-02  5.862e+03  -3.321 0.000901
## bond_humanity1              -8.321e-02  3.264e-02  5.862e+03  -2.550 0.010808
## demo_age                    -9.052e-03  9.911e-04  5.862e+03  -9.134  < 2e-16
## demo_genderFemale            4.740e-01  2.544e-02  5.862e+03  18.633  < 2e-16
## demo_genderOther/non-binary  4.558e-01  1.328e-01  5.862e+03   3.433 0.000601
## demo_educationundergraduate -3.265e-02  3.048e-02  5.862e+03  -1.071 0.283995
## demo_educationpostgraduate  -3.634e-02  3.343e-02  5.862e+03  -1.087 0.277144
## demo_educationNA             1.849e-01  1.490e-01  5.862e+03   1.241 0.214703
## GDP                          5.113e-05  4.216e-06  5.862e+03  12.126  < 2e-16
## Country_NameBrazil           2.839e+00  2.290e-01  5.862e+03  12.397  < 2e-16
## Country_NameCroatia          2.059e+00  2.087e-01  5.862e+03   9.864  < 2e-16
## Country_NameFinland         -1.522e-02  8.876e-02  5.862e+03  -0.171 0.863855
## Country_NameFrance           6.094e-01  1.160e-01  5.862e+03   5.255 1.54e-07
## Country_NameGermany          3.585e-01  8.713e-02  5.862e+03   4.114 3.94e-05
## Country_NameItaly            1.133e+00  1.253e-01  5.862e+03   9.041  < 2e-16
## Country_NameNew Zealand      3.927e-01  1.114e-01  5.862e+03   3.524 0.000429
## Country_NamePortugal         1.697e+00  1.683e-01  5.862e+03  10.083  < 2e-16
## Country_NameUK               8.430e-01  8.998e-02  5.862e+03   9.368  < 2e-16
##
## (Intercept)                 ***
## bond_family1                ***
```

```
## bond_friend1                **
## bond_country1               ***
## bond_humanity1              *
## demo_age                    ***
## demo_genderFemale           ***
## demo_genderOther/non-binary ***
## demo_educationundergraduate
## demo_educationpostgraduate
## demo_educationNA
## GDP                         ***
## Country_NameBrazil          ***
## Country_NameCroatia         ***
## Country_NameFinland
## Country_NameFrance          ***
## Country_NameGermany         ***
## Country_NameItaly           ***
## Country_NameNew Zealand     ***
## Country_NamePortugal        ***
## Country_NameUK              ***
## ---
## Signif. codes:  0 '***' 0.001 '**' 0.01 '*' 0.05 '.' 0.1 ' ' 1
## fit warnings:
## fixed-effect model matrix is rank deficient so dropping 1 column / coefficient
## Some predictor variables are on very different scales: consider rescaling
## optimizer (nloptwrap) convergence code: 0 (OK)
## boundary (singular) fit: see ?isSingular
```

```
anova(bond_anx_A100)
```

```
## Type III Analysis of Variance Table with Satterthwaite's method
##                 Sum Sq Mean Sq NumDF DenDF  F value    Pr(>F)
## bond_family      10.05  10.046     1  5862  11.7016 0.0006287 ***
## bond_friend       6.59   6.594     1  5862   7.6806 0.0055993 **
## bond_country      9.47   9.471     1  5862  11.0319 0.0009011 ***
## bond_humanity     5.58   5.581     1  5862   6.5007 0.0108083 *
## demo_age         71.62  71.625     1  5862  83.4274 < 2.2e-16 ***
## demo_gender     299.82 149.910     2  5862 174.6134 < 2.2e-16 ***
## demo_education    2.97   0.990     3  5862   1.1536 0.3259690
## GDP             126.24 126.238     1  5862 147.0400 < 2.2e-16 ***
## Country_Name    323.91  35.990     9  5862  41.9209 < 2.2e-16 ***
## ---
## Signif. codes:  0 '***' 0.001 '**' 0.01 '*' 0.05 '.' 0.1 ' ' 1
```

```
# Depression
summary(bond_dep_A100 <- lmer(dep_sum1_scaled ~ bond_family + bond_friend +
                                      bond_country + bond_humanity  +
                                      demo_age + demo_gender + demo_education + GDP + Country_Name +
                                    (1|Country_Name), data=DatasetA_N100, na.action=na.exclude))
```

```
## Linear mixed model fit by REML. t-tests use Satterthwaite's method [
## lmerModLmerTest]
## Formula: dep_sum1_scaled ~ bond_family + bond_friend + bond_country +
##     bond_humanity + demo_age + demo_gender + demo_education +
```

```
##     GDP + Country_Name + (1 | Country_Name)
##    Data: DatasetA_N100
##
## REML criterion at convergence: 16274
##
## Scaled residuals:
##     Min      1Q  Median      3Q     Max
## -2.6270 -0.7580 -0.0591  0.6646  4.0202
##
## Random effects:
##  Groups       Name        Variance Std.Dev.
##  Country_Name (Intercept) 0.001171 0.03422
##  Residual                 0.914721 0.95641
## Number of obs: 5886, groups:  Country_Name, 11
##
## Fixed effects:
##                              Estimate Std. Error         df t value Pr(>|t|)
## (Intercept)                -7.868e-01  3.666e-01  1.653e-09  -2.147 1.000000
## bond_family1               -1.723e-01  3.022e-02  5.865e+03  -5.703 1.24e-08
## bond_friend1               -2.636e-01  4.489e-02  5.865e+03  -5.871 4.57e-09
## bond_country1              -1.029e-01  4.463e-02  5.865e+03  -2.307 0.021109
## bond_humanity1             -1.699e-01  3.369e-02  5.865e+03  -5.043 4.73e-07
## demo_age                   -5.960e-03  1.023e-03  5.865e+03  -5.827 5.96e-09
## demo_genderFemale           2.428e-01  2.625e-02  5.865e+03   9.249  < 2e-16
## demo_genderOther/non-binary  5.143e-01  1.370e-01  5.865e+03   3.753 0.000177
## demo_educationundergraduate -1.186e-01  3.145e-02  5.865e+03  -3.771 0.000164
## demo_educationpostgraduate  -1.408e-01  3.450e-02  5.865e+03  -4.081 4.54e-05
## demo_educationNA           -5.865e-03  1.538e-01  5.865e+03  -0.038 0.969587
## GDP                         1.790e-05  6.091e-06  1.390e-09   2.939 1.000000
## Country_NameBrazil          1.039e+00  3.225e-01  1.555e-09   3.221 1.000000
## Country_NameCroatia         6.510e-01  2.865e-01  1.763e-09   2.272 1.000000
## Country_NameFinland        -9.354e-02  1.066e-01  4.869e-09  -0.877 1.000000
## Country_NameFrance         -1.436e-02  1.489e-01  2.659e-09  -0.096 1.000000
## Country_NameGermany         7.601e-02  1.099e-01  3.050e-09   0.692 1.000000
## Country_NameItaly           8.624e-01  1.741e-01  1.658e-09   4.955 1.000000
## Country_NameNew Zealand     9.680e-02  1.396e-01  3.226e-09   0.693 1.000000
## Country_NamePortugal        4.391e-01  2.331e-01  1.680e-09   1.884 1.000000
## Country_NameUK              4.174e-01  1.232e-01  1.786e-09   3.389 1.000000
##
## (Intercept)
## bond_family1               ***
## bond_friend1               ***
## bond_country1              *
## bond_humanity1             ***
## demo_age                   ***
## demo_genderFemale          ***
## demo_genderOther/non-binary ***
## demo_educationundergraduate ***
## demo_educationpostgraduate  ***
## demo_educationNA
## GDP
## Country_NameBrazil
## Country_NameCroatia
## Country_NameFinland
```

```
## Country_NameFrance
## Country_NameGermany
## Country_NameItaly
## Country_NameNew Zealand
## Country_NamePortugal
## Country_NameUK
## ---
## Signif. codes:  0 '***' 0.001 '**' 0.01 '*' 0.05 '.' 0.1 ' ' 1
## fit warnings:
## fixed-effect model matrix is rank deficient so dropping 1 column / coefficient
## Some predictor variables are on very different scales: consider rescaling
```

```
anova(bond_dep_A100)
```

```
## Type III Analysis of Variance Table with Satterthwaite's method
##                 Sum Sq Mean Sq NumDF DenDF F value    Pr(>F)
## bond_family     29.747  29.747     1  5865 32.5207 1.237e-08 ***
## bond_friend     31.528  31.528     1  5865 34.4676 4.572e-09 ***
## bond_country     4.867   4.867     1  5865  5.3206 0.0211089 *
## bond_humanity   23.259  23.259     1  5865 25.4273 4.732e-07 ***
## demo_age        31.054  31.054     1  5865 33.9493 5.957e-09 ***
## demo_gender     85.261  42.631     2  5865 46.6050 < 2.2e-16 ***
## demo_education  18.323   6.108     3  5865  6.6772 0.0001698 ***
## GDP              7.900   7.900     1     0  8.6366 1.0000000
## Country_Name   123.780  13.753     9     0 15.0356 0.9999999
## ---
## Signif. codes:  0 '***' 0.001 '**' 0.01 '*' 0.05 '.' 0.1 ' ' 1
```

Hypothesis 2b, Dataset A

```
# Anxiety
summary(bond_mult_anx_A100 <- lmer(anx_sum1_scaled ~ bond_mult +
                                      demo_age + demo_gender + demo_education + GDP +
                                    (1|Country_Name), data=DatasetA_N100, na.action=na.exclude))
```

```
## Linear mixed model fit by REML. t-tests use Satterthwaite's method [
## lmerModLmerTest]
## Formula:
## anx_sum1_scaled ~ bond_mult + demo_age + demo_gender + demo_education +
##     GDP + (1 | Country_Name)
##    Data: DatasetA_N100
##
## REML criterion at convergence: 15927.2
##
## Scaled residuals:
##     Min      1Q  Median      3Q     Max
## -2.6216 -0.7385 -0.0741  0.6567  3.5852
##
## Random effects:
##  Groups       Name        Variance Std.Dev.
##  Country_Name (Intercept) 0.06304  0.2511
```

```
##  Residual                 0.85814  0.9264
## Number of obs: 5895, groups:  Country_Name, 11
##
## Fixed effects:
##                               Estimate Std. Error         df t value Pr(>|t|)
## (Intercept)                  1.493e-01  1.972e-01  9.831e+00   0.757 0.466855
## bond_mult1                  -1.517e-01  2.796e-02  5.878e+03  -5.426 6.00e-08
## bond_mult2                  -1.997e-01  4.102e-02  5.877e+03  -4.869 1.15e-06
## bond_mult3                  -3.405e-01  6.652e-02  5.877e+03  -5.119 3.16e-07
## bond_mult4                  -3.559e-01  1.138e-01  5.880e+03  -3.128 0.001769
## demo_age                    -9.045e-03  9.826e-04  5.878e+03  -9.205  < 2e-16
## demo_genderFemale            4.736e-01  2.534e-02  5.879e+03  18.689  < 2e-16
## demo_genderOther/non-binary  4.451e-01  1.314e-01  5.875e+03   3.388 0.000708
## demo_educationundergraduate -3.264e-02  3.037e-02  5.882e+03  -1.075 0.282514
## demo_educationpostgraduate  -3.425e-02  3.328e-02  5.883e+03  -1.029 0.303408
## demo_educationNA             2.322e-01  1.456e-01  5.875e+03   1.595 0.110684
## GDP                         -2.838e-06  4.783e-06  8.993e+00  -0.593 0.567503
##
## (Intercept)
## bond_mult1                  ***
## bond_mult2                  ***
## bond_mult3                  ***
## bond_mult4                  **
## demo_age                    ***
## demo_genderFemale           ***
## demo_genderOther/non-binary ***
## demo_educationundergraduate
## demo_educationpostgraduate
## demo_educationNA
## GDP
## ---
## Signif. codes:  0 '***' 0.001 '**' 0.01 '*' 0.05 '.' 0.1 ' ' 1
##
## Correlation of Fixed Effects:
##             (Intr) bnd_m1 bnd_m2 bnd_m3 bnd_m4 demo_g dm_gnF dm_O/- dm_dctnn
## bond_mult1  -0.046
## bond_mult2  -0.023  0.263
## bond_mult3  -0.008  0.171  0.134
## bond_mult4  -0.006  0.110  0.090  0.073
## demo_age    -0.154 -0.073 -0.105 -0.126 -0.117
## dem_gndrFml -0.044 -0.033 -0.061 -0.054 -0.023 -0.026
## dm_gndrOt/- -0.016 -0.007 -0.012  0.002 -0.002  0.035  0.109
## dm_dctnndrg -0.082  0.023  0.004  0.026  0.001 -0.068 -0.017  0.016
## dm_dctnpstg -0.059  0.008  0.005  0.018  0.031 -0.169 -0.043  0.006  0.568
## demo_dctnNA -0.012 -0.006 -0.010 -0.063 -0.026 -0.018 -0.024  0.004  0.115
## GDP         -0.894  0.012  0.012  0.010  0.014 -0.014 -0.018 -0.006 -0.002
##             dm_dctnp dm_dNA
## bond_mult1
## bond_mult2
## bond_mult3
## bond_mult4
## demo_age
## dem_gndrFml
## dm_gndrOt/-
```

```
## dm_dctnndrg
## dm_dctnpstg
## demo_dctnNA  0.112
## GDP          0.004    0.001
## fit warnings:
## Some predictor variables are on very different scales: consider rescaling
```

```
anova(bond_mult_anx_A100)
```

```
## Type III Analysis of Variance Table with Satterthwaite's method
##                 Sum Sq Mean Sq NumDF  DenDF  F value    Pr(>F)
## bond_mult       53.378  13.345     4 5877.4  15.5507 1.164e-12 ***
## demo_age        72.719  72.719     1 5877.7  84.7407 < 2.2e-16 ***
## demo_gender    301.286 150.643     2 5877.1 175.5465 < 2.2e-16 ***
## demo_education   3.879   1.293     3 5880.1   1.5068    0.2106
## GDP              0.302   0.302     1    9.0   0.3522    0.5675
## ---
## Signif. codes:  0 '***' 0.001 '**' 0.01 '*' 0.05 '.' 0.1 ' ' 1
```

```
# Depression
summary(bond_mult_dep_A100 <- lmer(dep_sum1_scaled ~ bond_mult +
                                      demo_age + demo_gender + demo_education + GDP +
                                    (1|Country_Name), data=DatasetA_N100, na.action=na.exclude))
```

```
## Linear mixed model fit by REML. t-tests use Satterthwaite's method [
## lmerModLmerTest]
## Formula:
## dep_sum1_scaled ~ bond_mult + demo_age + demo_gender + demo_education +
##     GDP + (1 | Country_Name)
##    Data: DatasetA_N100
##
## REML criterion at convergence: 16303.9
##
## Scaled residuals:
##     Min      1Q  Median      3Q     Max
## -2.6311 -0.7683 -0.0650  0.6676  4.0083
##
## Random effects:
##  Groups       Name        Variance Std.Dev.
##  Country_Name (Intercept) 0.0493   0.2220
##  Residual                 0.9145   0.9563
## Number of obs: 5897, groups:  Country_Name, 11
##
## Fixed effects:
##                                Estimate Std. Error         df t value Pr(>|t|)
## (Intercept)                   2.677e-01  1.767e-01  1.003e+01   1.514 0.160761
## bond_mult1                   -2.100e-01  2.885e-02  5.881e+03  -7.277 3.85e-13
## bond_mult2                   -3.855e-01  4.234e-02  5.880e+03  -9.105  < 2e-16
## bond_mult3                   -4.609e-01  6.866e-02  5.879e+03  -6.713 2.08e-11
## bond_mult4                   -6.064e-01  1.174e-01  5.884e+03  -5.164 2.50e-07
## demo_age                     -5.875e-03  1.014e-03  5.868e+03  -5.794 7.21e-09
## demo_genderFemale             2.391e-01  2.615e-02  5.883e+03   9.145  < 2e-16
## demo_genderOther/non-binary   4.882e-01  1.356e-01  5.877e+03   3.600 0.000321
```

```
## demo_educationundergraduate -1.199e-01  3.135e-02  5.885e+03  -3.826 0.000132
## demo_educationpostgraduate  -1.417e-01  3.434e-02  5.885e+03  -4.125 3.76e-05
## demo_educationNA             1.122e-02  1.503e-01  5.878e+03   0.075 0.940457
## GDP                         -1.571e-06  4.254e-06  8.905e+00  -0.369 0.720569
##
## (Intercept)
## bond_mult1                  ***
## bond_mult2                  ***
## bond_mult3                  ***
## bond_mult4                  ***
## demo_age                    ***
## demo_genderFemale           ***
## demo_genderOther/non-binary ***
## demo_educationundergraduate ***
## demo_educationpostgraduate  ***
## demo_educationNA
## GDP
## ---
## Signif. codes:  0 '***' 0.001 '**' 0.01 '*' 0.05 '.' 0.1 ' ' 1
##
## Correlation of Fixed Effects:
##             (Intr) bnd_m1 bnd_m2 bnd_m3 bnd_m4 demo_g dm_gnF dm_O/- dm_dctnn
## bond_mult1  -0.053
## bond_mult2  -0.026  0.263
## bond_mult3  -0.009  0.171  0.134
## bond_mult4  -0.007  0.110  0.090  0.073
## demo_age    -0.178 -0.074 -0.106 -0.126 -0.117
## dem_gndrFml -0.051 -0.033 -0.061 -0.054 -0.023 -0.026
## dm_gndrOt/- -0.018 -0.007 -0.012  0.002 -0.002  0.035  0.109
## dm_dctnndrg -0.095  0.023  0.004  0.026  0.001 -0.069 -0.017  0.016
## dm_dctnpstg -0.068  0.008  0.006  0.018  0.031 -0.169 -0.041  0.006  0.569
## demo_dctnNA -0.014 -0.006 -0.010 -0.063 -0.026 -0.018 -0.024  0.004  0.115
## GDP         -0.887  0.014  0.014  0.012  0.017 -0.016 -0.021 -0.007 -0.002
##             dm_dctnp dm_dNA
## bond_mult1
## bond_mult2
## bond_mult3
## bond_mult4
## demo_age
## dem_gndrFml
## dm_gndrOt/-
## dm_dctnndrg
## dm_dctnpstg
## demo_dctnNA  0.112
## GDP          0.004    0.001
## fit warnings:
## Some predictor variables are on very different scales: consider rescaling
```

```
anova(bond_mult_dep_A100)
```

```
## Type III Analysis of Variance Table with Satterthwaite's method
##                  Sum Sq Mean Sq NumDF  DenDF F value    Pr(>F)
## bond_mult       133.391  33.348     4 5880.2 36.4674 < 2.2e-16 ***
## demo_age         30.703  30.703     1 5868.0 33.5751 7.213e-09 ***
```

```
## demo_gender     82.727  41.363     2 5879.8 45.2332 < 2.2e-16 ***
## demo_education  18.911   6.304     3 5881.5  6.8935 0.0001247 ***
## GDP              0.125   0.125     1    8.9  0.1363 0.7205691
## ---
## Signif. codes:  0 '***' 0.001 '**' 0.01 '*' 0.05 '.' 0.1 ' ' 1
```

Table S11B: Main analyses only within N>100 countries, Dataset B

Hypothesis 1a, Dataset B

```
# Distancing
summary(bond_distancing_B100 <- lmer(comply_self_scale ~ bond_country + bond_gvmt  +
                                       age + gender + education + GDP + country_now_Name +
                                    (1|country_now_Name), data=DatasetB_N100, na.action=na.exclude))
```

```
## Linear mixed model fit by REML. t-tests use Satterthwaite's method [
## lmerModLmerTest]
## Formula: comply_self_scale ~ bond_country + bond_gvmt + age + gender +
##     education + GDP + country_now_Name + (1 | country_now_Name)
##    Data: DatasetB_N100
##
## REML criterion at convergence: 14839.2
##
## Scaled residuals:
##     Min      1Q  Median      3Q     Max
## -3.1681 -0.6819 -0.2977  0.7653  2.1340
##
## Random effects:
##  Groups           Name        Variance Std.Dev.
##  country_now_Name (Intercept) 0.1288   0.3588
##  Residual                     0.9311   0.9649
## Number of obs: 5334, groups:  country_now_Name, 11
##
## Fixed effects:
##                              Estimate Std. Error        df t value Pr(>|t|)
## (Intercept)                -2.980e-01  2.627e+00  5.315e+03  -0.113  0.90968
## bond_country1              -2.900e-02  3.680e-02  5.315e+03  -0.788  0.43070
## bond_gvmt1                  7.149e-02  6.140e-02  5.315e+03   1.164  0.24439
## age                         1.238e-03  1.023e-03  5.315e+03   1.211  0.22596
## genderwoman                 6.066e-02  3.014e-02  5.315e+03   2.013  0.04417 *
## gendernb                    3.539e-02  1.356e-01  5.315e+03   0.261  0.79405
## gendernone                  3.133e-01  1.567e-01  5.315e+03   2.000  0.04558 *
## educationundergraduate      5.390e-02  3.845e-02  5.315e+03   1.402  0.16104
## educationpostgraduate       1.127e-01  4.188e-02  5.315e+03   2.691  0.00714 **
## GDP                         2.578e-06  4.547e-05  5.315e+03   0.057  0.95479
## country_now_NameBangladesh -7.825e-02  2.549e+00  5.315e+03  -0.031  0.97551
## country_now_NameCanada      1.123e-01  7.887e-01  5.315e+03   0.142  0.88677
## country_now_NameFrance     -2.836e-02  9.488e-01  5.315e+03  -0.030  0.97616
## country_now_NameGermany    -6.328e-03  6.780e-01  5.315e+03  -0.009  0.99255
## country_now_NameItaly      -4.063e-02  1.250e+00  5.315e+03  -0.033  0.97407
## country_now_NamePeru        4.014e-02  2.377e+00  5.315e+03   0.017  0.98653
## country_now_NameSweden      2.528e-01  5.072e-01  5.315e+03   0.498  0.61821
```

```
## country_now_NameTurkey      4.225e-01  2.269e+00  5.315e+03   0.186  0.85226
## country_now_NameUK          6.429e-02  8.658e-01  5.315e+03   0.074  0.94082
## ---
## Signif. codes:  0 '***' 0.001 '**' 0.01 '*' 0.05 '.' 0.1 ' ' 1
## fit warnings:
## fixed-effect model matrix is rank deficient so dropping 1 column / coefficient
## Some predictor variables are on very different scales: consider rescaling
## optimizer (nloptwrap) convergence code: 0 (OK)
## unable to evaluate scaled gradient
## Model failed to converge: degenerate  Hessian with 1 negative eigenvalues
```

```
anova(bond_distancing_B100)
```

```
## Type III Analysis of Variance Table with Satterthwaite's method
##                  Sum Sq Mean Sq NumDF DenDF F value  Pr(>F)
## bond_country     0.5782  0.5782     1  5315  0.6210 0.43070
## bond_gvmt        1.2619  1.2619     1  5315  1.3554 0.24439
## age              1.3653  1.3653     1  5315  1.4664 0.22596
## gender           6.6334  2.2111     3  5315  2.3749 0.06815 .
## education        7.1992  3.5996     2  5315  3.8661 0.02100 *
## GDP              0.0030  0.0030     1  5315  0.0032 0.95479
## country_now_Name 1.5206  0.1690     9  5315  0.1815 0.99602
## ---
## Signif. codes:  0 '***' 0.001 '**' 0.01 '*' 0.05 '.' 0.1 ' ' 1
```

Hypothesis 1b, Dataset B

```
# Hypothesis 1b
summary(bond_mult_distancing_B100 <- lmer(comply_self_scale ~ bond_mult*country_now_Name  +
                                      age + gender + education + GDP +
                                   (1|country_now_Name), data=DatasetB_N100, na.action=na.exclude))
```

```
## Linear mixed model fit by REML. t-tests use Satterthwaite's method [
## lmerModLmerTest]
## Formula: comply_self_scale ~ bond_mult * country_now_Name + age + gender +
##     education + GDP + (1 | country_now_Name)
##    Data: DatasetB_N100
##
## REML criterion at convergence: 15375.4
##
## Scaled residuals:
##     Min      1Q  Median      3Q     Max
## -3.1921 -0.6857 -0.2740  0.7609  2.1665
##
## Random effects:
##  Groups           Name        Variance Std.Dev.
##  country_now_Name (Intercept) 0.0284   0.1685
##  Residual                     0.9308   0.9648
## Number of obs: 5534, groups:  country_now_Name, 11
##
## Fixed effects:
```

```
##                                           Estimate Std. Error         df t value
## (Intercept)                             -2.495e-01  2.024e-01  1.309e-07  -1.233
## bond_mult1                               3.823e-01  1.939e-01  5.495e+03   1.972
## bond_mult2                               3.465e-01  3.561e-01  5.495e+03   0.973
## country_now_NameBangladesh              -1.362e-01  2.704e-01  1.042e-07  -0.504
## country_now_NameCanada                   1.512e-01  2.825e-01  1.241e-07   0.535
## country_now_NameFrance                   1.625e-02  2.653e-01  9.650e-08   0.061
## country_now_NameGermany                  7.326e-02  2.679e-01  1.004e-07   0.273
## country_now_NameItaly                   -4.877e-02  2.791e-01  1.183e-07  -0.175
## country_now_NamePeru                     1.028e-01  2.638e-01  9.446e-08   0.389
## country_now_NameSweden                   3.886e-01  2.755e-01  1.122e-07   1.411
## country_now_NameTurkey                   4.364e-01  2.605e-01  8.981e-08   1.675
## country_now_NameUK                       1.444e-01  2.597e-01  8.864e-08   0.556
## country_now_NameUSA                      1.622e-01  2.622e-01  9.215e-08   0.618
## age                                      3.071e-04  1.013e-03  5.495e+03   0.303
## genderwoman                              5.035e-02  2.953e-02  5.495e+03   1.705
## gendernb                                -1.616e-02  1.331e-01  5.495e+03  -0.121
## gendernone                               3.359e-01  1.550e-01  5.495e+03   2.167
## educationundergraduate                   7.173e-02  3.764e-02  5.495e+03   1.906
## educationpostgraduate                    1.254e-01  4.127e-02  5.495e+03   3.038
## bond_mult1:country_now_NameBangladesh -3.951e-01  2.342e-01  5.495e+03  -1.687
## bond_mult2:country_now_NameBangladesh -4.033e-02  4.330e-01  5.495e+03  -0.093
## bond_mult1:country_now_NameCanada       -1.068e-01  3.078e-01  5.495e+03  -0.347
## bond_mult2:country_now_NameCanada       -1.486e-01  4.932e-01  5.495e+03  -0.301
## bond_mult1:country_now_NameFrance       -2.019e-01  2.331e-01  5.495e+03  -0.866
## bond_mult2:country_now_NameFrance       -2.890e-01  4.725e-01  5.495e+03  -0.612
## bond_mult1:country_now_NameGermany      -3.192e-01  2.946e-01  5.495e+03  -1.084
## bond_mult2:country_now_NameGermany      -1.386e-01  5.144e-01  5.495e+03  -0.269
## bond_mult1:country_now_NameItaly        -2.422e-01  3.267e-01  5.495e+03  -0.741
## bond_mult2:country_now_NameItaly         3.009e-01  4.910e-01  5.495e+03   0.613
## bond_mult1:country_now_NamePeru         -5.703e-01  2.118e-01  5.495e+03  -2.693
## bond_mult2:country_now_NamePeru         -5.890e-01  3.745e-01  5.495e+03  -1.573
## bond_mult1:country_now_NameSweden       -5.199e-01  2.980e-01  5.495e+03  -1.745
## bond_mult2:country_now_NameSweden       -2.729e-01  4.618e-01  5.495e+03  -0.591
## bond_mult1:country_now_NameTurkey       -4.488e-01  2.148e-01  5.495e+03  -2.090
## bond_mult2:country_now_NameTurkey       -4.660e-01  4.529e-01  5.495e+03  -1.029
## bond_mult1:country_now_NameUK           -3.657e-01  2.022e-01  5.495e+03  -1.808
## bond_mult2:country_now_NameUK           -2.199e-01  3.714e-01  5.495e+03  -0.592
## bond_mult1:country_now_NameUSA          -4.722e-01  2.375e-01  5.495e+03  -1.988
## bond_mult2:country_now_NameUSA          -6.431e-01  5.327e-01  5.495e+03  -1.207
##                                         Pr(>|t|)
## (Intercept)                              1.00000
## bond_mult1                               0.04872 *
## bond_mult2                               0.33045
## country_now_NameBangladesh               1.00000
## country_now_NameCanada                   1.00000
## country_now_NameFrance                   1.00000
## country_now_NameGermany                  1.00000
## country_now_NameItaly                    1.00000
## country_now_NamePeru                     1.00000
## country_now_NameSweden                   1.00000
## country_now_NameTurkey                   1.00000
## country_now_NameUK                       1.00000
## country_now_NameUSA                      1.00000
```

```
## age                                  0.76184
## genderwoman                          0.08822 .
## gendernb                             0.90335
## gendernone                           0.03024 *
## educationundergraduate               0.05673 .
## educationpostgraduate                0.00239 **
## bond_mult1:country_now_NameBangladesh  0.09162 .
## bond_mult2:country_now_NameBangladesh  0.92579
## bond_mult1:country_now_NameCanada      0.72855
## bond_mult2:country_now_NameCanada      0.76320
## bond_mult1:country_now_NameFrance      0.38633
## bond_mult2:country_now_NameFrance      0.54078
## bond_mult1:country_now_NameGermany     0.27862
## bond_mult2:country_now_NameGermany     0.78764
## bond_mult1:country_now_NameItaly       0.45856
## bond_mult2:country_now_NameItaly       0.54007
## bond_mult1:country_now_NamePeru        0.00710 **
## bond_mult2:country_now_NamePeru        0.11578
## bond_mult1:country_now_NameSweden      0.08112 .
## bond_mult2:country_now_NameSweden      0.55458
## bond_mult1:country_now_NameTurkey      0.03669 *
## bond_mult2:country_now_NameTurkey      0.30348
## bond_mult1:country_now_NameUK          0.07060 .
## bond_mult2:country_now_NameUK          0.55374
## bond_mult1:country_now_NameUSA         0.04682 *
## bond_mult2:country_now_NameUSA         0.22739
## ---
## Signif. codes:  0 '***' 0.001 '**' 0.01 '*' 0.05 '.' 0.1 ' ' 1
## fit warnings:
## fixed-effect model matrix is rank deficient so dropping 1 column / coefficient
```

```
anova(bond_mult_distancing_B100)
```

```
## Type III Analysis of Variance Table with Satterthwaite's method
##                         Sum Sq Mean Sq NumDF DenDF F value   Pr(>F)
## bond_mult               2.1875  1.0937     2  5495  1.1750 0.308891
## country_now_Name        5.3216  0.5322    10     2  0.5717 0.776846
## age                     0.0855  0.0855     1  5495  0.0919 0.761842
## gender                  6.4496  2.1499     3  5495  2.3096 0.074323 .
## education               8.6864  4.3432     2  5495  4.6660 0.009447 **
## bond_mult:country_now_Name 22.2659  1.1133    20  5495  1.1960 0.246454
## GDP
## ---
## Signif. codes:  0 '***' 0.001 '**' 0.01 '*' 0.05 '.' 0.1 ' ' 1
```

Hypothesis 2a, Dataset B

```
# Wellbeing
summary(bond_wellbeing_B100 <- lmer(wellbeing_scale ~ bond_country + bond_gvmt  +
                                       age + gender + education + GDP + country_now_Name +
                                     (1|country_now_Name), data=DatasetB_N100, na.action=na.exclude))
```

```
## Linear mixed model fit by REML. t-tests use Satterthwaite's method [
## lmerModLmerTest]
## Formula:
## wellbeing_scale ~ bond_country + bond_gvmt + age + gender + education +
##     GDP + country_now_Name + (1 | country_now_Name)
##    Data: DatasetB_N100
##
## REML criterion at convergence: 14320.3
##
## Scaled residuals:
##     Min      1Q  Median      3Q     Max
## -4.7163 -0.6736 -0.0611  0.6088  4.0130
##
## Random effects:
##  Groups           Name        Variance Std.Dev.
##  country_now_Name (Intercept) 0.2667   0.5165
##  Residual                     0.8445   0.9189
## Number of obs: 5334, groups:  country_now_Name, 11
##
## Fixed effects:
##                              Estimate Std. Error         df t value Pr(>|t|)
## (Intercept)                 4.186e-02  3.741e+00  5.315e+03   0.011 0.991074
## bond_country1               2.648e-01  3.505e-02  5.315e+03   7.554 4.94e-14
## bond_gvmt1                  2.086e-01  5.848e-02  5.315e+03   3.567 0.000364
## age                         1.479e-02  9.739e-04  5.315e+03  15.185  < 2e-16
## genderwoman                -1.562e-01  2.870e-02  5.315e+03  -5.442 5.50e-08
## gendernb                   -2.359e-01  1.291e-01  5.315e+03  -1.827 0.067737
## gendernone                 -3.615e-01  1.492e-01  5.315e+03  -2.423 0.015430
## educationundergraduate      7.056e-02  3.662e-02  5.315e+03   1.927 0.054035
## educationpostgraduate       1.570e-01  3.988e-02  5.315e+03   3.938 8.33e-05
## GDP                        -1.468e-05  6.484e-05  5.315e+03  -0.226 0.820853
## country_now_NameBangladesh -1.663e-01  3.631e+00  5.315e+03  -0.046 0.963478
## country_now_NameCanada     -4.432e-01  1.119e+00  5.315e+03  -0.396 0.692038
## country_now_NameFrance     -1.174e-01  1.350e+00  5.315e+03  -0.087 0.930685
## country_now_NameGermany     7.039e-02  9.638e-01  5.315e+03   0.073 0.941787
## country_now_NameItaly      -3.724e-01  1.777e+00  5.315e+03  -0.210 0.834020
## country_now_NamePeru       -1.798e-02  3.386e+00  5.315e+03  -0.005 0.995764
## country_now_NameSweden     -9.770e-04  7.196e-01  5.315e+03  -0.001 0.998917
## country_now_NameTurkey     -5.726e-01  3.232e+00  5.315e+03  -0.177 0.859381
## country_now_NameUK         -1.884e-01  1.233e+00  5.315e+03  -0.153 0.878548
##
## (Intercept)
## bond_country1              ***
## bond_gvmt1                 ***
## age                        ***
## genderwoman                ***
## gendernb                   .
## gendernone                 *
## educationundergraduate     .
## educationpostgraduate      ***
## GDP
## country_now_NameBangladesh
## country_now_NameCanada
## country_now_NameFrance
```

```
## country_now_NameGermany
## country_now_NameItaly
## country_now_NamePeru
## country_now_NameSweden
## country_now_NameTurkey
## country_now_NameUK
## ---
## Signif. codes:  0 '***' 0.001 '**' 0.01 '*' 0.05 '.' 0.1 ' ' 1
## fit warnings:
## fixed-effect model matrix is rank deficient so dropping 1 column / coefficient
## Some predictor variables are on very different scales: consider rescaling
## optimizer (nloptwrap) convergence code: 0 (OK)
## unable to evaluate scaled gradient
## Model failed to converge: degenerate  Hessian with 1 negative eigenvalues
```

```
anova(bond_wellbeing_B100)
```

```
## Type III Analysis of Variance Table with Satterthwaite's method
##                   Sum Sq Mean Sq NumDF DenDF  F value    Pr(>F)
## bond_country      48.188  48.188     1  5315  57.0640 4.938e-14 ***
## bond_gvmt         10.745  10.745     1  5315  12.7241 0.0003642 ***
## age              194.721 194.721     1  5315 230.5886 < 2.2e-16 ***
## gender            28.249   9.416     3  5315  11.1507 2.717e-07 ***
## education         14.306   7.153     2  5315   8.4707 0.0002124 ***
## GDP                0.043   0.043     1  5315   0.0513 0.8208532
## country_now_Name   1.160   0.129     9  5315   0.1526 0.9979789
## ---
## Signif. codes:  0 '***' 0.001 '**' 0.01 '*' 0.05 '.' 0.1 ' ' 1
```

Hypothesis 2b, Dataset B

```
# Hypothesis 2b
summary(bond_mult_wellbeing_B100 <- lmer(wellbeing_scale ~ bond_mult*country_now_Name  +
                                       age + gender + education + GDP +
                                    (1|country_now_Name), data=DatasetB_N100, na.action=na.exclude))
```

```
## Linear mixed model fit by REML. t-tests use Satterthwaite's method [
## lmerModLmerTest]
## Formula: wellbeing_scale ~ bond_mult * country_now_Name + age + gender +
##     education + GDP + (1 | country_now_Name)
##    Data: DatasetB_N100
##
## REML criterion at convergence: 14822
##
## Scaled residuals:
##     Min      1Q  Median      3Q     Max
## -4.7942 -0.6684 -0.0567  0.6016  4.0338
##
## Random effects:
##  Groups           Name        Variance Std.Dev.
##  country_now_Name (Intercept) 0.0438   0.2093
```

```
##  Residual                      0.8416   0.9174
## Number of obs: 5534, groups:  country_now_Name, 11
##
## Fixed effects:
##                                        Estimate Std. Error         df t value
## (Intercept)                          -7.356e-01  2.349e-01  1.400e-07  -3.132
## bond_mult1                            2.236e-01  1.844e-01  5.495e+03   1.213
## bond_mult2                            8.399e-01  3.386e-01  5.495e+03   2.481
## country_now_NameBangladesh            5.193e-01  3.199e-01  1.205e-07   1.623
## country_now_NameCanada               -2.581e-01  3.292e-01  1.351e-07  -0.784
## country_now_NameFrance                1.315e-01  3.160e-01  1.147e-07   0.416
## country_now_NameGermany               1.558e-01  3.180e-01  1.176e-07   0.490
## country_now_NameItaly                 2.127e-02  3.266e-01  1.309e-07   0.065
## country_now_NamePeru                  7.445e-01  3.149e-01  1.131e-07   2.364
## country_now_NameSweden               -9.848e-03  3.238e-01  1.264e-07  -0.030
## country_now_NameTurkey                8.419e-02  3.124e-01  1.096e-07   0.269
## country_now_NameUK                   -4.131e-02  3.118e-01  1.087e-07  -0.132
## country_now_NameUSA                  -1.701e-01  3.137e-01  1.114e-07  -0.542
## age                                   1.461e-02  9.635e-04  5.495e+03  15.164
## genderwoman                          -1.469e-01  2.808e-02  5.495e+03  -5.233
## gendernb                             -2.629e-01  1.266e-01  5.495e+03  -2.077
## gendernone                           -3.222e-01  1.473e-01  5.495e+03  -2.187
## educationundergraduate                7.036e-02  3.579e-02  5.495e+03   1.966
## educationpostgraduate                 1.600e-01  3.924e-02  5.495e+03   4.077
## bond_mult1:country_now_NameBangladesh  3.057e-01  2.227e-01  5.495e+03   1.373
## bond_mult2:country_now_NameBangladesh -3.234e-01  4.117e-01  5.495e+03  -0.785
## bond_mult1:country_now_NameCanada    -2.323e-01  2.927e-01  5.495e+03  -0.794
## bond_mult2:country_now_NameCanada    -6.746e-01  4.690e-01  5.495e+03  -1.438
## bond_mult1:country_now_NameFrance    -1.641e-01  2.216e-01  5.495e+03  -0.741
## bond_mult2:country_now_NameFrance    -4.603e-01  4.493e-01  5.495e+03  -1.025
## bond_mult1:country_now_NameGermany    9.180e-02  2.801e-01  5.495e+03   0.328
## bond_mult2:country_now_NameGermany   -2.773e-01  4.891e-01  5.495e+03  -0.567
## bond_mult1:country_now_NameItaly     -5.171e-01  3.107e-01  5.495e+03  -1.664
## bond_mult2:country_now_NameItaly     -4.220e-01  4.669e-01  5.495e+03  -0.904
## bond_mult1:country_now_NamePeru      -1.344e-01  2.014e-01  5.495e+03  -0.668
## bond_mult2:country_now_NamePeru      -5.788e-01  3.561e-01  5.495e+03  -1.625
## bond_mult1:country_now_NameSweden     6.580e-02  2.834e-01  5.495e+03   0.232
## bond_mult2:country_now_NameSweden    -2.023e-01  4.391e-01  5.495e+03  -0.461
## bond_mult1:country_now_NameTurkey     1.239e-01  2.042e-01  5.495e+03   0.607
## bond_mult2:country_now_NameTurkey    -5.757e-01  4.306e-01  5.495e+03  -1.337
## bond_mult1:country_now_NameUK         1.474e-01  1.923e-01  5.495e+03   0.766
## bond_mult2:country_now_NameUK        -2.812e-01  3.531e-01  5.495e+03  -0.796
## bond_mult1:country_now_NameUSA        1.696e-01  2.258e-01  5.495e+03   0.751
## bond_mult2:country_now_NameUSA        3.401e-01  5.066e-01  5.495e+03   0.671
##                                      Pr(>|t|)
## (Intercept)                            1.0000
## bond_mult1                             0.2252
## bond_mult2                             0.0131 *
## country_now_NameBangladesh             1.0000
## country_now_NameCanada                 1.0000
## country_now_NameFrance                 1.0000
## country_now_NameGermany                1.0000
## country_now_NameItaly                  1.0000
## country_now_NamePeru                   1.0000
```

```
## country_now_NameSweden                  1.0000
## country_now_NameTurkey                  1.0000
## country_now_NameUK                      1.0000
## country_now_NameUSA                     1.0000
## age                                   < 2e-16 ***
## genderwoman                          1.73e-07 ***
## gendernb                                0.0378 *
## gendernone                              0.0288 *
## educationundergraduate                  0.0493 *
## educationpostgraduate                4.62e-05 ***
## bond_mult1:country_now_NameBangladesh   0.1698
## bond_mult2:country_now_NameBangladesh   0.4322
## bond_mult1:country_now_NameCanada       0.4275
## bond_mult2:country_now_NameCanada       0.1504
## bond_mult1:country_now_NameFrance       0.4589
## bond_mult2:country_now_NameFrance       0.3056
## bond_mult1:country_now_NameGermany      0.7431
## bond_mult2:country_now_NameGermany      0.5708
## bond_mult1:country_now_NameItaly        0.0961 .
## bond_mult2:country_now_NameItaly        0.3662
## bond_mult1:country_now_NamePeru         0.5044
## bond_mult2:country_now_NamePeru         0.1041
## bond_mult1:country_now_NameSweden       0.8164
## bond_mult2:country_now_NameSweden       0.6450
## bond_mult1:country_now_NameTurkey       0.5442
## bond_mult2:country_now_NameTurkey       0.1813
## bond_mult1:country_now_NameUK           0.4434
## bond_mult2:country_now_NameUK           0.4258
## bond_mult1:country_now_NameUSA          0.4527
## bond_mult2:country_now_NameUSA          0.5020
## ---
## Signif. codes:  0 '***' 0.001 '**' 0.01 '*' 0.05 '.' 0.1 ' ' 1
## fit warnings:
## fixed-effect model matrix is rank deficient so dropping 1 column / coefficient
```

```
anova(bond_mult_wellbeing_B100)
```

```
## Type III Analysis of Variance Table with Satterthwaite's method
##                             Sum Sq Mean Sq NumDF DenDF  F value    Pr(>F)
## bond_mult                   40.978  20.489     2  5495  24.3441 2.979e-11 ***
## country_now_Name            14.981   1.498    10     2   1.7799 0.4128252
## age                        193.540 193.540     1  5495 229.9541 < 2.2e-16 ***
## gender                      26.247   8.749     3  5495  10.3951 8.099e-07 ***
## education                   15.313   7.657     2  5495   9.0971 0.0001137 ***
## bond_mult:country_now_Name  27.155   1.358    20  5495   1.6132 0.0409907 *
## GDP
## ---
## Signif. codes:  0 '***' 0.001 '**' 0.01 '*' 0.05 '.' 0.1 ' ' 1
```

Table S12A: Only country bonding as fixed-effect, Dataset A

Given relatively higher zero-order correlations of other bonding variables with country bonding and some discrepancies between our findings and prior literature *(28)*, we re-ran the analyses using only country

bonding as the fixed-effects variable. All findings held the same as is reported in the paper for both datasets and hypotheses.

Hypothesis 1a, Dataset A

```r
# Distancing
summary(bond_country_distancing_A <- lme(Pro_Distancing_scaled ~ bond_country  +
                                 demo_age + demo_gender + demo_education + GDP,
                                 (~1|Country_Name), data=DatasetA, na.action=na.exclude))
```

```
## Linear mixed-effects model fit by REML
##   Data: DatasetA
##        AIC      BIC    logLik
##   18379.04 18453.66 -9178.518
##
## Random effects:
##  Formula: ~1 | Country_Name
##         (Intercept)  Residual
## StdDev:   0.8309995 0.9708505
##
## Fixed effects:  Pro_Distancing_scaled ~ bond_country + demo_age + demo_gender +      demo_education +
##                                  Value  Std.Error   DF   t-value p-value
## (Intercept)                 -0.5369015 0.17652047 6460 -3.041582  0.0024
## bond_country1                0.0395864 0.04058753 6460  0.975334  0.3294
## demo_age                     0.0014221 0.00097011 6460  1.465907  0.1427
## demo_genderFemale            0.1587454 0.02519757 6460  6.300026  0.0000
## demo_genderOther/non-binary  0.0355921 0.12907799 6460  0.275741  0.7828
## demo_educationundergraduate  0.1152725 0.03057386 6460  3.770297  0.0002
## demo_educationpostgraduate   0.0913990 0.03327672 6460  2.746636  0.0060
## demo_educationNA            -0.1210031 0.14396798 6460 -0.840486  0.4007
## GDP                         -0.0000009 0.00000404   69 -0.223979  0.8234
##  Correlation:
##                             (Intr) bnd_c1 demo_g dm_gnF dm_O/- dm_dctnn
## bond_country1               -0.017
## demo_age                    -0.181 -0.106
## demo_genderFemale           -0.070 -0.003 -0.037
## demo_genderOther/non-binary -0.011 -0.001  0.035  0.113
## demo_educationundergraduate -0.086  0.010 -0.071 -0.017  0.017
## demo_educationpostgraduate  -0.071  0.022 -0.165 -0.039  0.008  0.580
## demo_educationNA            -0.037 -0.007 -0.024 -0.017  0.005  0.121
## GDP                         -0.698  0.007  0.002 -0.001 -0.031 -0.010
##                             dm_dctnp dm_dNA
## bond_country1
## demo_age
## demo_genderFemale
## demo_genderOther/non-binary
## demo_educationundergraduate
## demo_educationpostgraduate
## demo_educationNA             0.118
## GDP                         -0.008    0.012
##
## Standardized Within-Group Residuals:
```

```
##        Min         Q1        Med         Q3        Max
## -6.7467181 -0.1882206  0.3316969  0.5670914  2.4860840
##
## Number of Observations: 6538
## Number of Groups: 71
```

```
anova.lme(bond_country_distancing_A,type="marginal")
```

```
##                 numDF denDF   F-value p-value
## (Intercept)         1  6460  9.251223  0.0024
## bond_country        1  6460  0.951277  0.3294
## demo_age            1  6460  2.148883  0.1427
## demo_gender         2  6460 19.941694  <.0001
## demo_education      3  6460  5.503103  0.0009
## GDP                 1    69  0.050167  0.8234
```

```
# Hygiene
summary(bond_country_hygiene_A <- lme(Pro_Hygiene_scaled ~ bond_country  +
                                 demo_age + demo_gender + demo_education + GDP,
                                 (~1|Country_Name), data=DatasetA, na.action=na.exclude))
```

```
## Linear mixed-effects model fit by REML
##   Data: DatasetA
##        AIC      BIC    logLik
##   18022.41 18097.01 -9000.205
##
## Random effects:
##  Formula: ~1 | Country_Name
##         (Intercept)  Residual
## StdDev:   0.2261751 0.9537496
##
## Fixed effects:  Pro_Hygiene_scaled ~ bond_country + demo_age + demo_gender +      demo_education + G
##                                 Value  Std.Error   DF   t-value p-value
## (Intercept)                -0.2771994 0.09613677 6444 -2.883386  0.0039
## bond_country1               0.0515396 0.03975562 6444  1.296410  0.1949
## demo_age                    0.0070775 0.00094984 6444  7.451220  0.0000
## demo_genderFemale           0.2854267 0.02469767 6444 11.556825  0.0000
## demo_genderOther/non-binary -0.0603974 0.12612393 6444 -0.478873  0.6320
## demo_educationundergraduate -0.0297110 0.03002202 6444 -0.989640  0.3224
## demo_educationpostgraduate  -0.1124579 0.03263240 6444 -3.446205  0.0006
## demo_educationNA            0.3807077 0.14027149 6444  2.714078  0.0067
## GDP                        -0.0000054 0.00000202   70 -2.662136  0.0096
##  Correlation:
##                             (Intr) bnd_c1 demo_g dm_gnF dm_O/- dm_dctnn
## bond_country1               -0.028
## demo_age                    -0.331 -0.108
## demo_genderFemale           -0.119 -0.003 -0.037
## demo_genderOther/non-binary -0.027  0.000  0.036  0.113
## demo_educationundergraduate -0.161  0.009 -0.072 -0.020  0.016
## demo_educationpostgraduate  -0.126  0.022 -0.164 -0.041  0.007  0.582
## demo_educationNA            -0.052 -0.006 -0.024 -0.017  0.006  0.122
## GDP                         -0.746  0.013  0.001 -0.010 -0.034 -0.013
##                             dm_dctnp dm_dNA
```

```
## bond_country1
## demo_age
## demo_genderFemale
## demo_genderOther/non-binary
## demo_educationundergraduate
## demo_educationpostgraduate
## demo_educationNA             0.119
## GDP                         -0.011    0.016
##
## Standardized Within-Group Residuals:
##         Min          Q1         Med          Q3         Max
## -3.65250013 -0.62278286  0.05753114  0.71131672  2.53031040
##
## Number of Observations: 6523
## Number of Groups: 72
```

```
anova.lme(bond_country_hygiene_A,type="marginal")
```

```
##                 numDF denDF  F-value p-value
## (Intercept)         1  6444  8.31392  0.0039
## bond_country        1  6444  1.68068  0.1949
## demo_age            1  6444 55.52068  <.0001
## demo_gender         2  6444 68.39055  <.0001
## demo_education      3  6444  7.62609  <.0001
## GDP                 1    70  7.08697  0.0096
```

```
# Masking
summary(bond_country_masking_A <- lme(Pro_MaskWearing_scaled ~ bond_country  +
                                 demo_age + demo_gender + demo_education + GDP,
                                 (~1|Country_Name), data=DatasetA, na.action=na.exclude))
```

```
## Linear mixed-effects model fit by REML
##   Data: DatasetA
##        AIC      BIC    logLik
##   17776.88 17851.49 -8877.441
##
## Random effects:
##  Formula: ~1 | Country_Name
##         (Intercept)  Residual
## StdDev:   0.4322335 0.9322965
##
## Fixed effects:  Pro_MaskWearing_scaled ~ bond_country + demo_age + demo_gender +      demo_education
##                                  Value  Std.Error   DF   t-value p-value
## (Intercept)                  0.1155505 0.12265365 6451  0.942088  0.3462
## bond_country1                0.0347292 0.03897616 6451  0.891037  0.3729
## demo_age                     0.0031257 0.00093172 6451  3.354754  0.0008
## demo_genderFemale            0.0482123 0.02418042 6451  1.993857  0.0462
## demo_genderOther/non-binary  0.0031475 0.12466823 6451  0.025247  0.9799
## demo_educationundergraduate  0.0132297 0.02935925 6451  0.450615  0.6523
## demo_educationpostgraduate  -0.0601411 0.03196168 6451 -1.881663  0.0599
## demo_educationNA             0.3353972 0.13761214 6451  2.437264  0.0148
## GDP                         -0.0000056 0.00000268   69 -2.084754  0.0408
##  Correlation:
```

```
##                               (Intr) bnd_c1 demo_g dm_gnF dm_O/- dm_dctnn
## bond_country1                 -0.023
## demo_age                      -0.249 -0.107
## demo_genderFemale             -0.097 -0.004 -0.038
## demo_genderOther/non-binary   -0.018  0.000  0.034  0.112
## demo_educationundergraduate   -0.123  0.010 -0.072 -0.017  0.018
## demo_educationpostgraduate    -0.101  0.022 -0.166 -0.040  0.008  0.582
## demo_educationNA              -0.048 -0.007 -0.024 -0.017  0.006  0.122
## GDP                           -0.725  0.010  0.001 -0.002 -0.036 -0.011
##                               dm_dctnp dm_dNA
## bond_country1
## demo_age
## demo_genderFemale
## demo_genderOther/non-binary
## demo_educationundergraduate
## demo_educationpostgraduate
## demo_educationNA               0.119
## GDP                           -0.009    0.016
##
## Standardized Within-Group Residuals:
##        Min         Q1        Med         Q3        Max
## -1.8989709 -0.6693565 -0.2813121  0.5946783  3.3631700
##
## Number of Observations: 6529
## Number of Groups: 71
```

```
anova.lme(bond_country_masking_A,type="marginal")
```

```
##                  numDF denDF   F-value p-value
## (Intercept)          1  6451  0.887530  0.3462
## bond_country         1  6451  0.793948  0.3729
## demo_age             1  6451 11.254374  0.0008
## demo_gender          2  6451  2.007533  0.1344
## demo_education       3  6451  4.569990  0.0033
## GDP                  1    69  4.346199  0.0408
```

Hypothesis 2a, Dataset A

```
# Anxiety
summary(bond_country_anx_A <- lme(anx_sum1_scaled ~ bond_country  +
                                 demo_age + demo_gender + demo_education + GDP,
                                 (~1|Country_Name), data=DatasetA, na.action=na.exclude))
```

```
## Linear mixed-effects model fit by REML
##   Data: DatasetA
##        AIC      BIC    logLik
##   17733.92 17808.56 -8855.959
##
## Random effects:
##  Formula: ~1 | Country_Name
##         (Intercept)  Residual
```

```
## StdDev:   0.2271704 0.9275825
##
## Fixed effects:  anx_sum1_scaled ~ bond_country + demo_age + demo_gender + demo_education +      GDP
##                                   Value  Std.Error   DF    t-value p-value
## (Intercept)                  -0.0040386 0.09453911 6471  -0.042718  0.9659
## bond_country1                -0.2279234 0.03855735 6471  -5.911283  0.0000
## demo_age                     -0.0094107 0.00092107 6471 -10.217176  0.0000
## demo_genderFemale             0.4502097 0.02397968 6471  18.774635  0.0000
## demo_genderOther/non-binary   0.5395497 0.12267158 6471   4.398327  0.0000
## demo_educationundergraduate -0.0259696 0.02911372 6471  -0.892004  0.3724
## demo_educationpostgraduate  -0.0267371 0.03166925 6471  -0.844260  0.3986
## demo_educationNA              0.1306313 0.13641750 6471   0.957584  0.3383
## GDP                          -0.0000012 0.00000199   70  -0.620954  0.5366
##  Correlation:
##                              (Intr) bnd_c1 demo_g dm_gnF dm_O/- dm_dctnn
## bond_country1                -0.027
## demo_age                     -0.326 -0.109
## demo_genderFemale            -0.118 -0.003 -0.039
## demo_genderOther/non-binary -0.026  0.000  0.036  0.113
## demo_educationundergraduate -0.160  0.010 -0.071 -0.018  0.016
## demo_educationpostgraduate  -0.124  0.022 -0.164 -0.040  0.007  0.581
## demo_educationNA             -0.052 -0.006 -0.023 -0.017  0.006  0.122
## GDP                          -0.746  0.013  0.001 -0.010 -0.034 -0.013
##                              dm_dctnp dm_dNA
## bond_country1
## demo_age
## demo_genderFemale
## demo_genderOther/non-binary
## demo_educationundergraduate
## demo_educationpostgraduate
## demo_educationNA              0.119
## GDP                          -0.011    0.017
##
## Standardized Within-Group Residuals:
##         Min          Q1         Med          Q3         Max
## -2.66881598 -0.74528929 -0.07189847  0.66149022  3.61511865
##
## Number of Observations: 6550
## Number of Groups: 72
```

```r
anova.lme(bond_country_anx_A,type="marginal")
```

```
##                 numDF denDF   F-value p-value
## (Intercept)         1  6471   0.00182  0.9659
## bond_country        1  6471  34.94326  <.0001
## demo_age            1  6471 104.39068  <.0001
## demo_gender         2  6471 178.88475  <.0001
## demo_education      3  6471   0.72192  0.5388
## GDP                 1    70   0.38558  0.5366
```

```r
# Depression
summary(bond_country_dep_A <- lme(dep_sum1_scaled ~ bond_country  +
                                 demo_age + demo_gender + demo_education + GDP,
                                 (~1|Country_Name), data=DatasetA, na.action=na.exclude))
```

```
## Linear mixed-effects model fit by REML
##   Data: DatasetA
##        AIC      BIC    logLik
##   18234.32 18308.97 -9106.161
##
## Random effects:
##  Formula: ~1 | Country_Name
##         (Intercept)  Residual
## StdDev:   0.1937813 0.9641066
##
## Fixed effects:  dep_sum1_scaled ~ bond_country + demo_age + demo_gender + demo_education +      GDP
##                                 Value  Std.Error   DF   t-value p-value
## (Intercept)                 0.2387185 0.09162172 6472  2.605479  0.0092
## bond_country1              -0.2599206 0.04004964 6472 -6.489962  0.0000
## demo_age                   -0.0070607 0.00095635 6472 -7.382994  0.0000
## demo_genderFemale           0.1971057 0.02490666 6472  7.913778  0.0000
## demo_genderOther/non-binary  0.5300862 0.12742591 6472  4.159957  0.0000
## demo_educationundergraduate -0.1106299 0.03024931 6472 -3.657270  0.0003
## demo_educationpostgraduate  -0.1297285 0.03289693 6472 -3.943483  0.0001
## demo_educationNA           -0.0340476 0.14168856 6472 -0.240299  0.8101
## GDP                        -0.0000028 0.00000190   70 -1.467472  0.1467
##  Correlation:
##                             (Intr) bnd_c1 demo_g dm_gnF dm_O/- dm_dctnn
## bond_country1               -0.029
## demo_age                    -0.349 -0.110
## demo_genderFemale           -0.125 -0.003 -0.039
## demo_genderOther/non-binary -0.029  0.000  0.036  0.113
## demo_educationundergraduate -0.172  0.010 -0.071 -0.017  0.016
## demo_educationpostgraduate  -0.132  0.022 -0.164 -0.039  0.007  0.582
## demo_educationNA            -0.052 -0.006 -0.023 -0.017  0.006  0.122
## GDP                         -0.748  0.015 -0.001 -0.013 -0.033 -0.013
##                             dm_dctnp dm_dNA
## bond_country1
## demo_age
## demo_genderFemale
## demo_genderOther/non-binary
## demo_educationundergraduate
## demo_educationpostgraduate
## demo_educationNA             0.119
## GDP                         -0.011    0.016
##
## Standardized Within-Group Residuals:
##         Min          Q1         Med          Q3         Max
## -2.46430100 -0.74972701 -0.08799193  0.66410428  4.05138301
##
## Number of Observations: 6551
## Number of Groups: 72
```

```
anova.lme(bond_country_dep_A,type="marginal")
```

```
##                 numDF denDF  F-value p-value
```

```
## (Intercept)        1  6472  6.78852  0.0092
## bond_country       1  6472 42.11961  <.0001
## demo_age           1  6472 54.50860  <.0001
## demo_gender        2  6472 36.72763  <.0001
## demo_education     3  6472  6.15893  0.0004
## GDP                1    70  2.15347  0.1467
```

Table S12B: Only country bonding as fixed-effect, Dataset B

Hypothesis 1a, Dataset B

```
# Distancing
summary(bond_country_distancing_B <- lme(comply_self_scale ~ bond_country  +
                                 age + gender + education + GDP,
                                 (~1|country_now_Name), data=DatasetB_phase1, na.action=na.exclude))
```

```
## Linear mixed-effects model fit by REML
##   Data: DatasetB_phase1
##        AIC      BIC    logLik
##   18011.05 18085.52 -8994.523
##
## Random effects:
##  Formula: ~1 | country_now_Name
##         (Intercept)  Residual
## StdDev:   0.1604893 0.9682376
##
## Fixed effects:  comply_self_scale ~ bond_country + age + gender + education +      GDP
##                              Value  Std.Error   DF   t-value p-value
## (Intercept)            -0.23910813 0.06163812 6334 -3.879225  0.0001
## bond_country1           0.00168619 0.03074950 6334  0.054836  0.9563
## age                     0.00075190 0.00094909 6334  0.792230  0.4283
## genderwoman             0.06380026 0.02704442 6334  2.359092  0.0183
## gendernb                0.01016651 0.12865702 6334  0.079020  0.9370
## gendernone              0.25897662 0.14792918 6334  1.750680  0.0800
## educationundergraduate  0.06580495 0.03591902 6334  1.832036  0.0670
## educationpostgraduate   0.11579127 0.03866268 6334  2.994911  0.0028
## GDP                     0.00000379 0.00000128  106  2.962305  0.0038
##  Correlation:
##                        (Intr) bnd_c1 age    gndrwm gndrnb gndrnn edctnn edctnp
## bond_country1          -0.055
## age                    -0.376 -0.210
## genderwoman            -0.259 -0.025  0.082
## gendernb               -0.078  0.017  0.055  0.151
## gendernone             -0.068  0.012  0.029  0.124  0.029
## educationundergraduate -0.356  0.015 -0.182 -0.030 -0.002  0.004
## educationpostgraduate  -0.310  0.057 -0.254 -0.046 -0.002  0.007  0.722
## GDP                    -0.497  0.059 -0.085 -0.078 -0.023 -0.004  0.020 -0.009
##
## Standardized Within-Group Residuals:
##        Min         Q1        Med         Q3        Max
## -3.1496529 -0.6816534 -0.3056902  0.7738097  2.1241684
##
```

```
## Number of Observations: 6449
## Number of Groups: 108
```

```r
anova.lme(bond_country_distancing_B,type="marginal")
```

```
##               numDF denDF   F-value p-value
## (Intercept)       1  6334 15.048387  0.0001
## bond_country      1  6334  0.003007  0.9563
## age               1  6334  0.627629  0.4283
## gender            3  6334  2.605210  0.0501
## education         2  6334  4.598097  0.0101
## GDP               1   106  8.775250  0.0038
```

Hypothesis 2a, Dataset B

```r
# Wellbeing
summary(bond_country_wellbeing_B <- lme(wellbeing_scale ~ bond_country  +
                                 age + gender + education + GDP,
                                 (~1|country_now_Name), data=DatasetB_phase1, na.action=na.exclude))
```

```
## Linear mixed-effects model fit by REML
##   Data: DatasetB_phase1
##        AIC      BIC    logLik
##   17435.11 17509.58 -8706.554
##
## Random effects:
##  Formula: ~1 | country_now_Name
##         (Intercept)  Residual
## StdDev:   0.2008581 0.9248021
##
## Fixed effects:  wellbeing_scale ~ bond_country + age + gender + education + GDP
##                             Value  Std.Error   DF   t-value p-value
## (Intercept)            -0.5398797 0.06347937 6334 -8.504805  0.0000
## bond_country1           0.3355238 0.02943178 6334 11.400050  0.0000
## age                     0.0144336 0.00090843 6334 15.888513  0.0000
## genderwoman            -0.1604394 0.02586802 6334 -6.202230  0.0000
## gendernb               -0.3081276 0.12295203 6334 -2.506080  0.0122
## gendernone             -0.3210245 0.14136175 6334 -2.270943  0.0232
## educationundergraduate  0.0692556 0.03433385 6334  2.017125  0.0437
## educationpostgraduate   0.1448172 0.03700030 6334  3.913947  0.0001
## GDP                    -0.0000022 0.00000138  106 -1.591340  0.1145
##  Correlation:
##                        (Intr) bnd_c1 age    gndrwm gndrnb gndrnn edctnn edctnp
## bond_country1          -0.049
## age                    -0.351 -0.211
## genderwoman            -0.242 -0.025  0.083
## gendernb               -0.073  0.017  0.055  0.151
## gendernone             -0.064  0.012  0.029  0.124  0.029
## educationundergraduate -0.331  0.015 -0.182 -0.030 -0.002  0.004
## educationpostgraduate  -0.292  0.057 -0.253 -0.046 -0.002  0.007  0.721
## GDP                    -0.523  0.050 -0.073 -0.067 -0.018 -0.001  0.016 -0.008
```

```
##
## Standardized Within-Group Residuals:
##         Min          Q1         Med          Q3         Max
## -4.65833205 -0.67413303 -0.05547987  0.60145236  3.97206560
##
## Number of Observations: 6449
## Number of Groups: 108
```

```
anova.lme(bond_country_wellbeing_B,type="marginal")
```

```
##                numDF denDF   F-value p-value
## (Intercept)        1  6334  72.33171  <.0001
## bond_country       1  6334 129.96114  <.0001
## age                1  6334 252.44486  <.0001
## gender             3  6334  14.41092  <.0001
## education          2  6334   8.33761  0.0002
## GDP                1   106   2.53236  0.1145
```

Table S13A: Longitudinal comparisons, Dataset A

Dataset A had 2 time-points, with the bonding, distancing and mental health questions repeated at both time-points. Using these data, we examined whether the findings held at T2 and whether bonding at T1 predicted the variables of interest at T2. Dataset A, T2 dates: 2nd August June 2020 - 11th September 2020

Hypothesis 1a, Dataset A

```
# Does bonding at T1 predict distancing at T2?
summary(bond_distancing_A_long1<- lme(Pro_Distancing2_scaled ~ bond_family + bond_friend +
                                        bond_country + bond_humanity +
                                 demo_age + demo_gender + demo_education + GDP,
                                 (~1|Country_Name), data=DatasetA, na.action=na.exclude))
```

```
## Linear mixed-effects model fit by REML
##   Data: DatasetA
##        AIC      BIC    logLik
##   1922.561 1986.997 -947.2803
##
## Random effects:
##  Formula: ~1 | Country_Name
##         (Intercept)  Residual
## StdDev:   0.8952929 0.7960363
##
## Fixed effects:  Pro_Distancing2_scaled ~ bond_family + bond_friend + bond_country +      bond_humani
##                              Value Std.Error  DF    t-value p-value
## (Intercept)             -0.6763822 0.3474212 709 -1.9468649  0.0519
## bond_family1             0.0658222 0.0745750 709  0.8826307  0.3777
## bond_friend1            -0.1204168 0.1141244 709 -1.0551366  0.2917
## bond_country1           -0.0204047 0.1120957 709 -0.1820296  0.8556
## bond_humanity1           0.0452078 0.0787354 709  0.5741737  0.5660
## demo_age                 0.0073892 0.0026313 709  2.8081938  0.0051
```

```
## demo_genderFemale            0.1648808 0.0630280 709  2.6159911  0.0091
## demo_genderOther/non-binary  0.0431230 0.2392433 709  0.1802476  0.8570
## demo_educationundergraduate  0.0792463 0.0825616 709  0.9598444  0.3375
## demo_educationpostgraduate   0.0632092 0.0860426 709  0.7346263  0.4628
## demo_educationNA            -0.3469359 1.2329182  28 -0.2813940  0.7805
## GDP                         -0.0000068 0.0000081  28 -0.8440233  0.4058
##  Correlation:
##                             (Intr) bnd_fm1 bnd_fr1 bnd_c1 bnd_h1 demo_g dm_gnF
## bond_family1                -0.046
## bond_friend1                -0.003 -0.130
## bond_country1               -0.015 -0.087  -0.136
## bond_humanity1              -0.053 -0.065  -0.017  -0.268
## demo_age                    -0.254 -0.104  -0.001   0.047  0.008
## demo_genderFemale           -0.040 -0.079  -0.071   0.006  0.019 -0.046
## demo_genderOther/non-binary -0.032  0.067  -0.063   0.012 -0.020  0.033  0.150
## demo_educationundergraduate -0.136  0.074  -0.014   0.035  0.014 -0.023 -0.052
## demo_educationpostgraduate  -0.118  0.044   0.023   0.059  0.006 -0.142 -0.071
## demo_educationNA            -0.226  0.021   0.004   0.002  0.013  0.017 -0.035
## GDP                         -0.776  0.034   0.003  -0.025  0.017 -0.004 -0.039
##                             dm_O/- dm_dctnn dm_dctnp dm_dNA
## bond_family1
## bond_friend1
## bond_country1
## bond_humanity1
## demo_age
## demo_genderFemale
## demo_genderOther/non-binary
## demo_educationundergraduate -0.005
## demo_educationpostgraduate   0.035  0.682
## demo_educationNA             0.000  0.043    0.046
## GDP                         -0.009 -0.019   -0.015    0.170
##
## Standardized Within-Group Residuals:
##        Min         Q1        Med         Q3        Max
## -4.1181177 -0.5569072  0.1824498  0.6396861  3.9324497
##
## Number of Observations: 749
## Number of Groups: 31
```

```
anova.lme(bond_distancing_A_long1,type="marginal") # n.s.
```

```
##                 numDF denDF  F-value p-value
## (Intercept)         1   708 3.790283  0.0519
## bond_family         1   708 0.779037  0.3777
## bond_friend         1   708 1.113313  0.2917
## bond_country        1   708 0.033135  0.8556
## bond_humanity       1   708 0.329675  0.5660
## demo_age            1   708 7.885952  0.0051
## demo_gender         2   708 3.444960  0.0324
## demo_education      3   708 0.346430  0.7918
## GDP                 1    29 0.712375  0.4056
```

```r
# Does bonding at T2 predict distancing at T2?
summary(bond_distancing_A_T2<- lme(Pro_Distancing2_scaled ~ bond_family2 + bond_friend2 +
                                        bond_country2 + bond_humanity2 +
                                 demo_age + demo_gender + demo_education + GDP,
                                 (~1|Country_Name), data=DatasetA, na.action=na.exclude))
```

```
## Linear mixed-effects model fit by REML
##   Data: DatasetA
##        AIC      BIC    logLik
##   1846.425 1910.437 -909.2123
##
## Random effects:
##  Formula: ~1 | Country_Name
##         (Intercept)  Residual
## StdDev:    0.914117 0.7835722
##
## Fixed effects:  Pro_Distancing2_scaled ~ bond_family2 + bond_friend2 + bond_country2 +      bond_huma
##                                  Value Std.Error  DF    t-value p-value
## (Intercept)                 -0.7199117 0.3629289 688 -1.9836165  0.0477
## bond_family2                 0.0864058 0.0759974 688  1.1369564  0.2560
## bond_friend2                -0.1684875 0.1423080 688 -1.1839633  0.2368
## bond_country2               -0.0738011 0.1244239 688 -0.5931423  0.5533
## bond_humanity2               0.0347784 0.0968215 688  0.3592010  0.7196
## demo_age                     0.0071025 0.0026360 688  2.6944126  0.0072
## demo_genderFemale            0.1299307 0.0627370 688  2.0710394  0.0387
## demo_genderOther/non-binary  0.2512214 0.2455289 688  1.0231847  0.3066
## demo_educationundergraduate  0.0921549 0.0823452 688  1.1191291  0.2635
## demo_educationpostgraduate   0.0437439 0.0858328 688  0.5096409  0.6105
## demo_educationNA            -0.2735757 1.2418949  27 -0.2202890  0.8273
## GDP                         -0.0000052 0.0000084  27 -0.6144677  0.5441
##  Correlation:
##                             (Intr) bnd_fm2 bnd_fr2 bnd_c2 bnd_h2 demo_g dm_gnF
## bond_family2                -0.022
## bond_friend2                -0.026 -0.208
## bond_country2                0.036 -0.050  -0.182
## bond_humanity2              -0.076  0.038  -0.068  -0.210
## demo_age                    -0.249 -0.093   0.026  -0.063  0.046
## demo_genderFemale           -0.031 -0.077  -0.026   0.019 -0.018 -0.044
## demo_genderOther/non-binary -0.030  0.038   0.012   0.049 -0.010  0.039  0.141
## demo_educationundergraduate -0.122  0.087  -0.024   0.007  0.029 -0.035 -0.052
## demo_educationpostgraduate  -0.096  0.001   0.019   0.000 -0.054 -0.152 -0.062
## demo_educationNA            -0.235  0.015   0.006  -0.006  0.019  0.018 -0.037
## GDP                         -0.789  0.008   0.019  -0.038  0.032  0.003 -0.044
##                             dm_O/- dm_dctnn dm_dctnp dm_dNA
## bond_family2
## bond_friend2
## bond_country2
## bond_humanity2
## demo_age
## demo_genderFemale
## demo_genderOther/non-binary
## demo_educationundergraduate  0.003
## demo_educationpostgraduate   0.037  0.676
```

```
## demo_educationNA             0.000  0.042    0.041
## GDP                         -0.012 -0.022   -0.022    0.180
##
## Standardized Within-Group Residuals:
##        Min         Q1        Med         Q3        Max
## -4.2139545 -0.5437865  0.1984536  0.6363779  4.0435088
##
## Number of Observations: 727
## Number of Groups: 30
```

```
anova.lme(bond_distancing_A_T2,type="marginal") # n.s.
```

```
##                numDF denDF  F-value p-value
## (Intercept)        1   687 3.934734  0.0477
## bond_family2       1   687 1.292670  0.2560
## bond_friend2       1   687 1.401769  0.2368
## bond_country2      1   687 0.351818  0.5533
## bond_humanity2     1   687 0.129025  0.7196
## demo_age           1   687 7.259859  0.0072
## demo_gender        2   687 2.417211  0.0899
## demo_education     3   687 0.477679  0.6979
## GDP                1    28 0.377571  0.5439
```

Hypothesis 1b, Dataset A

```
# Does bonding at T1 predict distancing at T2?
summary(bond_distancing_A_long1<- lme(Pro_Distancing2_scaled ~ bond_mult +
                                 demo_age + demo_gender + demo_education + GDP,
                                 (~1|Country_Name), data=DatasetA, na.action=na.exclude))
```

```
## Linear mixed-effects model fit by REML
##   Data: DatasetA
##        AIC      BIC    logLik
##   1920.786 1985.241 -946.3928
##
## Random effects:
##  Formula: ~1 | Country_Name
##         (Intercept)  Residual
## StdDev:   0.8830056 0.7961447
##
## Fixed effects:  Pro_Distancing2_scaled ~ bond_mult + demo_age + demo_gender +      demo_education + G
##                                  Value Std.Error  DF    t-value p-value
## (Intercept)                 -0.6332788 0.3451147 710 -1.8349807  0.0669
## bond_mult1                  -0.0570972 0.0682186 710 -0.8369735  0.4029
## bond_mult2                   0.0572001 0.1041869 710  0.5490146  0.5832
## bond_mult3                   0.0465512 0.1960838 710  0.2374046  0.8124
## bond_mult4                   0.1594221 0.3425334 710  0.4654206  0.6418
## demo_age                     0.0073480 0.0026474 710  2.7755808  0.0057
## demo_genderFemale            0.1586235 0.0627871 710  2.5263694  0.0117
## demo_genderOther/non-binary  0.0024136 0.2387617 710  0.0101090  0.9919
## demo_educationundergraduate  0.0823799 0.0827696 710  0.9952914  0.3199
```

```
## demo_educationpostgraduate   0.0672733 0.0867631 710  0.7753676  0.4384
## demo_educationNA            -0.3795797 1.2234873  28 -0.3102441  0.7587
## GDP                         -0.0000072 0.0000080  28 -0.9030193  0.3742
##  Correlation:
##                             (Intr) bnd_m1 bnd_m2 bnd_m3 bnd_m4 demo_g dm_gnF
## bond_mult1                  -0.102
## bond_mult2                  -0.050  0.210
## bond_mult3                  -0.026  0.114  0.070
## bond_mult4                  -0.007  0.061  0.031  0.045
## demo_age                    -0.262  0.047  0.033 -0.035 -0.140
## demo_genderFemale           -0.043 -0.001 -0.049 -0.079 -0.018 -0.050
## demo_genderOther/non-binary -0.034  0.048 -0.026  0.011  0.001  0.040  0.153
## demo_educationundergraduate -0.130 -0.025  0.033  0.101  0.044 -0.028 -0.055
## demo_educationpostgraduate  -0.114  0.008 -0.022  0.130  0.087 -0.155 -0.074
## demo_educationNA            -0.225  0.025  0.014  0.013  0.010  0.018 -0.035
## GDP                         -0.774  0.028  0.005  0.001  0.018 -0.001 -0.037
##                             dm_O/- dm_dctnn dm_dctnp dm_dNA
## bond_mult1
## bond_mult2
## bond_mult3
## bond_mult4
## demo_age
## demo_genderFemale
## demo_genderOther/non-binary
## demo_educationundergraduate -0.011
## demo_educationpostgraduate   0.036  0.684
## demo_educationNA             0.000  0.042    0.046
## GDP                         -0.009 -0.022   -0.015    0.170
##
## Standardized Within-Group Residuals:
##        Min         Q1        Med         Q3        Max
## -3.9906270 -0.5364152  0.1800464  0.6462276  4.0399872
##
## Number of Observations: 750
## Number of Groups: 31
```

```
anova.lme(bond_distancing_A_long1,type="marginal") # n.s.
```

```
##                 numDF denDF  F-value p-value
## (Intercept)         1   709 3.367154  0.0669
## bond_mult           4   709 0.395472  0.8120
## demo_age            1   709 7.703849  0.0057
## demo_gender         2   709 3.263597  0.0388
## demo_education      3   709 0.378012  0.7689
## GDP                 1    29 0.815444  0.3740
```

```
# Does bonding at T2 predict distancing at T2?
summary(bond_distancing_A_T2<- lme(Pro_Distancing2_scaled ~ bond_mult2  +
                                 demo_age + demo_gender + demo_education + GDP,
                                 (~1|Country_Name), data=DatasetA, na.action=na.exclude))
```

```
## Linear mixed-effects model fit by REML
##   Data: DatasetA
```

```
##      AIC      BIC    logLik
##   1842.3 1906.331 -907.1499
##
## Random effects:
##  Formula: ~1 | Country_Name
##         (Intercept)  Residual
## StdDev:   0.9185461 0.7820927
##
## Fixed effects:  Pro_Distancing2_scaled ~ bond_mult2 + demo_age + demo_gender +      demo_education +
##                                  Value Std.Error  DF    t-value p-value
## (Intercept)                 -0.7393341 0.3633685 689 -2.0346674  0.0423
## bond_mult21                  0.0941017 0.0691231 689  1.3613639  0.1738
## bond_mult22                 -0.1687984 0.1244664 689 -1.3561760  0.1755
## bond_mult23                  0.1977788 0.2425718 689  0.8153413  0.4152
## bond_mult24                 -0.3512032 0.4574183 689 -0.7677942  0.4429
## demo_age                     0.0075196 0.0026173 689  2.8731037  0.0042
## demo_genderFemale            0.1386854 0.0625007 689  2.2189426  0.0268
## demo_genderOther/non-binary  0.2680787 0.2449194 689  1.0945589  0.2741
## demo_educationundergraduate  0.0928362 0.0821934 689  1.1294847  0.2591
## demo_educationpostgraduate   0.0500800 0.0857369 689  0.5841130  0.5593
## demo_educationNA            -0.2719949 1.2443936  27 -0.2185762  0.8286
## GDP                         -0.0000054 0.0000084  27 -0.6465260  0.5234
##  Correlation:
##                             (Intr) bnd_21 bnd_22 bnd_23 bnd_24 demo_g dm_gnF
## bond_mult21                 -0.065
## bond_mult22                 -0.034  0.167
## bond_mult23                 -0.010  0.097  0.061
## bond_mult24                  0.004  0.041  0.031  0.008
## demo_age                    -0.244 -0.039 -0.035 -0.005 -0.066
## demo_genderFemale           -0.032 -0.027 -0.067 -0.037 -0.006 -0.049
## demo_genderOther/non-binary -0.033  0.052  0.034  0.027  0.005  0.044  0.141
## demo_educationundergraduate -0.123  0.081  0.004  0.033  0.037 -0.032 -0.047
## demo_educationpostgraduate  -0.099  0.018 -0.060 -0.011  0.037 -0.150 -0.057
## demo_educationNA            -0.235  0.022  0.014  0.005  0.003  0.017 -0.036
## GDP                         -0.788  0.010  0.015 -0.004  0.001  0.000 -0.043
##                             dm_O/- dm_dctnn dm_dctnp dm_dNA
## bond_mult21
## bond_mult22
## bond_mult23
## bond_mult24
## demo_age
## demo_genderFemale
## demo_genderOther/non-binary
## demo_educationundergraduate  0.004
## demo_educationpostgraduate   0.035  0.680
## demo_educationNA             0.001  0.041    0.041
## GDP                         -0.010 -0.022   -0.022    0.180
##
## Standardized Within-Group Residuals:
##        Min         Q1        Med         Q3        Max
## -4.2285209 -0.5579455  0.2034590  0.6371500  4.0986605
##
## Number of Observations: 728
## Number of Groups: 30
```

```r
anova.lme(bond_distancing_A_T2,type="marginal") # n.s.
```

```
##                 numDF denDF  F-value p-value
## (Intercept)         1   688 4.139871  0.0423
## bond_mult2          4   688 1.408281  0.2295
## demo_age            1   688 8.254725  0.0042
## demo_gender         2   688 2.773645  0.0631
## demo_education      3   688 0.468945  0.7040
## GDP                 1    28 0.417996  0.5232
```

Hypothesis 2a, Dataset A

```r
# Does bonding at T1 predict anxiety at T2?
summary(bond_anx_A_long1<- lme(anx_sum2_scaled ~ bond_family + bond_friend +
                                 bond_country + bond_humanity +
                                demo_age + demo_gender + demo_education + GDP,
                                (~1|Country_Name), data=DatasetA, na.action=na.exclude))
```

```
## Linear mixed-effects model fit by REML
##   Data: DatasetA
##        AIC      BIC    logLik
##   2124.851 2189.268 -1048.425
##
## Random effects:
##  Formula: ~1 | Country_Name
##         (Intercept)  Residual
## StdDev:   0.2562272 0.9423898
##
## Fixed effects:  anx_sum2_scaled ~ bond_family + bond_friend + bond_country +      bond_humanity + der
##                                  Value Std.Error  DF   t-value p-value
## (Intercept)                  0.1809688 0.2123795 708  0.852101  0.3944
## bond_family1                -0.0522511 0.0875453 708 -0.596847  0.5508
## bond_friend1                -0.1090806 0.1346390 708 -0.810171  0.4181
## bond_country1               -0.1888609 0.1315435 708 -1.435730  0.1515
## bond_humanity1              -0.1433129 0.0923696 708 -1.551516  0.1212
## demo_age                    -0.0086607 0.0030591 708 -2.831184  0.0048
## demo_genderFemale            0.4320662 0.0739797 708  5.840336  0.0000
## demo_genderOther/non-binary  0.3172759 0.2815844 708  1.126752  0.2602
## demo_educationundergraduate -0.0068942 0.0972022 708 -0.070926  0.9435
## demo_educationpostgraduate  -0.0489311 0.1009918 708 -0.484506  0.6282
## demo_educationNA            -0.9162591 0.9915370  28 -0.924080  0.3633
## GDP                         -0.0000057 0.0000041  28 -1.409234  0.1698
##  Correlation:
##                             (Intr) bnd_fm1 bnd_fr1 bnd_c1 bnd_h1 demo_g dm_gnF
## bond_family1                -0.055
## bond_friend1                -0.023 -0.130
## bond_country1               -0.053 -0.087  -0.141
## bond_humanity1              -0.085 -0.067  -0.015  -0.263
## demo_age                    -0.458 -0.111   0.000   0.039  0.013
## demo_genderFemale           -0.087 -0.080  -0.071   0.003  0.018 -0.053
## demo_genderOther/non-binary -0.065  0.063  -0.062   0.011 -0.020  0.034  0.147
```

```
## demo_educationundergraduate -0.288  0.073  -0.019   0.032  0.020 -0.025 -0.056
## demo_educationpostgraduate  -0.234  0.043   0.021   0.058  0.010 -0.139 -0.077
## demo_educationNA            -0.148  0.026   0.010   0.009  0.015  0.020 -0.049
## GDP                         -0.669  0.048   0.020  -0.035  0.018 -0.055 -0.075
##                             dm_O/- dm_dctnn dm_dctnp dm_dNA
## bond_family1
## bond_friend1
## bond_country1
## bond_humanity1
## demo_age
## demo_genderFemale
## demo_genderOther/non-binary
## demo_educationundergraduate -0.007
## demo_educationpostgraduate   0.033  0.684
## demo_educationNA             0.001  0.069    0.068
## GDP                         -0.015 -0.021   -0.012    0.122
##
## Standardized Within-Group Residuals:
##         Min          Q1         Med          Q3         Max
## -2.11532843 -0.69137140 -0.07308006  0.60554692  3.05603739
##
## Number of Observations: 748
## Number of Groups: 31
```

```
anova.lme(bond_anx_A_long1,type="marginal") # n.s.
```

```
##                 numDF denDF   F-value p-value
## (Intercept)         1   707  0.726077  0.3944
## bond_family         1   707  0.356226  0.5508
## bond_friend         1   707  0.656376  0.4181
## bond_country        1   707  2.061320  0.1515
## bond_humanity       1   707  2.407201  0.1212
## demo_age            1   707  8.015604  0.0048
## demo_gender         2   707 17.092241  <.0001
## demo_education      3   707  0.393473  0.7577
## GDP                 1    29  1.985939  0.1694
```

```
# Does bonding at T2 predict anxiety at T2?
summary(bond_anx_A_T2<- lme(anx_sum2_scaled ~ bond_family2 + bond_friend2 +
                                 bond_country2 + bond_humanity2  +
                                demo_age + demo_gender + demo_education + GDP,
                                (~1|Country_Name), data=DatasetA, na.action=na.exclude))
```

```
## Linear mixed-effects model fit by REML
##   Data: DatasetA
##        AIC      BIC    logLik
##   2030.876 2094.868 -1001.438
##
## Random effects:
##  Formula: ~1 | Country_Name
##         (Intercept)  Residual
## StdDev:   0.2586345 0.9208731
##
```

```
## Fixed effects:  anx_sum2_scaled ~ bond_family2 + bond_friend2 + bond_country2 +      bond_humanity2 +
##                                  Value Std.Error  DF   t-value p-value
## (Intercept)                  0.1792703 0.2123236 687  0.844326  0.3988
## bond_family2                -0.3034307 0.0885578 687 -3.426356  0.0006
## bond_friend2                -0.2956429 0.1652630 687 -1.788923  0.0741
## bond_country2               -0.4107107 0.1437900 687 -2.856323  0.0044
## bond_humanity2              -0.2591987 0.1114880 687 -2.324902  0.0204
## demo_age                    -0.0074495 0.0030390 687 -2.451320  0.0145
## demo_genderFemale            0.4214754 0.0731785 687  5.759550  0.0000
## demo_genderOther/non-binary  0.2104864 0.2866209 687  0.734372  0.4630
## demo_educationundergraduate -0.0281591 0.0962951 687 -0.292424  0.7701
## demo_educationpostgraduate  -0.0363649 0.1000409 687 -0.363500  0.7163
## demo_educationNA            -0.9451263 0.9716676  27 -0.972685  0.3393
## GDP                         -0.0000046 0.0000041  27 -1.127503  0.2695
##  Correlation:
##                             (Intr) bnd_fm2 bnd_fr2 bnd_c2 bnd_h2 demo_g dm_gnF
## bond_family2                -0.014
## bond_friend2                -0.071 -0.202
## bond_country2                0.041 -0.050  -0.179
## bond_humanity2              -0.099  0.035  -0.075  -0.199
## demo_age                    -0.461 -0.098   0.029  -0.058  0.045
## demo_genderFemale           -0.082 -0.087  -0.023   0.019 -0.014 -0.048
## demo_genderOther/non-binary -0.068  0.035   0.010   0.040 -0.007  0.041  0.137
## demo_educationundergraduate -0.273  0.086  -0.023   0.006  0.035 -0.035 -0.058
## demo_educationpostgraduate  -0.210  0.000   0.018   0.002 -0.051 -0.148 -0.069
## demo_educationNA            -0.151  0.018   0.013  -0.003  0.018  0.021 -0.051
## GDP                         -0.679 -0.013   0.061  -0.070  0.032 -0.044 -0.080
##                             dm_O/- dm_dctnn dm_dctnp dm_dNA
## bond_family2
## bond_friend2
## bond_country2
## bond_humanity2
## demo_age
## demo_genderFemale
## demo_genderOther/non-binary
## demo_educationundergraduate  0.000
## demo_educationpostgraduate   0.034  0.678
## demo_educationNA             0.002  0.068    0.064
## GDP                         -0.020 -0.026   -0.018    0.126
##
## Standardized Within-Group Residuals:
##         Min          Q1         Med          Q3         Max
## -2.03112089 -0.70607492 -0.08726521  0.59294398  2.91187647
##
## Number of Observations: 726
## Number of Groups: 30
```

```
anova.lme(bond_anx_A_T2,type="marginal") # all but friend sig.
```

```
##               numDF denDF   F-value p-value
## (Intercept)       1   686  0.712886  0.3988
## bond_family2      1   686 11.739913  0.0006
## bond_friend2      1   686  3.200246  0.0741
## bond_country2     1   686  8.158583  0.0044
```

```
## bond_humanity2     1   686  5.405170  0.0204
## demo_age           1   686  6.008968  0.0145
## demo_gender        2   686 16.587775  <.0001
## demo_education     3   686  0.346049  0.7920
## GDP                1    28  1.271263  0.2691
```

```r
# Does bonding at T1 predict depression at T2?
summary(bond_dep_A_long1<- lme(dep_sum2_scaled ~ bond_family + bond_friend +
                                  bond_country + bond_humanity +
                                 demo_age + demo_gender + demo_education + GDP,
                                 (~1|Country_Name), data=DatasetA, na.action=na.exclude))
```

```
## Linear mixed-effects model fit by REML
##   Data: DatasetA
##        AIC      BIC    logLik
##   2160.711 2225.148 -1066.356
##
## Random effects:
##  Formula: ~1 | Country_Name
##         (Intercept)  Residual
## StdDev:   0.3032615 0.9618497
##
## Fixed effects:  dep_sum2_scaled ~ bond_family + bond_friend + bond_country +      bond_humanity + de
##                                   Value Std.Error  DF    t-value p-value
## (Intercept)                   0.2422515 0.2282539 709  1.0613247  0.2889
## bond_family1                 -0.1299768 0.0893856 709 -1.4541127  0.1464
## bond_friend1                 -0.3123677 0.1374909 709 -2.2719153  0.0234
## bond_country1                -0.1173796 0.1344403 709 -0.8730986  0.3829
## bond_humanity1               -0.0602386 0.0943601 709 -0.6383908  0.5234
## demo_age                     -0.0002580 0.0031304 709 -0.0824101  0.9343
## demo_genderFemale             0.1570593 0.0755301 709  2.0794251  0.0379
## demo_genderOther/non-binary   0.2132181 0.2876238 709  0.7413090  0.4588
## demo_educationundergraduate -0.0551004 0.0990032 709 -0.5565515  0.5780
## demo_educationpostgraduate   -0.1879044 0.1029364 709 -1.8254404  0.0684
## demo_educationNA             -1.6219831 1.0253261  28 -1.5819193  0.1249
## GDP                          -0.0000088 0.0000045  28 -1.9543468  0.0607
##  Correlation:
##                              (Intr) bnd_fm1 bnd_fr1 bnd_c1 bnd_h1 demo_g dm_gnF
## bond_family1                 -0.054
## bond_friend1                 -0.021 -0.130
## bond_country1                -0.047 -0.088  -0.140
## bond_humanity1               -0.082 -0.067  -0.015  -0.264
## demo_age                     -0.438 -0.111   0.000   0.041  0.012
## demo_genderFemale            -0.083 -0.079  -0.071   0.004  0.018 -0.051
## demo_genderOther/non-binary -0.060  0.063  -0.063   0.011 -0.020  0.034  0.148
## demo_educationundergraduate -0.270  0.072  -0.018   0.031  0.019 -0.025 -0.054
## demo_educationpostgraduate   -0.221  0.041   0.021   0.057  0.010 -0.140 -0.075
## demo_educationNA             -0.157  0.025   0.009   0.008  0.015  0.020 -0.049
## GDP                          -0.690  0.047   0.017  -0.036  0.018 -0.048 -0.072
##                              dm_O/- dm_dctnn dm_dctnp dm_dNA
## bond_family1
## bond_friend1
## bond_country1
## bond_humanity1
```

```
## demo_age
## demo_genderFemale
## demo_genderOther/non-binary
## demo_educationundergraduate -0.008
## demo_educationpostgraduate   0.033  0.682
## demo_educationNA             0.000  0.067    0.067
## GDP                         -0.015 -0.021   -0.012    0.129
##
## Standardized Within-Group Residuals:
##        Min         Q1        Med         Q3        Max
## -1.9856065 -0.7822208 -0.1034837  0.6109664  3.5154751
##
## Number of Observations: 749
## Number of Groups: 31
```

```
anova.lme(bond_dep_A_long1,type="marginal") # only friend sig.
```

```
##                 numDF denDF  F-value p-value
## (Intercept)         1   708 1.126410  0.2889
## bond_family         1   708 2.114444  0.1464
## bond_friend         1   708 5.161599  0.0234
## bond_country        1   708 0.762301  0.3829
## bond_humanity       1   708 0.407543  0.5234
## demo_age            1   708 0.006791  0.9343
## demo_gender         2   708 2.258450  0.1053
## demo_education      3   708 2.148008  0.0929
## GDP                 1    29 3.819471  0.0604
```

```
# Does bonding at T2 predict depression at T2?
summary(bond_dep_A_T2<- lme(dep_sum2_scaled ~ bond_family2 + bond_friend2 +
                                  bond_country2 + bond_humanity2  +
                                 demo_age + demo_gender + demo_education + GDP,
                                 (~1|Country_Name), data=DatasetA, na.action=na.exclude))
```

```
## Linear mixed-effects model fit by REML
##   Data: DatasetA
##        AIC      BIC    logLik
##   2066.142 2130.154 -1019.071
##
## Random effects:
##  Formula: ~1 | Country_Name
##         (Intercept)  Residual
## StdDev:   0.2919815 0.9407698
##
## Fixed effects:  dep_sum2_scaled ~ bond_family2 + bond_friend2 + bond_country2 +      bond_humanity2 +
##                                   Value Std.Error  DF   t-value p-value
## (Intercept)                   0.2515219 0.2249097 688  1.118324  0.2638
## bond_family2                 -0.3170395 0.0904811 688 -3.503930  0.0005
## bond_friend2                 -0.3770811 0.1690111 688 -2.231102  0.0260
## bond_country2                -0.3170717 0.1471251 688 -2.155116  0.0315
## bond_humanity2               -0.3453257 0.1140246 688 -3.028520  0.0025
## demo_age                      0.0001776 0.0031103 688  0.057090  0.9545
## demo_genderFemale             0.1348065 0.0747445 688  1.803565  0.0717
```

```
## demo_genderOther/non-binary  0.0693960 0.2929691 688  0.236871  0.8128
## demo_educationundergraduate -0.0730560 0.0981211 688 -0.744549  0.4568
## demo_educationpostgraduate  -0.1577832 0.1020303 688 -1.546435  0.1225
## demo_educationNA            -1.6326835 1.0016230  27 -1.630038  0.1147
## GDP                         -0.0000072 0.0000044  27 -1.632522  0.1142
##  Correlation:
##                             (Intr) bnd_fm2 bnd_fr2 bnd_c2 bnd_h2 demo_g dm_gnF
## bond_family2                -0.014
## bond_friend2                -0.069 -0.202
## bond_country2                0.044 -0.051  -0.179
## bond_humanity2              -0.099  0.035  -0.075  -0.200
## demo_age                    -0.447 -0.098   0.029  -0.059  0.046
## demo_genderFemale           -0.079 -0.086  -0.023   0.020 -0.014 -0.047
## demo_genderOther/non-binary -0.064  0.034   0.010   0.040 -0.007  0.041  0.138
## demo_educationundergraduate -0.260  0.084  -0.023   0.004  0.035 -0.036 -0.055
## demo_educationpostgraduate  -0.200 -0.002   0.018   0.000 -0.051 -0.149 -0.067
## demo_educationNA            -0.157  0.018   0.013  -0.004  0.018  0.021 -0.050
## GDP                         -0.694 -0.011   0.058  -0.070  0.033 -0.039 -0.078
##                             dm_O/- dm_dctnn dm_dctnp dm_dNA
## bond_family2
## bond_friend2
## bond_country2
## bond_humanity2
## demo_age
## demo_genderFemale
## demo_genderOther/non-binary
## demo_educationundergraduate -0.001
## demo_educationpostgraduate   0.033  0.676
## demo_educationNA             0.001  0.066    0.063
## GDP                         -0.020 -0.026   -0.018    0.131
##
## Standardized Within-Group Residuals:
##         Min          Q1         Med          Q3         Max
## -2.13636911 -0.71996324 -0.08451817  0.55600696  3.82537514
##
## Number of Observations: 727
## Number of Groups: 30
```

```
anova.lme(bond_dep_A_T2,type="marginal") # all sig.
```

```
##                numDF denDF   F-value p-value
## (Intercept)        1   687  1.250648  0.2638
## bond_family2       1   687 12.277526  0.0005
## bond_friend2       1   687  4.977816  0.0260
## bond_country2      1   687  4.644526  0.0315
## bond_humanity2     1   687  9.171935  0.0025
## demo_age           1   687  0.003259  0.9545
## demo_gender        2   687  1.626502  0.1974
## demo_education     3   687  1.653166  0.1758
## GDP                1    28  2.665128  0.1138
```

Hypothesis 2b, Dataset A

```
# Does bonding at T1 predict anxiety at T2?
summary(bond_anx_A_long1<- lme(anx_sum2_scaled ~ as.factor(bond_mult) +
                                 demo_age + demo_gender + demo_education + GDP,
                                 (~1|Country_Name), data=DatasetA, na.action=na.exclude))
```

```
## Linear mixed-effects model fit by REML
##   Data: DatasetA
##        AIC      BIC    logLik
##   2118.445 2182.881 -1045.223
##
## Random effects:
##  Formula: ~1 | Country_Name
##         (Intercept) Residual
## StdDev:   0.2623008 0.938615
##
## Fixed effects:  anx_sum2_scaled ~ as.factor(bond_mult) + demo_age + demo_gender +      demo_educatio
##                                  Value Std.Error  DF   t-value p-value
## (Intercept)                  0.2311080 0.2148631 709  1.075606  0.2825
## as.factor(bond_mult)1       -0.3000517 0.0796993 709 -3.764797  0.0002
## as.factor(bond_mult)2       -0.1365592 0.1220302 709 -1.119060  0.2635
## as.factor(bond_mult)3       -0.1723805 0.2302690 709 -0.748605  0.4543
## as.factor(bond_mult)4       -0.5711572 0.3958641 709 -1.442811  0.1495
## demo_age                    -0.0085251 0.0030614 709 -2.784729  0.0055
## demo_genderFemale            0.4206078 0.0734126 709  5.729368  0.0000
## demo_genderOther/non-binary  0.2614137 0.2799340 709  0.933841  0.3507
## demo_educationundergraduate  0.0136748 0.0969956 709  0.140983  0.8879
## demo_educationpostgraduate  -0.0426133 0.1013444 709 -0.420480  0.6743
## demo_educationNA            -0.9544065 0.9898060  28 -0.964236  0.3432
## GDP                         -0.0000063 0.0000041  28 -1.527630  0.1378
##  Correlation:
##                             (Intr) a.(_)1 a.(_)2 a.(_)3 a.(_)4 demo_g dm_gnF
## as.factor(bond_mult)1       -0.168
## as.factor(bond_mult)2       -0.082  0.209
## as.factor(bond_mult)3       -0.062  0.115  0.072
## as.factor(bond_mult)4       -0.011  0.062  0.036  0.044
## demo_age                    -0.459  0.041  0.024 -0.037 -0.134
## demo_genderFemale           -0.088 -0.003 -0.054 -0.077 -0.020 -0.057
## demo_genderOther/non-binary -0.069  0.042 -0.029  0.012  0.002  0.041  0.149
## demo_educationundergraduate -0.275 -0.017  0.035  0.099  0.037 -0.028 -0.059
## demo_educationpostgraduate  -0.225  0.015 -0.019  0.129  0.077 -0.149 -0.080
## demo_educationNA            -0.151  0.032  0.020  0.022  0.014  0.022 -0.048
## GDP                         -0.671  0.033 -0.001  0.010  0.042 -0.052 -0.070
##                             dm_O/- dm_dctnn dm_dctnp dm_dNA
## as.factor(bond_mult)1
## as.factor(bond_mult)2
## as.factor(bond_mult)3
## as.factor(bond_mult)4
## demo_age
## demo_genderFemale
## demo_genderOther/non-binary
## demo_educationundergraduate -0.013
```

```
## demo_educationpostgraduate   0.035  0.685
## demo_educationNA             0.001  0.067    0.067
## GDP                         -0.015 -0.023   -0.009    0.123
##
## Standardized Within-Group Residuals:
##         Min          Q1         Med          Q3         Max
## -1.98354645 -0.69864013 -0.06402396  0.62504066  3.29469779
##
## Number of Observations: 749
## Number of Groups: 31
```

```
anova.lme(bond_anx_A_long1,type="marginal") # sig.
```

```
##                       numDF denDF   F-value p-value
## (Intercept)               1   708  1.156928  0.2825
## as.factor(bond_mult)      4   708  3.951311  0.0035
## demo_age                  1   708  7.754715  0.0055
## demo_gender               2   708 16.416225  <.0001
## demo_education            3   708  0.478868  0.6971
## GDP                       1    29  2.333654  0.1374
```

```
# Does bonding at T2 predict anxiety at T2?
summary(bond_anx_A_T2<- lme(anx_sum2_scaled ~ bond_mult2  +
                                 demo_age + demo_gender + demo_education + GDP,
                                 (~1|Country_Name), data=DatasetA, na.action=na.exclude))
```

```
## Linear mixed-effects model fit by REML
##   Data: DatasetA
##        AIC      BIC    logLik
##   2028.646 2092.658 -1000.323
##
## Random effects:
##  Formula: ~1 | Country_Name
##         (Intercept)  Residual
## StdDev:   0.2617966 0.9202083
##
## Fixed effects:  anx_sum2_scaled ~ bond_mult2 + demo_age + demo_gender + demo_education +      GDP
##                                  Value Std.Error  DF   t-value p-value
## (Intercept)                  0.2016755 0.2126872 688  0.948226  0.3433
## bond_mult21                 -0.3384643 0.0806552 688 -4.196436  0.0000
## bond_mult22                 -0.6084980 0.1445530 688 -4.209514  0.0000
## bond_mult23                 -0.7583247 0.2840143 688 -2.670023  0.0078
## bond_mult24                 -1.6050310 0.5371915 688 -2.987819  0.0029
## demo_age                    -0.0075547 0.0030209 688 -2.500783  0.0126
## demo_genderFemale            0.4193261 0.0729459 688  5.748454  0.0000
## demo_genderOther/non-binary  0.2137922 0.2863563 688  0.746595  0.4556
## demo_educationundergraduate -0.0323432 0.0962494 688 -0.336036  0.7369
## demo_educationpostgraduate  -0.0365665 0.1000779 688 -0.365380  0.7149
## demo_educationNA            -0.9604009 0.9720477  27 -0.988018  0.3319
## GDP                         -0.0000049 0.0000041  27 -1.192118  0.2436
##  Correlation:
##                             (Intr) bnd_21 bnd_22 bnd_23 bnd_24 demo_g dm_gnF
## bond_mult21                 -0.113
```

```
## bond_mult22                  -0.039  0.165
## bond_mult23                  -0.022  0.095  0.057
## bond_mult24                   0.001  0.043  0.030  0.009
## demo_age                     -0.453 -0.034 -0.035 -0.005 -0.069
## demo_genderFemale            -0.082 -0.029 -0.069 -0.038 -0.007 -0.055
## demo_genderOther/non-binary -0.073  0.050  0.028  0.019  0.006  0.045  0.138
## demo_educationundergraduate -0.278  0.087  0.008  0.032  0.035 -0.032 -0.053
## demo_educationpostgraduate  -0.215  0.024 -0.060 -0.013  0.036 -0.146 -0.064
## demo_educationNA            -0.153  0.030  0.016  0.008  0.006  0.021 -0.050
## GDP                         -0.677  0.001  0.009 -0.013  0.012 -0.051 -0.078
##                              dm_O/- dm_dctnn dm_dctnp dm_dNA
## bond_mult21
## bond_mult22
## bond_mult23
## bond_mult24
## demo_age
## demo_genderFemale
## demo_genderOther/non-binary
## demo_educationundergraduate  0.002
## demo_educationpostgraduate   0.034  0.682
## demo_educationNA             0.002  0.068    0.065
## GDP                         -0.017 -0.025   -0.018    0.126
##
## Standardized Within-Group Residuals:
##         Min          Q1         Med          Q3         Max
## -1.99847223 -0.72029237 -0.09198198  0.61447320  2.90287345
##
## Number of Observations: 727
## Number of Groups: 30
```

```
anova.lme(bond_anx_A_T2,type="marginal") # sig.
```

```
##                 numDF denDF   F-value p-value
## (Intercept)         1   687  0.899133  0.3433
## bond_mult2          4   687 10.563722  <.0001
## demo_age            1   687  6.253915  0.0126
## demo_gender         2   687 16.523350  <.0001
## demo_education      3   687  0.358330  0.7831
## GDP                 1    28  1.421146  0.2432
```

```
# Does bonding at T1 predict depression at T2?
summary(bond_dep_A_long1<- lme(dep_sum2_scaled ~ as.factor(bond_mult) +
                                 demo_age + demo_gender + demo_education + GDP,
                                 (~1|Country_Name), data=DatasetA, na.action=na.exclude))
```

```
## Linear mixed-effects model fit by REML
##   Data: DatasetA
##        AIC    BIC    logLik
##   2156.845 2221.3 -1064.423
##
## Random effects:
##  Formula: ~1 | Country_Name
##         (Intercept)  Residual
```

```
## StdDev:    0.302631 0.9599694
##
## Fixed effects:  dep_sum2_scaled ~ as.factor(bond_mult) + demo_age + demo_gender +      demo_educatio
##                                  Value Std.Error  DF   t-value p-value
## (Intercept)                  0.2990447 0.2290902 710  1.305358  0.1922
## as.factor(bond_mult)1       -0.2804223 0.0815233 710 -3.439781  0.0006
## as.factor(bond_mult)2       -0.2138583 0.1248363 710 -1.713109  0.0871
## as.factor(bond_mult)3       -0.1990072 0.2355912 710 -0.844714  0.3986
## as.factor(bond_mult)4       -0.6077650 0.4055112 710 -1.498763  0.1344
## demo_age                    -0.0005473 0.0031376 710 -0.174420  0.8616
## demo_genderFemale            0.1363725 0.0750913 710  1.816090  0.0698
## demo_genderOther/non-binary  0.1635923 0.2864732 710  0.571056  0.5681
## demo_educationundergraduate -0.0396854 0.0989977 710 -0.400871  0.6886
## demo_educationpostgraduate  -0.1779522 0.1035091 710 -1.719193  0.0860
## demo_educationNA            -1.6488420 1.0233842  28 -1.611166  0.1184
## GDP                         -0.0000089 0.0000045  28 -2.001083  0.0552
##  Correlation:
##                             (Intr) a.(_)1 a.(_)2 a.(_)3 a.(_)4 demo_g dm_gnF
## as.factor(bond_mult)1       -0.161
## as.factor(bond_mult)2       -0.078  0.208
## as.factor(bond_mult)3       -0.058  0.114  0.072
## as.factor(bond_mult)4       -0.012  0.062  0.035  0.044
## demo_age                    -0.443  0.041  0.025 -0.037 -0.134
## demo_genderFemale           -0.084 -0.002 -0.053 -0.077 -0.020 -0.055
## demo_genderOther/non-binary -0.065  0.042 -0.029  0.012  0.002  0.041  0.150
## demo_educationundergraduate -0.260 -0.020  0.033  0.098  0.037 -0.028 -0.056
## demo_educationpostgraduate  -0.214  0.013 -0.020  0.128  0.078 -0.151 -0.077
## demo_educationNA            -0.158  0.032  0.019  0.021  0.014  0.022 -0.048
## GDP                         -0.688  0.032 -0.001  0.008  0.041 -0.046 -0.068
##                             dm_O/- dm_dctnn dm_dctnp dm_dNA
## as.factor(bond_mult)1
## as.factor(bond_mult)2
## as.factor(bond_mult)3
## as.factor(bond_mult)4
## demo_age
## demo_genderFemale
## demo_genderOther/non-binary
## demo_educationundergraduate -0.013
## demo_educationpostgraduate   0.034  0.683
## demo_educationNA             0.001  0.065    0.066
## GDP                         -0.015 -0.023   -0.009    0.129
##
## Standardized Within-Group Residuals:
##         Min          Q1         Med          Q3         Max
## -1.92033140 -0.77583152 -0.07550935  0.59552600  3.67198537
##
## Number of Observations: 750
## Number of Groups: 31
```

```
anova.lme(bond_dep_A_long1,type="marginal") # sig.
```

```
##                      numDF denDF  F-value p-value
## (Intercept)              1   709 1.703958  0.1922
## as.factor(bond_mult)     4   709 3.650588  0.0059
```

```
## demo_age                  1   709 0.030422  0.8616
## demo_gender               2   709 1.694838  0.1844
## demo_education            3   709 2.140757  0.0938
## GDP                       1    29 4.004334  0.0548
```

```r
# Does bonding at T2 predict depression at T2?
summary(bond_dep_A_T2<- lme(dep_sum2_scaled ~ bond_mult2  +
                                 demo_age + demo_gender + demo_education + GDP,
                                 (~1|Country_Name), data=DatasetA, na.action=na.exclude))
```

```
## Linear mixed-effects model fit by REML
##   Data: DatasetA
##        AIC      BIC    logLik
##   2060.075 2124.107 -1016.038
##
## Random effects:
##  Formula: ~1 | Country_Name
##         (Intercept)  Residual
## StdDev:   0.2948066 0.9375283
##
## Fixed effects:  dep_sum2_scaled ~ bond_mult2 + demo_age + demo_gender + demo_education +      GDP
##                                   Value Std.Error  DF   t-value p-value
## (Intercept)                   0.2750547 0.2246716 689  1.224252  0.2213
## bond_mult21                  -0.4386254 0.0821489 689 -5.339396  0.0000
## bond_mult22                  -0.6333109 0.1473585 689 -4.297756  0.0000
## bond_mult23                  -0.6150879 0.2894713 689 -2.124867  0.0340
## bond_mult24                  -1.6034832 0.5474360 689 -2.929079  0.0035
## demo_age                      0.0003646 0.0030833 689  0.118267  0.9059
## demo_genderFemale             0.1304991 0.0743079 689  1.756194  0.0795
## demo_genderOther/non-binary   0.0564883 0.2918923 689  0.193524  0.8466
## demo_educationundergraduate  -0.0829089 0.0978014 689 -0.847727  0.3969
## demo_educationpostgraduate   -0.1650752 0.1017881 689 -1.621754  0.1053
## demo_educationNA             -1.6567931 0.9994896  27 -1.657639  0.1090
## GDP                          -0.0000072 0.0000044  27 -1.637336  0.1132
##  Correlation:
##                              (Intr) bnd_21 bnd_22 bnd_23 bnd_24 demo_g dm_gnF
## bond_mult21                  -0.108
## bond_mult22                  -0.038  0.165
## bond_mult23                  -0.020  0.095  0.057
## bond_mult24                   0.001  0.042  0.030  0.009
## demo_age                     -0.439 -0.034 -0.035 -0.005 -0.069
## demo_genderFemale            -0.079 -0.027 -0.068 -0.038 -0.006 -0.054
## demo_genderOther/non-binary -0.069  0.050  0.028  0.019  0.006  0.044  0.139
## demo_educationundergraduate -0.265  0.084  0.006  0.031  0.035 -0.033 -0.050
## demo_educationpostgraduate  -0.205  0.021 -0.061 -0.014  0.036 -0.147 -0.061
## demo_educationNA             -0.159  0.029  0.016  0.008  0.006  0.021 -0.050
## GDP                          -0.692  0.002  0.009 -0.012  0.011 -0.046 -0.076
##                              dm_O/- dm_dctnn dm_dctnp dm_dNA
## bond_mult21
## bond_mult22
## bond_mult23
## bond_mult24
## demo_age
## demo_genderFemale
```

```
## demo_genderOther/non-binary
## demo_educationundergraduate  0.000
## demo_educationpostgraduate   0.033  0.680
## demo_educationNA             0.002  0.067    0.063
## GDP                         -0.017 -0.025   -0.018    0.131
##
## Standardized Within-Group Residuals:
##         Min          Q1         Med          Q3         Max
## -2.17290301 -0.70408071 -0.08473208  0.58007190  3.79144066
##
## Number of Observations: 728
## Number of Groups: 30
```

```
anova.lme(bond_dep_A_T2,type="marginal") # sig.
```

```
##                 numDF denDF   F-value p-value
## (Intercept)         1   688  1.498794  0.2213
## bond_mult2          4   688 12.390876  <.0001
## demo_age            1   688  0.013987  0.9059
## demo_gender         2   688  1.543376  0.2144
## demo_education      3   688  1.738476  0.1578
## GDP                 1    28  2.680870  0.1128
```

Table S13B: Longitudinal comparisons, Dataset B

Dataset B had 6 time-points, with the distancing and wellbeing questions repeated at all time-points. Using these data, we examined whether the findings held at a second time-point and whether bonding at T1 predicted the variables of interest at T2. To keep the times as similar as possible between the two datasets, we used phase 6 of Dataset B, which was closest to the T2 of of Dataset A. Dataset B, phase 6 dates: 22nd June 2020 - 13th August 2020

Hypothesis 1a, Dataset B

```
# Does bonding at T1 predict distancing at T2?
summary(bond_distancing_B_long1<- lme(comply_self6 ~ bond_country1 + bond_gvmt1 +
                                 age1 + gender1 + education1 + GDP1,
                                 (~1|country_now_Name1), data=DatasetB_wide, na.action=na.exclude))
```

```
## Linear mixed-effects model fit by REML
##   Data: DatasetB_wide
##        AIC      BIC    logLik
##   10323.38 10383.75 -5149.689
##
## Random effects:
##  Formula: ~1 | country_now_Name1
##         (Intercept) Residual
## StdDev:    3.341959 22.13431
##
## Fixed effects:  comply_self6 ~ bond_country1 + bond_gvmt1 + age1 + gender1 +      education1 + GDP1
##                              Value Std.Error   DF   t-value p-value
```

```
## (Intercept)              46.63116  3.276791 1068 14.230737  0.0000
## bond_country11            2.68876  1.759776 1068  1.527896  0.1268
## bond_gvmt11              -1.98850  3.029813 1068 -0.656312  0.5118
## age1                      0.14694  0.044450 1068  3.305843  0.0010
## gender1woman              3.45818  1.559631 1068  2.217310  0.0268
## gender1nb                15.66307  8.539580 1068  1.834174  0.0669
## gender1none              17.79441  9.161655 1068  1.942271  0.0524
## education1undergraduate   3.55947  2.230677 1068  1.595690  0.1109
## education1postgraduate    5.73908  2.195147 1068  2.614441  0.0091
## GDP1                      0.00002  0.000054   63  0.296133  0.7681
##  Correlation:
##                          (Intr) bnd_c11 bnd_g11 age1   gndr1w gndr1nb gndr1nn
## bond_country11           -0.027
## bond_gvmt11              -0.053 -0.355
## age1                     -0.389 -0.230   0.002
## gender1woman             -0.343 -0.064   0.080   0.137
## gender1nb                -0.068  0.013   0.015   0.085  0.153
## gender1none              -0.075  0.018   0.013   0.051  0.131  0.029
## education1undergraduate -0.416  0.068   0.022  -0.206 -0.017 -0.033   0.001
## education1postgraduate  -0.399  0.105   0.023  -0.240 -0.046 -0.052  -0.008
## GDP1                    -0.502  0.056  -0.039  -0.074 -0.091 -0.031  -0.011
##                          edctn1n edctn1p
## bond_country11
## bond_gvmt11
## age1
## gender1woman
## gender1nb
## gender1none
## education1undergraduate
## education1postgraduate   0.791
## GDP1                     0.007  -0.014
##
## Standardized Within-Group Residuals:
##        Min         Q1        Med         Q3        Max
## -2.6886818 -0.7070303 -0.1693726  0.7834346  2.3316884
##
## Number of Observations: 1141
## Number of Groups: 65
```

```
anova.lme(bond_distancing_B_long1,type="marginal") # n.s.
```

```
##                 numDF denDF   F-value p-value
## (Intercept)         1  1068 202.51387  <.0001
## bond_country1       1  1068   2.33447  0.1268
## bond_gvmt1          1  1068   0.43074  0.5118
## age1                1  1068  10.92860  0.0010
## gender1             3  1068   3.31145  0.0195
## education1          2  1068   3.71444  0.0247
## GDP1                1    63   0.08770  0.7681
```

```
# Does bonding at T2 predict distancing at T2?
summary(bond_distancing_A_T2<- lme(comply_self_scale6 ~ bond_country6 + bond_gvmt6 +
                                  age6 + gender6 + education6 + GDP6,
                                  (~1|country_now_Name6), data=DatasetB_wide, na.action=na.exclude))
```

```
## Linear mixed-effects model fit by REML
##   Data: DatasetB_wide
##        AIC      BIC    logLik
##   3341.377 3401.747 -1658.688
##
## Random effects:
##  Formula: ~1 | country_now_Name6
##         (Intercept) Residual
## StdDev:   0.1300067 1.011416
##
## Fixed effects:  comply_self_scale6 ~ bond_country6 + bond_gvmt6 + age6 + gender6 +      education6 +
##                               Value Std.Error   DF   t-value p-value
## (Intercept)              -0.6809539 0.1475629 1068 -4.614669  0.0000
## bond_country61            0.1178050 0.0803156 1068  1.466776  0.1427
## bond_gvmt61              -0.0844754 0.1381252 1068 -0.611586  0.5409
## age6                      0.0067359 0.0020316 1068  3.315561  0.0009
## gender6woman              0.1604292 0.0711880 1068  2.253598  0.0244
## gender6nb                 0.7228507 0.3901497 1068  1.852752  0.0642
## gender6none               0.8086851 0.4185986 1068  1.931887  0.0536
## education6undergraduate  0.1614564 0.1019027 1068  1.584417  0.1134
## education6postgraduate   0.2653727 0.1001092 1068  2.650833  0.0081
## GDP6                     -0.0000008 0.0000024   63 -0.333814  0.7396
##  Correlation:
##                          (Intr) bnd_c61 bnd_g61 age6   gndr6w gndr6nb gndr6nn
## bond_country61           -0.022
## bond_gvmt61              -0.050 -0.355
## age6                     -0.389 -0.232   0.005
## gender6woman             -0.348 -0.064   0.081   0.138
## gender6nb                -0.067  0.012   0.016   0.086  0.153
## gender6none              -0.077  0.018   0.014   0.051  0.131  0.029
## education6undergraduate -0.432  0.069   0.021  -0.210 -0.019 -0.033   0.000
## education6postgraduate  -0.410  0.107   0.022  -0.243 -0.047 -0.052  -0.007
## GDP6                     -0.498  0.050  -0.039  -0.092 -0.092 -0.035  -0.012
##                          edctn6n edctn6p
## bond_country61
## bond_gvmt61
## age6
## gender6woman
## gender6nb
## gender6none
## education6undergraduate
## education6postgraduate   0.791
## GDP6                      0.031   0.001
##
## Standardized Within-Group Residuals:
##        Min         Q1        Med         Q3        Max
## -2.7083515 -0.7131679 -0.1759162  0.7778670  2.3298018
##
## Number of Observations: 1141
## Number of Groups: 65
```

```r
anova.lme(bond_distancing_A_T2,type="marginal") # n.s.
```

```
##                 numDF denDF   F-value p-value
## (Intercept)         1  1068 21.295166  <.0001
## bond_country6       1  1068  2.151432  0.1427
## bond_gvmt6          1  1068  0.374038  0.5409
## age6                1  1068 10.992944  0.0009
## gender6             3  1068  3.361960  0.0182
## education6          2  1068  3.863745  0.0213
## GDP6                1    63  0.111432  0.7396
```

Hypothesis 1b, Dataset B

```r
# Does bonding at T1 predict distancing at T2?
summary(bond_mult_distancing_B_long1<- lme(comply_self6 ~ bond_mult1 +
                                 age1 + gender1 + education1 + GDP1,
                                 (~1|country_now_Name1), data=DatasetB_wide, na.action=na.exclude))
```

```
## Linear mixed-effects model fit by REML
##   Data: DatasetB_wide
##        AIC      BIC    logLik
##   10465.32 10525.86 -5220.661
##
## Random effects:
##  Formula: ~1 | country_now_Name1
##         (Intercept) Residual
## StdDev:    3.172252 22.11563
##
## Fixed effects:  comply_self6 ~ bond_mult1 + age1 + gender1 + education1 + GDP1
##                              Value Std.Error   DF   t-value p-value
## (Intercept)               46.83531  3.239077 1084 14.459459  0.0000
## bond_mult11                1.81279  1.738587 1084  1.042682  0.2973
## bond_mult12                1.34918  3.101682 1084  0.434982  0.6637
## age1                       0.14831  0.044126 1084  3.360999  0.0008
## gender1woman               3.65267  1.546915 1084  2.361259  0.0184
## gender1nb                 15.81096  8.529252 1084  1.853733  0.0640
## gender1none               17.87262  9.152244 1084  1.952813  0.0511
## education1undergraduate    3.22453  2.212497 1084  1.457418  0.1453
## education1postgraduate     5.41919  2.175778 1084  2.490690  0.0129
## GDP1                       0.00001  0.000053   63  0.282716  0.7783
##  Correlation:
##                           (Intr) bnd_11 bnd_12 age1   gndr1w gndr1nb gndr1nn
## bond_mult11               -0.022
## bond_mult12               -0.075  0.139
## age1                      -0.394 -0.222 -0.124
## gender1woman              -0.346 -0.066  0.052  0.135
## gender1nb                 -0.068  0.016  0.020  0.084  0.151
## gender1none               -0.075  0.020  0.023  0.051  0.130  0.029
## education1undergraduate -0.419  0.059  0.065 -0.206 -0.014 -0.032   0.001
## education1postgraduate  -0.402  0.099  0.086 -0.239 -0.042 -0.051  -0.007
## GDP1                      -0.500  0.022  0.020 -0.070 -0.089 -0.031  -0.011
```

```
##                           edctn1n edctn1p
## bond_mult11
## bond_mult12
## age1
## gender1woman
## gender1nb
## gender1none
## education1undergraduate
## education1postgraduate   0.790
## GDP1                     0.008  -0.014
##
## Standardized Within-Group Residuals:
##        Min         Q1        Med         Q3        Max
## -2.6959656 -0.7112749 -0.1720689  0.7933435  2.3232307
##
## Number of Observations: 1157
## Number of Groups: 65
```

```
anova.lme(bond_mult_distancing_B_long1,type="marginal") # n.s.
```

```
##             numDF denDF   F-value p-value
## (Intercept)     1  1084 209.07597  <.0001
## bond_mult1      2  1084   0.58657  0.5564
## age1            1  1084  11.29632  0.0008
## gender1         3  1084   3.52471  0.0146
## education1      2  1084   3.44905  0.0321
## GDP1            1    63   0.07993  0.7783
```

```
# Does bonding at T2 predict distancing at T2?
summary(bond_distancing_A_T2<- lme(comply_self_scale6 ~ bond_mult6 +
                                 age6 + gender6 + education6 + GDP6,
                                 (~1|country_now_Name6), data=DatasetB_wide, na.action=na.exclude))
```

```
## Linear mixed-effects model fit by REML
##   Data: DatasetB_wide
##        AIC      BIC    logLik
##   3384.278 3444.816 -1680.139
##
## Random effects:
##  Formula: ~1 | country_now_Name6
##         (Intercept) Residual
## StdDev:   0.1274079 1.010222
##
## Fixed effects:  comply_self_scale6 ~ bond_mult6 + age6 + gender6 + education6 +      GDP6
##                              Value Std.Error   DF   t-value p-value
## (Intercept)             -0.6823947 0.1461475 1084 -4.669219  0.0000
## bond_mult61              0.0816529 0.0793491 1084  1.029035  0.3037
## bond_mult62              0.0589586 0.1415416 1084  0.416546  0.6771
## age6                     0.0067594 0.0020162 1084  3.352532  0.0008
## gender6woman             0.1685115 0.0705977 1084  2.386925  0.0172
## gender6nb                0.7265645 0.3895723 1084  1.865031  0.0624
## gender6none              0.8118185 0.4180385 1084  1.941971  0.0524
## education6undergraduate  0.1470093 0.1010595 1084  1.454680  0.1460
```

```
## education6postgraduate   0.2516434 0.0992170 1084  2.536294  0.0113
## GDP6                    -0.0000005 0.0000024   63 -0.199595  0.8424
##  Correlation:
##                         (Intr) bnd_61 bnd_62 age6   gndr6w gndr6nb gndr6nn
## bond_mult61             -0.018
## bond_mult62             -0.072  0.138
## age6                    -0.393 -0.221 -0.123
## gender6woman            -0.350 -0.065  0.053  0.136
## gender6nb               -0.067  0.016  0.020  0.085  0.151
## gender6none             -0.077  0.020  0.023  0.051  0.130  0.029
## education6undergraduate -0.434  0.059  0.065 -0.210 -0.016 -0.033   0.001
## education6postgraduate  -0.412  0.099  0.087 -0.241 -0.043 -0.052  -0.007
## GDP6                    -0.495  0.020  0.018 -0.086 -0.090 -0.035  -0.012
##                         edctn6n edctn6p
## bond_mult61
## bond_mult62
## age6
## gender6woman
## gender6nb
## gender6none
## education6undergraduate
## education6postgraduate   0.790
## GDP6                     0.031  -0.001
##
## Standardized Within-Group Residuals:
##        Min         Q1        Med         Q3        Max
## -2.7111578 -0.7146908 -0.1737665  0.7827404  2.3214819
##
## Number of Observations: 1157
## Number of Groups: 65
```

```
anova.lme(bond_distancing_A_T2,type="marginal") # n.s.
```

```
##             numDF denDF   F-value p-value
## (Intercept)     1  1084 21.801607  <.0001
## bond_mult6      2  1084  0.567880  0.5669
## age6            1  1084 11.239473  0.0008
## gender6         3  1084  3.557100  0.0139
## education6      2  1084  3.619520  0.0271
## GDP6            1    63  0.039838  0.8424
```

Hypothesis 2a, Dataset B

```
# Does bonding at T1 predict wellbeing at T2?
summary(bond_wellbeing_B_long1<- lme(wellbeing_scale6 ~ bond_country1 + bond_gvmt1 +
                                 age1 + gender1 + education1 + GDP1,
                                 (~1|country_now_Name1), data=DatasetB_wide, na.action=na.exclude))
```

```
## Linear mixed-effects model fit by REML
##   Data: DatasetB_wide
##        AIC      BIC    logLik
```

```
##   3300.664 3361.034 -1638.332
##
## Random effects:
##  Formula: ~1 | country_now_Name1
##         (Intercept)  Residual
## StdDev:   0.1484089 0.9925675
##
## Fixed effects:  wellbeing_scale6 ~ bond_country1 + bond_gvmt1 + age1 + gender1 +      education1 + GD
##                              Value Std.Error   DF   t-value p-value
## (Intercept)             -0.5626264 0.1467916 1068 -3.832824  0.0001
## bond_country11           0.3488808 0.0789089 1068  4.421312  0.0000
## bond_gvmt11              0.2828770 0.1358521 1068  2.082243  0.0376
## age1                     0.0099653 0.0019930 1068  5.000209  0.0000
## gender1woman            -0.0053721 0.0699338 1068 -0.076818  0.9388
## gender1nb               -0.0590922 0.3829304 1068 -0.154316  0.8774
## gender1none             -0.7753480 0.4108322 1068 -1.887262  0.0594
## education1undergraduate  0.0987072 0.1000244 1068  0.986830  0.3239
## education1postgraduate   0.2535569 0.0984265 1068  2.576105  0.0101
## GDP1                    -0.0000001 0.0000024   63 -0.043875  0.9651
##  Correlation:
##                         (Intr) bnd_c11 bnd_g11 age1   gndr1w gndr1nb gndr1nn
## bond_country11          -0.027
## bond_gvmt11             -0.053 -0.355
## age1                    -0.390 -0.230   0.002
## gender1woman            -0.343 -0.064   0.080   0.137
## gender1nb               -0.068  0.013   0.015   0.085  0.153
## gender1none             -0.075  0.018   0.013   0.051  0.131  0.029
## education1undergraduate -0.417  0.068   0.022  -0.206 -0.017 -0.033   0.001
## education1postgraduate  -0.399  0.105   0.023  -0.240 -0.046 -0.052  -0.007
## GDP1                    -0.501  0.056  -0.039  -0.074 -0.091 -0.031  -0.011
##                         edctn1n edctn1p
## bond_country11
## bond_gvmt11
## age1
## gender1woman
## gender1nb
## gender1none
## education1undergraduate
## education1postgraduate   0.791
## GDP1                     0.007  -0.014
##
## Standardized Within-Group Residuals:
##         Min          Q1         Med          Q3         Max
## -3.71096940 -0.63173217 -0.03223431  0.55301909  3.55932361
##
## Number of Observations: 1141
## Number of Groups: 65
```

```
anova.lme(bond_wellbeing_B_long1,type="marginal") # sig.
```

```
##                numDF denDF   F-value p-value
## (Intercept)        1  1068 14.690539  0.0001
## bond_country1      1  1068 19.548001  <.0001
## bond_gvmt1         1  1068  4.335736  0.0376
```

```
## age1               1  1068 25.002088  <.0001
## gender1            3  1068  1.202485  0.3076
## education1         2  1068  4.789623  0.0085
## GDP1               1    63  0.001925  0.9651
```

```r
# Does bonding at T2 predict wellbeing at T2?
summary(bond_wellbeing_A_T2<- lme(wellbeing_scale6 ~ bond_country6 + bond_gvmt6 +
                                 age6 + gender6 + education6 + GDP6,
                                 (~1|country_now_Name6), data=DatasetB_wide, na.action=na.exclude))
```

```
## Linear mixed-effects model fit by REML
##   Data: DatasetB_wide
##        AIC      BIC    logLik
##   3298.126 3358.497 -1637.063
##
## Random effects:
##  Formula: ~1 | country_now_Name6
##         (Intercept) Residual
## StdDev:    0.169266 0.990542
##
## Fixed effects:  wellbeing_scale6 ~ bond_country6 + bond_gvmt6 + age6 + gender6 +      education6 + GI
##                              Value Std.Error   DF   t-value p-value
## (Intercept)             -0.5482271 0.1488422 1068 -3.683277  0.0002
## bond_country61           0.3471094 0.0787934 1068  4.405312  0.0000
## bond_gvmt61              0.2858932 0.1356248 1068  2.107971  0.0353
## age6                     0.0103338 0.0019984 1068  5.171130  0.0000
## gender6woman             0.0007251 0.0698660 1068  0.010379  0.9917
## gender6nb               -0.0348193 0.3823940 1068 -0.091056  0.9275
## gender6none             -0.7660914 0.4100601 1068 -1.868242  0.0620
## education6undergraduate  0.0948787 0.0999555 1068  0.949209  0.3427
## education6postgraduate   0.2513534 0.0983387 1068  2.555998  0.0107
## GDP6                    -0.0000006 0.0000026   63 -0.241553  0.8099
##  Correlation:
##                         (Intr) bnd_c61 bnd_g61 age6   gndr6w gndr6nb gndr6nn
## bond_country61          -0.028
## bond_gvmt61             -0.050 -0.354
## age6                    -0.370 -0.231   0.005
## gender6woman            -0.340 -0.063   0.081   0.139
## gender6nb               -0.066  0.013   0.017   0.087  0.154
## gender6none             -0.073  0.018   0.013   0.052  0.131  0.029
## education6undergraduate -0.422  0.070   0.021  -0.209 -0.019 -0.034   0.000
## education6postgraduate  -0.402  0.107   0.022  -0.241 -0.047 -0.053  -0.008
## GDP6                    -0.511  0.054  -0.044  -0.093 -0.085 -0.032  -0.012
##                         edctn6n edctn6p
## bond_country61
## bond_gvmt61
## age6
## gender6woman
## gender6nb
## gender6none
## education6undergraduate
## education6postgraduate   0.791
## GDP6                     0.027   0.002
##
```

```
## Standardized Within-Group Residuals:
##         Min          Q1         Med          Q3         Max
## -3.71523053 -0.62559777 -0.03239688  0.55323578  3.58719488
##
## Number of Observations: 1141
## Number of Groups: 65
```

```
anova.lme(bond_wellbeing_A_T2,type="marginal") # sig.
```

```
##                 numDF denDF   F-value p-value
## (Intercept)         1  1068 13.566531  0.0002
## bond_country6       1  1068 19.406773  <.0001
## bond_gvmt6          1  1068  4.443541  0.0353
## age6                1  1068 26.740583  <.0001
## gender6             3  1068  1.187572  0.3133
## education6          2  1068  4.804266  0.0084
## GDP6                1    63  0.058348  0.8099
```

Hypothesis 2b, Dataset B

```
# Does bonding at T1 predict wellbeing at T2?
summary(bond_mult_wellbeing_B_long1<- lme(wellbeing_scale6 ~ bond_mult1 +
                                 age1 + gender1 + education1 + GDP1,
                                 (~1|country_now_Name1), data=DatasetB_wide, na.action=na.exclude))
```

```
## Linear mixed-effects model fit by REML
##   Data: DatasetB_wide
##       AIC      BIC    logLik
##   3342.71 3403.249 -1659.355
##
## Random effects:
##  Formula: ~1 | country_now_Name1
##         (Intercept) Residual
## StdDev:   0.1425094 0.991422
##
## Fixed effects:  wellbeing_scale6 ~ bond_mult1 + age1 + gender1 + education1 +      GDP1
##                               Value Std.Error   DF   t-value p-value
## (Intercept)              -0.5398583 0.1452361 1084 -3.717107  0.0002
## bond_mult11               0.3509370 0.0779401 1084  4.502649  0.0000
## bond_mult12               0.6099457 0.1390473 1084  4.386607  0.0000
## age1                      0.0099784 0.0019782 1084  5.044209  0.0000
## gender1woman             -0.0133083 0.0693476 1084 -0.191908  0.8479
## gender1nb                -0.0644223 0.3823600 1084 -0.168486  0.8662
## gender1none              -0.7815867 0.4102868 1084 -1.904976  0.0570
## education1undergraduate   0.0820743 0.0991852 1084  0.827485  0.4081
## education1postgraduate    0.2420010 0.0975402 1084  2.481039  0.0133
## GDP1                     -0.0000003 0.0000024   63 -0.108817  0.9137
##  Correlation:
##                          (Intr) bnd_11 bnd_12 age1   gndr1w gndr1nb gndr1nn
## bond_mult11              -0.022
## bond_mult12              -0.075  0.139
```

```
## age1                       -0.394 -0.222 -0.124
## gender1woman               -0.345 -0.066  0.052  0.135
## gender1nb                  -0.068  0.016  0.020  0.084  0.151
## gender1none                -0.075  0.020  0.023  0.051  0.130  0.029
## education1undergraduate -0.419  0.059  0.065 -0.206 -0.014 -0.032   0.001
## education1postgraduate  -0.402  0.099  0.086 -0.239 -0.042 -0.051  -0.007
## GDP1                       -0.500  0.022  0.020 -0.070 -0.088 -0.031  -0.011
##                            edctn1n edctn1p
## bond_mult11
## bond_mult12
## age1
## gender1woman
## gender1nb
## gender1none
## education1undergraduate
## education1postgraduate   0.790
## GDP1                      0.008  -0.014
##
## Standardized Within-Group Residuals:
##         Min          Q1         Med          Q3         Max
## -3.71538569 -0.63812780 -0.03209936  0.55708120  3.56898125
##
## Number of Observations: 1157
## Number of Groups: 65
```

```
anova.lme(bond_mult_wellbeing_B_long1,type="marginal") # sig.
```

```
##             numDF denDF   F-value p-value
## (Intercept)     1  1084 13.816887  0.0002
## bond_mult1      2  1084 17.351927  <.0001
## age1            1  1084 25.444045  <.0001
## gender1         3  1084  1.215811  0.3027
## education1      2  1084  4.786913  0.0085
## GDP1            1    63  0.011841  0.9137
```

```
# Does bonding at T2 predict wellbeing at T2?
summary(bond_mult_wellbeing_A_T2<- lme(wellbeing_scale6 ~ bond_mult6 +
                                 age6 + gender6 + education6 + GDP6,
                                 (~1|country_now_Name6), data=DatasetB_wide, na.action=na.exclude))
```

```
## Linear mixed-effects model fit by REML
##   Data: DatasetB_wide
##        AIC      BIC    logLik
##   3339.702 3400.241 -1657.851
##
## Random effects:
##  Formula: ~1 | country_now_Name6
##         (Intercept)  Residual
## StdDev:   0.1690392 0.9889738
##
## Fixed effects:  wellbeing_scale6 ~ bond_mult6 + age6 + gender6 + education6 +      GDP6
##                                 Value Std.Error   DF   t-value p-value
## (Intercept)             -0.5208130 0.1476877 1084 -3.526447  0.0004
```

```
## bond_mult61               0.3500132 0.0778166 1084  4.497926  0.0000
## bond_mult62               0.6107302 0.1388308 1084  4.399097  0.0000
## age6                      0.0103731 0.0019828 1084  5.231658  0.0000
## gender6woman             -0.0063061 0.0692619 1084 -0.091047  0.9275
## gender6nb                -0.0364167 0.3816906 1084 -0.095409  0.9240
## gender6none              -0.7717890 0.4093526 1084 -1.885390  0.0596
## education6undergraduate   0.0775190 0.0990942 1084  0.782276  0.4342
## education6postgraduate    0.2394465 0.0974400 1084  2.457374  0.0142
## GDP6                     -0.0000010 0.0000025   63 -0.397391  0.6924
##  Correlation:
##                          (Intr) bnd_61 bnd_62 age6   gndr6w gndr6nb gndr6nn
## bond_mult61              -0.022
## bond_mult62              -0.075  0.139
## age6                     -0.373 -0.220 -0.124
## gender6woman             -0.341 -0.064  0.053  0.137
## gender6nb                -0.066  0.017  0.020  0.086  0.152
## gender6none              -0.072  0.020  0.023  0.052  0.131  0.029
## education6undergraduate -0.422  0.059  0.065 -0.209 -0.016 -0.034   0.000
## education6postgraduate  -0.404  0.100  0.087 -0.239 -0.043 -0.052  -0.008
## GDP6                     -0.510  0.018  0.017 -0.087 -0.083 -0.032  -0.013
##                          edctn6n edctn6p
## bond_mult61
## bond_mult62
## age6
## gender6woman
## gender6nb
## gender6none
## education6undergraduate
## education6postgraduate   0.791
## GDP6                     0.027   0.000
##
## Standardized Within-Group Residuals:
##         Min          Q1         Med          Q3         Max
## -3.72104276 -0.62802173 -0.03803993  0.55584633  3.60014205
##
## Number of Observations: 1157
## Number of Groups: 65
```

```
anova.lme(bond_mult_wellbeing_A_T2,type="marginal") # sig.
```

```
##             numDF denDF   F-value p-value
## (Intercept)     1  1084 12.435828  0.0004
## bond_mult6      2  1084 17.376981  <.0001
## age6            1  1084 27.370244  <.0001
## gender6         3  1084  1.194467  0.3106
## education6      2  1084  4.814626  0.0083
## GDP6            1    63  0.157920  0.6924
```
